# Mechano-growth factor E-domain modulates cardiac contractile function through 14-3-3 protein interactomes

**DOI:** 10.3389/fphys.2022.1028345

**Published:** 2022-11-16

**Authors:** Christopher Solís, Walter C. Thompson, James R. Peña, Christopher McDermott-Roe, Paulina Langa, Chad M. Warren, Magdalena Chrzanowska, Beata M. Wolska, R. John Solaro, Pieter Detombe, Paul H. Goldspink

**Affiliations:** ^1^ Department of Physiology and Biophysics, University of Illinois Chicago, Chicago, IL, United States; ^2^ Department of Physiology, Medical College of Wisconsin, Milwaukee, WI, United States; ^3^ Department of Medicine, and Department of Genetics, Perelman School of Medicine, Cardiovascular Institute, University of Pennsylvania, Philadelphia, PA, United States; ^4^ Center for Cardiovascular Research, University of Illinois at Chicago, Chicago, IL, United States; ^5^ Blood Research Institute, Versiti, Department of Pharmacology and Toxicology, Cardiovascular Center, Medical College of Wisconsin, Milwaukee, WI, United States; ^6^ Department of Medicine, Division of Cardiology, University of Illinois at Chicago, Chicago, IL, United States; ^7^ Phymedexp, Université de Montpellier, Inserm, CNRS, Montpellier, France

**Keywords:** mechano-growth factor, E-domain peptide, 14-3-3 protein, contractile function of the heart, protein-protein interaction (PPI)

## Abstract

In the heart, alternative splicing of the *igf-I* gene produces two isoforms: IGF-IEa and IGF-IEc, (Mechano-growth factor, MGF). The sequence divergence between their E-domain regions suggests differential isoform function. To define the biological actions of MGF’s E-domain, we performed *in silico* analysis of the unique C-terminal sequence and identified a phosphorylation consensus site residing within a putative 14-3-3 binding motif. To test the functional significance of Ser 18 phosphorylation, phospho-mimetic (S/E^18^) and phospho-null (S/A^18^) peptides were delivered to mice at different doses for 2 weeks. Cardiovascular function was measured using echocardiography and a pressure-volume catheter. At the lowest (2.25 mg/kg/day) and highest (9 mg/kg/day) doses, the peptides produced a depression in systolic and diastolic parameters. However, at 4.5 mg/kg/day the peptides produced opposing effects on cardiac function. Fractional shortening analysis also showed a similar trend, but with no significant change in cardiac geometry. Microarray analysis discovered 21 genes (FDR *p* < 0.01), that were expressed accordant with the opposing effects on contractile function at 4.5 mg/kg/day, with the nuclear receptor subfamily 4 group A member 2 (*Nr4a2*) identified as a potential target of peptide regulation. Testing the regulation of the Nr4a family, showed the E-domain peptides modulate *Nr4a* gene expression following membrane depolarization with KCl *in vitro*. To determine the potential role of 14-3-3 proteins, we examined 14-3-3 isoform expression and distribution. 14-3-3γ localized to the myofilaments in neonatal cardiac myocytes, the cardiac myocytes and myofilament extracts from the adult heart. Thermal shift analysis of recombinant 14-3-3γ protein showed the S/A^18^ peptide destabilized 14-3-3γ folding. Also, the S/A^18^ peptide significantly inhibited 14-3-3γ’s ability to interact with myosin binding protein C (MYPC3) and phospholamban (PLN) in heart lysates from dobutamine injected mice. Conversely, the S/E^18^ peptide showed no effect on 14-3-3γ stability, did not inhibit 14-3-3γ’s interaction with PLN but did inhibit the interaction with MYPC3. Replacing the glutamic acid with a phosphate group on Ser 18 (pSer^18^), significantly increased 14-3-3γ protein stability. We conclude that the state of Ser 18 phosphorylation within the 14-3-3 binding motif of MGF’s E-domain, modulates protein-protein interactions within the 14-3-3γ interactome, which includes proteins involved in the regulation of contractile function.

## Introduction

**FIGURE 1:**
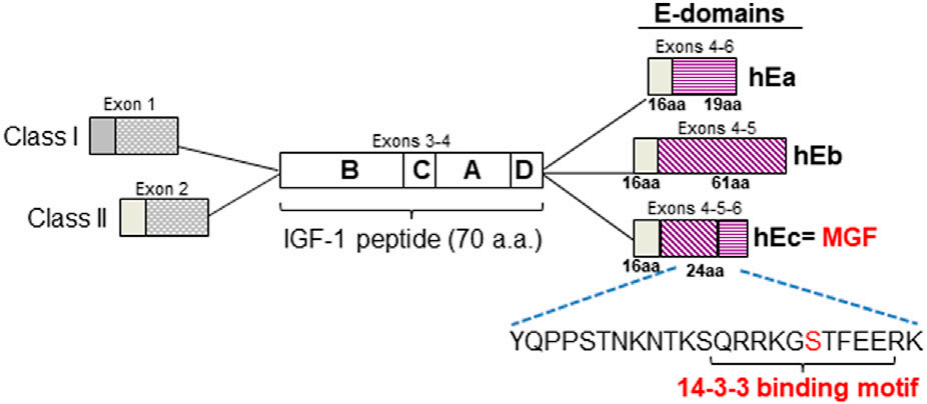
Human IGF-1 isoforms. Alternative splicing of the IGF-1 pre-mRNA produces three isoforms which encode prepropeptides containing a signal peptide, the conserved 70 a.a IGF-1 peptide and different E-domain regions in which the first 16 a.a are conserved. The sequence of the unique C-terminal 24 a.a of the MGF isoform E-domain is shown. The putative phosphoserine (Ser^18^) site (shown in red) resides within the putative 14-3-3 binding motif as depicted.

Insulin-like growth factor-1 (IGF-1) is a major homeostatic regulator of physiologic growth and protector against cellular stress in the heart (Weeks et al., 2017). IGF-1 belongs to the insulin/IGF/relaxin superfamily of proteins which are composed of polypeptide domains denoted as A, B, and C domains, although some also contain D and E domains (Kasik et al., 2000). The IGFs evolved through gene duplication and undergo alternative splicing to yield different prepropeptide isoforms. In humans alternative splicing of *igf-1* at the 5′-end (exons 1 or 2 to exon 3), yields prepropeptides with different signal peptides. Each prepropeptide contains the 70-aa peptide ligand comprised of the conserved B, C, A and D domains encoded by exons 3 and 4. Splicing of exons 4, 5 and 6 produces 3 different extension peptides (a.k.a. E-domains), which are cleaved during propeptide processing (Oberbauer 2013) (Figure 1). Within the E-domain the first 16 a.a in the N-terminus are conserved between the isoforms while the C-terminal sequences are unique to each isoform (IGF-Ea, Eb, Ec). **(** Philippou et al., 2014; Rotwein 2018). This pattern of splicing is conserved and ensures sequence conservation of the IGF-1 peptide and the N-terminal portion of the E-domain amongst the vertebrates (Chen et al., 2001; Wallis 2009). However, it is the sequence divergence within the C-terminal region of the E-domain that has prompted interest in their role in IGF-1 isoform function.

In the striated muscle of rodents two IGF-1 isoforms are expressed, IGF-1Ea and IGF-1Ec. IGF-1Ec is also known as Mechano-growth factor (MGF), due its response to mechanical stretch in skeletal muscle (Yang et al., 1996; McKoy et al., 1999). Temporal regulation of IGF-1 isoform mRNA expression occurs in both skeletal and cardiac muscle in response to growth stimuli and injury. Induction of MGF mRNA peaks earlier (∼24 h) despite being in lower abundance than the IGF-1Ea mRNA, which peaks later (∼7 days) (Cheema et al., 2003; Hill et al., 2003; Mavrommatis et al., 2013). Studies examining E-domain function have generally used two different approaches, overexpression of genetically manipulated IGF-1Ea and MGF cDNAs or delivery of E-domain peptide analogs. Both approaches indicate the E-domains do exert biological activity, but the mechanism is not understood. Overexpression of IGF-1Ea and MGF cDNAs with the cleavage site between the D and E-domains rendered inactive through insertion of a stop-codon, elicits separate gene expression profiles compared to the propeptide forms suggesting differential regulation of IGF-1 mediated gene expression by virtue of the E-domains (Barton et al., 2010). Likewise, expression of a MGF cDNA harboring a mutation in the IGF-1 polypeptide, was sufficient to induce skeletal muscle hypertrophy without an increase in specific strength, suggesting autonomous E-domain function (Brisson et al., 2014). An alternative strategy for testing predominantly MGF E-domain function, has focused on the use of peptide analogs corresponding to the unique C-terminal sequence. While this approach has produced conflicting reports in skeletal muscle cell lines, studies in other cell types implicate these peptides in regulation of cellular signaling, migration and proliferation *in vitro* (Yang and Goldspink, 2002; Mills et al., 2007; Collins et al., 2010; Deng et al., 2011; Fornaro et al., 2014). In addition, a stabilized MGF E-domain peptide has also been shown to pass through the blood brain barrier to exert neuroprotective effects during transient cerebral ischemia and in response to neurotoxic agents *in vivo* (Dluzniewska et al., 2005; Quesada et al., 2009; Podratz et al., 2020).

In the heart, short-term intracoronary delivery of the MGF E-domain peptide produced cardioprotective actions and functional benefits in an ovine model of myocardial ischemia (Carpenter et al., 2008). Advancing this approach, we have previously reported that systemic delivery of the MGF E-domain peptide preserves cardiac function, prevents pathologic remodeling, myocyte apoptosis and delays decompensated heart failure. These positive outcomes were dependent on the time of delivery post-myocardial infarction (MI) in mice (Mavrommatis et al., 2013; Shioura et al., 2014). Moreover, these functional improvements were also found post-MI following cardiac restricted delivery of the MGF E-domain peptide *via* peptide eluting polymeric microstructures, providing further indication of a direct action of the peptide on the heart (Peña et al., 2015).

To gain mechanistic insight into the actions of the peptide and to better understand the function of MGF’s E-domain, we conducted *in silico* analysis of functional motifs within the C-terminal 24-aa primary sequence of the human MGF E-domain. We identified two putative phosphorylation motifs on either side of a polybasic stretch. In particular, the phosphorylation motif surrounding Ser^18^ also resides within a putative 14-3-3 binding domain. To test the biological function of this motif, we used peptides containing amino acid substitutions that mimic or prevent phosphorylation of Ser^18^. In the results presented here, we show that delivery of these peptide variants to healthy mice reciprocally regulates cardiac contractility and genes expression profiles in the heart and skeletal muscles *in vivo*. Mechanistic studies in skeletal and cardiac cell lines, show that peptide mediated modulation of the Nuclear Receptor Subfamily 4 Group A (Nr4a) family gene expression, occurs in response to membrane depolarization. We define a 14-3-3 isoform interactome that integrates proteins involved in contractile function, in which the thermal stability of 14-3-3γ and its ability to interact with target proteins in the heart is modulated by the phosphorylation state of the MGF E-domain at Ser18. Overall, our data suggests MGF’s E-domain may function as an intracellular fragment of the IGF-IEc isoform propeptide and that MGF’s E-domain phosphorylation modulates 14-3-3/client protein interactions through biologically functional motifs.

## Materials and methods

The experiments were approved by the Institutional Animal Care and Use Committee in accordance with the National Institutes of Health Guide for the Care and Use of Laboratory Animals. Additional Supplementary Figures S1–S10), Supplementary Methods (Supplementary Tables S1–S2) and microarray datasets (Supplementary Tables S3–S6) can be found in the Supplementary Materials section online.

### *In silico* analysis of the E-domain

The amino acid sequences of the human class 1 MGF prepropeptide (RefSeq: NP_001104753.1, UniProt: Q13429), rat (RefSeq: NP_001075947.1, UniProt: F8WFZ5) and mouse (RefSeq: NP_034642.2, UniProt: Q4VJB9) were aligned using the Align Sequences Protein BLAST (bl2seq) tool. The entire E-domain and unique C-terminal 24-aa region were analyzed using web-based bioinformatics tools; NetPhos 2.01 for predicting serine, threonine or tyrosine residue phosphorylation, NetPhosK 1.0 for kinase specific phosphorylation sites, t*he Eukaryotic linear motif resource* (*ELM*)*, and 14-3-3 Pred, a webserver to predict 14-3-3 binding sites in protei*ns (Blom et al., 1999; Blom et al., 2004; Madeira et al., 2015; Kumar et al., 2020) (Supplementary Figure S1).

### I*n Vitro* peptide phosphorylation

Peptide phosphorylation with human recombinant cyclic AMP-dependent protein kinase, catalytic subunit (PKAc, Sigma, C 8482) was performed in 75 μl volumes with final concentrations of peptide (90 µM) plus or minus PKAc (450 nM) in relax buffer [68 mM KOH, 6.24 mM ATP, 10 mM EGTA, 10 mM creatine phosphate, 47.6 mM potassium propanoate, 100 mM BES, 6.5 mM MgCl2, pH 7.0] for 1 h at 30°C, at 300 RPM in a Thermomixer. Equal volumes of reaction mixture containing approximately 2 μg of peptide were run on 4–20% SDS-PAGE Mini PROTEAN TGX gels (BioRad), with the PeppermintStick^TM^ Phosphoprotein standard (ThermoFisher). Gels were fixed for 1.5 h at room temperature in a 100 ml solution that contained 25 ml absolute ethanol, 15 ml 37% Formaldehyde (Fisher, F79-500) and 60 ml Milli-Q water. After brief rinses in Milli-Q water, gels were stained with Pro-Q Diamond (Invitrogen, Molecular Probes, P33301) per the manufacturer’s specifications.

### E-domain peptide treatments

Human MGF E-domain C-terminal 24-aa peptide analogs were synthesized and purified to >90% by HPLC (Lifetein Corp, NJ). Peptides were stabilized by amidating the C-terminus and Arg^14,15^ switched to the D-stereoisomer. All the peptide variants studied are listed in Supplementary Tables S1. Male B6C3F1/J (25–30 g) mice were anesthetized with 3% isoflurane maintained through a nose cone. Micro-osmotic pumps (Alzet, Model 1002, Durect Corp) were loaded with saline or peptide, and implanted subcutaneously. Mice received doses of 2.25, 4.5 and 9 mg/kg/day for 2 weeks.

### Echocardiography

A cohort of mice was sedated with Etomidate (10 mg/kg, i.p.) and sedation was maintained with 3% isoflurane inhaled through a nose cone. Echocardiography was performed with a M12L (11-MHz) linear array transducer (Vivid 7, GE Medical Systems, Milwaukee, WI). Closed-chest imaging was performed in the short-axis view at the mid-level of the left ventricle, verified by the presence of prominent papillary muscles. Image depth was 1.5 cm, 293.6 frames/s (fps) acquisition, with electrocardiographic gating performed by an experienced sonographer blinded to the study groups. An M-mode display was generated from raw data 2D images using the EchoPac software (EchoPac, General Electric, Wauwatosa WI), from a line selected passing through the anterior and inferior segments. Ejection fraction % (EF) was measured using left ventricle end diastolic and systolic volumes, with the formula EF= (LVEDV – LVESV)/ LVEDV x 100. Fractional shortening % (FS) was calculated by FS = [LV end diastolic dimension (LVEDD)—end systolic dimension (LVESD)]/LVEDD x 100. For strain analysis, images were processed with EchoPAC Q analysis software. The cardiac cycle was defined from the peak of consecutive R waves (Migrino et al., 2007). The endocardial border was traced in an end-systolic frame and the outer border adjusted to approximate the epicardial border. Six equidistant tissue-tracking regions within the myocardium provided a profile of radial (myocardial deformation toward the center) and circumferential (myocardial deformation along the curvature) strain (%) with time. Due to poor apical imaging windows in rodents, only the apical four chamber view was obtained for longitudinal strain analysis (myocardial deformation toward the base). The region of interest (ROI) was corrected manually to include the septal and lateral wall of the left ventricle. End systole was set at the zero crossing of the parametric curve on the radial strain rate curve. End-systolic radial, circumferential and longitudinal strain was obtained for each of the 6 segments and global strain calculated as the average. The early (E′) and late (A’) diastolic strain rates were measured as a marker of the diastolic function (Schattke et al., 2014). Three consecutive heart beats were measured, and the average was used for analysis.

### Pressure-volume loops

Mice were anesthetized with 3% isoflurane inhaled in a closed chamber followed by Etomidate (10 mg/kg, i.p.). Mice were intubated and connected to a rodent ventilator with a tidal volume 140–170 μl and a rate of 130–170 breaths/min (Harvard Apparatus). Surgical anesthesia was regulated by delivery of 1.5% isoflurane through a vaporizer with 95% oxygen and routinely monitored by the toe pinch reflex response. A 1.2 French pressure-volume conductance catheter (Scisense Instruments, London, Ontario) was inserted into the right carotid artery then advanced retrograde into the LV to record baseline hemodynamics with the ADVantage Pressure Volume Conductance System (Scisense Instruments, London, Ontario). A small incision in the abdomen was made and hemodynamics recorded following transient occlusion of the abdominal vena cava to vary venous return. Data were collected with the Iworx IX/228S Data Acquisition System and analyzed with Labscribe 2.0 software package from Iworx (Dover, NU). In addition, total peripheral resistance (TPR) was derived by dividing mean arterial pressure by cardiac output. Cardiac index (CI), the ratio of cardiac output divided by body weight, was calculated as previously described (Mavrommatis et al., 2013; Shioura et al., 2014; Peña et al., 2015). Surface ECG profiles were obtained with standard 3 lead subdermal needle electrodes (C-ISO-GNE5, Scisense) inserted in the right and left upper limbs and right lower limb of the animals. Data acquisition and analysis were performed using IWORX Labscribe2 software. Following the procedure, mice were euthanized with 5% isoflurane, their hearts removed and weighed before freezing in liquid nitrogen.

### Circulating catecholamines

Circulating catecholamines were measured in blood collected *via* ventricular puncture (1 ml) by Reverse-phase HPLC. Briefly, 0.5 ml of plasma was extracted with acid-washed alumina and 3, 4-Dihydroxybenzylamine (DHBA) used as an internal standard. The HPLC system included a Hitachi L-7000 isocratic pump, BASi LC-4B electrochemical detector, Rheodyne 7125 manual injector with a 100 μl loop and a Hitachi D-2500 Chromato-Integrator. The reverse phase column was a Supelco LC-18, 25 cm × 2.1 mm, with 5μm particle size. The mobile phase consisted of 25 mM citric acid, 25 mM dibasic sodium phosphate, 1 mM disodium EDTA and 45 mg/L sodium octyl sulfate. The flow rate was 0.7 ml/min and run time was 40 min / sample at room temperature. The detector was set with an applied potential of 650 mV with a sensitivity of 1nA.

### Gene expression

Total RNA was extracted from the apex of the heart with TRIzol (Invitrogen) and run on an Experion gel (Bio-Rad) to evaluate integrity. Samples (3 hearts /group) were labeled and hybridized according to the standard whole transcript ST protocol recommended by Affymetrix and hybridized using an Affymetrix GeneChip Mouse Gene 2.0 ST Array. Each image was analyzed for the following quality metrics: total background, raw noise (Q), average signal present, signal intensity of species-specific house-keeping genes, 3’/5’ signal ratio of house-keeping genes, relative signal intensities of labeling controls, and absolute signal intensities of hybridization controls. Microarray data was processed using Partek 6.12.0530. Briefly, Robust Multi-array Average (RMA) was used for background correction and quantile normalization, followed by one-way ANOVA to identify differentially expressed genes.

### Cell lines and membrane depolarization

HL-1 atrial myocytes were grown in Claycomb media (Sigma) supplemented with 10% fetal bovine serum (FBS), 100 μM norepinephrine, 4 mM L-glutamine and antibiotics (100 units/ml penicillin G and 10 μg/ml streptomycin). Prior to treatments, cells were switched to serum free Claycomb media, without norepinephrine plus 0.25% BSA overnight (Claycomb et al., 1998). C2C12 skeletal myoblasts were grown in DMEM supplemented with 10% FBS and antibiotics (100 units/ml penicillin G and 10 μg/ml streptomycin) and differentiated to form myotubes in DMEM with 2% heat inactivated horse serum for 5 days. Cells were switched to serum free DMEM plus 0.25% BSA overnight prior to treatments. Cells were washed with Ca^2+^ and Mg^2+^-free PBS and equilibrated in Krebs-Ringer buffer for 30 min [118 mM NaCl, 4.7 mM KCl, 3 mM CaCl_2_, 1.2 mM MgCl_2_, 10 mM glucose and 20 mM HEPES-Tris (pH 7.4)]. To induced depolarization with high [K+]_o_, NaCl was isosmotically replaced with 84 mM KCl (Cárdenas et al., 2004). High KCl buffer without [Ca^2+^]_o_ contained 0.5 mM EGTA and 4.2 mM MgCl_2_. Peptides were dissolved in water and added immediately following membrane depolarization to achieve a final concentration as indicated.

### Quantitative RT-PCR

Total RNA was extracted with TRIZOL and used in a one-step RT-PCR reaction with the SYBR Green RNA Amplification kit (Roche Molecular Biochemical, IN) in a LightCycler thermocycler (Roche Diagnostics), as previously described (Mavrommatis et al., 2013; Shioura et al., 2014; Peña et al., 2015). Tissue RNA was normalized against peptidylprolyl isomerase E (cyclophilin E) expression and cellular RNA normalized against 18s. Primers were obtained from PrimerBank (http://pga.mgh.harvard.edu/primerbank) or purchased as pre-made primers (Sigma). Primer sequences were run against the BLAST database and used for melting curve analysis prior to ensure single gene specific product amplification during four-step PCR amplification. All the primer sequences used are listed in Supplementary Table S2.

### Western blotting

Proteins were extracted in Tissue Protein Extraction Reagent (T-PER) with the addition of Halt Protease and Phosphatase Inhibitor Cocktail (Thermo Scientific) using a PowerGen 700 tissue homogenizer (Fisher Scientific). Proteins (25 μg) were separated on 10% Criterion gels (Bio-Rad), transferred to nitrocellulose and probed using standard immuno-blotting techniques. Blots were incubated overnight at 4°C with primary antibodies (IGF1R^(Y1135/1136)^/InsR^(Y1150/1151)^, IGF1R^(Y1316)^, IGF1R^(Y980)^, IRS-1^(S307)^, PI3Kp85^(Y458)^/p55^(Y199)^, PDK1^(S241)^, Akt^(T308)^, Akt^(S473)^, mTOR^(S2448)^, S6 ribosomal protein^(S235/236)^ and p44/42 (Erk1/2)^(T202/Y204)^ (Cell Signaling) and NR4A2 (Nurr1 M-196, Santa Cruz Biotechnology). HRP-conjugated secondary antibodies (Cell Signaling) were used and immunoreactive bands were detected with the ECL Western Blotting Chemiluminescent Substrate (Pierce). Bands were visualized using Bio-Rad XRS^+^ ChemiDoc station and expressed relative to corresponding non-phosphorylated protein and GAPDH expression (Cell Signaling) as previously described (Mavrommatis et al., 2013).

### Myofilament extracts

Excised hearts were immediately frozen in liquid nitrogen. 10–20 mg of tissue was homogenized 1:10 relative to original tissue weight in standard relax buffer [SRB; 75 mM KCl, 10 mM imidazole pH 7.2, 2 mM MgCl_2_, 2 mM EGTA, 1 mM NaN_3_] with protease and phosphatase inhibitors [1:100 v/v Sigma, P-8340, 1:100 v/v Millipore, 524624, and 100 nM of Calyculin A in DMSO, Cell Signaling, 9902]. The tissue was homogenized at 4°C with a Bead Ruptor 24 Elite (Omni International, 19-040E) at power, 5 m/s; time, 15s; for 3 cycles; with a 3 min dwell time between cycles as previously described (Capote et al., 2021). The myofibril preparations were washed once with Triton X-100 added to 0.5% (v/v) in SRB buffer. The myofibril preparation was spun clarified at 4°C, 15,000 X g for 1 min, and 500µL of SRB with 1% (v/v) Triton X-100 was added to the pellet and incubated on ice with intermittent vortexing for 15 min and repeated. The pellet was then washed with SRB without Triton X-100 and resuspended 1:5 relative to the original tissue weight in 2X Laemmli buffer (BioRad, #161–0747).

### 14-3-3 Protein expression and purification

All seven mouse 14-3-3 isoforms’ cDNAs (YWHAB, YWHAE, YWHAG, YWHAH, YWHAQ, SFN and YWHAZ) cloned into pPROEX-HTb vector with N-terminal six His-tags were kindly provided by Dr. Sheila Baker (Department of Biochemistry, University of Iowa). Single colony transformed *E. coli* BL21 (DE3) Rosetta cells (Millipore, 70954-4) were grown overnight (37°C, 225 RPM, 4 ml volumes) in LB (Luria Broth, RPI Research Products International, L24045-500.0) supplemented with 100 µg per ml ampicillin. Overnight cultures were brought to mid-log phase growth in LB/ampicillin (37°C, 225 RPM, 500 ml volumes). Protein expression was induced by adding isopropyl β-D-1-thiogalactopyranoside (1 mM, final concentration) and cells were incubated for 3 h at 37°C. Cells from 500 ml cultures were collected by centrifugation and sonicated to homogeneity on ice in detergent-free buffer [25 ml; 50 mM NaH_2_PO_4_, 200 mM NaCl, 10% (v/v) glycerol, 25 mM imidazole, pH 8.0]. Lysates were centrifuged (40,000 x g, 4°C, 20 min) and 14-3-3 proteins were batch-purified by adding 0.8 ml slurry HisPur Nickel-NTA-agarose (Thermo Fisher Scientific, 88221) to 25 ml supernatant. Supernatants were rotated (4 °C, 30 min,) centrifuged (500x g, 4°C, 1 min) and removed. Similarly, beads were washed three times with detergent-free buffer (10 ml each, 10 min each). Purified 14-3-3 proteins were eluted from centrifuged, supernatant-free beads by performing four sequential 750 µl elutions with detergent-free elution buffer [50 mM NaH_2_PO_4_, 200 mM NaCl, 10% (v/v) glycerol, 250 mM imidazole pH 8.0]. Typical yields were 6-7 mg purified 14-3-3 protein per 500 ml culture with >95% purity as assessed by SDS-PAGE gel and Coomassie staining (Supplementary Figure S2).

### 14-3-3 Affinity capture

Affinity capture was used to identify novel 14-3-3γ interacting proteins in whole heart lysates. To validate our approach, we tested whether 14-3-3/client protein interactions were specific using negative controls (no 14-3-3 present during capture), blocking with a known 14-3-3 inhibitor, R18, (R18 trifluoroacetate, Sigma Aldrich, SML0108) and varying the amounts of input lysates to optimize capture of client proteins (Supplementary Figure S3). In experiments that tested the effect of peptides on affinity capture, peptides were added (20 µM) during each incubation step (e.g., 14-3-3 binding, blocking and overnight heart lysate incubation) except for the wash buffers steps.

Mice were injected with saline or dobutamine (1.5 mg/kg i.p., 10 mins) to stimulate the β_1_-adrenergic receptor (β_1_-AR). Mice were euthanized with 5% isoflurane, their hearts removed and frozen in liquid nitrogen. To prepare lysates, whole hearts (∼110 mg, wet weight) were used to extract soluble proteins using a Duall homogenizer and 2 mls of ice-cold lysis buffer [50 mM Tris-HCl, 150 mM NaCl, 1.0% NP-40 with protease (MilliporeSigma, P8340), phosphatase (MilliporeSigma, 524624) inhibitors and Calyculin A (Cell Signaling Technology, 9902S, 100 nM, final concentration)]. Lysates were pre-cleared by the addition of Pierce Protein A/G Plus agarose beads (Thermo Fisher Scientific, 20423) and rotated for 2 h at 4°C. Insoluble cellular debris was removed by 20,000 x g centrifugation for 10 min at 4°C. Protein concentrations were determined using the Pierce 660 nm Protein Assay Reagent (Thermo Fisher Scientific, 22660) according to the manufacturer’s specifications. While total heart lysates were being prepared, purified 14-3-3 proteins were bound to Ni-NTA-agarose beads in “batch mode” [100 µg in 1 ml of 50 mM NaH_2_PO_4_, 200 mM NaCl, 10% (v/v) glycerol, pH 8.0]. Beads were briefly centrifuged, and supernatants removed. Beads were then incubated in 1 ml blocking buffer [50 mM Tris-HCl, 150 mM NaCl, 0.1% Tween-20, 2% bovine serum albumin, pH 7.5] briefly centrifuged, and supernatants removed. After addition of heart lysate (typically 375 µg unless otherwise indicated) to incubation buffer [1 ml total volume of 50 mM Tris-HCl, 150 mM NaCl, 0.1% NP-40 with protease, phosphatase and Calyculin A], samples were rotated overnight at 4°C. Beads were washed sequentially by rotation at 4°C in 1 ml per sample of the following solutions: 1) 50 mM Tris-HCl, 150 mM NaCl, 1.0% NP-40, pH 7.5, 5 min; 2) 50 mM Tris-HCl, 150 mM NaCl, 50 mM imidazole, pH 7.5, 10 min; 3) 50 mM Tris-HCl, 150 mM NaCl, 100 mM imidazole, pH 7.5, 5 min; 4) Rapid wash in 50 mM Tris-HCl, 150 mM NaCl, 150 mM imidazole, pH 7.5; and finally, 5) Elution with 300 µl per sample of 50 mM Tris-HCl, 150 mM NaCl, 250 mM imidazole, pH 7.8. Eluted proteins were acetone precipitated overnight at -20 °C, then collected by centrifugation (20,000 x g, 4°C, 10 min), solubilized in sample buffer (0.05 M Tris-HCL, 8 M urea, 2M thiourea, 75 mM dithiothreitol, 3% SDS, 0.005% bromophenol blue, pH 6.8) and heated for 3 min at 95°C (Fritz et al., 1989).

Proteins from 14-3-3 affinity capture samples, along with indicated amounts of heart lysate (typically 20 µg), were separated on 12% Mini Protean TGX gels (Bio-Rad), transferred to nitrocellulose and probed using standard immuno-blotting techniques. Blots were incubated overnight at 4°C with primary antibodies (MYBPC3 (Santa Cruz), PLN (Badrilla), ARRB2 (Boster), 14-3-3γ, PKD, HSP20 (Cell Signaling). HRP-conjugated secondary antibodies (Cell Signaling) were used and immunoreactive bands were detected with SuperSignal West Femto Maximum Sensitivity Substrate (ThermoFisher, 34096). Bands were visualized using Bio-Rad ChemiDoc MP station and analyzed with ImageLab (Bio-Rad, v. 6.0.1). To quantify changes in relative protein abundance, samples were pooled, proteins transferred and run-in triplicate by slot blotting (Minifold II Slot-Blot, Schleicher & Schuell) to Whatman Protran BA85 nitrocellulose per the manufacturer’s specifications. Signals for mouse MYPC3 and PLN were normalized to the 14-3-3 signal detected using a rabbit pan 14-3-3 antibody (8312, Cell Signaling).

### Thermal shift assay

Thermal shift assays were performed using a BioRad CFX Connect Real-Time CFX96 PCR System running CFX Maestro Software and configured to perform thermal shift assays as per the manufacturer’s specifications (BioRad Technical Bulletin 7180). All melt curve data were collected using the SYBR acquisition mode channel with 0.5°C. per 10 s temperature increases over a 10–95°C range. Reactions were performed in 200 μl, 8-tube per strip, flat-capped tubes (Amplifit, Thomas Scientific, 1149K07) in final reaction volumes of 20 μls. Each 20 µl reaction volume contained 1 µl of 1:40 Milli-Q-diluted dye (GloMelt Thermal Shift Dye, Kit 33021-1, Biotium), recombinant 14-3-3γ (typically 8 μM, final concentration), or added peptides (from 1 mg/ml Milli-Q stock solutions) in 10 mM sodium phosphate buffer, pH 7.5. Discrete midpoint temperatures of protein-unfolding transition (Tm) were calculated by subtracting blank reaction constituent and “dye alone” RFU data from the sample RFU data. RFU minima to RFU maxima data were exported into GraphPad Prism 9 software, normalized for highest signal arbitrarily set to one and lowest signal set to 0. Normalized values were then analyzed *via* the Boltzmann Sigmoidal equation to generate specific Tm values.

### Neonatal ventricular rat cardiac myocyte isolation and culture

NRVMs were isolated as previously described (Solís and Russell, 2019). Briefly, hearts excised from 0-2 days-old Sprague–Dawley rats were minced and digested with collagenase type II (Worthington, # LS004177). After incubation at 37°C for 10 min with agitation, NRVMs in suspension (10 ml) were collected and added to fetal bovine serum (10 ml). This process was repeated until the tissue was completely digested (usually between five to six repetitions). NRVMs were filtered through a 70-µm nylon sieve, centrifuged, and resuspended in media containing DMEM and Medium 199 at a 1:5 ratio, 10% (v/v) horse serum, and 5% (v/v) fetal bovine serum. Myocytes were plated on glass-bottom dishes and replaced with serum-free (DMEM and Medium 199 only) media the next day.

### Immunofluorescence staining

NRVMs were washed with PBS and treated with the extraction buffer I derived from the ProteoExtract Subcellular Proteome Extraction kit (EMD Millipore, 539790) with the addition of protease (Calbiochem, 539131) and phosphatase (EMD Millipore, 524625) inhibitors. Extraction was conducted for 10 min at 4°C. NRVMs were gently washed with PBS and fixed with a formaldehyde solution (10%) for 10 min at room temperature. Mouse anti-α-actinin (Abcam, 9465, 1:1000) and rabbit anti-14-3-3γ (Cell Signaling, 5522, 1:100) antibodies were incubated overnight at 4°C. In addition, rabbit anti-14-3-3α/β, ε, η, τ, ζ/δ isoform specific antibodies were also tested (Cell Signaling, 9636, 9635, 5521, 9638, 7413, respectively). After washing with PBS, the cells were incubated with secondary anti-rabbit AF488 (ThermoFisher, 21206, 1:1000) and anti-mouse AF555 (ThermoFisher, A21425, 1:1000) for 1 h at RT. Images were acquired on a Zeiss Axio Observer inverted microscope using a 63x Plan-Apochromat (N.A. 1.4x, oil DIC). Images were analyzed using the Fiji image processing software and signals enhanced in unbiased way using the auto function for all 3 channels (Solís and Russell, 2019). In addition, raw image files and images of cells incubated with the secondary antibodies only as negative controls are provided (Supplementary Figures S4, S5).

### Histology

Hearts were excised and placed into cold PBS where they were cleaned of extraneous tissue. The hearts were quickly sliced in basal, midpapillary and apical parts, and placed into biopsy cassettes, followed by fixation in 10% neutral buffered formalin (Millipore-Sigma, HT501128), then washed and stored in 70% Ethanol. Samples were paraffin embedded and non-consecutive transverse 10 μm sections were cut. Slides were deparaffinized with 100% xylene (2 × 7 min) followed by rehydration with incremental washes of decreasing aqueous ethanol (100% for 2 × 5 min, 95% for 5 min, 70% for 5 min, and 50% for 5 min) solutions, washed in H_2_O for 20 min and used for staining. Antigen retrieval was performed using Tris-EDTA solution at 95°C for 1.5 h. Slides were then blocked in 5% BSA in TBST (0.1% Tween-20) for 1 h at room temperature. Slides were incubated in goat anti-14-3-3γ (Biorbyt, orb44462, 1:200) and rat anti-CD31 antibody (Dianova, DAI, 310, 1:10) in TBST (Tween 0.1%) overnight at 4°C. Following three 5min washes with TBST (Tween 0.1%), slides were incubated with antibodies secondary anti-rabbit AF568 (Invitrogen, A11011, 1:1500) and anti-rat AF633 (Invitrogen, A21094, 1:1500) for 1 h at RT. Slides were also incubated with secondary antibodies as negative controls (Supplementary Figure S5) Slides were washed three times 5 min and incubated with DAPI for nuclear counterstaining for 20 min at RT. Slides were then washed in TBST and mounted with a mounting medium (ThermoFisher Scientific, P10144) and imaged on a Zeiss LSM880 confocal microscope.

### Statistics

Data are expressed as means ± standard error. Cardiac function data were analyzed using two-way ANOVA to test for the influence of two independent variables: peptide and dose. All other assays were analyzed using one-way ANOVA with the Holm-Sidak method used to test for statistical significance set at *p < 0.05.*

## Results

### The MGF E-domain C-terminus contains putative phosphorylation and 14-3-3 motifs

Comparison of the MGF prepropeptide amino acid sequence shows 87% and 88% homology between human vs. rat and human vs. mouse, respectively (Supplementary Figure S1). Within the signal peptide (1–48), the BCAD domains of the IGF-1 polypeptide (49–118) and N-terminus of the E-domain (119–134), there is 93% homology between human vs. rat and 92% between human vs. mouse. However, within the C-terminus of the E-domain (135–159) only 67% homology exists between human vs. rat and 70% between human vs. mouse. Nevertheless, within this region a polybasic motif and an adjacent sequence (^148^RRKGSTF^154^) are conserved throughout the vertebrates with the latter showing similarities to the serine/threonine kinase consensus phosphorylation motif (RKGS/TF). Analysis of the MGF prepropeptide predicted Ser^152^ within this motif to have the highest potential score for phosphorylation in human and rodent sequences (human = 0.994, rat = 0.996, mouse = 0.996). Analysis of the E-domain 24-aa C-terminal sequence indicated that Thr^6^, Ser^12^ and Ser^18^ (numbering based on the position within the 24-aa sequence) could be potentially phosphorylated, with Ser^18^ (corresponding to Ser 152 in the prepropeptide) having the highest score. Further analysis with NetPhosK and Eukaryotic Linear Motif (ELM), identified cAMP-dependent protein kinase (PKA) as a potential kinase that could phosphorylate Ser^18^ residing within a 14-3-3 binding domain (Supplementary Figure S1).

To identify putative 14-3-3 binding sites in interactor proteins, the 14-3-3 Pred tool revealed a single putative 14-3-3 binding site at SQRRKG [S]TFFEE, (where [S] corresponds to Ser^152^ in the human prepropeptide or Ser^18^ in the E-domain 24-aa C-terminal sequence). In the human this 14-3-3 site was relatively weak (0.475 consensus = average of the scores provided by the three methods (cut-off = 0.50)) but was predicted to be stronger in the rat (0.621) and the strongest in the mouse prepropeptide (0.764), (Figure 1).

**FIGURE 2:**
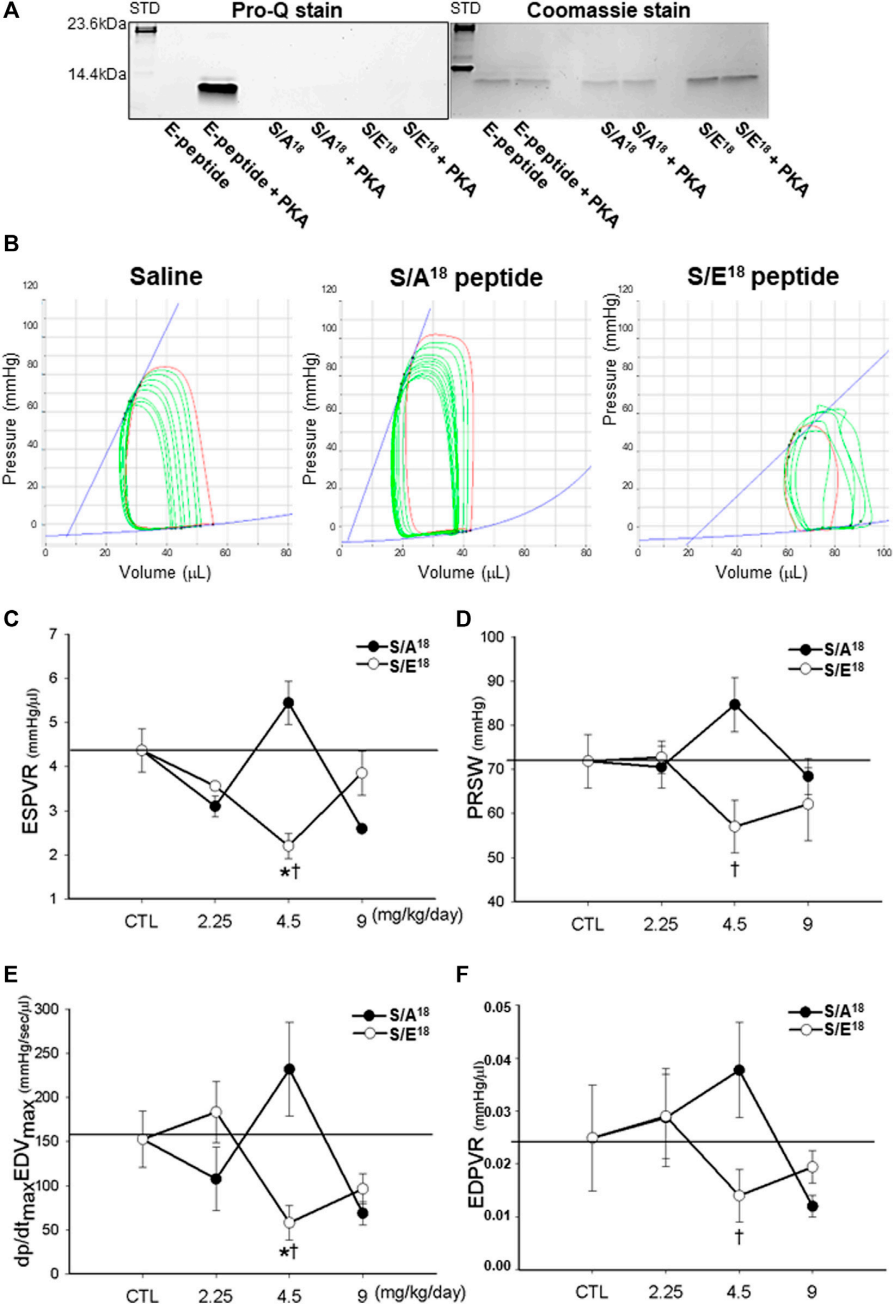
E-domain phosphorylation and measurements of contractility in the hearts of phospho-null and phosphomimetic peptides treated mice. **(A)**. PKA phosphorylation of the native E-domain peptide, phospho-null and phosphomimetic peptides *in vitro.* **(B)**. Representative P-V loops following inferior vena cava occlusion in the hearts of peptide treated mice at 4.5 mg/kg/day dose. **(C)**. ESPVR-end systolic pressure volume relationship. **(D)**. PRSW-preload recruitable stroke work. **(E)**. Maximum dP/dt vs maximum EDV. **(F)**. EDPVR-end diastolic pressure volume relationship. (*p < 0.05 vs. CTL and ^*†*^ *p* < 0.05 vs. peptide at same dose, *n* = 10).

To examine the potential for E-domain peptide phosphorylation, we phosphorylated the native human E-domain peptide with the catalytic subunit of PKA *in vitro*. We also tested whether other potential sites (Thr^6^ and Ser^12^) in addition to Ser^18^, could be phosphorylated using the phospho-null (S/A^18^) and phospho-mimetic (S/E^18^) E-domain peptides. While PKA clearly phosphorylated the native E-domain peptide, the amino acid substitutions at Ser^18^ prevented further phosphorylation at other sites (Figure 2A). While other kinases may preferentially target other sites, the data suggests that Ser^18^ identified with the *in silico* approach, may be a physiologically relevant PKA site (Figure 2A).

### E-domain Ser18 phosphorylation modulates cardiovascular function and contractility

To examine the actions associated with altering Ser^18^ phosphorylation, we delivered phospho-mimetic (S/E^18^) and phospho-null (S/A^18^) E-domain peptides to healthy mice. The cardiovascular hemodynamics measured *in situ* showed a decline in LV function at the lowest and highest doses of both peptides compared to controls (Table 1). This was evident in the decline in heart rate, systolic (ESP, dP/dt_max_) and diastolic function (Tau, dP/dt_min_). In contrast, the peptides produced opposing effects at the medium dose (4.5 mg/kg/day). The S/A^18^ peptide treatment restored all hemodynamic measurements to controls levels compared to the S/E^18^ peptide treatment which significantly exacerbated their decline. Total peripheral resistance (TPR) showed no statistical difference with the S/A^18^ peptide but increased at the medium dose S/E^18^ peptide which may be a compensatory response to a decline in mean arterial pressure (MAP). A right shift in the PV-loops with a decline in the pressure signal and greater LV operating volume was evident in S/E^18^ treated mice, with an opposing effect noted in the S/A^18^ treated mice at the same dose (Figure 2B).

**TABLE 1:** Cardiac function in mice with either S/A^18^ or S/E^18^ modified MGF E-domain peptide treatment at different doses compared to saline. Pressure-volume loop measurements collected in the closed chest configuration.

Group parameter	Control	S1A^18^ peptide	SIA^18^ peptide	SIA^18^ peptide	SlE^18^ peptide	S/E^18^ peptide	SIE^18^ peptide
Dose (mg/kg/day)	Saline	2.25	4.5	9	2.25	4.5	9
HR	571–120	477 ± 15^*^	553 ± 18w	478 ± 6^*^	497 ± 6^*^	477 ± 7^*^t	514–121
MAP	67.8 ± 1.6	59.8 ± 3.4	66 2 ± 2	60.2 ± 1.8	56.3 ± 0.56	52.8 ± 2.	57 ± 1.8
ESP	91.5 ± 1.7	79.8 ± 3.7^*^	92.2 ± 2.8^4^	86.8 ± 3.3	75.8 ± 2.1^*^	71.6 ± 4.3^*^t	79 ± 2.8^*^
EDP	4.1 ± 0.7	4.7 ± 0.7	2.9 ± 0.6	2.3 ± 0.2	4.0 ± 0.8	3.3 ± 0.8	3.1 ± 0.3
EDV	49.8 ± 2.8	58.2 ± 3.5	46.5 ± 2.2″	59.7 ± 1.0	59.3 ± 2.7	53.8 ± 3.7	56.7 ± 3
ESV	26.4 ± 2.6	36.6 ± 2.7^*^	22.6 ± 2″	36.6 ± 1.7^*^	36.6 ± 2.9^*^	37.2 ± 0.3^*^t	32.3–13.1
SV	23.4 ± 1.2	21.6 ± 0.8	23.9 ± 0.9	23 ± 0.8	22.6 ± 0.6	16.5 ± 1.8^*o^t	24.5 ± 0.5
CO	13271 ± 504	10294 ± 372^*^	13262-688″	10994 ± 284	11279 ± 421	7887 ± 879^*^"t	12582 ± 436
SW	2587 ± 217	2256 ± 232	2504 ± 161	2095 ± 280	1927 ± 65	1427 ± 122^*^t	2159 ± 87
EF	47.6 ± 2.6	37.3 ± 1.2^*^	52.0 ± 2.3″	38.60 ± 1.8	38.6 ± 2.4^*^	30.6 ± 2.5^*^t^#^	43.6 ± 2.6
dP/dt	9256 ± 787	5795 ± 951^*^	9242 ± 967″	5546 ± 267^*^	5611 ± 301^*^	4123 ± 497^*^t	5944 ± 606^*^
dPldt_r_	-8344 ± 500	-5847 ± 491^*^	-8286451″	-594147^*^	-6013 ± 118^*^	-4887 ± 573^*^t	-6040 ± 354^*^
Tau-G	7.28 ± 0.4	10.04 ± 0.4^*^	7.6 ± 1.0″	11.23 ± 0.6^*^	9.17 ± 0.1	10.32 ± 0.6^*^t	9.12 ± 0.7
maxPwr	716172 ± 82859	352976 ± 95077^*^	728855 ± 119539^#^	323343 ± 19057^*^	309809 ± 22109*	212332 ± 42995*t	346815 ± 58055^*^
plPwr	309 ± 49.8	114.5 ± 39	401.2 ± 97w	92.0 ± 8.2	92.34 ± 13.6	100_._3 ± 35_._5*t	112.2 ± 23.4
CI	217.6 ± 18.7	175.5 ± 8.2	215 ± 10.7	154.0 ± 10.8	171.3 ± 11.6	149.5 ± 5.8	177.6 ± 9.5
TPR	4.6 ± 0.1	5.2 ± 0.3	4.6 ± 0.2	5.1 ± 0.1	4.5 ± 0.2	690.9^*^t	4.1 ± 0.2

HR, heart rate (beats per minute); MAP, mean arterial pressure (mmHg); ESP-end-systolic pressure (mmHg), EDP, end-diastolic pressure (mmHg), EDV, end diastolic volume (µl), ESV, end systolic volume (µl); SV, stroke volume (µl), CO, cardiac output (μl/min); SW, stroke work (mmHg/µl); EF, ejection fraction (%), dP/dt_max_, maximum first derivative of change in systolic pressure with respect to time (mmHg/sec); dP/dt_min_, maximum first derivative of change in diastolic pressure with respect to time (mmHg/sec); Tau-Glantz-time constant of fall in ventricular pressure by Glantz method (msec); maxPower, maximum power (mWatts); plPwr, preload adjusted maximal power (mWatts/ml^2^); CI, cardiac index (L/min^−1^/kg); TPR, total peripheral resistance (mmHg/ml^−1^/min).

*p < 0.05 vs. control, ^*¥*^ *p < 0.05* vs. low dose, ^*#*^ *p < 0.05* vs. high dose, ^*†*^ *p < 0.05* vs. S/A^18^ 4.5 mg/kg/day dose (*n* = 10).

Measurements of cardiac contractility [End-systolic pressure volume relationship (ESPVR), Preload recruitable stroke work (PRSW) and the maximum first derivative of change in systolic pressure rise with respect to time versus the maximum end-diastolic volume (dP/dt_max_ vs EDV_max_)], were similar at the low and high dose peptide treatments. The opposing effect was also evident at a medium dose where the S/A^18^ peptide tended to enhance contractility while the S/E^18^ peptide resulted in significantly lower contractility compared to control (Figures 2C–F). The end-diastolic pressure volume relationship (EDPVR), an index of ventricular stiffness, was increased with S/A^18^ peptide but decreased with S/E^18^ peptide treatments. The decrease in stiffness (increased compliance) noted may explain the preservation of end diastolic pressure (EDP) with the S/E^18^ peptide.

Given the opposing response noted at the 4.5 mg/kg/day dose, we examined whether changes in cardiac geometry and electrophysiology could account for these differences. No difference in cardiac mass expressed as heart weight to body weight (HW/BW) or tibia length (HW/TL) with treatments was detected (Table 2). However, echocardiography revealed a small increase in posterior wall thickness in S/A^18^ treated mice compared to S/E^18^ treated mice. Expressed as relative wall thickness to ventricular diameter (2 x posterior wall/LV diameter at diastole), a mild concentric hypertrophy was induced by the S/A^18^ peptide (Saline = 0.38, S/A^18^ = 0.41 and S/E^18^ = 0.35). Fractional shortening analysis also identified a similar trend in opposing contractile function with peptide treatment as seen with the P-V loop data. Analysis of the major components of myocardial strain showed that radial (transmural) and longitudinal strains were affected. Radial strain showed opposing effects with peptide treatment which appears to correlate with the positive and negative inotropic effects noted in these mice. ECG data showed no apparent signs of cardiac arrhythmia, although a shortened QT interval was evident. Such a shortened QT interval may underlie the significantly lower longitudinal strain measurements identified in the S/E^18^ peptide treated mice.

**TABLE 2:** Cardiac geometry and strain rate analysis determined by echocardiography in mice at 2 weeks with and without MGF E-peptide treatment.

Group parameter	Control	S/A^18^ peptide	S/E^18^ peptide
Dose (mq/kg/clay)	Saline	4.5	4.5
BW(g)	29.5 = 0.6	27.5 = 0.9	29.2 = 0.6
aHW(mq)	120.9 = 2.8	116.9 = 2.9	120.1 = 2.8
HW/BW	4.0 = 0.06	4.27 = 0.1	4.0 = 0.07
Tibia (mm)	17.9 = 0.09	17.9 = 0.12	18.0 = 0.10
HW/TL	6.5 = 0.12	6.5 = 0.16	6.6 = 0.16
%Lung H_2_O	76.0 = 0.004	77.7 = 0.003	77.0 = 0.003
M-mode Echo			
IVSd	0.073 = 0.004	0.079 = 0.004	0.077 = 0.004
LVPWd	0.077 = 0.003	0.083 ± 0.004t	0.07 = 0.003
LVIDd	0.41 = 0.009	0.4 = 0.009	0.39 = 0.005
LVIDs	0.29 = 0.012	0.27 = 0.012	0.28 = 0.007
EF	61.7 = 2.3	64.6 = 2.6	61.0 = 2.4
FS	28.7 = 1.5	30.7 = 1.9	25.6 = 0.9
Strain analysis			
RS	38.9 = 4.8	41.8 = 3.5	31.3 = 2.8
CS	−12.4 = 0.9	−12.2 = 0.5	−11.7 = 0.3
LS	−12.2 = 1.2	−11.7 = 1.1	−7.4 ± 1.8k
ECG analysis			
RR	138.7 ± 11.0	144.4 = 5.1	149.6 = 7.0
PR	34.4 = 0.6	34.8 = 2.0	34.6 = 1.7
QRS	24.0 = 1.0	21.3 = 0.6	22.7 = 0.5
QT	51.1 = 1.0	49.0 = 0.6	46.2 ± 1.2*

BW, body weight (g); HW, heart weight (mg); HW/BW ratio, heart weight (mg)/body weight (g); Tibia, tibia length (mm); HW/TL ratio, heart weight (mg)/ Tibia length (mm), %Lung H_2_O, Wet weight/Dry weight (%); IVSd, Intraventricular septum thickness during diastole *(cm*); LVPWd, left ventricular posterior wall thickness during diastole *(cm*); LVIDd, left ventricular internal dimension during diastole *(cm*); LVIDs, left ventricular internal dimension during systole *(cm*); EF, ejection fraction (%); FS, fractional shortening (%); RS, radial strain global (%); CS, circumferential strain global (%); LS, longitudinal strain global (%); RR-R, wave to R wave interval (ms); PR-P, wave to R wave interval (ms); QRS, QRS complex interval (ms); QT-Q, wave to T wave interval (ms).

*p < 0.05 vs. control, ^*†*^ *p < 0.05* vs. S/A^18^ 4.5 mg/kg/day dose (*n* = 10).

### E-domain Ser18 phosphorylation alters phosphorylation of IGF-1/IGF-1R signaling intermediaries

**FIGURE 3:**
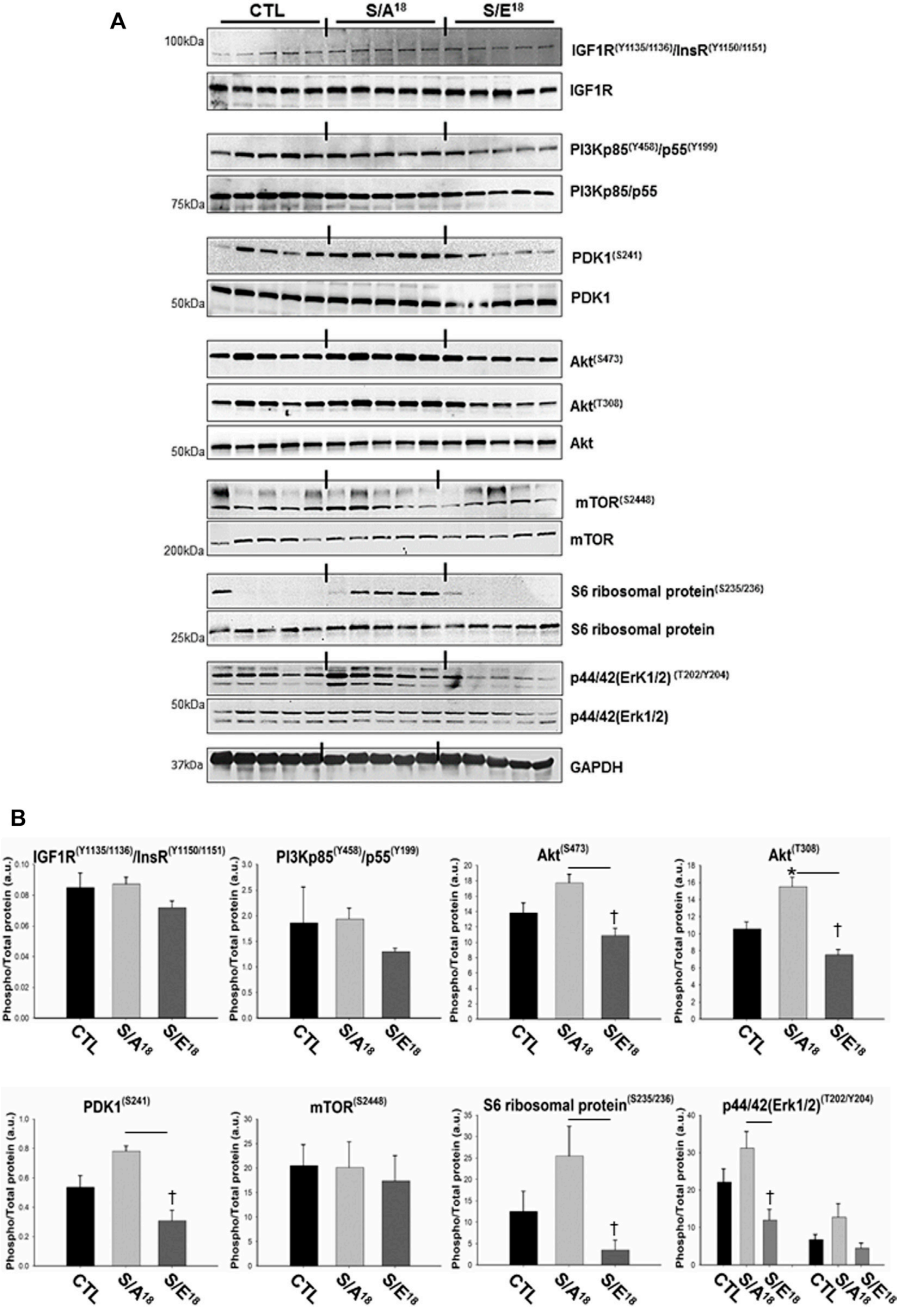
Analysis of the IGF-1R signaling pathway in the hearts of peptide treated mice at 4.5 mg/kg/day dose. **(A)**. Immuno-blots showing phosphorylation of IGF-1R pathway signaling proteins. **(B)**. Quantification of phospho-protein signaling relative to non-phosphorylated proteins (*p < 0.05, vs. CTL and ^*†*^ *p* < 0.05 vs. peptide at same dose, *n* = 5).

The data surrounding the involvement of the IGF-1R in mediating the actions of the E-domain peptide have been equivocal. Studies in skeletal myoblasts and neuronal cell lines indicate antibody blockage of the IGF-1R does not prevent the actions of the stabilized native E-domain peptide (Yang and Goldspink, 2002; Mills et al., 2007; Quesada et al., 2011). Conversely, it has been suggested that the MGF E-domain peptide does not activate the IGF-1R directly but increases activation by IGF-1 (Brisson and Barton, 2012). Consequently, we examined aspects of the IGF-1/IGF-1R canonical signaling pathway with phospho-specific antibodies against well-established signaling intermediaries in the hearts treated at 4.5 mg/kg/day. The phosphorylation of IGF-1R tyrosine kinase domain and IRS-1 associated with IGF-1 binding assessed with different antibodies (IGF-1R^(Y1135/1136)^/InsR^(Y1150/1151)^, IGF-1R^(Y1316)^, IGF-1R^(Y980)^, IRS-1^(S307)^), did not differ between control and peptide treated mice. However, the phosphorylation of several downstream proteins including PDK1, Akt, S6 ribosomal protein and p44/42 tended to increase in S/A^18^ peptide treated hearts with the phosphorylation of Atk^T308^ significantly increased versus control (Figures 3A,B). In contrast, phosphorylation of these proteins was in most cases depressed in S/E^18^ treated hearts compared to control and significantly lower for PDK1, Akt, S6 ribosomal protein and p44/42 compared to S/A^18^ peptide treated hearts. Despite no detected change in IGF-1R activation and protein expression, these signaling events culminated in S6 ribosomal protein phosphorylation in the majority of S/A^18^ peptide treated hearts, suggesting an activation of protein synthesis which account for the slight increase in LV posterior wall thickness noted in these mice.

### E-domain Ser18 phosphorylation regulates gene expression

**FIGURE 4:**
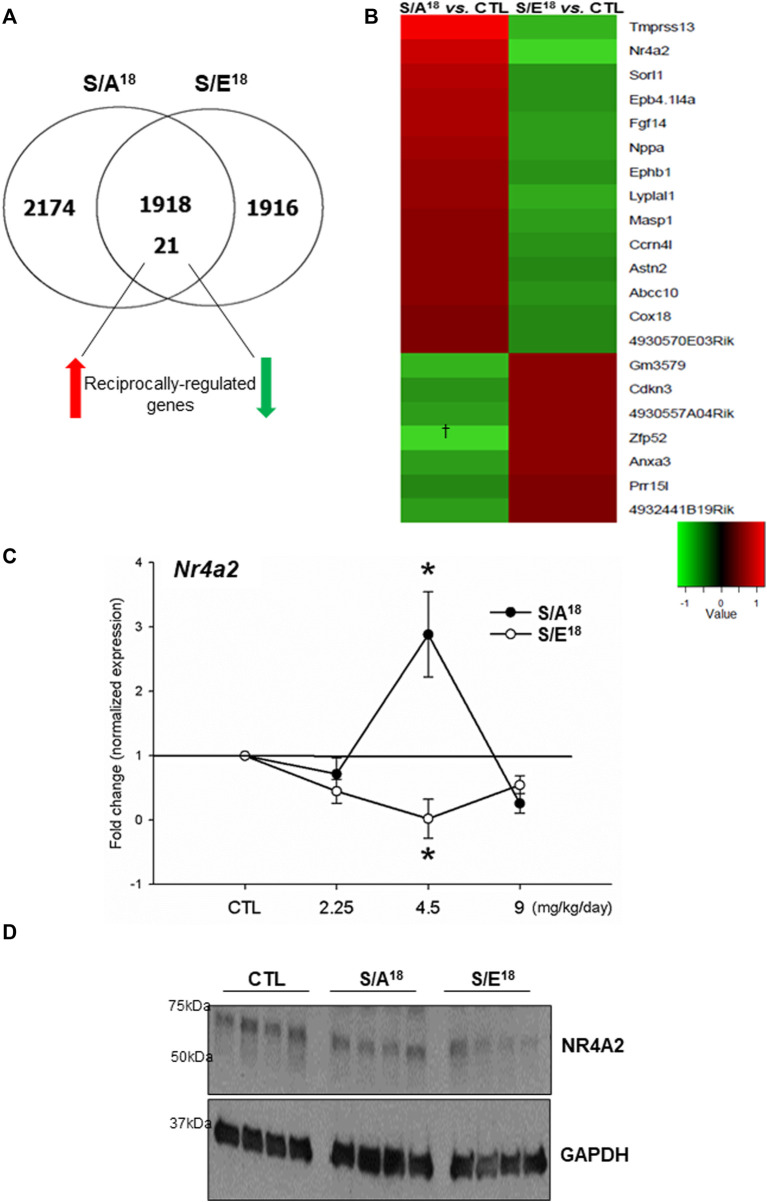
Microarray analysis of gene expression in the hearts of peptide treated mice at 4.5 mg/kg/day dose. **(A)**. Venn-diagram showing the segregation of differentially expressed genes relative to control. **(B)**. Heat map of the 21 reciprocally regulated genes (Red = up-regulated, Green = down regulated). **(C)**. *Nr4a2* mRNA expression in the heart at different doses (*p < 0.05 vs. CTL *n* = 5). **(D)**. Immunoblots of NR4A2 protein expression abundance in the heart at 4.5 mg/kg/day dose.

Microarrays were employed to examine gene expression profiles in the 4.5 mg/kg/day treated hearts. Overall, there were a total of 4113 differentially-expressed genes in the S/A^18^ and 3855 in the S/E^18^ peptide treated hearts relative to controls. Subsequently, these differentially-expressed genes were segregated into four distinct groups (Figure 4A). *i*). 1918 differentially-expressed genes in both S/A^18^ and S/E^18^ peptide-treated hearts regulated in a concordant manner relative to controls (Supplementary Table S3). *ii*). 21 differentially-expressed genes in both S/A^18^ and S/E^18^ peptide-treated hearts regulated in a discordant, or reciprocal manner relative to controls (Supplementary Table S4). *iii*). 2174 differentially-expressed genes in only S/A^18^ treated hearts (Supplementary Table S5). *iv*). 1916 differentially-expressed genes in only S/E^18^ treated hearts (Supplementary Table S6).

Since the discordantly regulated gene set may represent genes more directly modulated as a consequence of the single amino acid difference within the peptides and the opposing effects of the peptides on contractility, we focused on the transcripts that were reciprocally-regulated (≥1.2-fold and *p* ≤ 0.05, Supplementary Table S4) between S/A^18^ and S/E^18^ peptide-treated mice (Figure 4B). We confirmed the existence of reciprocal regulation of genes identified within this group (*Nppa, Ephb1, Epb4.1l4a*) at the 4.5 mg/kg/day dose (Supplementary Figure S6). Based on the reports of insulin stimulation of the Nr4a orphan nuclear hormone receptor subgroup expression and their role in regulating metabolic genes in skeletal muscles; we selected Nr4a2 (Nurr 1) as a candidate of interest (Maxwell et al., 2005; Chao et al., 2007; Wu et al., 2007; Pearen et al., 2008). Analysis of *Nr4a2* mRNA expression at different doses showed an expression profile concordant with the changes in cardiac contractility and validated our microarray data (Figure 4C). While analysis of NR4A2 protein expression in the heart at the 4.5 mg/kg/day dose did not show increased expression in S/A^18^ peptide-treated mice, a repression of NR4A2 expression was evident in S/E^18^ peptide-treated mice (Figure 4D).

**FIGURE 5:**
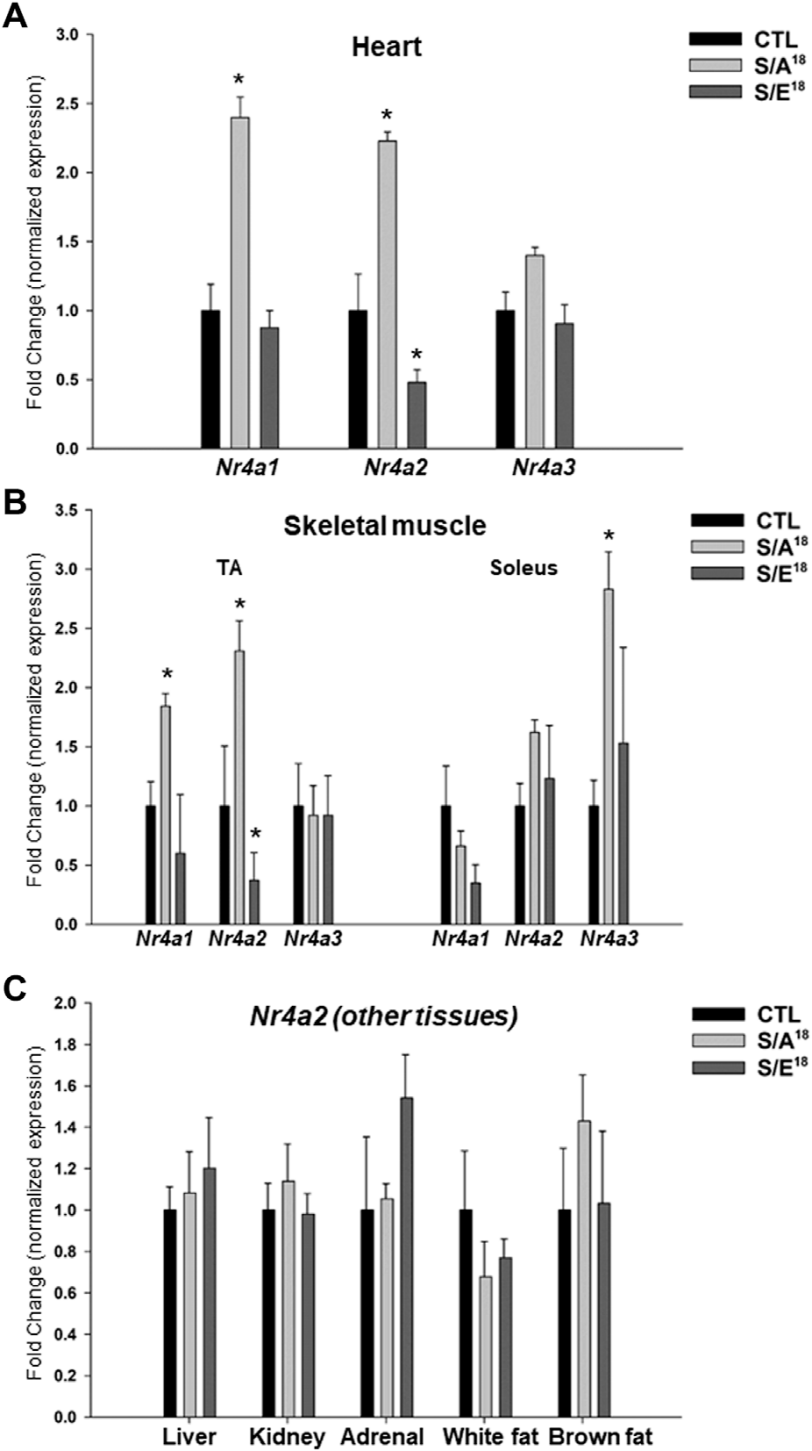
Analysis of NR4A family members mRNA expression in the heart, skeletal muscles, and other organs in peptide treated mice at 4.5 mg/kg/day dose. **(A)**. *Nr4a1,2,3* subgroup mRNA expression in the heart. **(B)**. *Nr4a1,2,3* subgroup mRNA expression in the *tibialis anterior* (TA) and *soleus* (Sol) muscles (*p < 0.05 vs. CTL, *n* = 5). **(C)**. *Nr4a2* mRNA expression in other tissues in peptide treated mice at 4.5 mg/kg/day dose.

Extending our analysis to the other Nr4a family members that were also identified as differentially-expressed genes in the S/A^18^ peptide-treated mice (Supplementary Table S3), we found a significant increase in *Nr4a1* and *Nr4a2* while *Nr4a3* expression was not significantly increased in the hearts of S/A^18^ treated mice (Figure 5A). To examine the effects of systemic MGF E-domain delivery we assayed Nr4a gene expression in the skeletal muscles and Nr4a2 expression in other tissues of the peptide treated mice. Reciprocal regulation of *Nr4a2* mRNA occurred in the *tibialis anterior* (TA), but with no change detected in the *soleus* (Figure 5B). As noted in the heart, *Nr4a1* and *Nr4a2* were significantly increased in the TA of S/A^18^ peptide treated mice but no change in the *soleus*. Interestingly, *Nr4a3* showed no sign of regulation in the TA, but was significantly increased in the *soleus* of S/A^18^ peptide treated mice. Analysis of *Nr4a2* expression in the adrenal gland, kidney, liver, white and brown fat, showed no sign of regulation by peptide treatment (Figure 5C).

**FIGURE 6:**
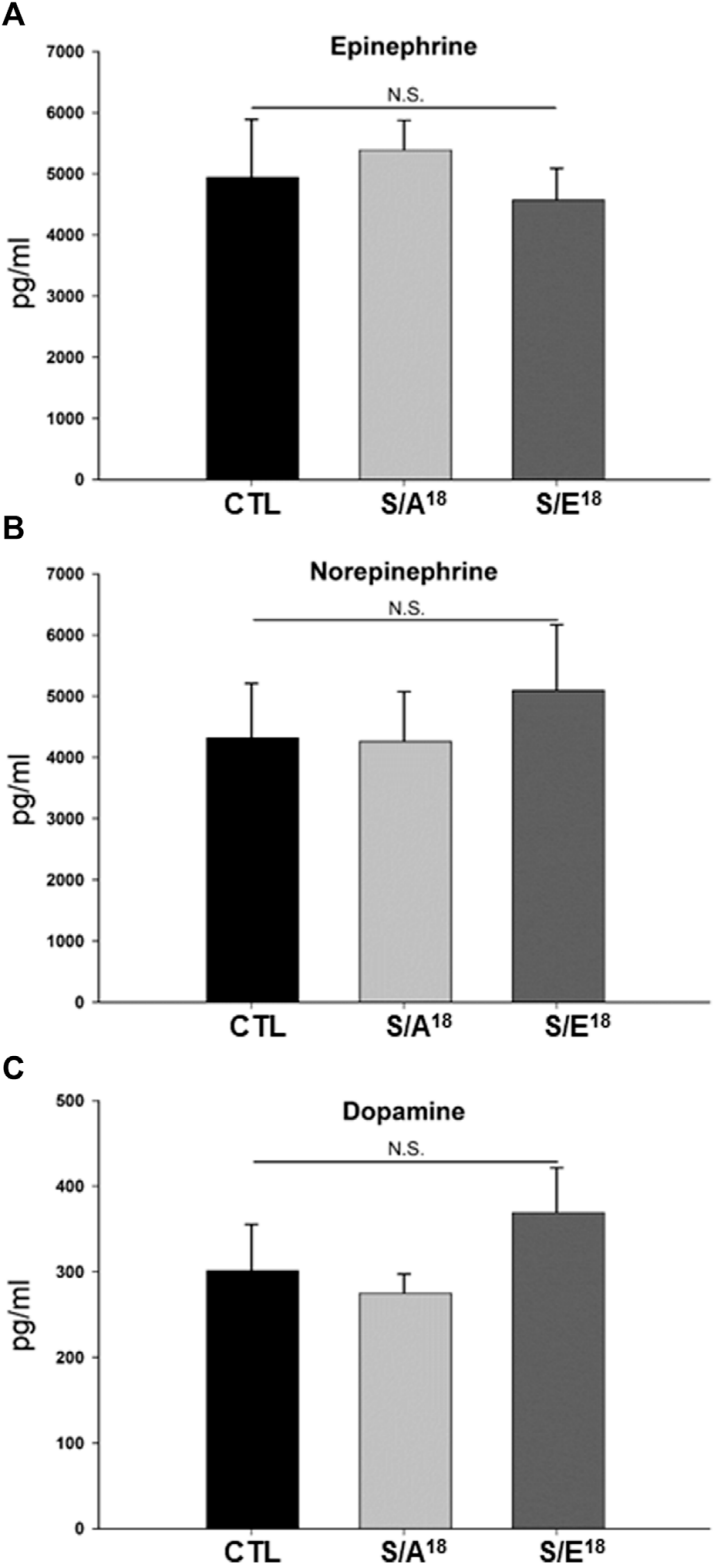
**(A–C)** Circulating levels of catecholamines in S/A^18^ and S/E^18^ peptide mice treated at 4.5 mg/kg/day dose for 2 weeks (*n* = 10).

Several studies have shown that Nr4a2 plays a role in dopaminergic neuron differentiation and the regulation of tyrosine hydroxylase expression which catalyzes the conversion of L-tyrosine to L-3,4-dihydroxyphenylalanine (L-DOPA) in the synthesis of dopamine. In addition, Nr4a2 expression is responsive to catecholamines in skeletal muscles and stress responses in the hypothalamus-pituitary-adrenal (HPA) axis (Saucedo-Cardenas et al., 1998; Kim et al., 2007; Pearen et al., 2008; Helbling et al., 2014). This prompted us to consider that the changes in contractile function and Nr4a2 expression in the heart could be mediated by changing levels of circulating catecholamines, either through the E-domain peptides crossing the blood brain barrier (Dluzniewska et al., 2005; Quesada et al., 2009) or acting upon the adrenal glands following systemic delivery. However, we found no change in circulating catecholamines based on peptide treatment compared to saline controls, suggesting the opposing actions noted on contractile function and gene expression may be occurring in the absence of neurohormonal regulation (Figure 6).

### E-domain Ser18 phosphorylation modulates NR4A family gene expression in response to membrane depolarization *in vitro*

**FIGURE 7:**
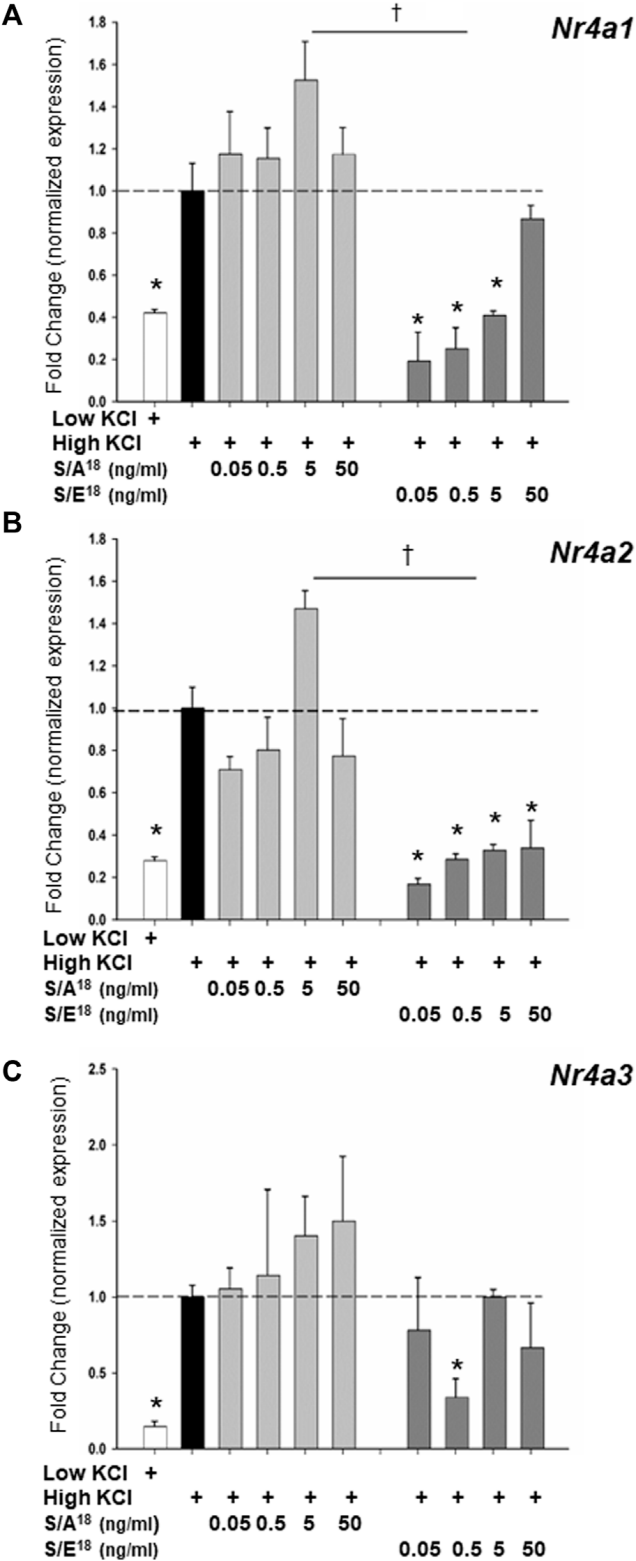
**(A–C)** Modulation of NR4A subgroup (*Nr4a1,2,3*) mRNA expression in HL-1 cardiac myocytes in response to peptide treatments following membrane depolarization. Membrane depolarization was induced by incubating cell in Krebs-Ringer buffer containing 84 mM KCl and 3 mM CaCl_2_ (High KCl) followed by peptide treatments for 1 h (*p < 0.05 vs. CTL in high KCl alone, ^*†*^ *p < 0.05* vs peptide at the same dose, *n* = 3 independent experiments).

**FIGURE 8:**
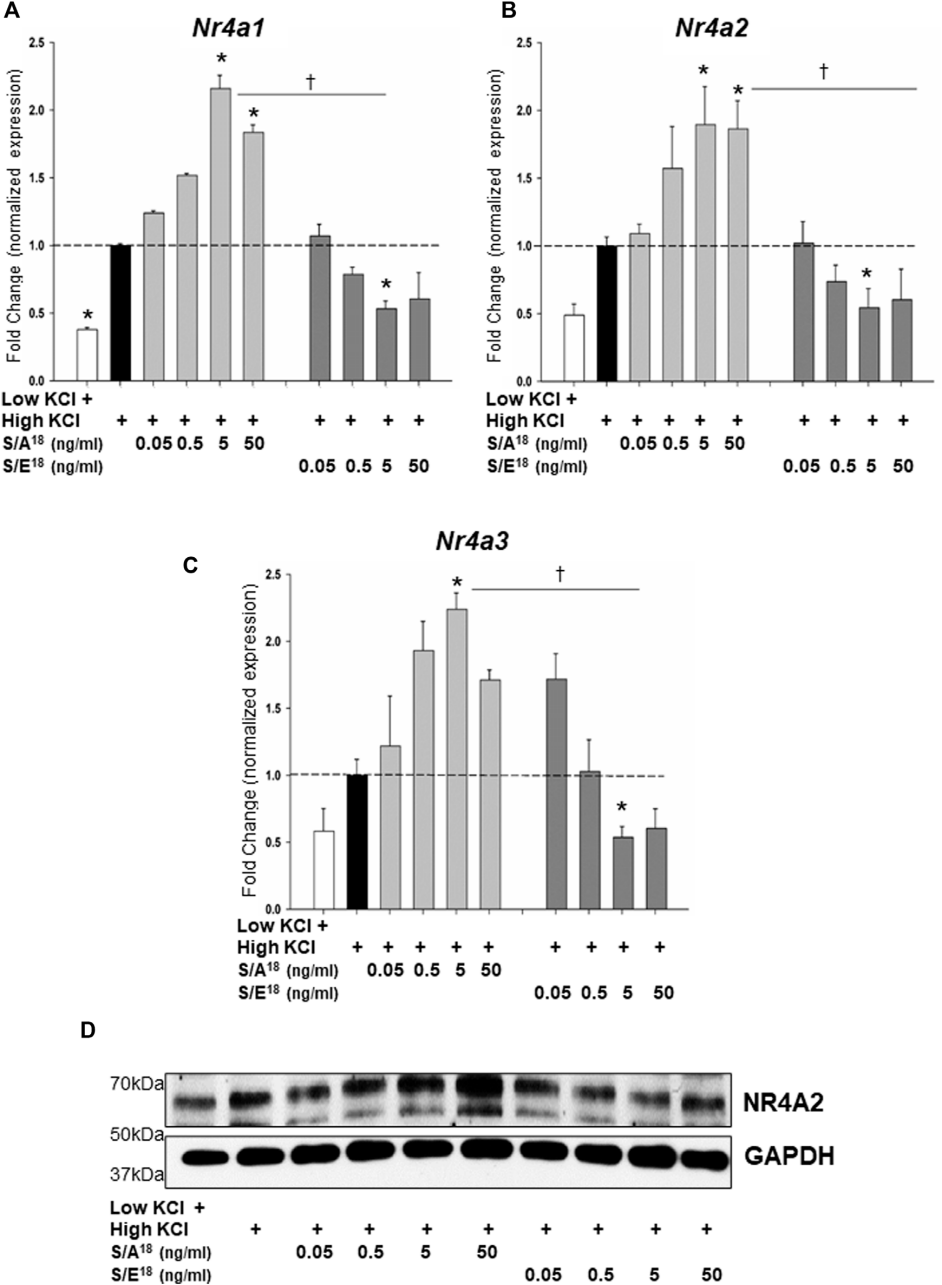
Modulation of NR4A subgroup (*Nr4a1,2,3*) mRNA expression in C_2_C_12_ myotubes in response to peptide treatments following membrane depolarization. Membrane depolarization was induced by incubating cell in Krebs-Ringer buffer containing 84 mM KCl and 3 mM CaCl_2_ (High KCl) followed by peptide treatments. **(A,B,C)**. *Nr4a1*, *Nr4A2* and *Nr4a3* mRNA expression at 1 h (*p < 0.05 vs CTL in high KCl alone, ^*†*^ *p < 0.05* vs peptide at the same dose, n = 3 independent experiments). **(D)**. NR4A2 protein expression in C_2_C_12_ myotubes in response to peptide treatments following membrane depolarization at 1 h.

To examine the dynamics of NR4A gene expression *in vitro*, we set out to recapitulate the actions of the E-domain peptides in both cardiac (HL-1) and skeletal muscle (C2C12) cell lines. Application of the S/A^18^ and S/E^18^ peptides to unstimulated cells grown in DMEM produced no significant effect on the Nr4a family mRNA expression (Supplementary Figure S7). However, membrane depolarization with KCl was sufficient to induce increases in *Nr4a* mRNA expression. Treatment with the S/A^18^ peptide at different concentrations following depolarization did not significantly augment Nr4a family mRNA expression in HL-1 cells (Figures 7A–C) but did significantly increase their expression at 5 ng/ml in C2C12 cells (Figures 8A–C). In contrast, treatment with the S/E^18^ peptide significantly repressed *Nr4a* expression at 5 ng/ml following membrane depolarization compared to both untreated and S/A^18^ peptide treated cells. This occurred in both cell lines with the exception for *Nr4a3* in the HL-1 cells (Figures 7A–C, Figures 8A–C). Analysis of NR4A2 protein expression in the C2C12 cells paralleled the significant changes in mRNA levels compared to the changes *in vivo* (Figure 8D). The discrepancy in our ability to detect an increase in NR4A2 protein *in vivo,* may be reflective of the diminishing effects of the S/A^18^ peptide towards the end of a 2-weeks treatment compared to the onset of an acute treatment *in vitro*. It may also be reflective of the unavoidable time differences involved in manipulating the mice during surgeries, recordings and obtaining tissues compared to being able to exert greater control over the onset and termination of experiments *in vitro*. Nevertheless, a consistent observation between these datasets is the apparent suppressive effect of the S/E^18^ peptide on Nr4a2 mRNA and protein expression. Together, these data indicate that MGF E-domain peptide treatment is not sufficient to elicit changes in Nr4a expression *per se*. However, when coupled with events associated with membrane excitability such as excitation-contraction coupling (ECC) and excitation-transcription coupling in muscle, its modulatory actions are unmasked and regulated by the state of Ser18 phosphorylation.

### E-domain Ser18 phosphorylation modulates 14-3-3 protein interactions

**FIGURE 9:**
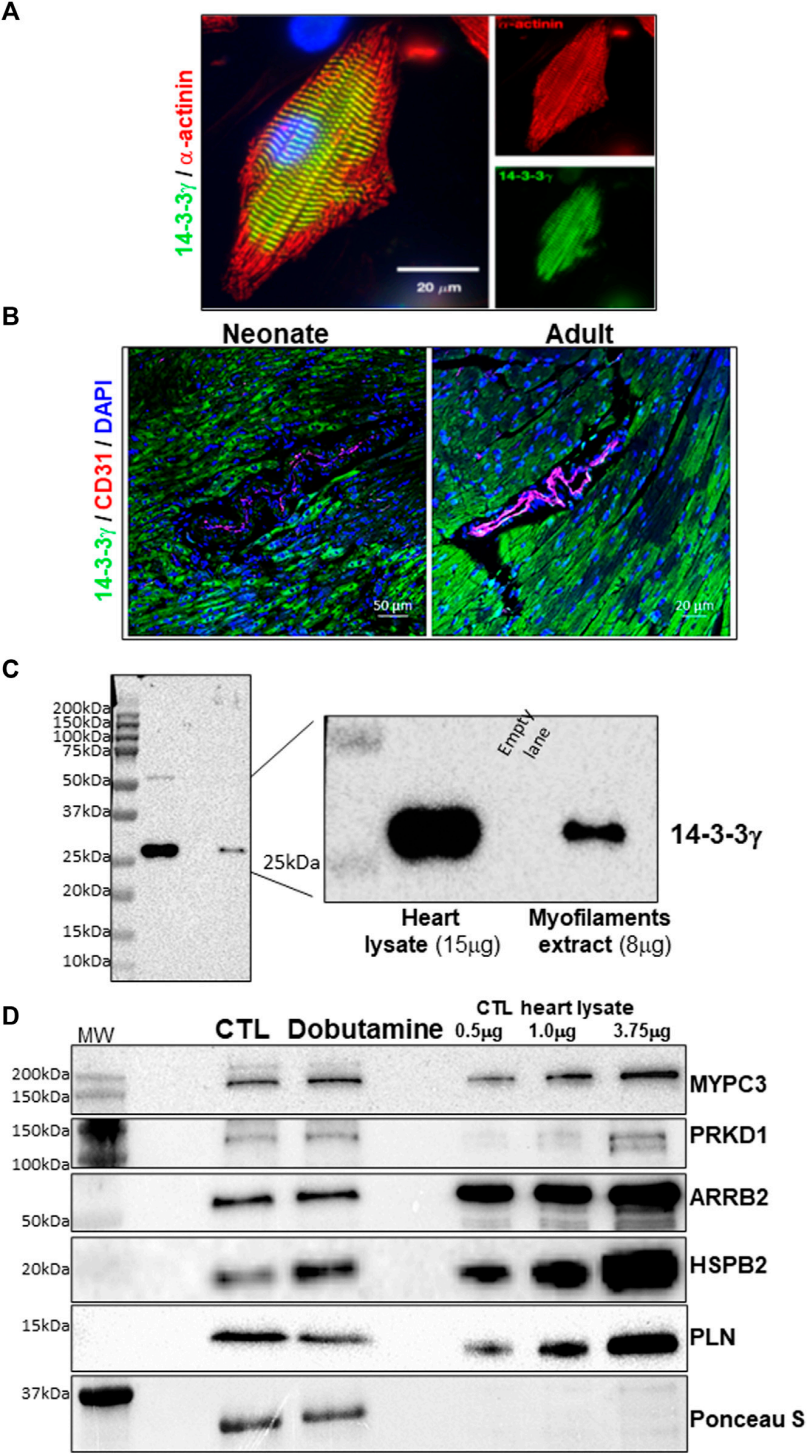
14-3-3γ subcellular distribution and 14-3-3 γ interactome in the heart. **(A)**. Immunofluorescence staining in cultured neonatal rat ventricular myocytes. Co-staining with 14-3-3γ specific antibody (*green*), α-actinin (*red*) and with DAPI (*blue*). **(B)**. Immunofluorescence staining of 14-3-3γ in the mouse heart at different ages (Neonate = 2 weeks and adult = 1 month). Co-staining with 14-3-3γ specific antibody (*green*), CD-31 (*red*) and with DAPI (*blue*). **(C)**. Immunoblotting mouse heart lysate and the myofilament fraction with 14-3-3γ specific antibody. **(D)**. 14-3-3γ affinity capture of client protein in heart lysates of saline and dobutamine injected mice. Representative immunoblots of affinity capture signal compared to varying amounts of total lysate for each protein.

Since Ser18 resides within a putative 14-3-3 binding motif within the MGF E-domain, we investigated whether the phosphomimetic peptides exert their effects on 14-3-3 protein interactions with proteins involved in contractile function. 14-3-3 proteins are expressed ubiquitously in all eukaryotes and in rodents and humans 7 isoforms exist. Thus, we sought to determine which 14-3-3 isoforms interact with the contractile apparatus by immunostaining neonatal rat ventricular myocytes (NRVM) with isoform specific antibodies. Immunofluorescence staining of 14-3-3 isoforms in isolated myocytes showed 14-3-3γ localized to the myofilaments in unstimulated myocytes (Figure 9A). All the other isoforms (α/β, ε, η, δ/ζ, τ) showed diffuse cytoplasmic and nuclear staining (Supplementary Figure S8). Histological analysis of 14-3-3γ expression in the mouse heart revealed 14-3-3γ was predominantly expressed in the cardiac myocytes and is abundant in the myofilament extracts prepared from the adult heart (Figures 9B,C).

To predict the potential for interactions with proteins involved in contractile function, we analyzed for the existence of putative 14-3-3 binding domains within major contractile and Ca^2+^-handing proteins. Comparisons across web-based resources (ELM and 14-3-3-Pred), uncovered several potential targets, but highlighted the sequences surrounding S^273^ in myosin binding protein C (MYPC3) and S^16^/T^17^ in phospholamban (PLN) in the mouse (Uniport ID, O70468 and P61014 respectively). Given the well-established role of phosphorylation at these sites in altering cardiac contractility, we performed 14-3-3 affinity capture of client proteins in heart lysates from saline and dobutamine injected mice to stimulate the β_1_-adrenergic receptor (β_1_-AR). To establish 14-3-3 isoform specific affinity capture, we expressed the 14-3-3 isoforms with a N-terminal His_6_ tag. 14-3-3γ affinity capture confirmed that 14-3-3γ interacts with MYPC3 and PLN and several signaling proteins with known 14-3-3 interactions (ARRB2, HSPB2, PRKD1) that have previously been shown to target the myofilaments (Qian et al., 2011; Edwards et al., 2012; Sin and Baillie, 2015; Ryba et al., 2017) (Figure 9D). Since the sequence surrounding the serine 16 phosphorylation site of HSPB2 contains a putative 14-3-3 binding consensus motif (RXXpXXP) (Yaffe et al., 1997), we examined the putative 14-3-3 binding sites in those proteins identified here using the *14-3-3 Pred tool* (Supplementary Figure S9). All the proteins identified contained strong putative phosphoserine sites with an Arg residue in the pS -2 position except for ARRB2. However, ARRB2 has a Pro residue in the pS +2 position like HSPB2 and has been shown to interact with 14-3-3 proteins (Crépieux et al., 2017).

**FIGURE 10:**
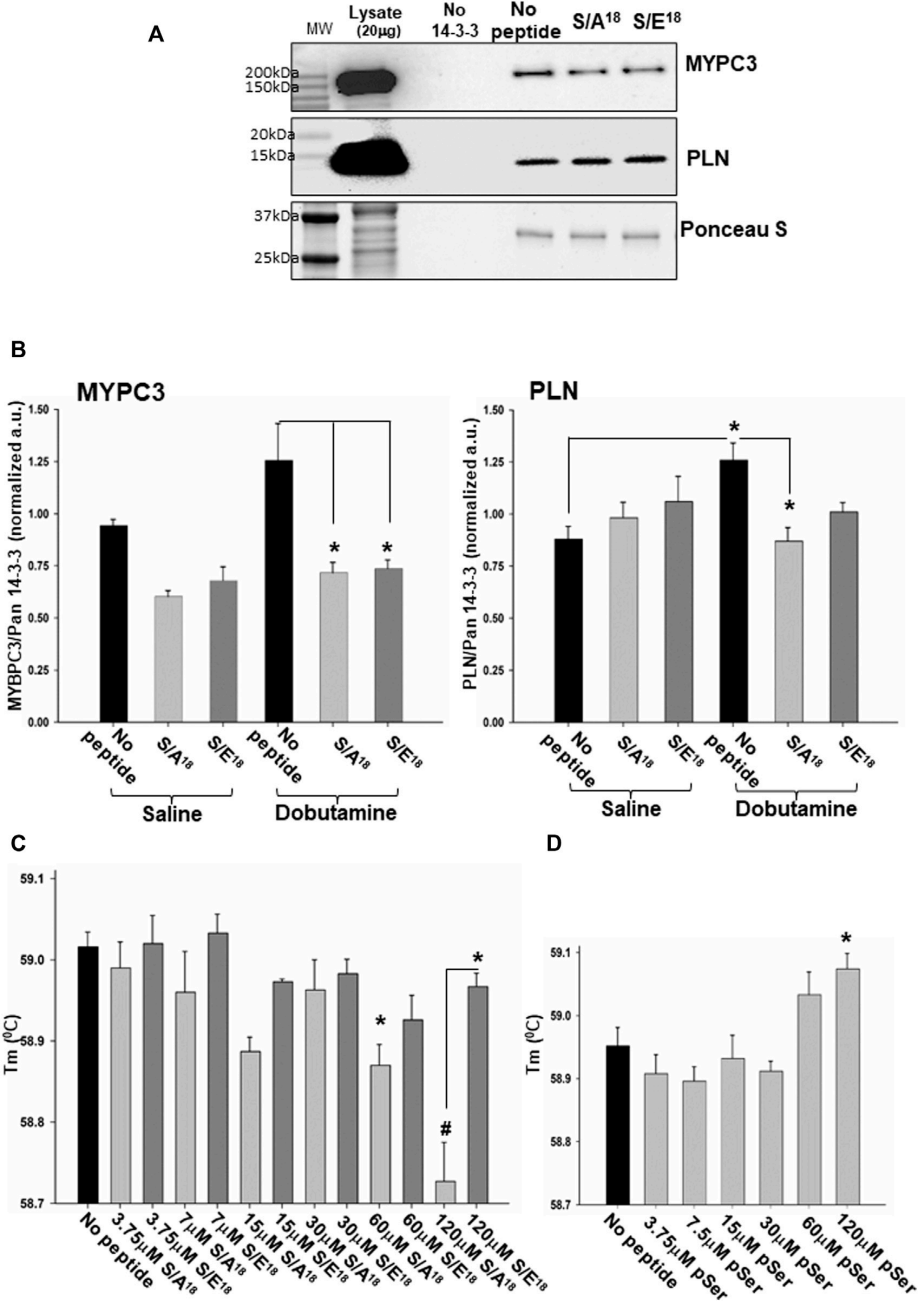
MGF E-domain peptide modulation of 14-3-3γ interactions. **(A)**. Representative immunoblot of 14-3-3γ capture of myosin binding protein C3 and phospholamban with S/A^18^ and S/E^18^ peptides in heart lysates of saline injected mice (lysate 20μg, peptides 20μM). **(B)**. Quantification of 14-3-3γ/MYPC3 and 14-3-3γ/PLN interactions in heart lysates of saline and dobutamine injected mice (*p < 0.05 vs no peptide, *n* = 4). **(C)**. Thermal stability of recombinant 14-3-3γ with varying concentrations of S/A^18^, S/E^18^ peptides. **(D)**. Thermal stability of recombinant 14-3-3γ with varying concentrations of pS^18^ E-domain peptide (*p < 0.05 vs no peptide CTL or between peptides, #*p < 0.01* vs no peptide, *n* = 3 independent experiments).

Consequently, we tested whether the MGF E-domain phosphomimetic peptides could modulate the interactions between MYPC3 and PLN within the 14-3-3γ interactome to potentially explain the peptide mediated effects on the contractile function observed (Figure 10A). Short-term β_1_-AR stimulation increased the ability of 14-3-3γ to interact with MYPC3 and PLN in heart lysates with the 14-3-3γ/PLN interaction reaching significance in the absence of phosphomimetic peptides. Incubating the heart lysates with phosphomimetic peptides during the affinity capture showed the S/A^18^ and S/E^18^ peptides tended to inhibit the 14-3-3γ/MYPC3 in control and significantly inhibited in β_1_-AR stimulated hearts to the same extent. (Figure 10B). However, neither peptide altered the interaction between 14-3-3γ and PLN in the controls, but the S/A^18^ peptide did significantly inhibit the 14-3-3γ/PLN interaction in β_1_-AR stimulated hearts, indicating peptide mediated differential regulation of this 14-3-3 client protein within the 14-3-3γ interactome.

Finally, to further examine the interaction of the E-domain peptides with 14-3-3γ, we analyzed the thermal stability of 14-3-3γ under constant pH and ionic strength. Thermal shift analysis showed the S/A^18^ peptide significantly destabilizes 14-3-3γ folding with increasing concentrations (Figure 10C). We found no significant effect of the S/E^18^ peptide, but at the highest concentration there was a significant difference between the two peptides. Finally, we ascertained whether phosphorylation of Ser18 altered the E-domain peptide interaction with 14-3-3γ as opposed to mimicking the charge change associated with phosphorylation. Incubating 14-3-3γ with a stabilized phospho-Ser18 E-domain peptide stabilizing 14-3-3γ folding with increasing concentrations, thereby produced an opposite effect compared to the S/A^18^ peptide. These data suggest a potential mechanism by which the MGF E-domain may act as an allosteric modulator by altering the stability of 14-3-3γ protein-protein interactions depending on the state of Ser18 phosphorylation within its 14-3-3 motif.

## Discussion

Interest has grown with respect to the actions of IGF-1 isoforms expressed in various tissues (Hill et al., 2003; Dluzniewska et al., 2005; Barton et al., 2010; Mavrommatis et al., 2013). The mechanisms by which the various E-domains exert their biological actions are largely unknown but may account for isoform function. Our data show for the first time that phosphorylation sites may regulate the activity of the MGF E-domain and its interactions with 14-3-3 proteins. Since this pattern of alternate splicing is conserved, our data suggest conservation of these motifs may be functional. This additional level of regulation has not been identified previously using either genetic or peptide-based approaches (Yang and Goldspink, 2002; Barton et al., 2010; Mavrommatis et al., 2013; Brisson et al., 2014; Fornaro et al., 2014). As such, the MGF E-domain peptide analog provides a useful tool to perturb the structure and function of the E-domain and provide insight into the potential actions of the endogenous MGF E-domain without the confounding effects of IGF-1.

One of the most pronounced physiologic responses to manifest with the phosphomimetics peptides were the opposing effects on cardiac contractile function in the absence of adverse remodeling. Physiologically, contractility is regulated at several extrinsic and intrinsic levels, including circulating neurohormones, autonomic regulation, the length-tension relationship, and genetic background all regulate the events of ECC. While our analysis of circulating catecholamines mitigates concerns related to secondary effects associated with catecholamine synthesis or secretion with systemic delivery, it does not entirely rule out peptide effects on autonomic regulation or other neurohormones. Given the ability of the E-domain peptide to cross the blood brain barrier, the influence over the autonomic regulation, still needs to be addressed. Nonetheless, we have previously reported beneficial effects associated with cardiac restricted delivery of a stabilized human MGF E-domain analog *via* peptide eluting polymeric microstructures, which supports the notion that the E-domain peptides exert direct effects on the myocardium (Peña et al., 2015). Additionally, we have also shown that treatment with exogenous FITC-labeled MGF E-domain peptide can accumulate within cardiomyocytes (Mavrommatis et al., 2013). The acute effects on excitation-transcription coupling reported here *in vitro*, combined with other studies suggesting cellular uptake and even a nuclear presence for the E-domain peptide, strongly suggest that the MGF E-domain peptides may act as cell penetrating peptides. While the precise mechanism by which the peptides enter the cell is unknown, the polybasic motif (^14^RRK^16^) may enable translocation across the cell membrane as peptides that contain one or more arginines can form guanidine-mediated bidentate hydrogen bonds with anionic groups on the cell surface to facilitate their internalization (Shimoni and Glusker 1995).

The NR4A family members function as orphan nuclear receptors and their immediate early expression is induced by cell stressors and membrane receptor agonists. Currently, no endogenous ligands have been identified and their activity is thought to be regulated by their expression. Expression of all three Nr4a family members is induced by stimuli that include G-protein agonists, tyrosine kinase receptors, cAMP/PKA activation, mechanical stress, and exercise (Mahoney et al., 2005; Wu et al., 2007; Pearen et al., 2008). While the actions of NR4A2 in the heart are unknown, microarray-based studies show its expression is increased with exercise and decreased in models of heart failure (Colak et al., 2009; Galindo et al., 2009). More germane however are array data indicating that cardiac restricted over-expression of the IGF-1R increases *Nr4a2* expression, suggesting a link between IGF-1R signaling and NR4A expression (McMullen et al., 2004). Reinforcing this link further are the studies suggesting an auto-regulatory feedback loop between NR4A1 and IGF-1 expression exists to modulate skeletal muscle fiber size (Tontonoz et al., 2015). Therefore, the rationale for our selection of NR4A2 as a candidate of interest, was the thought the peptides may bind to the ligand binding domain of the Nr4a proteins and act as cognate nuclear receptors. However, our treatments of unstimulated HL-1 cells (Supplementary Figure S7) did not support this hypothesis. Consequently, these data along with the opposing effects on cardiac contractility and *Nr4a* gene expression *in vivo* caused us to consider whether the electrical or mechanical events associated with muscle contraction were required to elicit the modulatory actions of the peptides in regulating the *Nr4a* gene expression. The data which demonstrate membrane depolarization in muscle cell lines is sufficient to induce *Nr4a* subfamily expression but necessary to elicit the peptide actions, indicate that the E-domain peptides modulate membrane initiated signaling events rather than stimulating them *per se*. Together these data may also help reconcile the conflicting data in other studies that have shown direct application of the human E-domain analog to various cell types, either induces changes in signaling intermediaries and cellular physiology, or exerts no effect *in vitro* (Yang and Goldspink, 2002; Mills et al., 2007; Collins et al., 2010; Deng et al., 2011; Fornaro et al., 2014). We speculate these differences may exist either due to the state of cell membrane electrical excitability in the cells studied or potentially post-translational modification of the peptide within the intracellular environment. These points align with our observed effects on the phosphorylation of intracellular signaling protein in the hearts of peptide treated mice, which suggest that the E-domain couples to and modulates signaling pathways through interactions with 14-3-3 protein signaling complexes *via* phosphorylation of residues within its 14-3-3 binding motif.

14-3-3 proteins (14-3-3s) are a family of highly conserved proteins that regulate many cellular processes by interacting with a diverse array of client proteins. Once bound they generally modulate client protein activation, inhibition, structural stabilization and intracellular localization (Obsil and Obsilova 2011). In striated muscle, most studies have focused on 14-3-3 protein involvement in the signaling cascades leading to transcriptional changes, apoptosis, autophagy, ER stress, and cell cycle regulation. Although a subset of studies has examined 14-3-3 interactions with proteins and events associated with ECC in the heart, these have largely focused on the trafficking of ion channels and their activity in heterologous cell lines (Thompson and Goldspink, 2022). Our data demonstrate for the first time that a 14-3-3 isoform interactome not only encompasses signaling proteins, but also integrates contractile proteins with proteins involved in intracellular calcium handling in the myocardium. We also show a potential mechanism by which MGF E-domain peptides modulate protein-protein interactions within the myofilament targeted interactome through altering 14-3-3 protein stability. A similar effect has been attributed to a phosphopeptide analog corresponding to the 14-3-3 binding motif surrounding Ser16 in the small heat shock protein B2 (HSP20). Linked to a protein transduction domain (PTD) from the HIV Tat protein, the peptide was used to elucidate the role of HSP20 in smooth muscle relaxation and actin cytoskeletal remodeling (Flynn et al., 2003; Dreiza et al., 2005). Analysis of the phosphorylated HSP20/14-3-3 complex showed it stabilized the 14-3-3 proteins and impeded their proteolytic degradation (Sluchanko et al., 2012). Even though the consequences associated with altering the stability of 14-3-3 interactions *in vivo* are complex, our data suggest that destabilizing 14-3-3 interactions with the S/A^18^ peptide may lead to the activation of kinases that might otherwise be bound and inactivated by 14-3-3 proteins. Changing the stability of 14-3-3 protein interactions with c-Raf are known to alter its autoinhibited state or activation through 14-3-3 mediated dimerization of the RAF kinase domain (Obsilova and Obsil 2020). Likewise, while the overall effect of the S/E^18^ peptide appears to be inhibitory despite no clear effect on 14-3-3 stability, we cannot rule out the possibility of competitive inhibition by this peptide for 14-3-3 binding sites on target proteins within the 14-3-3γ interactome or other 14-3-3 isoform interactomes. Nevertheless, the opposing actions of the phosphomimetic MGF E-domain peptides we uncovered based on single amino acid substitutions within a biologically functional motif, tend to suggest allosteric modulation as a mechanism of action.

While it is still unknown whether 14-3-3 protein interactions may be involved in orchestrating events associated with myofilament activation, their interactions with membrane pumps involved in intracellular calcium homeostasis (sodium/calcium exchanger, plasma membrane calcium-transporting ATPase) have been defined mainly in heterologous cell lines (Rimessi et al., 2005; Pulina et al., 2006). Nevertheless, the most compelling examination of 14-3-3 interactions in the regulation of contractility to date, pertain to their interaction with Ser16 and Thr17 on phospholamban (PLN) (Menzel et al., 2020). Here, the 14-3-3/PLN interaction was promoted following β-adrenergic (β_1_ and β_2_) stimulation and shown to slow the kinetics of PLN dephosphorylation by masking the phosphosites on PLN. We consistently identified PLN in our 14-3-3γ interactome and detected an increased interaction following β_1_-adrenergic stimulation, indicating changes in the 14-3-3γ interactome are responsive to physiologic stimulation.

MYPC3 phosphorylation plays a regulatory role in cardiac contractility by binding both myosin and actin and mediates myosin crossbridge kinetics through the phosphorylatable regulatory domain in its N-terminus region (Solís and Solaro 2021). Mice that express a phosphorylation deficient MYPC3 (S273A/S282A/S302A) develop cardiac hypertrophy, diastolic dysfunction, stiffer ventricles, slower relaxation kinetics and exercise intolerance compared to wild type or phosphomimetic (S273D/S282D/S302D) controls (Rosas et al., 2015). In addition to the regulatory role phosphorylation at these sites in MYPC3 plays on contractile function, their phosphorylation also appears to be cardioprotective following ischemia, reperfusion injury and prevents proteolytic cleavage of the N-terminus of MYPC3 (Sadayappan et al., 2006). Even though we did not define the specific sites on MYPC3 that 14-3-3γ interacts with, the 14-3-3 Pred tool predicts the sequence surrounding S273 to be a strong 14-3-3 site (0.753), S282 a weaker site (0.515), S302 weaker still (0.407) and S307 stronger (0.593). While other potential strong 14-3-3 sites exist elsewhere in MYPC3, calpain cleavage of the N-terminus of MYPC3 appears to occur following dephosphorylation between T272-R280 *in vivo* (Barefield et al., 2019). Thus, it is interesting to speculate that the 14-3-3 proteins binding to the site surrounding S273, may mask this phosphoserine to modulate the accessibility of modifying enzymes such as kinases, phosphatases or proteases thereby adding an additional level of regulation not previously recognized.

Our data point toward a potential mechanism by which the E-domain phosphomimetics may act as allosteric modulators of 14-3-3 protein-protein interactions to modulate cardiac contractility. Defining that the 14-3-3 proteins interact with the force producing proteins of the contractile apparatus and calcium handling proteins as terminal effectors, provides further insight into the integration of targeted proteins and signaling intermediaries involved in the regulation of cardiac contractility. While defining these potential mechanisms and primary effects is not always useful within the context of a disease background, extending these observations in healthy mice to our prior studies with myocardial infarction, may further explain the cardioprotective actions and improvements in contractile function found with E-domain peptide delivery (Mavrommatis et al., 2013; Shioura et al., 2014; Peña, et al., 2015). We acknowledge a limitation to the present study is the lack of data demonstrating the endogenous E-domain is phosphorylated in the infarcted heart. However, as our study provides the first indication that functionally relevant motifs exist within the E-domain region of the MGF isoform, strategies and tools directed towards examining this modification are now warranted based on the actions of the E-domain peptide. If identified, it will also provide further support for use of peptide as a tractable approach to investigate the mechanistic actions of the E-domains. Thematically analogous to the C-peptide of pro-insulin which was thought to be biologically inert for decades but is now recognized to exert physiological effects (Yosten et al., 2014), we posit that the MGF E-domain may act as an intracrine component of this IGF-1 isoform that plays a regulatory role in modulating the 14-3-3 protein interactomes in cells.

## Data Availability

The original contributions presented in the study are included in the article/Supplementary Materials, further inquiries can be directed to the corresponding author.
