# Supplementary material for: Mechano-growth factor E-domain modulates cardiac contractile function through 14-3-3 protein interactomes
Source: Front Physiol. 2022 Nov 16;13:1028345. doi: 10.3389/fphys.2022.1028345 (PMC9709209; doi:10.3389/fphys.2022.1028345)
Supplement: Supplementary file 1 [file DataSheet1.PDF]

Supplementary Materials

Supplementary Figure 1

A

```
Human   1     MGKISSLPTQLFKCCFCDFLKVKMHTMSSSHLFYLALCLLTFTSSATAGPETLCGAELVD  60
              MGKISSLPTQLFK C CDFLK+K+H MSSSHLFYLALCLLTFTSSATAGPETLCGAELVD
Rat     1     MGKISSLPTQLFKICLCDFLKIKIHIMSSSHLFYLALCLLTFTSSATAGPETLCGAELVD  60

Human   61    ALQFVCGDRGFYFNKPTGYGSSSRRAPQTGIVDECCFRSCDLRRLEMYCAPLKPAKSARS  120
              ALQFVCG RGFYFNKPTGYGSS RRAPQTGIVDECCFRSCDLRRLEMYCAPLKP KSARS
Rat     61    ALQFVCGPRGFYFNKPTGYGSSIRRAPQTGIVDECCFRSCDLRRLEMYCAPLKPTKSARS  120

Human   121   VRAQRHTDMPKTQKYQPPSTN-KNTKSQRRKGSTFEERK  158
              +RAQRHTDMPKTQK QP ST+ K    +RRKGST EE K
Rat     121   IRAQRHTDMPKTQKSQPLSTHKKRKLQRRRKGSTLEEHK  159


Human   1     MGKISSLPTQLFKCCFCDFLKVKMHTMSSSHLFYLALCLLTFTSSATAGPETLCGAELVD  60
              MGKISSLPTQLFK C CDFLK+K+H MSSSHLFYLALCLLTFTSS TAGPETLCGAELVD
Mouse   1     MGKISSLPTQLFKICLCDFLKIKIHIMSSSHLFYLALCLLTFTSSTTAGPETLCGAELVD  60

Human   61    ALQFVCGDRGFYFNKPTGYGSSSRRAPQTGIVDECCFRSCDLRRLEMYCAPLKPAKSARS  120
              ALQFVCG RGFYFNKPTGYGSS RRAPQTGIVDECCFRSCDLRRLEMYCAPLKP K+ARS
Mouse   61    ALQFVCGPRGFYFNKPTGYGSSIRRAPQTGIVDECCFRSCDLRRLEMYCAPLKPTKAARS  120

Human   121   VRAQRHTDMPKTQKYQPPSTNKNTKSQ-RRKGSTFEERK  158
              +RAQRHTDMPKTQK    STNK TK Q RRKGSTFEE K
Mouse   121   IRAQRHTDMPKTQKSPSLSTNKKTKLQRRRKGSTFEEHK  159
```

B

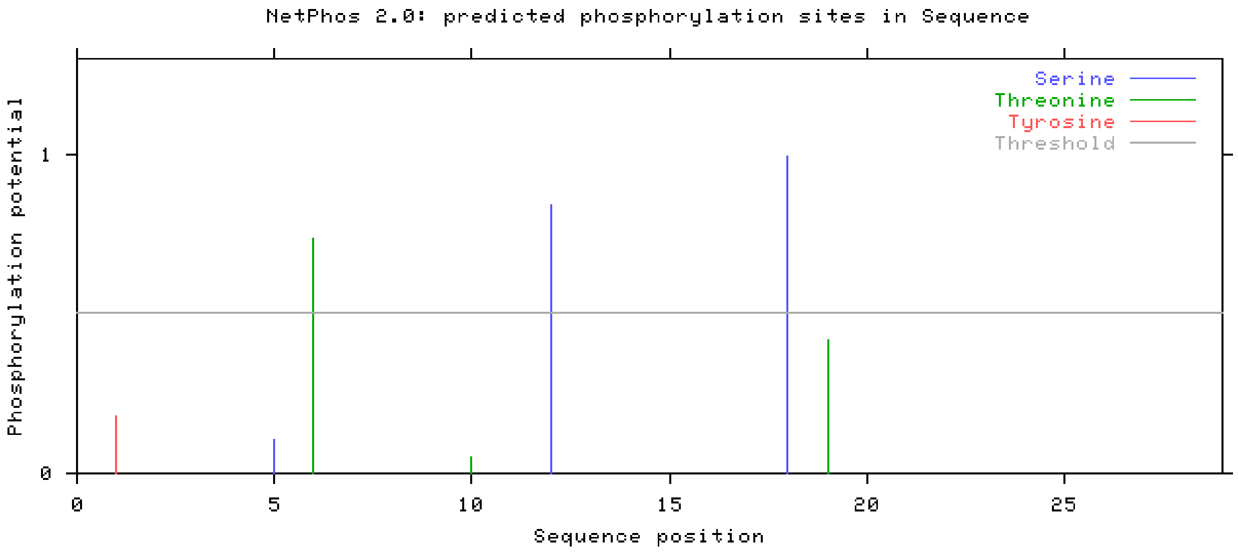

C

Serine predictions

Name	Pos	Context	Score	Pred
Sequence	5	YQPPSTNKN	0.103	.
Sequence	12	KNTKSQRRK	0.842	*S*
Sequence	18	RRKGSTFEE	0.994	*S*

Threonine predictions

Name	Pos	Context	Score	Pred
Sequence	6	QPPSTNKNT	0.734	*T*
Sequence	10	TNKNTKSQR	0.048	.
Sequence	19	RKGSTFEER	0.418	.

D

Method: NetPhosK without ESS filtering:
Query: Sequence YQPPSTNKNTKSQRRKGSTFEERK

Site	Kinase	Score
S-5	PKC	0.78
T-6	PKC	0.80
T-10	PKG	0.51
S-12	DNAPK	0.55
S-12	PKC	0.90
S-18	RSK	0.60
S-18	PKA	0.75
S-18	PKG	0.68
T-19	PKC	0.76

Supplementary Figure 1. A. Alignment of human, rat and mouse MGF proteins. Grey shaded region corresponds to entire E-domain; red font corresponds to the sequence of the unique region of the MGF E-domain. **B.** Potential phosphorylation sites within the 24 amino acid unique region of the human MGF E-domain identified using the NetPhos prediction program. Vertical lines that extend above the threshold indicate putative amino acid phosphorylation targets. **C.** Serine and threonine phosphorylation predictions and scores within the unique region of the human MGF E-domain. The serine at position 18 within the putative 14-3-3 binding domain received the highest score (red). **D.** NetPhosK analysis of specific kinase phosphorylation in the unique region of the human MGF E-domain. PKA phosphorylation of Ser18 received the higher score (red).

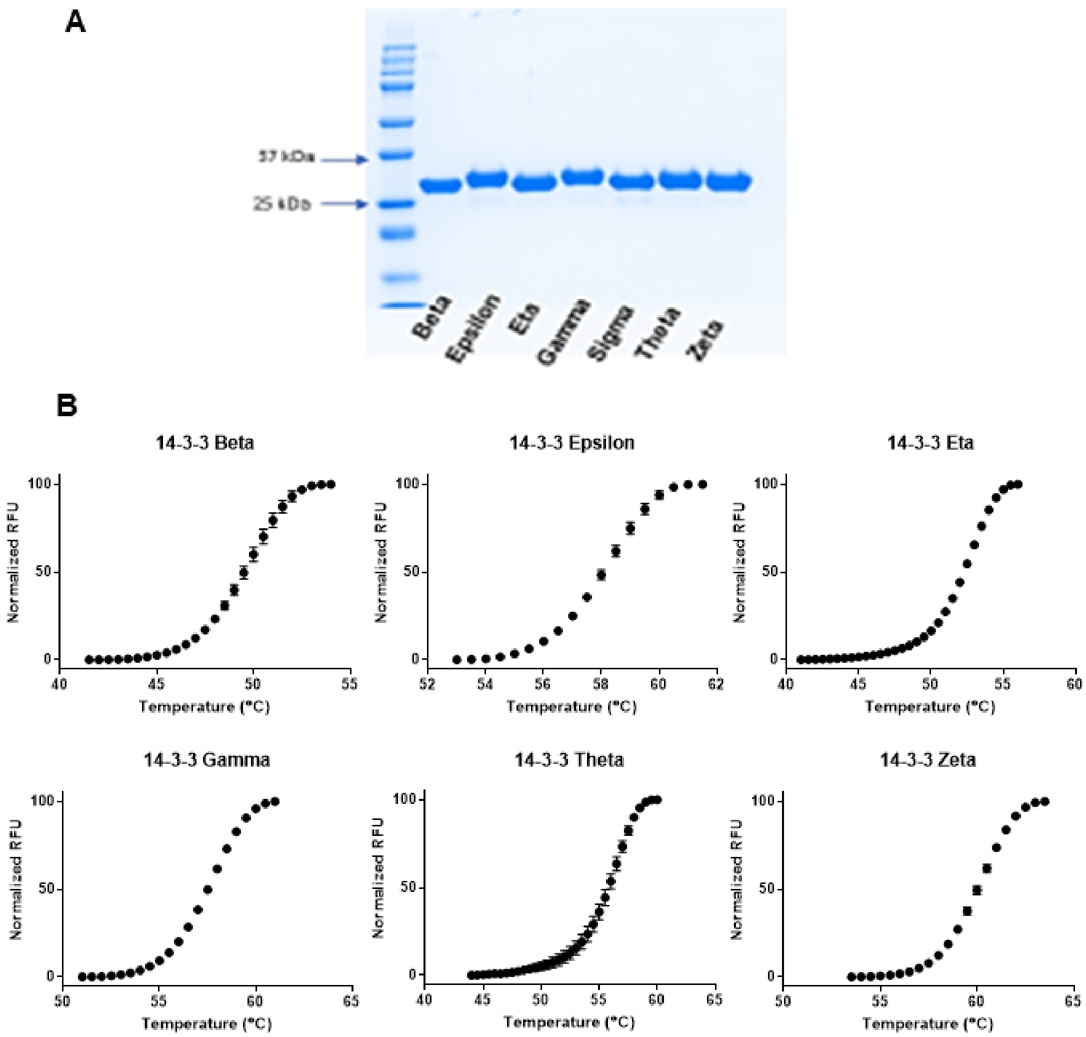

Supplementary Figure 2. A. Electrophoresis analysis of recombinantly expressed 14-3-3 isoforms (coomassie blue stain). **B.** Thermal shift analysis of each isoform to ensure correct folding (except sigma). For initial screening of recombinant 14-3-3 isoforms, each 20 µl reaction volume typically contained 10 µM final concentration of each 14-3-3 isoform diluted in Milli-Q water with 1 µl of 1:40 Milli-Q-diluted dye (GloMelt Thermal Shift Dye, Kit 33021-1, Biotium).

Supplementary Figure 3

A

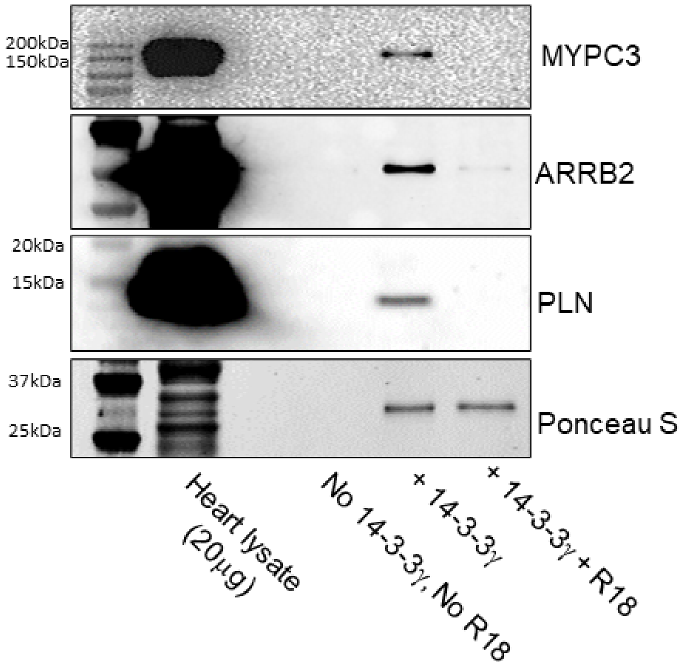

B

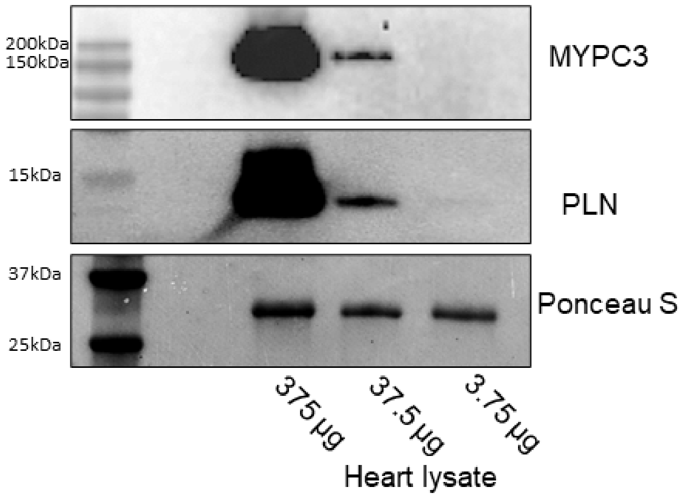

Supplementary Figure 3. Validation of 14-3-3 affinity capture of client proteins in heart lysates **A.** Negative controls were beads only (no 14-3-3γ) and blocking with R18 peptide. **B.** Affinity capture incubated with varying amounts of input lysate. Ponceau S band shows the 14-3-3γ on the beads.

Supplementary Figure 4

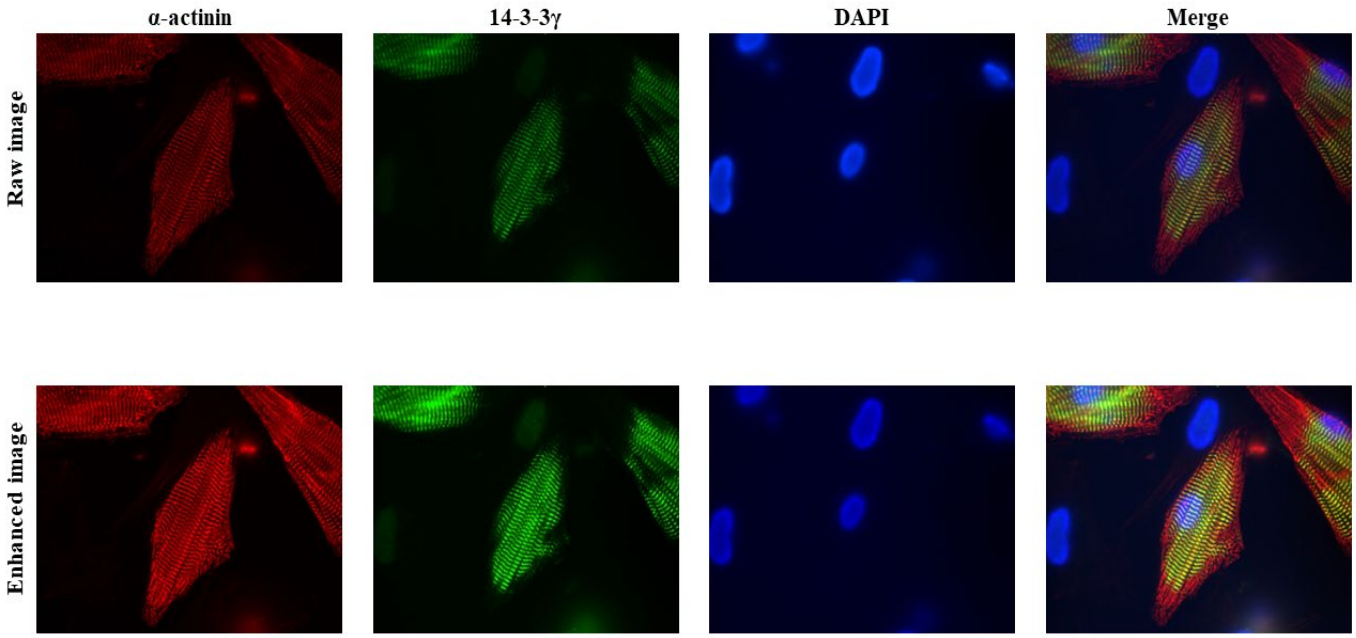

Supplementary Figure 4. Immunofluorescence staining in cultured neonatal rat ventricular myocytes. Co-staining with 14-3-3γ specific antibody (*green*), a-actinin (*red*) and with DAPI (*blue*). Top row depicts the raw, uncropped images captured by the microscope. Bottom row shows enhanced images using the auto brightness/contrast adjust options from the Fiji (ImageJ) software.

Supplementary Figure 5

A

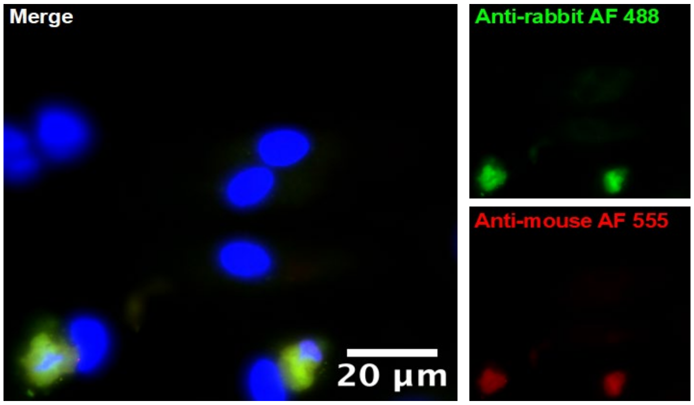

B

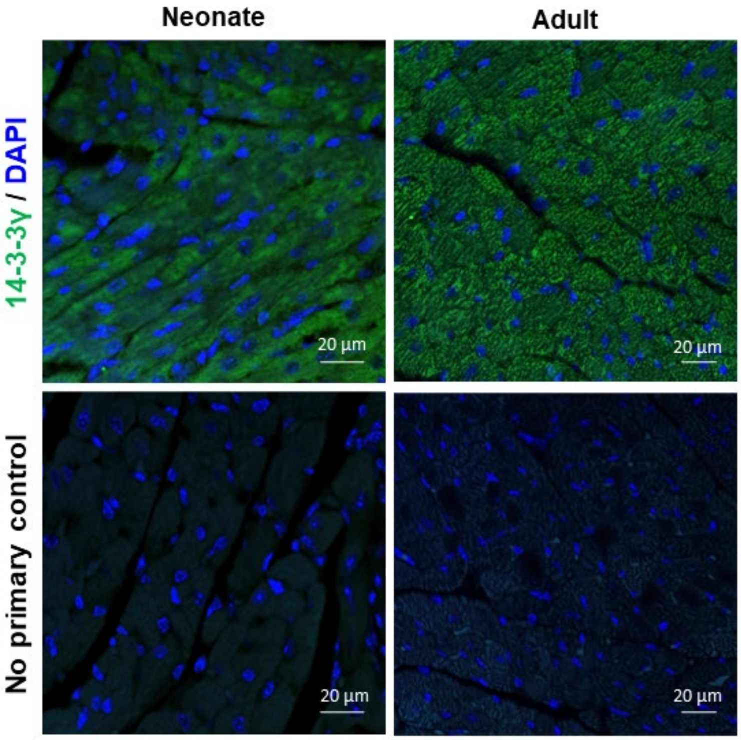

Supplementary Figure 5. Validation of 14-3-3 immunofluorescence staining in neonatal ventricular cardiac myocytes (NRVM) and the mouse heart. **A.** Secondary antibody staining alone in NRVMs, anti-rabbit AF488 (green) anti-mouse AF555 (red) and DAPI (blue). **B.** Upper panel shows 14-3-3γ expression (green) and nuclear counterstaining (blue, DAPI) in neonatal and adult mice. Bottom panel shows secondary antibody staining alone in mouse heart sections, anti-rabbit AF568 (green) and DAPI (blue).

Supplementary Figure 6

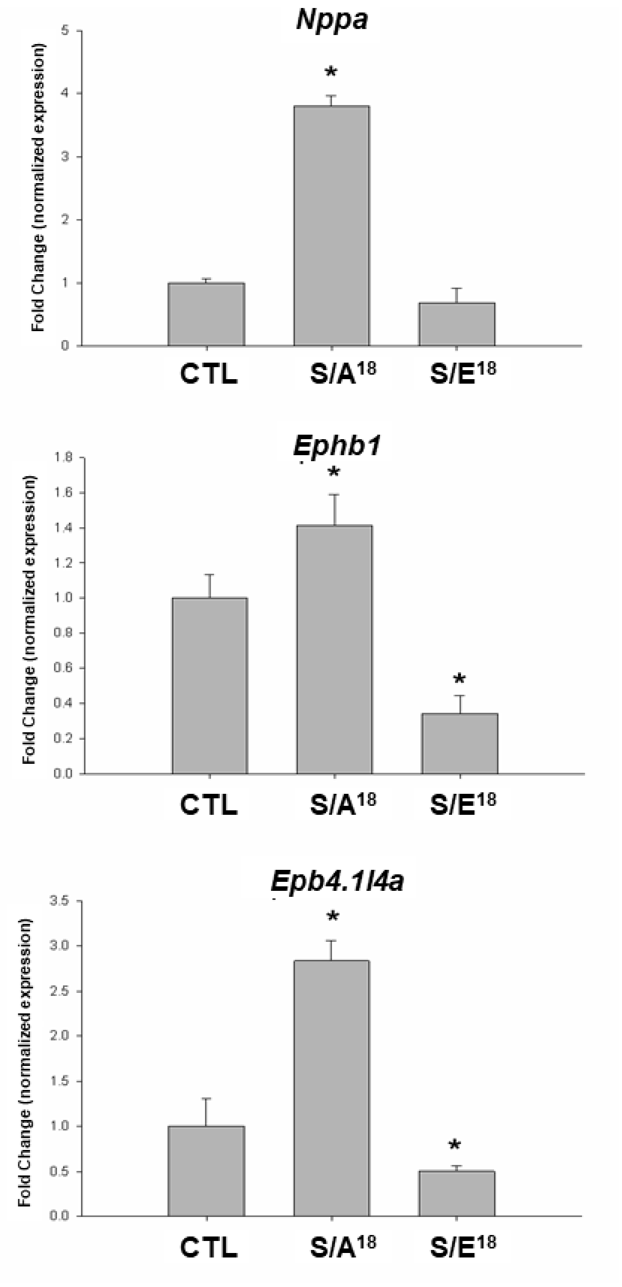

Supplementary Figure 6. Analysis and validation of reciprocally regulated genes in the hearts of peptide treated mice (4.5mg/kg/day) identified in the microarray. *P<0.05 *vs.* CTL, n=5.

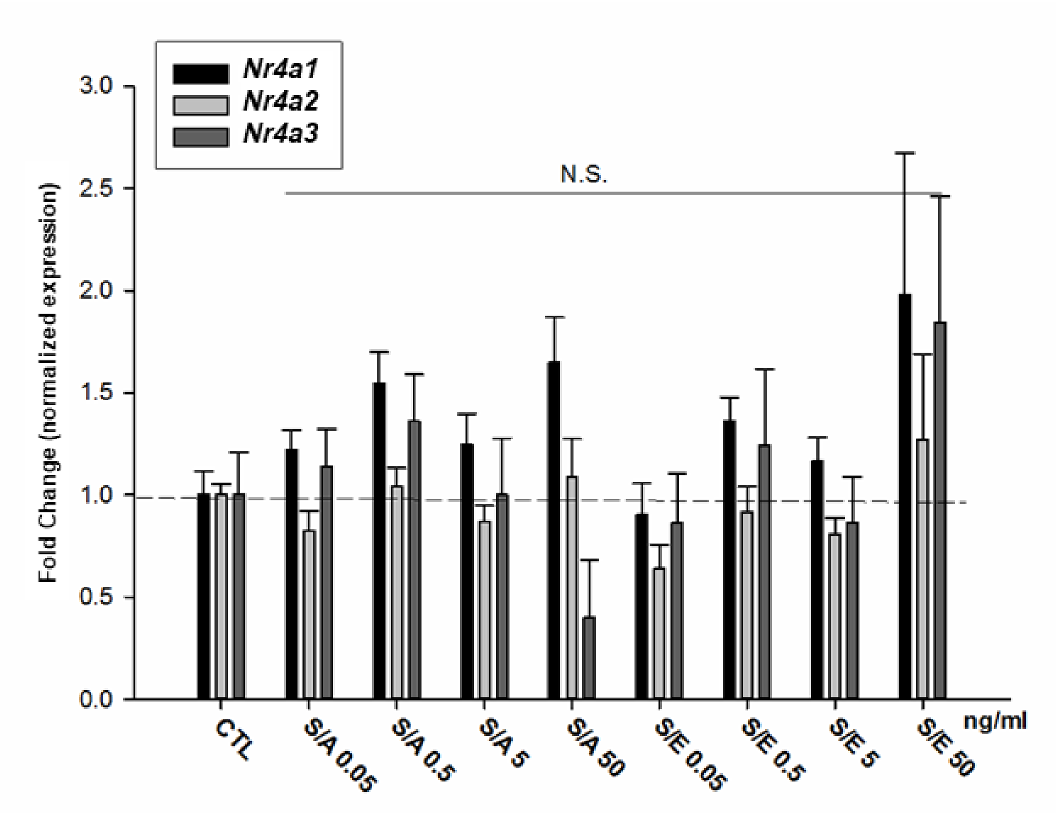

Supplementary Figure 7. Analysis of NR4A subfamily gene expression in HL-1 cells treated with MGF E-domain peptides for 1 hour in the DMEM media (n=6).

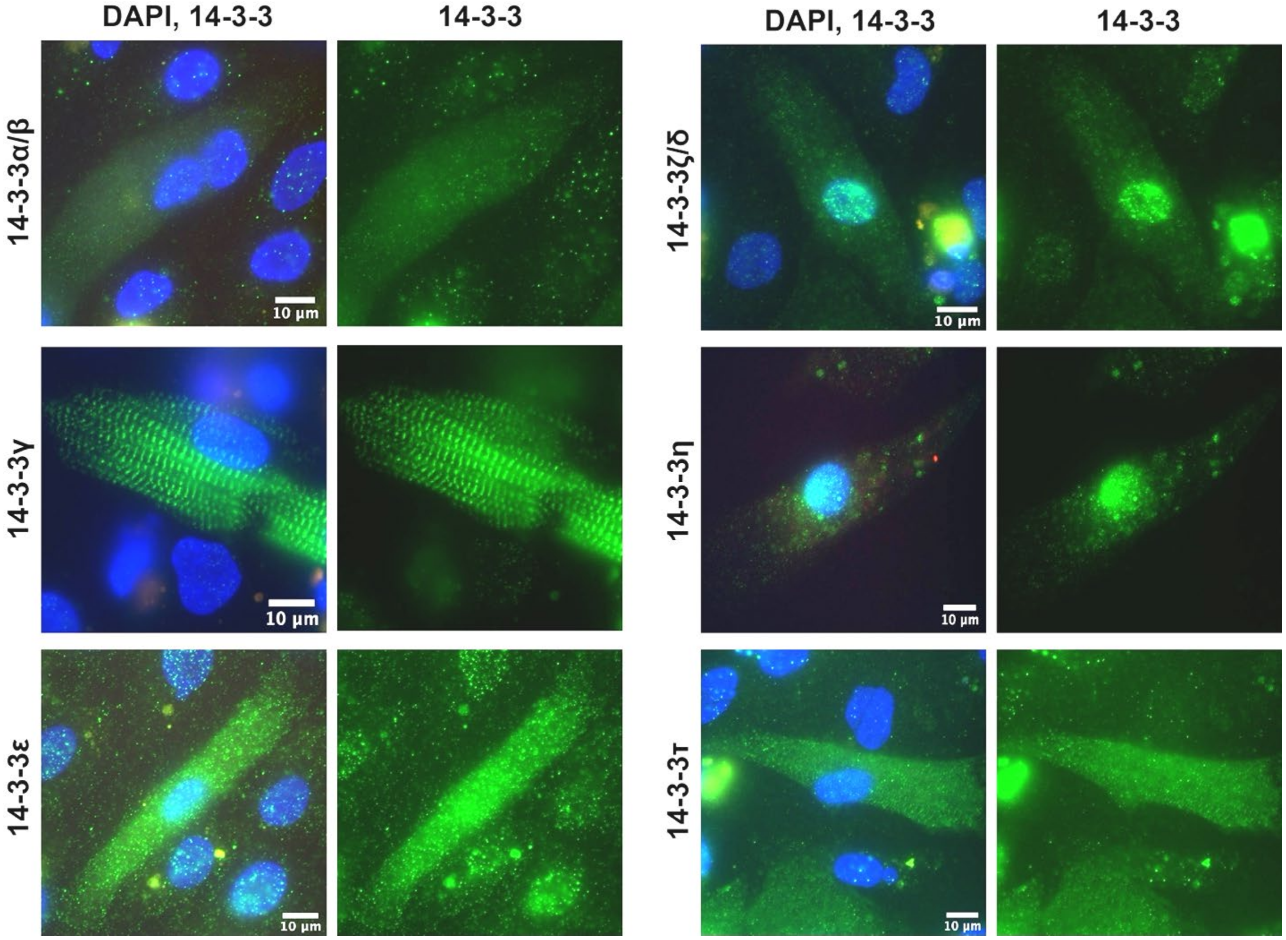

Supplementary Figure 8. Subcellular distribution of 14-3-3 isoforms in neonatal rat ventricular myocytes (NRVM). Image gallery depicts immunostains of NRVMs stained with DAPI (*blue*) and isoform specific 14-3-3α/β, 14-3-3γ, 14-3-3 ε, 14-3-3 ζ/δ, 14-3-3 η, and 14-3-3τ antibodies in descending order (*green*). Scale bar, 10 µm.

Supplementary Figure 9

**Name**	**Sequence**	**Uniprot ID**	**14-3-3 Pred score**
HSPB6(mouse)	RRA[S^{16}]AP<br>RAP[S^{59}]VA	Q5EBG6	1.133<br>0.585
ARRB2 (mouse)	PRQ[S^{361}]AP	Q91YI4	0.933
PRKD1(mouse)	RRL[S^{203}]NV<br>RSN[S^{255}]QS<br>RSS[S^{427}]TV	Q62101	0.870<br>0.833<br>0.619
MYPC3 (mouse)	RRT[S^{273}]LA<br>REL[S^{826}]HE<br>RTH[S^{1036}]GT<br>RVF[S^{1137}]HN	O70468	0.753<br>0.708<br>0.674<br>0.705
PLN (mouse)	RRA[S^{16}]TI	P61014	0.630
MGF (mouse)	RKG[S^{153}]TF	Q4VJB9	0.764
MGF (human)	RKG[S^{152}]TF	Q13429	0.475

Supplementary Figure 9. 14-3-3 Pred tool analysis of putative 14-3-3 binding sites in the proteins shown to interact with 14-3-3γ and the MGF E-domain. The phosphoserine sites identified in each protein were selected based on highest 14-3-3 prediction scores. Phosphothreonine sites are not included.

Supplementary Methods

Supplementary Table 1. Peptide sequences

PEPTIDE	SEQUENCE
Stabilized native E-domain	[1]YQPPSTNKNTKSQ(d-R)(d-R)KGSTFEERK[24]
Stabilized S/A[18]	[1]YQPPSTNKNTKSQ(d-R)(d-R)KGATFEERK[24]
Stabilized S/E[18]	[1]YQPPSTNKNTKSQ(d-R)(d-R)KGETFEERK[24]
Stabilized pSer[18]	[1]YQPPSTNKNTKSQ(d-R)(d-R)KGS(p)TFEERK[24]

Human MGF E-domain C-terminal 24-aa peptide analogs were synthesized and purified to >90% by HPLC (Lifetein Corp, NJ). All peptides were stabilized by amidating the C-terminus and switching the arginines[14,15] to the D-stereoisomer.

Supplementary Table 2. qPCR Primers

Gene	Forward primer	Reverse primer	GenBank accession number
Nr4a1	TCATCACTGATCGACACG	CTTCAGACAGCTAGCAATG	NM_010444
Nr4a2	CTATGGTCACAGAGAGACAC	CAACAGTTTAGACAGGTAGTTG	NM_001139509
Nr4a3	GAAAAGATCCCAGGATTCAC	GTTTGACCTGATGGAAAGTC	NM_015743
NPPA	TGGAGGAGAAGATGCCGGTA	CGAAGCAGCTGGATCTTCGTAG	NM_008725
Ephb1	CCTCCTCCTATGGACTGCCC	AAGGCCGTGAAGTCTGGGATA	NM_173447
Epb4.1l4a	CAGGTGAAGCAAGATGCCCTT	GTCGTGGTCTCCCAGTTCG	NM_013512
Ppie	CCCATGAAAAGGGCTTTGGC	TGGTGTTCGGGCCAGAATTG	NM_019489
18s	TGAGGCCATGATTAAGAGGG	AGTCGGCATCGTTTATGGTC	NR_003278

Supplementary Table 3: Protein-coding genes differentially-expressed (either induced (black type-face) or repressed (blue type-face)) in *both* S/A[18] and S/E[18] peptide-treated animals in a *concordant* manner relative to saline-treated controls. Only genes exhibiting a fold-change (FC) ≥ 1.2 and p value (P) ≤0.05 are considered. In cases of multiple probes mapping to the same transcript, the greatest FC is reported. Genes are ranked according to average fold-change.

		SA v Control		SE v Control		
**RefSeq/Ensembl ID**	**Gene Symbol**	**FC**	**P**	**FC**	**P**	**Mean FC**
NM_024228	Gdpd3	22.5	4E-07	1.6	3E-02	12.1
NR_024202	Rny3	10.2	6E-05	13.0	3E-05	11.6
NR_004419	Rny1	4.3	7E-03	8.4	9E-04	6.3
NR_028519	Scarna6	4.7	4E-04	6.2	1E-04	5.4
BC068263	Ptcd3	5.0	4E-06	4.8	4E-06	4.9
NM_025279	Hnrnpk	3.5	2E-02	4.8	7E-03	4.1
ENSMUST00000099431	Gm10132	3.4	5E-04	4.6	1E-04	4.0
ENSMUST00000129884	Ep400	3.3	4E-04	4.6	8E-05	3.9
NR_004412	Rnu1b1	3.7	4E-06	3.7	4E-06	3.7
NM_019769	1500003O03Rik	2.9	2E-03	4.2	4E-04	3.5
NR_002847	Malat1	2.9	5E-03	4.2	1E-03	3.5
NR_002842	Rnu3a	3.3	3E-04	3.6	2E-04	3.5
NR_001460	Rmrp	2.9	2E-04	4.0	4E-05	3.4
NR_046144	Rn5s20	2.8	5E-04	3.9	9E-05	3.3
ENSMUST00000111031	Cd209a	3.0	1E-05	3.6	4E-06	3.3
NR_004418	Rnu73b	2.7	3E-02	3.8	9E-03	3.2
NM_024212	Rpl4	3.1	8E-04	3.3	6E-04	3.2
NM_207162	LOC382133	3.1	2E-02	3.0	2E-02	3.1
XM_001473524	Gm2427	2.6	6E-03	3.5	1E-03	3.0
ENSMUST00000125969	Eif4a2	3.1	4E-05	3.0	5E-05	3.0
NM_201530	Sly	3.1	2E-02	3.0	3E-02	3.0
NM_008143	Gnb2l1	2.6	1E-02	3.4	3E-03	3.0
NR_004439	Rprl2	2.5	2E-04	3.5	3E-05	3.0
NM_022020	Rbp7	2.7	2E-03	3.3	6E-04	3.0
ENSMUST00000096020	Gm10335	2.5	7E-04	3.4	1E-04	3.0
NM_009609	Actg1	2.4	3E-02	3.5	6E-03	2.9
NR_002142	Rpph1	2.7	2E-05	3.1	1E-05	2.9
NM_001033865	Rps27a	2.8	2E-03	3.0	1E-03	2.9
BC006722	Hspa8	3.1	1E-03	2.6	3E-03	2.9
ENSMUST00000139730	Top2a	3.7	3E-05	2.0	2E-03	2.9
ENSMUST00000024846	Myl12a	2.6	1E-02	3.0	6E-03	2.8
NM_018853	Rplp1	3.0	5E-06	2.6	1E-05	2.8
NM_001177574	Gm2022	3.0	4E-03	2.6	7E-03	2.8
NM_023821	Cmya5	2.9	3E-05	2.7	4E-05	2.8
BC059277	Ppan	2.5	1E-02	2.8	5E-03	2.7
ENSMUST00000092880	Tlcd1	1.7	3E-03	3.5	2E-05	2.6
BC147042	Gm561	2.5	3E-04	2.7	2E-04	2.6
NM_025551	Ndufa12	2.5	5E-03	2.7	3E-03	2.6

XM_983891	Gm13035	2.8	1E-05	2.3	5E-05	2.5
XM_001476642	Gm9602	2.7	5E-04	2.3	1E-03	2.5
ENSMUST00000123995	Eif4a1	2.3	7E-04	2.7	2E-04	2.5
NM_001166750	Vmn1r143	2.5	2E-02	2.5	2E-02	2.5
BC115528	Vmn1r233	2.1	4E-03	2.8	6E-04	2.4
NM_001166759	Vmn1r107	2.3	5E-02	2.5	3E-02	2.4
BC110660	Taf1d	2.2	1E-02	2.6	7E-03	2.4
NM_001166848	Vmn1r130	2.4	3E-02	2.4	3E-02	2.4
XR_141763	LOC100862317	2.3	8E-03	2.5	5E-03	2.4
XM_003086913	Gm13149	2.5	6E-06	2.2	2E-05	2.4
NM_080575	Acss1	2.2	4E-02	2.5	2E-02	2.3
NM_010634	Fabp5	1.9	3E-02	2.8	4E-03	2.3
NM_009220	Ssty1	2.6	6E-03	2.0	3E-02	2.3
XM_001480161	Gm4184	2.0	2E-05	2.6	3E-06	2.3
NM_001122661	Speer4e	2.3	2E-02	2.2	3E-02	2.3
NR_028538	Scarna2	2.3	1E-04	2.2	2E-04	2.2
ENSMUST00000074177	Olfr530	1.7	3E-02	2.7	2E-03	2.2
NM_148949	Tdpoz1	1.9	3E-02	2.5	6E-03	2.2
NM_001166726	Vmn1r127	2.0	3E-02	2.4	1E-02	2.2
NM_008854	Prkaca	2.3	7E-03	2.1	1E-02	2.2
NM_019912	Ube2d2a	2.0	8E-03	2.3	3E-03	2.2
ENSMUST00000140659	Rpl5	2.3	4E-03	2.1	7E-03	2.2
NM_019749	Gabarap	1.9	2E-02	2.4	5E-03	2.2
ENSMUST00000160976	Nop56	2.0	3E-02	2.3	1E-02	2.1
NM_001199332	LOC100041550	2.3	2E-02	2.0	4E-02	2.1
NM_021338	Rpl35a	2.0	2E-04	2.3	4E-05	2.1
NR_028574	Snhg8	2.1	5E-05	2.1	4E-05	2.1
ENSMUST00000167743	Ube4b	1.8	6E-04	2.4	7E-05	2.1
NM_175341	Mbnl2	2.3	2E-03	1.9	9E-03	2.1
ENSMUST00000169749	Lpl	1.8	2E-02	2.4	3E-03	2.1
NR_028576	Scarna13	1.8	2E-03	2.4	1E-04	2.1
NM_010726	Phyh	2.1	4E-02	2.1	4E-02	2.1
NM_019443	Ndufa1	2.0	8E-04	2.1	6E-04	2.1
NR_027885	Vaultrc5	2.0	5E-03	2.1	3E-03	2.1
NM_001252521	Picalm	1.9	2E-02	2.2	8E-03	2.0
NM_025292	Synj2bp	2.1	1E-02	2.0	2E-02	2.0
NM_010119	Ehd1	1.7	3E-02	2.3	5E-03	2.0
NM_010706	Lgals4	2.5	6E-04	1.5	3E-02	2.0
NR_028517	Scarna10	2.2	1E-05	1.8	8E-05	2.0
NM_001122682	Vmn1r132	2.1	3E-02	2.0	5E-02	2.0
NM_026703	Ndufa8	2.0	4E-02	2.0	4E-02	2.0
NR_033123	4933409K07Rik	2.6	7E-05	1.4	1E-02	2.0
BC062127	Zfp781	1.9	9E-04	2.2	2E-04	2.0
NM_153567	Slain2	2.0	4E-02	2.0	3E-02	2.0
NM_019770	Tmed2	2.2	4E-03	1.8	2E-02	2.0
NM_146277	Olfr1412	1.9	7E-03	2.1	2E-03	2.0

NM_001166749	Vmn1r142	1.9	4E-02	2.0	3E-02	2.0
NM_008997	Rab11b	1.8	7E-03	2.1	2E-03	2.0
NR_040432	Gm15708	2.1	6E-03	1.8	2E-02	2.0
AK079109	9430008C03Rik	2.0	7E-05	2.0	7E-05	2.0
NM_025987	Ndufa6	1.9	4E-04	2.1	1E-04	2.0
ENSMUST00000027997	Rgs5	1.7	3E-02	2.2	5E-03	2.0
NM_146645	Olfr1158	2.0	2E-02	1.9	3E-02	2.0
NR_033727	Rps27	1.7	8E-03	2.2	1E-03	2.0
ENSMUST00000135417	Gm7429	1.8	2E-02	2.1	5E-03	2.0
AK139259	Ywhaz	2.1	1E-02	1.8	2E-02	2.0
NM_011743	Zfp106	2.0	1E-02	1.8	3E-02	1.9
NM_001114754	Gm6121	1.9	5E-02	2.0	4E-02	1.9
NM_010886	Ndufa4	1.9	1E-04	2.0	7E-05	1.9
NM_001166747	Vmn1r135	1.9	4E-02	1.9	4E-02	1.9
NM_009529	Gm4836	1.8	3E-02	2.0	1E-02	1.9
ENSMUST00000121900	Gm2012	1.8	3E-02	2.0	1E-02	1.9
NM_025317	Mrpl54	1.8	5E-03	2.0	3E-03	1.9
NM_016802	Rhoa	1.9	4E-02	1.9	4E-02	1.9
NR_028560	Scarna17	1.9	2E-03	1.9	2E-03	1.9
NM_001040669	Gm5169	1.8	4E-02	2.0	2E-02	1.9
NM_009456	Ube2l3	2.0	2E-04	1.7	1E-03	1.9
ENSMUST00000020022	Smpdl3a	1.7	2E-02	2.1	6E-03	1.9
NR_004432	Rnu12	1.6	2E-03	2.1	1E-04	1.9
ENSMUST00000080866	Gm5453	1.6	7E-03	2.1	6E-04	1.9
NM_011343	Sec61g	1.6	2E-04	2.2	6E-06	1.9
NM_010432	Hipk1	1.8	4E-04	1.9	3E-04	1.9
AK163470	Ell2	1.8	2E-04	2.0	8E-05	1.9
DQ237930	Klrb1-ps1	2.0	1E-02	1.7	5E-02	1.9
XM_486208	Gm5614	1.9	3E-04	1.9	3E-04	1.9
NM_197979	Uqcr10	1.8	2E-02	2.0	1E-02	1.9
ENSMUST00000085631	Gm7589	1.5	3E-02	2.2	2E-03	1.9
BC096589	Zfp526	1.6	5E-02	2.1	7E-03	1.8
ENSMUST00000046515	Nceh1	1.9	9E-03	1.8	1E-02	1.8
NR_033388	Gm3002	1.9	1E-03	1.8	1E-03	1.8
NM_207523	Rpl23a	1.7	1E-04	1.9	4E-05	1.8
X14625	Igkv5-43	1.8	4E-02	1.8	4E-02	1.8
NM_025424	Nenf	2.1	2E-04	1.6	4E-03	1.8
ENSMUST00000091931	Gm10166	1.6	2E-04	2.0	2E-05	1.8
NM_027342	Fam162a	2.0	7E-04	1.6	6E-03	1.8
NM_001167578	Tcp10c	1.8	4E-02	1.8	4E-02	1.8
ENSMUST00000058437	Rpl7	1.6	3E-02	2.0	5E-03	1.8
NM_024173	Atp6v1g1	1.7	2E-02	1.9	7E-03	1.8
NM_011295	Rps12	1.7	6E-04	1.9	1E-04	1.8
NM_177192	Dennd5b	1.9	1E-03	1.7	4E-03	1.8
NM_001013786	Zfp187	1.7	4E-03	1.8	2E-03	1.8
NM_001166637	Gm4301	1.8	4E-03	1.8	4E-03	1.8

NM_027360	2010107E04Rik	1.6	3E-02	2.0	5E-03	1.8
XM_483949	Gm5428	1.7	1E-03	1.9	4E-04	1.8
NM_008249	Tfb2m	1.6	2E-02	1.9	5E-03	1.8
XM_003086019	LOC100504755	1.8	2E-02	1.7	4E-02	1.8
NM_001177535	Gm4340	1.7	6E-03	1.8	4E-03	1.8
NM_007710	Ckm	1.7	5E-02	1.8	2E-02	1.8
NM_175000	Hbq1a	1.9	6E-03	1.6	2E-02	1.8
NM_001177408	Gm15319	1.8	5E-03	1.7	1E-02	1.8
BC061237	BC061237	1.8	2E-02	1.7	2E-02	1.8
ENSMUST00000022826	Fitm1	1.7	7E-03	1.7	7E-03	1.7
NM_009169	Shfm1	1.5	1E-02	2.0	1E-03	1.7
NR_028042	Gm10012	1.6	6E-03	1.9	1E-03	1.7
ENSMUST00000154227	4921517L17Rik	1.6	2E-05	1.9	4E-06	1.7
BC147049	Fam131c	2.0	4E-04	1.4	2E-02	1.7
NM_178059	Etl4	1.5	8E-03	2.0	4E-04	1.7
BC013508	9130017N09Rik	1.6	3E-02	1.9	7E-03	1.7
XR_141965	Gm13815	1.4	5E-03	2.0	1E-04	1.7
XM_001479836	Gm9564	1.5	1E-02	2.0	5E-04	1.7
ENSMUST00000012849	Retn	1.8	1E-03	1.7	2E-03	1.7
NM_001039368	Polr2k	1.6	1E-03	1.9	2E-04	1.7
XM_915186	Gm13777	1.6	7E-04	1.8	2E-04	1.7
BC094068	Jund	2.0	2E-03	1.4	4E-02	1.7
NM_016738	Rpl13	1.7	7E-04	1.7	1E-03	1.7
NM_133834	Hnrnpf	1.6	2E-03	1.8	5E-04	1.7
NM_010106	Eef1a1	1.6	3E-03	1.8	8E-04	1.7
NM_025366	Chchd1	1.6	7E-03	1.8	3E-03	1.7
XM_001477032	Gm3453	1.8	3E-03	1.6	1E-02	1.7
NM_030694	Ifitm2	1.6	1E-02	1.8	6E-03	1.7
XM_885137	Gm6177	1.7	5E-02	1.7	4E-02	1.7
NM_027945	Csl	1.6	3E-02	1.8	8E-03	1.7
NM_018730	Rpl36	1.8	6E-05	1.7	1E-04	1.7
ENSMUST00000031670	Gng11	1.5	1E-02	1.9	7E-04	1.7
ENSMUST00000150618	Chchd7	1.9	4E-04	1.5	1E-02	1.7
ENSMUST00000094065	1810027O10Rik	1.6	2E-03	1.8	4E-04	1.7
ENSMUST00000105412	Col6a1	1.6	2E-03	1.8	4E-04	1.7
NM_029288	Gm10413	2.0	2E-04	1.4	1E-02	1.7
NM_177361	Ifna12	1.5	1E-02	1.8	3E-03	1.7
NM_001168334	Gm2799	1.5	6E-03	1.9	6E-04	1.7
NM_018871	Ywhag	1.7	2E-02	1.6	2E-02	1.7
NM_011288	Mrpl23	1.7	1E-03	1.7	7E-04	1.7
NM_008722	Npm1	1.5	4E-03	1.8	5E-04	1.7
NM_152810	Cdc5l	1.7	8E-03	1.7	9E-03	1.7
ENSMUST00000105087	Gm3940	1.5	2E-02	1.9	2E-03	1.7
NM_207635	Rps24	1.5	6E-03	1.8	1E-03	1.7
ENSMUST00000089635	Gm10029	1.8	1E-02	1.6	4E-02	1.7
NM_001191032	Cypt14	1.7	3E-03	1.7	3E-03	1.7

M94350	Iglv1	1.7	1E-02	1.6	2E-02	1.7
NR_004415	Rnu3b1	1.5	4E-02	1.8	6E-03	1.7
XR_140979	Gm16210	1.5	2E-02	1.8	4E-03	1.7
NM_009964	Cryab	1.6	2E-02	1.7	2E-02	1.7
NM_011507	Suclg2	1.6	8E-03	1.7	3E-03	1.7
XR_104802	Gm17608	1.7	2E-02	1.6	5E-02	1.6
NR_015545	4930481A15Rik	1.5	6E-03	1.8	1E-03	1.6
NM_026068	Med31	1.7	1E-04	1.6	2E-04	1.6
BC147528	1700018B08Rik	1.4	2E-02	1.8	2E-03	1.6
NM_001024706	Gm5458	1.8	5E-03	1.5	3E-02	1.6
NM_134042	Aldh6a1	1.6	2E-02	1.7	1E-02	1.6
XM_195264	Gm5058	1.6	2E-02	1.7	1E-02	1.6
ENSMUST00000166658	Pfdn5	1.6	4E-04	1.7	2E-04	1.6
NM_001039553	4930467E23Rik	1.6	6E-03	1.6	6E-03	1.6
NM_009941	Cox4i1	1.7	1E-04	1.5	7E-04	1.6
NM_001024618	Xirp2	1.6	4E-02	1.6	4E-02	1.6
NR_027059	2810008D09Rik	1.6	9E-03	1.7	3E-03	1.6
ENSMUST00000075549	Gm5451	1.6	3E-03	1.7	1E-03	1.6
ENSMUST00000169853	Msi2	1.8	4E-03	1.4	4E-02	1.6
ENSMUST00000032918	2900092E17Rik	1.7	2E-04	1.6	5E-04	1.6
ENSMUST00000080545	Rpl9	1.5	4E-03	1.7	1E-03	1.6
ENSMUST00000113154	Tmsb15a	1.7	3E-03	1.5	9E-03	1.6
ENSMUST00000095128	Ndufb6	1.6	4E-03	1.6	4E-03	1.6
NM_025983	Atp5e	1.6	3E-04	1.6	1E-04	1.6
XM_003086469	Rps15a-ps5	1.4	2E-02	1.8	2E-03	1.6
NM_010261	Rabac1	1.6	4E-04	1.6	3E-04	1.6
ENSMUST00000021632	Akr1c12	1.7	1E-02	1.5	3E-02	1.6
NM_011975	Rpl27a	1.4	3E-02	1.8	3E-03	1.6
AK015919	4930527J03Rik	1.7	5E-03	1.5	2E-02	1.6
NM_024439	H47	1.6	3E-03	1.6	4E-03	1.6
XM_003086803	LOC100040196	1.3	4E-02	1.9	3E-04	1.6
NM_010787	Mea1	1.4	1E-02	1.8	6E-04	1.6
NM_009296	Supt4h1	1.6	3E-03	1.6	2E-03	1.6
NM_024266	Rps25	1.6	2E-02	1.6	1E-02	1.6
NM_010664	Krt18	1.6	5E-02	1.6	4E-02	1.6
NM_001081220	Gpr179	1.4	2E-02	1.8	2E-03	1.6
XR_107580	Gm20021	1.6	1E-03	1.6	1E-03	1.6
ENSMUST00000080861	Rpl35	1.5	7E-03	1.7	1E-03	1.6
NM_026744	Mrpl53	1.6	1E-03	1.6	2E-03	1.6
XM_911155	Gm13637	1.5	6E-05	1.7	1E-05	1.6
AK031098	Gm9968	1.4	2E-02	1.7	3E-03	1.6
NM_001166708	Vmn1r119	1.5	3E-02	1.7	1E-02	1.6
ENSMUST00000114881	Rps10	1.4	2E-02	1.7	2E-03	1.6
BC094602	Tnks	1.6	2E-03	1.6	3E-03	1.6
ENSMUST00000160141	Sssca1	1.5	3E-02	1.7	9E-03	1.6
NM_016794	Vamp8	1.6	6E-04	1.5	1E-03	1.6

NM_001081273	1600015I10Rik	1.5	7E-03	1.7	1E-03	1.6
NM_025384	Dnajc15	1.7	2E-03	1.5	1E-02	1.6
NM_001081351	A430107O13Rik	1.6	3E-02	1.6	3E-02	1.6
NR_045050	9130015A21Rik	1.5	2E-02	1.7	7E-03	1.6
NM_009084	Rpl37a	1.5	5E-04	1.6	2E-04	1.6
NR_033515	Cyp2d37-ps	1.6	1E-02	1.6	1E-02	1.6
NR_040503	8430437L04Rik	1.6	1E-03	1.5	4E-03	1.6
NM_146781	Olfr922	1.4	3E-02	1.7	2E-03	1.6
NR_003630	Gm6498	1.6	1E-02	1.5	2E-02	1.6
NM_001256522	C330006A16Rik	1.6	6E-03	1.5	9E-03	1.6
NM_013795	Atp5l	1.5	1E-02	1.7	3E-03	1.6
AK135827	BC005512	1.4	2E-02	1.7	2E-03	1.6
NM_025440	Mrps16	1.5	1E-03	1.7	2E-04	1.6
ENSMUST00000091066	Gm5848	1.7	1E-02	1.5	4E-02	1.6
NM_001190356	Gm4832	1.4	4E-02	1.7	5E-03	1.6
NM_177171	Heatr5a	1.6	9E-03	1.5	1E-02	1.6
NM_011034	Prdx1	1.4	9E-04	1.7	4E-05	1.6
NM_001252218	Rpl31	1.5	9E-03	1.6	2E-03	1.6
NR_037961	1600020E01Rik	1.6	4E-03	1.5	6E-03	1.6
ENSMUST00000140679	Uba52	1.5	4E-05	1.6	9E-06	1.5
NM_013908	Fbxw5	1.6	1E-02	1.5	4E-02	1.5
NM_001114383	Luzp4	1.6	1E-02	1.5	3E-02	1.5
ENSMUST00000030533	Vwa5b1	1.4	4E-02	1.7	6E-03	1.5
NM_030207	Sfi1	1.6	1E-02	1.5	2E-02	1.5
NM_020498	Ly6i	1.6	6E-03	1.4	2E-02	1.5
BC004786	Igh-VJ558	1.5	6E-03	1.6	1E-03	1.5
XR_106239	Gm20095	1.4	3E-02	1.7	3E-03	1.5
ENSMUST00000160822	A130010J15Rik	1.4	2E-03	1.7	2E-04	1.5
ENSMUST00000129267	Lass2	1.6	8E-04	1.5	2E-03	1.5
ENSMUST00000072789	Gm10063	1.6	4E-03	1.5	2E-02	1.5
NM_001110229	Celf2	1.4	5E-03	1.6	1E-03	1.5
NR_040755	4930568E12Rik	1.5	3E-02	1.6	2E-02	1.5
XM_001002269	Gm8290	1.5	3E-02	1.6	2E-02	1.5
ENSMUST00000036996	Ndufb7	1.5	2E-03	1.6	5E-04	1.5
ENSMUST00000044043	LOC100046079	1.5	1E-03	1.6	8E-04	1.5
ENSMUST00000144720	Med24	1.6	3E-02	1.5	5E-02	1.5
NM_007507	Atp5k	1.5	2E-02	1.5	2E-02	1.5
NM_175393	4930555G01Rik	1.4	4E-02	1.6	9E-03	1.5
NM_026369	Arpc5	1.5	1E-02	1.5	8E-03	1.5
NM_001005520	Olfr231	1.5	7E-03	1.5	6E-03	1.5
BC024574	1110012L19Rik	1.4	1E-03	1.6	1E-04	1.5
NM_198167	Tmem63b	1.5	5E-03	1.5	8E-03	1.5
NM_008448	Kif5b	1.5	1E-03	1.5	8E-04	1.5
NM_001166634	Gm4302	1.6	3E-03	1.5	8E-03	1.5
NM_001013816	Gm5622	1.4	1E-02	1.6	4E-03	1.5
NR_045739	D330022K07Rik	1.6	9E-03	1.4	3E-02	1.5

NM_025587	Rps21	1.5	3E-03	1.6	1E-03	1.5
NM_007564	Zfp36l1	1.4	3E-02	1.6	1E-02	1.5
NR_045363	1700024F13Rik	1.5	4E-03	1.5	5E-03	1.5
NM_001082974	Neurl2	1.7	1E-03	1.3	2E-02	1.5
AK020613	9530057J20Rik	1.4	5E-02	1.6	2E-02	1.5
NM_001159483	Rpl19	1.5	6E-05	1.5	3E-05	1.5
NR_037569	1810026B05Rik	1.5	1E-02	1.5	2E-02	1.5
NM_026624	Fam166a	1.4	4E-02	1.6	2E-02	1.5
XM_001474034	Gm8239	1.3	2E-02	1.7	1E-03	1.5
NM_001141921	Lrfn1	1.6	4E-03	1.4	3E-02	1.5
NM_029911	Kcnk10	1.4	3E-03	1.5	1E-03	1.5
XR_141809	Gm19270	1.4	2E-02	1.6	3E-03	1.5
NM_001145038	Gm2933	1.6	1E-02	1.4	3E-02	1.5
NM_147072	Olfr641	1.5	3E-02	1.5	2E-02	1.5
NM_001162933	Rpl10l	1.4	4E-02	1.6	1E-02	1.5
XR_141970	Gm20325	1.3	1E-02	1.7	3E-04	1.5
NM_009093	Rps29	1.4	6E-04	1.6	1E-04	1.5
NM_145587	Sbk1	1.7	3E-05	1.3	2E-03	1.5
NM_177715	Kctd12	1.5	3E-02	1.5	2E-02	1.5
ENSMUST00000108430	Rps19	1.4	4E-03	1.5	1E-03	1.5
ENSMUST00000052832	2410015M20Rik	1.4	2E-02	1.5	9E-03	1.5
NM_011029	Rpsa	1.5	4E-03	1.5	5E-03	1.5
NM_013536	Emg1	1.4	3E-03	1.5	6E-04	1.5
NM_134185	Vmn1r9	1.5	4E-03	1.5	3E-03	1.5
NM_001163713	Tufm	1.4	6E-03	1.5	2E-03	1.5
ENSMUST00000020717	Arf5	1.5	6E-03	1.4	1E-02	1.5
NM_001081662	C86695	1.5	3E-02	1.5	3E-02	1.5
NM_001029930	Gm5796	1.6	3E-03	1.4	2E-02	1.5
ENSMUST00000030348	Magoh	1.4	3E-02	1.5	3E-02	1.5
NM_019865	Rpl36a	1.5	6E-04	1.4	2E-03	1.5
NM_007748	Cox6a1	1.5	1E-04	1.5	1E-04	1.5
NM_007475	Rplp0	1.3	4E-02	1.7	1E-03	1.5
NM_008683	Nedd8	1.5	9E-03	1.4	1E-02	1.5
NR_045895	Gm13546	1.5	2E-03	1.4	4E-03	1.5
NM_133188	Dazap1	1.5	9E-05	1.4	4E-04	1.5
NM_001033142	Rnf166	1.6	4E-06	1.3	1E-04	1.5
NM_026533	Rps13	1.4	2E-02	1.5	1E-02	1.5
NM_175163	Zfp689	1.3	1E-02	1.6	3E-04	1.5
NM_153071	Gprc6a	1.5	9E-03	1.4	3E-02	1.5
ENSMUST00000030124	Tal2	1.5	5E-04	1.4	1E-03	1.5
NM_026014	Cdt1	1.5	2E-02	1.4	3E-02	1.5
NM_144942	Csad	1.4	8E-03	1.5	2E-03	1.5
NM_026794	Deb1	1.5	4E-03	1.4	6E-03	1.5
NM_145144	Aif1l	1.5	5E-03	1.4	2E-02	1.5
NM_001081312	Tmco2	1.3	3E-02	1.6	7E-04	1.5
NM_009775	Tspo	1.5	2E-04	1.4	1E-03	1.5

NM_025348	Ndufa3	1.4	1E-02	1.5	3E-03	1.4
NM_172511	Abhd10	1.5	1E-02	1.4	1E-02	1.4
NM_023684	Lime1	1.4	2E-02	1.5	2E-02	1.4
NM_010058	Dmwd	1.5	9E-04	1.4	1E-03	1.4
NM_021447	Trim54	1.4	9E-04	1.5	4E-04	1.4
NM_133711	Spata4	1.4	3E-03	1.5	2E-03	1.4
ENSMUST00000036088	Gngt2	1.3	1E-02	1.6	4E-04	1.4
J02622	Got2	1.5	4E-02	1.4	5E-02	1.4
NM_177213	Abca15	1.4	2E-02	1.4	2E-02	1.4
NM_001163028	Bcmo1	1.5	3E-02	1.4	4E-02	1.4
NM_001161338	Sh3bp5l	1.4	3E-02	1.5	1E-02	1.4
ENSMUST00000161023	1810035L17Rik	1.4	3E-02	1.5	2E-02	1.4
NM_133731	Prss22	1.5	4E-03	1.3	2E-02	1.4
XR_141767	Gm20555	1.4	6E-04	1.5	3E-04	1.4
NM_177723	Vsig8	1.5	4E-02	1.4	5E-02	1.4
NM_027244	Ndufa11	1.4	4E-02	1.5	1E-02	1.4
NM_019950	Chst5	1.4	3E-02	1.4	3E-02	1.4
AK138535	Bmpr2	1.4	3E-02	1.5	3E-02	1.4
NM_016749	Mybph	1.4	2E-02	1.5	6E-03	1.4
ENSMUST00000135884	Nme1	1.4	8E-03	1.5	4E-03	1.4
NM_001040461	Dear1	1.4	4E-02	1.4	4E-02	1.4
NR_045332	E330023G01	1.5	7E-03	1.4	1E-02	1.4
NM_013748	Clnk	1.4	3E-02	1.4	4E-02	1.4
NR_045299	2900076A07Rik	1.4	4E-02	1.5	2E-02	1.4
XR_142256	E330027M22Rik	1.3	4E-02	1.5	7E-03	1.4
ENSMUST00000014080	Myl2	1.5	1E-03	1.4	4E-03	1.4
NM_008944	Psma2	1.4	1E-02	1.5	6E-03	1.4
NM_009347	Tecta	1.5	2E-03	1.3	1E-02	1.4
NM_001160239	Fau	1.4	3E-03	1.5	6E-04	1.4
NM_009883	Cebpb	1.4	3E-02	1.4	2E-02	1.4
BC060261	Ppp1r3b	1.5	2E-02	1.4	5E-02	1.4
NM_009889	Cga	1.3	4E-02	1.6	2E-03	1.4
NM_001004365	Actr3b	1.5	1E-03	1.3	1E-02	1.4
ENSMUST00000106357	Ypel3	1.5	1E-02	1.4	2E-02	1.4
NM_019880	Mtch1	1.4	1E-04	1.4	2E-04	1.4
NR_028021	Ppifos	1.6	5E-04	1.3	1E-02	1.4
NM_001085500	Cisd3	1.4	2E-02	1.4	2E-02	1.4
XR_142086	Gm12739	1.3	3E-02	1.5	8E-03	1.4
NR_040580	1700094J05Rik	1.4	3E-02	1.4	3E-02	1.4
ENSMUST00000107237	Psmd4	1.4	1E-03	1.4	3E-03	1.4
NM_177250	Lingo4	1.3	2E-02	1.5	4E-03	1.4
NM_026468	Atp5g2	1.4	5E-05	1.4	3E-05	1.4
ENSMUST00000041366	Polr2j	1.3	1E-02	1.5	1E-03	1.4
NM_007886	Dtnb	1.4	2E-02	1.4	2E-02	1.4
BC145053	A530021J07Rik	1.3	2E-02	1.5	2E-03	1.4
NM_080455	Tshz2	1.5	5E-03	1.3	2E-02	1.4

ENSMUST00000033683	Rps4x	1.3	3E-04	1.5	2E-05	1.4
NM_025559	1810046J19Rik	1.3	2E-02	1.5	3E-03	1.4
NM_011289	Rpl27	1.4	4E-03	1.4	2E-03	1.4
NM_009470	Umod	1.4	1E-02	1.4	1E-02	1.4
NM_028809	Arpc5l	1.5	2E-05	1.3	9E-05	1.4
NM_138601	D10Jhu81e	1.5	4E-03	1.3	2E-02	1.4
NM_146305	Olfr420	1.5	3E-03	1.3	2E-02	1.4
NM_010689	Lat	1.4	5E-02	1.4	3E-02	1.4
NM_133236	Glcci1	1.4	3E-03	1.4	6E-03	1.4
NM_028157	Fam75d3	1.4	5E-02	1.4	3E-02	1.4
ENSMUST00000040372	1810006K21Rik	1.4	3E-02	1.4	2E-02	1.4
NM_001110148	Mgat1	1.5	2E-03	1.3	4E-02	1.4
NM_028659	Eif3k	1.4	9E-03	1.4	1E-02	1.4
NR_037679	5330439B14Rik	1.4	2E-02	1.3	4E-02	1.4
NM_025583	Ctrb1	1.4	4E-02	1.4	3E-02	1.4
NR_045476	1700007J10Rik	1.4	3E-02	1.4	3E-02	1.4
ENSMUST00000105454	Col13a1	1.4	2E-02	1.4	2E-02	1.4
NM_147070	Olfr603	1.3	6E-03	1.5	8E-04	1.4
NM_001166206	Erv3	1.3	2E-02	1.5	2E-03	1.4
NM_172457	Mob3a	1.4	2E-02	1.4	1E-02	1.4
NM_025790	Acot13	1.3	3E-02	1.5	2E-03	1.4
NR_045952	Gm4719	1.2	2E-02	1.6	4E-04	1.4
NM_011307	Uimc1	1.4	5E-04	1.4	5E-04	1.4
ENSMUST00000127405	Nhp2	1.3	2E-02	1.5	4E-03	1.4
NR_028367	Nip7	1.4	9E-03	1.4	7E-03	1.4
NM_008811	Pdha2	1.3	2E-02	1.4	9E-03	1.4
NM_001029890	Mex3a	1.4	2E-03	1.3	8E-03	1.4
BC096614	6030419C18Rik	1.5	2E-03	1.3	2E-02	1.4
NR_033444	Krt74	1.4	7E-03	1.4	4E-03	1.4
NM_018772	Bri3	1.5	9E-05	1.3	3E-03	1.4
NM_010599	Kcnab3	1.5	1E-04	1.2	1E-02	1.4
NM_133664	Lad1	1.4	3E-04	1.4	3E-04	1.4
NM_028923	Gle1	1.5	4E-04	1.3	8E-03	1.4
NR_045794	Gm16907	1.4	4E-02	1.4	4E-02	1.4
NM_028146	Dbndd1	1.3	4E-03	1.4	2E-03	1.4
ENSMUST00000003100	Cyp2f2	1.3	4E-02	1.4	2E-02	1.4
NM_013848	Ermap	1.4	2E-02	1.4	2E-02	1.4
ENSMUST00000085894	Ccdc19	1.3	1E-02	1.4	8E-03	1.4
ENSMUST00000033755	Asb11	1.3	2E-03	1.4	4E-04	1.4
ENSMUST00000005923	Psmb4	1.4	1E-02	1.3	5E-02	1.4
ENSMUST00000141116	Taf10	1.3	4E-04	1.4	1E-04	1.4
NM_001165957	Nme9	1.3	4E-02	1.4	2E-02	1.4
NM_007531	Phb2	1.4	3E-04	1.3	1E-03	1.4
NR_027352	Vash2	1.5	3E-03	1.3	2E-02	1.4
NM_026636	5430437P03Rik	1.4	1E-03	1.3	7E-03	1.4
ENSMUST00000000910	Dbh	1.3	5E-02	1.4	2E-02	1.4

NM_008206	H2-Oa	1.4	6E-03	1.4	7E-03	1.4
NM_022656	Nisch	1.4	7E-03	1.3	2E-02	1.4
NM_001081044	Mylk2	1.3	4E-02	1.4	1E-02	1.4
NM_182745	1700028K03Rik	1.3	8E-03	1.4	3E-03	1.4
ENSMUST00000081679	Gm6404	1.3	2E-02	1.4	3E-03	1.4
XM_893121	Rpl31-ps20	1.3	2E-02	1.4	1E-02	1.4
NM_021299	Ak3	1.3	3E-02	1.4	2E-02	1.4
NM_008251	Hmgn1	1.4	4E-03	1.3	3E-02	1.4
NM_139139	Dnajc17	1.3	1E-02	1.4	9E-03	1.4
ENSMUST00000028005	Mgst3	1.3	1E-02	1.4	9E-03	1.4
NR_045287	1700097N02Rik	1.4	7E-03	1.3	4E-02	1.4
ENSMUST00000029698	Lamtor2	1.4	5E-03	1.3	2E-02	1.4
NM_026972	Cd209b	1.4	1E-02	1.3	4E-02	1.4
AK154763	Gm10624	1.2	3E-02	1.5	2E-03	1.4
NR_029731	Mirlet7f-1	1.4	2E-02	1.3	3E-02	1.4
XR_106926	LOC100505027	1.2	4E-02	1.5	2E-03	1.4
NM_008248	Hint1	1.4	3E-05	1.3	2E-04	1.4
NM_027066	Tmem89	1.4	1E-02	1.3	4E-02	1.4
NM_011354	Serf2	1.4	2E-05	1.3	7E-05	1.4
NM_013648	Rtn2	1.3	2E-02	1.4	9E-03	1.4
NM_053251	Gm4736	1.3	4E-02	1.4	3E-02	1.4
NM_178594	Vtcn1	1.4	2E-02	1.3	5E-02	1.4
ENSMUST00000113749	Plcd4	1.4	7E-03	1.3	3E-02	1.4
NM_172895	Fam71e2	1.3	4E-02	1.4	2E-02	1.3
NM_013874	Dpf1	1.4	1E-02	1.3	2E-02	1.3
NM_026506	Snrpg	1.3	9E-03	1.4	5E-03	1.3
NM_028281	Pcbd2	1.3	4E-02	1.4	2E-02	1.3
NM_026042	Med29	1.3	4E-02	1.4	1E-02	1.3
NM_008705	Nme2	1.3	4E-04	1.4	1E-04	1.3
NM_019675	Stmn4	1.3	3E-03	1.3	3E-03	1.3
ENSMUST00000109913	Nr3c2	1.3	5E-02	1.4	2E-02	1.3
NM_021505	Anapc5	1.4	1E-03	1.3	2E-03	1.3
NM_001166480	Ssr4	1.3	2E-02	1.4	2E-03	1.3
NM_001164729	Tomm6	1.3	5E-04	1.3	5E-04	1.3
AK020767	A430105J06Rik	1.4	2E-02	1.3	3E-02	1.3
ENSMUST00000045602	Ndufb10	1.4	1E-02	1.3	1E-02	1.3
NM_028758	Gga2	1.4	2E-02	1.3	4E-02	1.3
NM_013523	Fshr	1.3	3E-02	1.4	2E-02	1.3
NR_037973	Gm20597	1.2	4E-03	1.4	1E-04	1.3
NM_009785	Cacna2d3	1.4	3E-02	1.3	4E-02	1.3
NM_025628	Cox6b1	1.3	1E-02	1.4	1E-03	1.3
NM_009113	S100a13	1.3	4E-03	1.4	2E-03	1.3
NM_027306	Zdhhc25	1.2	3E-02	1.4	2E-03	1.3
ENSMUST00000024697	Hcfc1r1	1.4	1E-02	1.3	2E-02	1.3
NM_001081069	Rgs11	1.4	1E-02	1.3	2E-02	1.3
NM_011886	Scamp3	1.3	2E-02	1.4	3E-03	1.3

NR_038151	2410004N09Rik	1.4	4E-03	1.2	4E-02	1.3
ENSMUST00000021412	Psma6	1.3	7E-03	1.3	5E-03	1.3
NM_194344	Sh3tc1	1.3	2E-02	1.4	1E-02	1.3
NM_178750	Ss18l1	1.4	2E-02	1.3	5E-02	1.3
NM_008162	Gpx4	1.3	3E-02	1.3	3E-02	1.3
NM_010795	Mgat3	1.4	1E-02	1.3	3E-02	1.3
NM_201531	Kcnf1	1.4	2E-02	1.3	4E-02	1.3
NM_025324	Zfp524	1.3	6E-04	1.4	1E-04	1.3
NM_001004147	Fbll1	1.3	3E-02	1.3	3E-02	1.3
NM_177200	Svopl	1.3	2E-02	1.4	4E-03	1.3
NR_037975	Gm5105	1.4	5E-03	1.3	2E-02	1.3
NM_001200055	AU018829	1.4	5E-03	1.3	2E-02	1.3
BC132142	4921504E06Rik	1.3	2E-02	1.3	1E-02	1.3
NM_145579	Mypop	1.4	2E-03	1.3	1E-02	1.3
NM_026526	N6amt2	1.3	5E-02	1.3	4E-02	1.3
AK019842	4930595D18Rik	1.2	2E-02	1.4	3E-03	1.3
NM_013541	Gstp1	1.4	2E-03	1.3	4E-03	1.3
NM_145495	Rin1	1.3	6E-04	1.4	1E-04	1.3
BC100531	4832428D23Rik	1.3	4E-02	1.4	2E-02	1.3
NM_026332	Dnajc19	1.3	3E-02	1.3	2E-02	1.3
NM_010798	Mif	1.4	7E-03	1.3	2E-02	1.3
NM_148952	E2f4	1.3	2E-05	1.3	5E-05	1.3
NM_001077696	Hdac5	1.4	3E-03	1.2	4E-02	1.3
NM_053249	Krt82	1.3	2E-02	1.3	3E-02	1.3
NM_016872	Vamp5	1.4	5E-05	1.2	2E-03	1.3
XM_003085851	Gm270	1.4	4E-03	1.3	2E-02	1.3
NR_033301	Gm5627	1.3	1E-02	1.3	2E-02	1.3
NM_008734	Nrg3	1.3	1E-02	1.3	2E-02	1.3
NM_001111066	Fkbp8	1.3	2E-03	1.3	5E-03	1.3
NM_011944	Map2k7	1.3	1E-03	1.4	3E-04	1.3
NM_011149	Ppib	1.2	7E-03	1.4	4E-04	1.3
XM_003085238	Gm19956	1.3	4E-02	1.3	5E-02	1.3
NM_053085	Tcf23	1.4	6E-03	1.3	3E-02	1.3
NM_207545	Vmn1r179	1.3	2E-02	1.3	3E-02	1.3
NM_177613	Cdc34	1.3	3E-02	1.4	9E-03	1.3
NM_001025608	D4Ertd22e	1.4	7E-03	1.2	3E-02	1.3
AK145005	2010300F17Rik	1.2	1E-02	1.4	3E-03	1.3
NM_172416	Ostm1	1.3	2E-02	1.3	3E-02	1.3
NM_145421	Fam108a	1.3	2E-02	1.3	8E-03	1.3
NM_023323	Rpf2	1.2	1E-02	1.4	8E-04	1.3
NM_018758	Apba3	1.4	1E-03	1.2	8E-03	1.3
NR_044987	Gm5441	1.2	8E-03	1.4	1E-03	1.3
NM_029775	Dcun1d5	1.3	8E-03	1.3	6E-03	1.3
NM_008045	Fshb	1.2	3E-02	1.4	4E-03	1.3
BC147515	Gm906	1.3	1E-02	1.4	3E-03	1.3
NM_011319	Sars	1.2	3E-02	1.4	2E-03	1.3

NM_001145096	Hhla1	1.3	3E-02	1.3	3E-02	1.3
NM_029555	Gstk1	1.3	2E-02	1.3	1E-02	1.3
NM_177261	Kndc1	1.3	2E-02	1.3	7E-03	1.3
XM_003084838	Gm20091	1.2	2E-02	1.4	4E-03	1.3
NM_010888	Ndufs6	1.3	3E-03	1.3	2E-03	1.3
NM_181394	Anapc13	1.2	8E-03	1.4	5E-04	1.3
ENSMUST00000028335	Grin1	1.3	7E-03	1.3	2E-02	1.3
NM_008427	Kcnj4	1.3	4E-02	1.3	3E-02	1.3
NM_139296	Moxd2	1.2	2E-02	1.3	5E-03	1.3
ENSMUST00000026236	Tlx1	1.3	4E-02	1.3	3E-02	1.3
NM_001171512	Obscn	1.4	2E-03	1.2	2E-02	1.3
ENSMUST00000056571	Syne1	1.3	4E-02	1.3	5E-02	1.3
NM_008233	Hdgfrp2	1.3	9E-04	1.3	1E-03	1.3
NM_001081644	Gm428	1.3	4E-02	1.3	4E-02	1.3
NM_001039198	Zfhx2	1.4	8E-04	1.2	1E-02	1.3
NM_009900	Clcn2	1.3	6E-03	1.3	1E-02	1.3
NM_011482	Nhp2l1	1.3	2E-03	1.3	5E-04	1.3
ENSMUST00000005015	Prcc	1.4	4E-04	1.2	7E-03	1.3
NM_001145537	4930544D05Rik	1.3	1E-02	1.3	4E-03	1.3
NM_001256056	9330182O14Rik	1.4	6E-03	1.2	5E-02	1.3
NM_001081382	Zfp777	1.2	3E-02	1.3	5E-03	1.3
NM_023670	Igf2bp3	1.2	3E-02	1.3	4E-03	1.3
NM_010072	Dpm1	1.3	9E-03	1.2	3E-02	1.3
NM_173016	Vat1l	1.3	3E-02	1.3	3E-02	1.3
NM_153388	Lrfn4	1.3	2E-02	1.3	2E-02	1.3
NM_001164735	Crlf2	1.3	1E-02	1.3	2E-02	1.3
NM_027909	C2cd2l	1.3	1E-02	1.2	5E-02	1.3
NM_001243937	Gm3139	1.3	8E-03	1.3	2E-02	1.3
NM_207678	Ccnl2	1.3	4E-03	1.2	2E-02	1.3
ENSMUST00000023851	Ndufa5	1.2	1E-02	1.3	3E-03	1.3
NM_007747	Cox5a	1.3	2E-03	1.2	1E-02	1.3
NM_133976	Imp3	1.2	8E-03	1.3	7E-04	1.3
NM_009943	Cox6a2	1.2	3E-03	1.3	5E-04	1.3
NM_028064	Slc39a4	1.3	2E-02	1.3	2E-02	1.3
NR_024208	Sycp1-ps1	1.3	7E-03	1.2	3E-02	1.3
NM_024256	B3gat3	1.2	2E-02	1.3	3E-03	1.3
ENSMUST00000004389	Grcc10	1.3	2E-02	1.2	4E-02	1.3
XR_032431	Gm4997	1.2	2E-02	1.3	3E-03	1.3
NM_080285	Cttnbp2	1.2	5E-02	1.3	2E-02	1.3
NM_025376	Tmem8c	1.3	8E-03	1.2	2E-02	1.3
NM_028820	1700017B05Rik	1.2	5E-02	1.3	4E-02	1.3
ENSMUST00000061745	Hoxd10	1.2	1E-02	1.3	5E-03	1.3
NM_029296	1700001C19Rik	1.2	4E-02	1.3	3E-02	1.3
NM_030068	Iqch	1.2	2E-02	1.3	1E-02	1.3
NM_009945	Cox7a2	1.2	4E-03	1.3	2E-03	1.3
NM_172775	Plxnb1	1.3	2E-03	1.3	2E-03	1.3

NM_012053	Rpl8	1.3	5E-03	1.3	5E-03	1.3
NM_007682	Cenpb	1.3	9E-03	1.2	1E-02	1.2
NM_007431	Alpl	1.3	4E-02	1.2	5E-02	1.2
NM_052992	Fxyd1	1.3	1E-04	1.2	2E-04	1.2
AK080553	A730085E03Rik	1.2	4E-02	1.3	3E-02	1.2
NM_175450	Wdr18	1.3	2E-02	1.2	3E-02	1.2
NM_028865	1110005A03Rik	1.2	4E-02	1.3	3E-02	1.2
NM_178631	Ralyl	1.3	3E-02	1.2	5E-02	1.2
NM_172747	Kctd13	1.2	2E-02	1.2	2E-02	1.2
NM_007589	Calm2	1.2	1E-02	1.3	3E-03	1.2
ENSMUST00000032912	Qprt	1.2	1E-02	1.2	1E-02	1.2
NM_009439	Psmd3	1.2	1E-02	1.2	1E-02	1.2
NM_025966	2310039H08Rik	1.2	1E-02	1.2	8E-03	1.2
ENSMUST00000088610	Romo1	1.2	3E-02	1.3	2E-02	1.2
ENSMUST00000163492	Slc29a1	1.2	4E-03	1.2	5E-03	1.2
NM_023326	Bmyc	1.2	3E-02	1.2	2E-02	1.2
NM_053231	Vmn1r10	1.2	3E-02	1.2	4E-02	1.2
NM_177603	Frat2	1.2	5E-02	1.3	2E-02	1.2
NM_001002267	Tmem158	1.2	4E-02	1.3	2E-02	1.2
NM_173013	Mtap1s	1.3	2E-02	1.2	4E-02	1.2
NM_001253843	Prpf19	1.2	9E-03	1.2	9E-03	1.2
NM_001083618	Ttll9	1.2	3E-02	1.2	3E-02	1.2
NM_146075	Lemd2	1.2	9E-04	1.2	7E-04	1.2
NM_178639	Sfxn5	1.2	3E-02	1.2	4E-02	1.2
NM_198613	Ap2s1	1.2	1E-02	1.2	7E-03	1.2
NM_026305	Tceb2	1.2	3E-02	1.2	3E-02	1.2
NM_026063	2900010M23Rik	1.2	3E-02	1.2	5E-02	1.2
NM_001242363	Fam160a2	1.2	2E-02	1.2	4E-02	1.2
NM_009448	Tuba1c	1.2	3E-02	1.2	5E-02	1.2
NM_011193	Pstpip1	1.2	3E-02	1.2	2E-02	1.2
NM_172914	Ccdc113	1.2	1E-02	1.2	3E-02	1.2
NM_016916	Blcap	1.2	3E-02	1.2	5E-02	1.2
NM_133865	Dclre1b	1.2	2E-02	1.2	1E-02	1.2
NM_001145884	Itgb5	1.2	6E-03	1.2	5E-03	1.2
NM_175552	Wdr3	1.2	1E-02	1.2	1E-02	1.2
ENSMUST00000063704	Gm13152	-6.4	2E-06	-1.5	2E-02	-3.9
NM_133871	Ifi44	-4.8	8E-05	-2.2	4E-03	-3.5
NM_001101475	F830016B08Rik	-4.5	4E-05	-2.0	4E-03	-3.2
NM_013751	Hrasls	-5.0	3E-07	-1.4	5E-03	-3.2
NM_172921	Fam55d	-4.5	2E-05	-1.5	3E-02	-3.0
NM_001013769	Rsl1	-3.8	1E-06	-2.1	5E-05	-3.0
NM_028747	0610012H03Rik	-3.9	3E-07	-1.5	7E-04	-2.7
NM_008777	Pah	-3.5	2E-04	-1.7	2E-02	-2.6
BC147845	Uprt	-2.5	5E-06	-2.6	4E-06	-2.6
NM_027698	Eri2	-2.7	3E-04	-2.3	9E-04	-2.5
NM_009082	Rpl29	-3.5	4E-06	-1.3	3E-02	-2.4

NM_145545	Gbp7	-2.7	7E-03	-2.0	4E-02	-2.3
NM_010554	Il1a	-1.8	7E-03	-2.8	3E-04	-2.3
NM_007646	Cd38	-2.5	5E-05	-2.0	3E-04	-2.3
NM_011765	Zfp97	-2.7	2E-03	-1.7	4E-02	-2.2
NM_020557	Cmpk2	-2.8	1E-05	-1.5	3E-03	-2.2
NM_001256005	Gbp4	-2.6	2E-03	-1.8	3E-02	-2.2
NM_018887	Cyp39a1	-2.7	6E-05	-1.6	4E-03	-2.2
NM_009855	Cd80	-2.5	6E-04	-1.8	6E-03	-2.2
NM_025679	Sike1	-2.1	3E-06	-2.2	2E-06	-2.1
NM_011654	Tuba1b	-1.7	2E-02	-2.5	1E-03	-2.1
NM_013912	Apln	-2.3	9E-04	-1.9	4E-03	-2.1
NM_011419	Kdm5d	-2.9	7E-07	-1.3	6E-03	-2.1
NM_153564	Gbp5	-2.6	9E-04	-1.6	3E-02	-2.1
NM_019440	Irgm2	-2.4	5E-04	-1.7	6E-03	-2.1
NM_026810	Mlh1	-2.6	1E-04	-1.5	2E-02	-2.1
NM_001113478	Frrs1	-2.2	7E-05	-1.9	3E-04	-2.0
ENSMUST00000115217	Napepld	-1.9	3E-03	-2.1	1E-03	-2.0
ENSMUST00000029270	Ccna2	-2.4	9E-04	-1.6	2E-02	-2.0
NM_146203	Zfp764	-2.1	1E-03	-1.9	2E-03	-2.0
NM_028319	Zfp518a	-1.9	1E-05	-2.1	4E-06	-2.0
NM_001033780	I830077J02Rik	-2.3	3E-03	-1.6	5E-02	-2.0
NM_016923	Ly96	-2.1	4E-04	-1.8	2E-03	-1.9
NM_001025373	Zfp943	-2.4	4E-05	-1.4	9E-03	-1.9
NM_027654	Pcgf6	-1.9	6E-03	-1.9	6E-03	-1.9
NM_011909	Usp18	-2.3	2E-03	-1.5	5E-02	-1.9
NM_015754	Rbbp9	-1.9	9E-04	-1.9	1E-03	-1.9
NM_177351	Agphd1	-1.9	3E-04	-1.9	2E-04	-1.9
NM_001252478	Slc30a6	-2.2	6E-04	-1.6	9E-03	-1.9
NM_178752	D330012F22Rik	-2.1	2E-05	-1.7	2E-04	-1.9
NM_001104563	Vmn2r101	-1.9	3E-02	-1.8	4E-02	-1.9
NM_001081075	5730455O13Rik	-2.0	1E-03	-1.7	3E-03	-1.9
NM_001042499	Rabl3	-2.1	6E-04	-1.6	6E-03	-1.9
NM_053143	Pcdhb18	-1.7	3E-02	-2.0	7E-03	-1.8
NM_011623	Top2a	-2.2	6E-04	-1.5	1E-02	-1.8
ENSMUST00000107439	Krtap4-8	-2.1	1E-03	-1.6	1E-02	-1.8
NM_175403	Mlec	-2.2	3E-05	-1.5	3E-03	-1.8
ENSMUST00000081186	Vmn1r21	-1.7	8E-03	-2.0	2E-03	-1.8
NM_008249	Tfb2m	-2.0	2E-02	-1.7	4E-02	-1.8
NM_175026	Pyhin1	-2.3	2E-04	-1.4	3E-02	-1.8
ENSMUST00000162512	Gtpbp8	-1.8	3E-04	-1.8	3E-04	-1.8
NM_011267	Rgs16	-1.6	3E-02	-2.1	2E-03	-1.8
NM_019425	Gnpnat1	-1.9	9E-04	-1.7	2E-03	-1.8
NM_145584	Spon1	-2.2	4E-04	-1.4	3E-02	-1.8
NR_038044	Gm10033	-2.0	6E-03	-1.6	3E-02	-1.8
AK007274	Fbxw27	-1.7	1E-02	-2.0	3E-03	-1.8
NM_007554	Bmp4	-1.9	1E-02	-1.7	3E-02	-1.8

NM_019683	Ankrd49	-1.8	6E-03	-1.8	7E-03	-1.8
NM_001081324	Neto2	-1.6	5E-04	-2.0	3E-05	-1.8
NM_026635	Fam96a	-2.1	9E-05	-1.5	3E-03	-1.8
NM_011607	Tnc	-1.9	2E-02	-1.7	3E-02	-1.8
NM_178070	Vps33b	-1.8	9E-03	-1.7	1E-02	-1.8
NM_001252639	Tbc1d7	-1.7	5E-04	-1.9	1E-04	-1.8
NM_153780	2610044O15Rik	-2.0	3E-05	-1.6	5E-04	-1.8
BC063263	Zfp141	-2.0	3E-03	-1.6	2E-02	-1.8
ENSMUST00000106157	Zranb1	-2.1	3E-04	-1.5	1E-02	-1.8
NM_001255990	Gm1553	-1.7	3E-03	-1.8	2E-03	-1.8
NM_001103182	Lin9	-2.0	2E-05	-1.6	3E-04	-1.8
NM_021384	Rsad2	-2.2	7E-06	-1.4	2E-03	-1.8
ENSMUST00000114900	Zc3hav1	-1.6	6E-04	-1.9	1E-04	-1.8
NM_011864	Papss2	-1.8	5E-05	-1.7	2E-04	-1.8
NM_019989	Sh3bgrl	-1.8	4E-04	-1.7	9E-04	-1.8
NM_001033284	BC026585	-1.7	2E-03	-1.8	8E-04	-1.8
NM_007669	Cdkn1a	-1.8	3E-02	-1.7	4E-02	-1.8
ENSMUST00000110455	Hist1h2bk	-1.8	1E-02	-1.7	3E-02	-1.8
NM_001013387	Zfp182	-2.0	3E-04	-1.6	4E-03	-1.8
NM_007934	Enpep	-2.0	1E-04	-1.5	3E-03	-1.8
NM_012048	Polk	-1.8	1E-03	-1.7	3E-03	-1.8
ENSMUST00000025170	Wdr46	-1.9	3E-04	-1.6	2E-03	-1.7
BC056964	Taf1d	-2.0	3E-03	-1.5	4E-02	-1.7
NM_153098	Cd109	-1.7	1E-02	-1.8	7E-03	-1.7
NM_001010839	Taar7f	-1.5	1E-02	-2.0	3E-04	-1.7
NM_010283	Ggta1	-1.9	2E-04	-1.6	2E-03	-1.7
NM_013831	Pstpip2	-1.9	3E-04	-1.6	1E-03	-1.7
NM_027464	5730469M10Rik	-1.6	2E-02	-1.8	6E-03	-1.7
NM_145148	Frmd4b	-1.7	1E-03	-1.8	5E-04	-1.7
NM_199311	Clec4a1	-2.0	2E-03	-1.4	5E-02	-1.7
NM_028349	Sass6	-1.9	3E-04	-1.6	3E-03	-1.7
NM_013813	Epb4.1l3	-1.9	2E-03	-1.5	1E-02	-1.7
NM_011232	Rad1	-1.8	3E-04	-1.6	1E-03	-1.7
NM_172773	Slc17a5	-1.7	1E-05	-1.7	1E-05	-1.7
NM_027324	Sfxn1	-1.9	6E-04	-1.6	3E-03	-1.7
NM_026345	Mansc1	-1.6	3E-05	-1.8	6E-06	-1.7
NM_011852	Oas1g	-2.1	5E-04	-1.4	3E-02	-1.7
NM_138751	Tmem47	-1.5	2E-03	-2.0	9E-05	-1.7
NM_001033181	Jrkl	-1.8	4E-03	-1.7	5E-03	-1.7
NM_011932	Dapp1	-1.8	9E-04	-1.6	3E-03	-1.7
NM_023142	Arpc1b	-1.8	1E-03	-1.7	3E-03	-1.7
NM_053145	Pcdhb20	-1.8	1E-03	-1.7	2E-03	-1.7
NM_023485	Sync	-1.7	4E-04	-1.8	2E-04	-1.7
ENSMUST00000108491	Ceacam3	-1.7	3E-04	-1.7	3E-04	-1.7
NM_172862	Frem2	-1.5	1E-03	-1.9	7E-05	-1.7
ENSMUST00000067582	Tmem62	-1.9	6E-05	-1.5	6E-04	-1.7

NM_025415	Cks2	-1.9	3E-03	-1.5	2E-02	-1.7
NM_008212	Hadh	-2.2	1E-07	-1.2	7E-04	-1.7
NM_007900	Ect2	-1.8	4E-03	-1.6	2E-02	-1.7
NM_177355	Plcxd3	-1.7	2E-02	-1.7	3E-02	-1.7
NR_046277	4930479D17Rik	-1.7	1E-02	-1.7	1E-02	-1.7
NM_009555	Zfp40	-1.8	8E-04	-1.6	2E-03	-1.7
NM_178675	Slc35f1	-1.7	3E-02	-1.7	3E-02	-1.7
NR_029849	Mir361	-1.8	9E-03	-1.6	2E-02	-1.7
NM_177382	Cyp2r1	-2.0	1E-05	-1.4	2E-03	-1.7
NM_008222	Hccs	-2.0	6E-05	-1.4	3E-03	-1.7
NM_028943	Sgms2	-1.9	9E-04	-1.5	1E-02	-1.7
NM_053141	Pcdhb16	-1.6	6E-03	-1.7	4E-03	-1.7
NM_001166710	Vmn1r7	-1.4	7E-04	-2.0	5E-06	-1.7
NM_009812	Casp8	-1.9	7E-06	-1.5	2E-04	-1.7
ENSMUST00000039373	Uba6	-1.9	5E-04	-1.5	7E-03	-1.7
NM_020278	Lgi1	-1.8	5E-03	-1.5	2E-02	-1.7
NM_029000	Gvin1	-1.9	4E-03	-1.4	5E-02	-1.7
NM_153543	Aldh1l2	-1.8	8E-03	-1.5	4E-02	-1.7
NM_172784	Lrp11	-1.5	3E-02	-1.8	8E-03	-1.7
NM_026011	Arl8b	-1.7	3E-05	-1.7	2E-05	-1.7
NM_144558	Bivm	-1.7	4E-04	-1.6	1E-03	-1.7
NR_029561	Mir151	-1.8	6E-04	-1.5	3E-03	-1.7
ENSMUST00000116437	Snap23	-1.7	4E-04	-1.6	9E-04	-1.7
NM_175271	Lpar4	-1.8	4E-03	-1.5	3E-02	-1.7
NM_145523	Gca	-1.4	3E-02	-1.9	5E-04	-1.7
NM_153143	Kctd11	-1.8	8E-04	-1.5	8E-03	-1.7
NM_029645	Gatc	-1.7	1E-03	-1.6	4E-03	-1.7
NM_024282	Pppde1	-1.5	4E-02	-1.8	1E-02	-1.6
NM_026398	Pop5	-1.8	2E-05	-1.5	4E-04	-1.6
ENSMUST00000115812	Pik3c3	-1.9	2E-04	-1.4	6E-03	-1.6
NM_010008	Cyp2j6	-1.9	1E-03	-1.4	3E-02	-1.6
NM_008924	Prkar2a	-1.6	2E-03	-1.7	1E-03	-1.6
NM_172392	Zfp759	-1.8	7E-04	-1.5	8E-03	-1.6
NM_010786	Mdm2	-1.6	2E-04	-1.7	5E-05	-1.6
NM_198019	Cep78	-1.7	3E-03	-1.6	5E-03	-1.6
NM_001177550	Zfp442	-1.7	1E-02	-1.5	3E-02	-1.6
NM_172600	6720456H20Rik	-1.8	4E-04	-1.5	5E-03	-1.6
NM_144846	Fam49b	-1.7	1E-03	-1.6	2E-03	-1.6
NM_001004066	Zfp386	-1.9	2E-05	-1.4	8E-04	-1.6
ENSMUST00000145910	Strn	-1.9	1E-05	-1.3	2E-03	-1.6
NM_009283	Stat1	-1.6	1E-02	-1.7	5E-03	-1.6
NM_172733	Dera	-1.5	1E-02	-1.8	2E-03	-1.6
NM_011650	Tsn	-1.5	2E-04	-1.7	5E-05	-1.6
NM_144945	Lgi2	-1.7	9E-04	-1.5	3E-03	-1.6
NM_018788	Extl3	-1.5	2E-02	-1.7	5E-03	-1.6
NM_024291	Ky	-1.4	4E-03	-1.8	1E-04	-1.6

NM_025921	2610002M06Rik	-1.4	3E-03	-1.8	1E-04	-1.6
NM_001253355	Hs3st5	-1.5	5E-03	-1.7	8E-04	-1.6
NM_181589	Ckap2l	-1.8	1E-03	-1.4	1E-02	-1.6
NM_007474	Aqp8	-1.8	4E-03	-1.4	4E-02	-1.6
NM_026232	Slc25a30	-1.7	5E-04	-1.5	2E-03	-1.6
NM_053078	D0H4S114	-1.8	2E-04	-1.4	6E-03	-1.6
NM_021789	Trappc4	-1.9	1E-04	-1.3	1E-02	-1.6
NR_045175	2700081L22Rik	-1.6	3E-02	-1.6	2E-02	-1.6
NM_011050	Pdcd4	-1.6	1E-03	-1.6	7E-04	-1.6
NM_053142	Pcdhb17	-1.5	7E-04	-1.7	3E-04	-1.6
NM_010415	Hbegf	-1.8	2E-04	-1.4	4E-03	-1.6
ENSMUST00000117146	Ube2w	-1.7	2E-03	-1.5	5E-03	-1.6
NM_025705	Dcbld1	-1.6	2E-02	-1.6	1E-02	-1.6
NM_021315	Noc3l	-1.8	4E-06	-1.4	9E-05	-1.6
NM_133955	Rhou	-1.6	2E-03	-1.6	2E-03	-1.6
NM_172877	Pramef8	-1.7	2E-04	-1.5	2E-03	-1.6
NM_144517	Tbc1d19	-1.6	2E-03	-1.5	5E-03	-1.6
NM_026992	Dnajc24	-1.5	2E-02	-1.6	1E-02	-1.6
NM_001099330	Gm12776	-1.7	8E-04	-1.5	2E-03	-1.6
ENSMUST00000038406	Akr1b8	-1.8	4E-03	-1.4	4E-02	-1.6
NM_007691	Chek1	-1.7	3E-03	-1.5	8E-03	-1.6
ENSMUST00000029414	Ssr3	-1.6	8E-04	-1.6	5E-04	-1.6
NM_030110	Efha2	-1.5	2E-02	-1.7	2E-03	-1.6
NM_178308	Abpg	-1.6	2E-03	-1.6	3E-03	-1.6
NM_011515	Vamp7	-1.7	2E-03	-1.5	6E-03	-1.6
NM_178889	Zscan29	-1.7	2E-04	-1.5	5E-04	-1.6
ENSMUST00000177184	Vmn2r5	-1.7	9E-03	-1.4	5E-02	-1.6
NM_001004149	Zfp366	-1.6	2E-02	-1.6	3E-02	-1.6
NM_011763	Zfp9	-1.7	2E-03	-1.5	1E-02	-1.6
NM_146231	Zfp825	-1.6	1E-02	-1.5	2E-02	-1.6
NM_008971	Twf1	-1.8	2E-04	-1.4	3E-03	-1.6
NM_001081179	Heatr5b	-1.8	1E-04	-1.3	7E-03	-1.6
NM_145524	Mettl8	-1.5	6E-04	-1.6	2E-04	-1.6
NM_001135577	BC024659	-1.3	4E-02	-1.8	1E-03	-1.6
ENSMUST00000021173	Mfsd11	-1.4	9E-04	-1.8	3E-05	-1.6
ENSMUST00000023686	Tmem50b	-1.6	1E-04	-1.6	9E-05	-1.6
NM_010085	Adam26a	-1.8	7E-04	-1.4	2E-02	-1.6
NM_054089	Tgs1	-1.5	5E-04	-1.7	1E-04	-1.6
NM_011249	Rbl1	-1.6	1E-04	-1.5	4E-04	-1.6
ENSMUST00000031171	Stap1	-1.7	9E-03	-1.5	3E-02	-1.6
ENSMUST00000105439	Lrrtm3	-1.6	9E-03	-1.5	2E-02	-1.6
NM_001145676	2210408I21Rik	-1.8	2E-03	-1.4	3E-02	-1.6
NM_001081090	Esf1	-1.6	5E-03	-1.6	4E-03	-1.6
NM_146529	Olfr1183	-1.3	2E-02	-1.9	1E-04	-1.6
NM_011274	C80913	-1.6	1E-03	-1.5	2E-03	-1.6
NM_001039088	Seh1l	-1.6	5E-05	-1.6	5E-05	-1.6

ENSMUST00000034761	Narg2	-1.6	4E-02	-1.6	4E-02	-1.6
NM_001115087	Fancf	-1.5	1E-02	-1.7	3E-03	-1.6
NM_025770	Atg10	-1.5	2E-02	-1.7	7E-03	-1.6
NM_026895	Tatdn3	-1.7	5E-03	-1.4	3E-02	-1.6
NM_172958	Mtmr12	-1.5	4E-03	-1.6	2E-03	-1.6
ENSMUST00000030296	Txndc12	-1.7	2E-03	-1.4	2E-02	-1.6
NM_001101478	D3Ertd254e	-1.7	6E-03	-1.5	2E-02	-1.6
NM_023292	Pus3	-1.5	5E-03	-1.6	2E-03	-1.6
NM_008032	Aff2	-1.4	2E-02	-1.7	2E-03	-1.6
NM_133738	Antxr2	-1.7	9E-04	-1.4	1E-02	-1.6
ENSMUST00000030074	Ugcg	-1.5	5E-03	-1.7	1E-03	-1.6
NM_027421	Ints2	-1.5	1E-02	-1.6	6E-03	-1.6
NM_013844	Zfp68	-1.7	1E-03	-1.5	6E-03	-1.6
NM_001163763	Tcf19	-1.7	4E-03	-1.4	3E-02	-1.6
NM_015812	Rgs6	-1.7	4E-03	-1.4	5E-02	-1.6
NM_028002	Dus4l	-1.5	1E-02	-1.6	8E-03	-1.6
NM_028053	Tmem38b	-1.7	2E-04	-1.4	5E-03	-1.6
ENSMUST00000044382	Zc4h2	-1.7	3E-03	-1.4	2E-02	-1.6
NM_023750	Zfp84	-1.6	9E-04	-1.5	1E-03	-1.6
NM_001081680	Zfp72	-1.6	3E-02	-1.6	3E-02	-1.6
NM_198605	F630043A04Rik	-1.4	5E-02	-1.8	3E-03	-1.6
NR_040402	Gm19522	-1.7	6E-03	-1.4	2E-02	-1.6
NM_145392	Bag2	-1.5	4E-02	-1.6	3E-02	-1.6
NM_001142357	Alg12	-1.4	2E-02	-1.7	3E-03	-1.6
NM_023587	Ptplb	-1.6	5E-04	-1.5	1E-03	-1.6
ENSMUST00000031402	Cct6a	-1.8	3E-05	-1.3	4E-03	-1.6
BC052634	Wdyhv1	-1.5	4E-02	-1.6	3E-02	-1.6
NM_025716	Spryd4	-1.4	1E-02	-1.7	1E-03	-1.6
ENSMUST00000053407	Alkbh8	-1.7	3E-03	-1.4	2E-02	-1.5
NM_181732	Aida	-1.8	2E-04	-1.3	7E-03	-1.5
NM_145930	AW549877	-1.6	3E-06	-1.5	3E-06	-1.5
NM_010615	Kif11	-1.8	3E-04	-1.3	4E-02	-1.5
NM_030692	Sacm1l	-1.9	2E-08	-1.2	5E-05	-1.5
NM_009808	Casp12	-1.7	2E-03	-1.4	2E-02	-1.5
NM_026437	Fam45a	-1.7	1E-04	-1.3	4E-03	-1.5
NM_030069	Fam55b	-1.8	1E-04	-1.3	1E-02	-1.5
ENSMUST00000041905	Nupl1	-1.5	2E-04	-1.5	2E-04	-1.5
NM_001081288	Taf2	-1.4	9E-04	-1.7	1E-04	-1.5
ENSMUST00000034457	Urb2	-1.6	2E-03	-1.5	7E-03	-1.5
NM_178283	Asb13	-1.7	4E-06	-1.4	7E-05	-1.5
ENSMUST00000034591	Bace1	-1.5	1E-03	-1.6	8E-04	-1.5
NM_027088	Bap1	-1.6	3E-05	-1.5	5E-05	-1.5
NM_001164621	Rnf14	-1.4	5E-04	-1.7	1E-05	-1.5
NM_011801	Cfdp1	-1.7	4E-03	-1.4	5E-02	-1.5
ENSMUST00000115441	Gtpbp10	-1.5	2E-02	-1.6	7E-03	-1.5
NM_001145898	BC052040	-1.5	8E-03	-1.6	4E-03	-1.5

NM_134005	Enpp3	-1.7	2E-03	-1.4	1E-02	-1.5
NR_015618	A930015D03Rik	-1.6	4E-04	-1.4	2E-03	-1.5
ENSMUST00000042163	Naa25	-1.6	7E-04	-1.5	2E-03	-1.5
NM_175007	Amph	-1.6	2E-02	-1.5	3E-02	-1.5
NM_144904	Rod1	-1.6	7E-03	-1.5	1E-02	-1.5
ENSMUST00000097772	Il1rl1	-1.7	1E-05	-1.3	6E-04	-1.5
NM_019748	Sae1	-1.6	4E-03	-1.5	9E-03	-1.5
NM_172920	Dpy19l1	-1.7	3E-03	-1.4	2E-02	-1.5
ENSMUST00000121465	Fuca2	-1.4	9E-04	-1.6	2E-04	-1.5
NM_145502	Erlin1	-1.6	6E-04	-1.4	5E-03	-1.5
NM_001024622	Pcnp	-1.4	3E-02	-1.6	1E-02	-1.5
ENSMUST00000058030	Mtap	-1.5	2E-03	-1.5	3E-03	-1.5
NM_001190466	Dact1	-1.6	9E-03	-1.5	2E-02	-1.5
NM_172598	Wdhd1	-1.6	1E-02	-1.5	2E-02	-1.5
NM_016785	Tpmt	-1.7	4E-04	-1.3	1E-02	-1.5
NM_007610	Casp2	-1.4	7E-03	-1.6	2E-03	-1.5
NM_145374	Mios	-1.7	4E-04	-1.3	2E-02	-1.5
NM_177038	Trappc8	-1.6	3E-05	-1.4	2E-04	-1.5
NM_032003	Enpp5	-1.7	4E-04	-1.3	2E-02	-1.5
NM_026490	Mrpl19	-1.4	3E-02	-1.6	7E-03	-1.5
ENSMUST00000072438	Cyp2b10	-1.6	2E-02	-1.5	5E-02	-1.5
NM_173744	2610019F03Rik	-1.4	3E-02	-1.6	8E-03	-1.5
ENSMUST00000128667	Fktn	-1.6	2E-02	-1.5	3E-02	-1.5
NM_020578	Ehd3	-1.4	9E-03	-1.7	6E-04	-1.5
NM_028908	4933403G14Rik	-1.4	2E-02	-1.6	4E-03	-1.5
NM_183116	1110021L09Rik	-1.5	8E-04	-1.5	1E-03	-1.5
NM_197982	Ddx39	-1.5	7E-03	-1.5	6E-03	-1.5
NM_025558	Cyb5b	-1.5	4E-03	-1.5	7E-03	-1.5
ENSMUST00000039541	Tubgcp4	-1.5	1E-05	-1.5	1E-05	-1.5
NM_172565	Klhl11	-1.4	4E-02	-1.6	1E-02	-1.5
NM_026448	Klhl7	-1.4	2E-03	-1.6	2E-04	-1.5
NM_019499	Mad2l1	-1.6	9E-04	-1.5	2E-03	-1.5
ENSMUST00000062289	Bend6	-1.5	1E-02	-1.5	2E-02	-1.5
ENSMUST00000094014	Fam57a	-1.5	3E-04	-1.5	2E-04	-1.5
NM_178925	Nsun3	-1.7	3E-03	-1.4	3E-02	-1.5
ENSMUST00000020204	Ntn4	-1.5	4E-03	-1.5	5E-03	-1.5
NM_011625	Ppp1r13b	-1.5	8E-04	-1.5	8E-04	-1.5
NM_011083	Pik3c2a	-1.5	3E-02	-1.6	1E-02	-1.5
NM_001024147	Gm5868	-1.5	3E-02	-1.5	3E-02	-1.5
NM_181315	Car5b	-1.5	1E-02	-1.5	5E-03	-1.5
NM_001077190	Abi1	-1.6	3E-03	-1.5	7E-03	-1.5
NM_029965	Rnf170	-1.4	2E-04	-1.6	4E-05	-1.5
AB540946	Vmn2r115	-1.6	1E-02	-1.4	3E-02	-1.5
ENSMUST00000021424	Sptlc2	-1.6	2E-03	-1.4	7E-03	-1.5
NM_011494	Stk16	-1.4	6E-03	-1.6	9E-04	-1.5
NM_001042719	Ddhd1	-1.5	1E-03	-1.5	1E-03	-1.5

NM_025815	Cpne8	-1.5	3E-02	-1.5	4E-02	-1.5
NM_001011811	Olfr487	-1.5	6E-03	-1.5	1E-02	-1.5
NM_013518	Fgf9	-1.2	2E-02	-1.8	7E-05	-1.5
NM_001002268	Gpr126	-1.7	3E-04	-1.3	2E-02	-1.5
NM_016908	Syt5	-1.5	1E-02	-1.5	2E-02	-1.5
NM_145586	Tmem159	-1.6	2E-04	-1.4	2E-03	-1.5
ENSMUST00000109731	Cdk5rap1	-1.3	1E-02	-1.7	6E-04	-1.5
ENSMUST00000063465	Wnt5a	-1.4	1E-02	-1.6	5E-03	-1.5
NM_001167730	Rad18	-1.5	2E-03	-1.5	2E-03	-1.5
NM_008397	Itga6	-1.5	9E-03	-1.5	1E-02	-1.5
ENSMUST00000099756	Olfr1269	-1.5	6E-03	-1.5	5E-03	-1.5
NM_145964	Ap1ar	-1.4	3E-03	-1.6	2E-04	-1.5
NM_001030307	Dkc1	-1.4	2E-02	-1.6	3E-03	-1.5
NM_010282	Ggps1	-1.6	2E-04	-1.4	2E-03	-1.5
NM_010496	Id2	-1.5	9E-03	-1.5	9E-03	-1.5
NM_176953	Lig4	-1.6	5E-03	-1.4	1E-02	-1.5
ENSMUST00000029891	Tmem68	-1.5	6E-03	-1.5	1E-02	-1.5
NM_026244	Slc39a9	-1.6	4E-04	-1.4	2E-03	-1.5
NM_133193	Il1rl2	-1.5	1E-02	-1.5	2E-02	-1.5
ENSMUST00000094541	Btbd8	-1.5	1E-03	-1.4	4E-03	-1.5
ENSMUST00000076123	Zfp58	-1.3	3E-02	-1.7	1E-03	-1.5
NM_181590	Shq1	-1.5	2E-02	-1.5	1E-02	-1.5
NM_177815	Rft1	-1.5	2E-02	-1.5	2E-02	-1.5
NM_175087	Aqp6	-1.6	1E-02	-1.4	3E-02	-1.5
ENSMUST00000155487	Hiatl1	-1.6	4E-03	-1.4	1E-02	-1.5
NM_007879	Drg1	-1.5	4E-05	-1.5	5E-05	-1.5
ENSMUST00000101208	Sod3	-1.6	8E-03	-1.4	3E-02	-1.5
NM_011421	Smpd1	-1.4	1E-02	-1.6	1E-03	-1.5
NM_026887	Ap1s2	-1.4	8E-03	-1.6	2E-03	-1.5
NR_040435	E130218I03Rik	-1.6	5E-03	-1.4	4E-02	-1.5
NM_009498	Vamp3	-1.5	8E-05	-1.5	2E-04	-1.5
ENSMUST00000084949	Impad1	-1.6	3E-04	-1.4	3E-03	-1.5
NM_130447	Dusp16	-1.4	2E-02	-1.6	2E-03	-1.5
NM_013692	Klf10	-1.4	3E-02	-1.6	3E-03	-1.5
NM_212444	Gyk	-1.4	4E-03	-1.6	4E-04	-1.5
ENSMUST00000155551	Dcaf10	-1.5	1E-02	-1.5	8E-03	-1.5
NM_009101	Rras	-1.5	1E-02	-1.4	3E-02	-1.5
ENSMUST00000069870	Arl5b	-1.3	9E-03	-1.7	3E-04	-1.5
NM_001113211	Tmem194	-1.4	5E-02	-1.6	8E-03	-1.5
ENSMUST00000039769	Sntb1	-1.5	4E-03	-1.5	3E-03	-1.5
NM_025985	Ube2g1	-1.4	5E-04	-1.5	2E-04	-1.5
NM_026981	Dtwd1	-1.6	2E-03	-1.4	9E-03	-1.5
NM_026184	Ero1lb	-1.5	1E-05	-1.4	4E-05	-1.5
NM_026658	Mto1	-1.5	4E-03	-1.5	3E-03	-1.5
NM_053139	Pcdhb14	-1.6	8E-03	-1.4	3E-02	-1.5
NM_021890	Fads3	-1.5	4E-04	-1.5	3E-04	-1.5

NM_025829	Eif4e3	-1.4	1E-04	-1.6	2E-05	-1.5
NM_019673	Actl6a	-1.5	2E-04	-1.5	2E-04	-1.5
NM_008697	Nin	-1.7	3E-04	-1.3	1E-02	-1.5
ENSMUST00000060108	1810030O07Rik	-1.5	2E-04	-1.5	1E-04	-1.5
NM_027869	Pnpt1	-1.4	1E-02	-1.5	5E-03	-1.5
NM_139143	Slc39a6	-1.5	3E-02	-1.5	3E-02	-1.5
NM_001081071	Lclat1	-1.2	3E-02	-1.7	2E-04	-1.5
NM_146701	Olfr1448	-1.5	8E-03	-1.4	2E-02	-1.5
NM_178773	Ano4	-1.5	2E-02	-1.4	4E-02	-1.5
ENSMUST00000108293	Tmem67	-1.6	5E-04	-1.4	3E-03	-1.5
NM_025998	Nkain1	-1.6	9E-04	-1.4	5E-03	-1.5
ENSMUST00000034000	Asah1	-1.7	1E-03	-1.3	2E-02	-1.5
NM_009147	Sec23a	-1.5	2E-04	-1.5	3E-04	-1.5
NM_001243908	Zfp383	-1.6	2E-03	-1.4	2E-02	-1.5
NM_029570	Atp11b	-1.4	3E-05	-1.6	4E-06	-1.5
NM_025813	Mfsd1	-1.5	1E-03	-1.5	2E-03	-1.5
NM_181401	Tmem64	-1.4	4E-03	-1.6	7E-04	-1.5
NM_177755	Klhl38	-1.5	7E-03	-1.4	1E-02	-1.5
NM_019437	Rfk	-1.5	1E-03	-1.4	3E-03	-1.5
NM_024472	Gltpd1	-1.5	3E-02	-1.5	2E-02	-1.5
NR_027872	4930515G01Rik	-1.5	4E-02	-1.5	3E-02	-1.5
NM_152809	Csnk1g3	-1.6	3E-03	-1.4	2E-02	-1.5
NM_017480	Icos	-1.3	1E-02	-1.6	4E-04	-1.5
NR_027492	Hspa13	-1.6	4E-03	-1.4	3E-02	-1.5
NM_001040026	Sco1	-1.4	9E-04	-1.5	4E-04	-1.5
NM_030188	Ttc30a1	-1.4	2E-02	-1.5	9E-03	-1.5
ENSMUST00000026927	Nudt5	-1.5	3E-03	-1.5	4E-03	-1.5
ENSMUST00000115110	Hyls1	-1.7	8E-04	-1.3	4E-02	-1.5
NM_029394	Snx24	-1.5	2E-04	-1.4	5E-04	-1.5
NM_008917	Ppt1	-1.6	5E-03	-1.4	4E-02	-1.5
NM_013918	Usp25	-1.4	7E-05	-1.5	4E-05	-1.5
NM_019941	Zfp235	-1.5	4E-02	-1.5	4E-02	-1.5
NM_145531	Spg11	-1.5	4E-04	-1.4	9E-04	-1.5
ENSMUST00000030306	Hook1	-1.4	1E-02	-1.6	3E-03	-1.5
NM_175128	4930430F08Rik	-1.5	2E-02	-1.5	3E-02	-1.5
ENSMUST00000115160	Tmem209	-1.5	7E-04	-1.4	2E-03	-1.5
NM_009029	Rb1	-1.4	1E-04	-1.5	5E-05	-1.5
NM_025841	Kdelr2	-1.4	1E-03	-1.5	6E-04	-1.5
ENSMUST00000162592	Zfp27	-1.5	1E-02	-1.4	2E-02	-1.5
ENSMUST00000069792	Nab1	-1.4	4E-04	-1.5	8E-05	-1.5
NM_001081187	Htra4	-1.7	5E-04	-1.3	2E-02	-1.5
NM_028785	Dock8	-1.5	1E-03	-1.5	1E-03	-1.5
NM_019936	Cript	-1.5	2E-04	-1.4	6E-04	-1.5
NM_013745	Nufip1	-1.5	7E-04	-1.4	2E-03	-1.5
NR_015524	4932415G12Rik	-1.5	3E-02	-1.4	4E-02	-1.5
NM_011233	Rad17	-1.4	3E-04	-1.5	6E-05	-1.5

NM_026662	Prps2	-1.5	2E-02	-1.4	3E-02	-1.5
ENSMUST00000088658	Mybl1	-1.5	5E-03	-1.4	2E-02	-1.5
NM_144535	Mudeng	-1.4	6E-04	-1.5	3E-04	-1.5
NM_001111060	Cd59a	-1.5	5E-04	-1.4	9E-04	-1.5
NM_001080941	Zfp429	-1.6	8E-03	-1.4	4E-02	-1.5
NM_178638	Tmem108	-1.4	5E-02	-1.5	2E-02	-1.5
ENSMUST00000150285	Slc35d1	-1.4	1E-02	-1.6	2E-03	-1.5
NM_009673	Anxa5	-1.6	3E-03	-1.3	4E-02	-1.5
ENSMUST00000113422	Hdx	-1.4	7E-03	-1.5	5E-03	-1.5
ENSMUST00000003843	Man1a	-1.5	3E-03	-1.4	5E-03	-1.5
ENSMUST00000003720	Crot	-1.5	8E-05	-1.4	2E-04	-1.5
NM_011955	Nubp1	-1.3	2E-03	-1.6	1E-04	-1.5
NM_001081204	B3galtl	-1.6	1E-03	-1.3	1E-02	-1.5
NM_176917	Mettl4	-1.4	6E-04	-1.5	2E-04	-1.5
NM_152825	Usp45	-1.6	7E-03	-1.4	4E-02	-1.5
NM_153591	Nars2	-1.4	2E-02	-1.5	8E-03	-1.5
NM_054041	Antxr1	-1.6	2E-03	-1.3	2E-02	-1.5
NM_024272	Ssbp2	-1.4	1E-03	-1.5	7E-04	-1.5
BC147588	4930449I24Rik	-1.4	2E-02	-1.5	3E-03	-1.5
NM_153592	Erlin2	-1.6	4E-04	-1.3	5E-03	-1.5
ENSMUST00000034610	Pate4	-1.5	2E-02	-1.4	5E-02	-1.5
NM_178778	Scai	-1.4	2E-02	-1.5	1E-02	-1.5
NM_177271	Samd5	-1.4	2E-03	-1.5	5E-04	-1.5
NM_134250	Havcr2	-1.6	2E-03	-1.3	2E-02	-1.5
NM_029623	3110002H16Rik	-1.5	4E-03	-1.4	6E-03	-1.5
NR_040439	1810006J02Rik	-1.5	6E-03	-1.4	9E-03	-1.5
NM_175751	Zfp608	-1.7	5E-04	-1.3	3E-02	-1.5
NM_022327	Ralb	-1.5	1E-02	-1.4	2E-02	-1.5
NM_026632	Rpa3	-1.5	7E-03	-1.4	2E-02	-1.5
NM_011992	Rcn2	-1.5	3E-03	-1.4	1E-02	-1.5
NM_133737	Lancl2	-1.4	5E-03	-1.5	4E-03	-1.5
NM_015824	Orc3	-1.6	8E-04	-1.3	8E-03	-1.5
NM_197990	1700025G04Rik	-1.3	1E-02	-1.6	4E-04	-1.5
ENSMUST00000031117	Gnpda2	-1.4	7E-03	-1.5	4E-03	-1.5
NM_183185	Zfp300	-1.5	2E-02	-1.4	2E-02	-1.5
ENSMUST00000114222	Gng12	-1.4	6E-04	-1.5	5E-04	-1.5
NM_016909	Tsnax	-1.5	1E-03	-1.4	1E-03	-1.5
ENSMUST00000096066	Cpa2	-1.4	5E-03	-1.5	4E-03	-1.5
ENSMUST00000153470	l7Rn6	-1.5	2E-03	-1.4	3E-03	-1.4
NM_177171	Heatr5a	-1.5	1E-03	-1.4	2E-03	-1.4
ENSMUST00000159645	Vprbp	-1.5	1E-03	-1.4	2E-03	-1.4
NM_178614	Samm50	-1.4	1E-03	-1.5	3E-04	-1.4
NM_029057	Tbc1d30	-1.5	2E-03	-1.4	3E-03	-1.4
ENSMUST00000034428	Gabarapl2	-1.5	5E-03	-1.4	6E-03	-1.4
NM_027297	Prpf4	-1.5	6E-04	-1.4	1E-03	-1.4
NM_009128	Scd2	-1.7	1E-04	-1.2	3E-02	-1.4

NM_145223	Alms1	-1.4	2E-03	-1.5	2E-04	-1.4
NM_001142916	Plod2	-1.5	3E-03	-1.4	7E-03	-1.4
ENSMUST00000054956	Stambpl1	-1.4	3E-03	-1.5	1E-03	-1.4
NM_145928	Tspan14	-1.5	7E-03	-1.4	2E-02	-1.4
NM_145441	Ubxn2a	-1.4	2E-02	-1.5	7E-03	-1.4
NM_028243	Prcp	-1.6	2E-04	-1.3	5E-03	-1.4
NM_030704	Hspb8	-1.5	6E-04	-1.4	8E-04	-1.4
NM_011169	Prlr	-1.5	2E-03	-1.4	1E-02	-1.4
NM_028108	Naa50	-1.4	9E-04	-1.4	1E-03	-1.4
ENSMUST00000029459	Gdap2	-1.5	4E-03	-1.4	2E-02	-1.4
ENSMUST00000088786	Sri	-1.5	2E-03	-1.4	6E-03	-1.4
NM_009700	Aqp4	-1.4	4E-02	-1.5	3E-02	-1.4
NM_019579	Mpp5	-1.5	4E-03	-1.4	1E-02	-1.4
NM_021509	Moxd1	-1.5	2E-03	-1.4	8E-03	-1.4
NM_020282	Nqo2	-1.5	6E-03	-1.3	4E-02	-1.4
NM_008428	Kcnj8	-1.5	2E-03	-1.4	7E-03	-1.4
NM_001110311	Snx12	-1.3	3E-02	-1.6	2E-03	-1.4
NR_033744	Cbwd1	-1.4	1E-03	-1.5	3E-04	-1.4
NM_027193	Dph5	-1.4	3E-02	-1.5	2E-02	-1.4
NM_029896	Wdr82	-1.6	2E-03	-1.3	2E-02	-1.4
NM_029145	Rnase10	-1.4	4E-03	-1.5	3E-03	-1.4
NM_008029	Flt4	-1.4	1E-02	-1.5	4E-03	-1.4
NM_021547	Stard3	-1.3	3E-02	-1.5	5E-03	-1.4
NM_173769	Zfp641	-1.3	4E-02	-1.5	7E-03	-1.4
AK046316	B230369F24Rik	-1.5	3E-03	-1.4	4E-03	-1.4
NM_013875	Pde7b	-1.5	8E-04	-1.4	1E-03	-1.4
NM_019793	Tspan3	-1.3	3E-07	-1.5	2E-08	-1.4
NM_018818	Chm	-1.5	1E-04	-1.4	3E-04	-1.4
NM_138303	Yipf2	-1.4	2E-02	-1.5	1E-02	-1.4
NM_026775	Tmed10	-1.4	2E-04	-1.4	1E-04	-1.4
NR_029726	Mirlet7a-2	-1.4	2E-02	-1.5	1E-02	-1.4
NM_009847	Cd2ap	-1.5	1E-03	-1.4	2E-03	-1.4
NM_177736	Lrrc61	-1.5	1E-03	-1.4	2E-03	-1.4
NM_008425	Kcnj2	-1.5	8E-04	-1.4	3E-03	-1.4
ENSMUST00000108551	Gp1ba	-1.4	3E-02	-1.4	3E-02	-1.4
NM_001085522	Gm13251	-1.6	5E-04	-1.2	4E-02	-1.4
NM_027667	Arhgap19	-1.4	4E-02	-1.5	2E-02	-1.4
ENSMUST00000027157	Rpe	-1.4	8E-03	-1.4	1E-02	-1.4
NM_172465	Zdhhc9	-1.3	1E-02	-1.5	1E-03	-1.4
NM_027468	Cpm	-1.5	1E-02	-1.4	2E-02	-1.4
NM_172643	Zbtb41	-1.4	2E-03	-1.5	7E-04	-1.4
ENSMUST00000064667	Rap1b	-1.5	4E-03	-1.4	1E-02	-1.4
NM_001255977	Gm4345	-1.5	6E-03	-1.3	4E-02	-1.4
NM_001111110	Cmah	-1.6	6E-06	-1.3	3E-04	-1.4
NM_130856	Krtap16-8	-1.4	4E-02	-1.4	5E-02	-1.4
NM_146103	Tmem185b	-1.5	2E-03	-1.4	6E-03	-1.4

ENSMUST00000171839	Zfp192	-1.3	2E-02	-1.6	1E-03	-1.4
NM_011596	Atp6v0a2	-1.5	1E-03	-1.3	9E-03	-1.4
NM_080446	Helb	-1.4	1E-02	-1.5	3E-03	-1.4
ENSMUST00000071230	Eif2s1	-1.4	7E-04	-1.5	3E-04	-1.4
NM_025972	Naaa	-1.5	1E-03	-1.4	5E-03	-1.4
ENSMUST00000110551	Sord	-1.4	6E-03	-1.5	1E-03	-1.4
ENSMUST00000105693	Nmnat1	-1.4	1E-02	-1.4	9E-03	-1.4
NM_026246	Mrpl49	-1.5	3E-03	-1.3	2E-02	-1.4
NM_001159942	Plekhg1	-1.4	2E-02	-1.4	2E-02	-1.4
NM_001165941	Nsun6	-1.5	7E-03	-1.4	2E-02	-1.4
NM_145484	Zfp758	-1.5	3E-02	-1.4	5E-02	-1.4
NM_008098	Mtpn	-1.4	3E-06	-1.4	6E-06	-1.4
NM_001177653	Hist1h2be	-1.5	5E-03	-1.4	1E-02	-1.4
NM_007923	Elk4	-1.4	3E-02	-1.5	2E-02	-1.4
NM_001195421	Hist1h4m	-1.4	9E-03	-1.4	1E-02	-1.4
NM_175111	Hspbap1	-1.5	1E-02	-1.4	2E-02	-1.4
NM_028288	Cul4b	-1.5	6E-03	-1.4	2E-02	-1.4
NM_175639	Wdr43	-1.5	1E-02	-1.4	2E-02	-1.4
NM_198007	Ascc3	-1.5	3E-05	-1.4	6E-05	-1.4
NR_040446	C920021L13Rik	-1.5	3E-03	-1.3	1E-02	-1.4
NM_001171052	Mta3	-1.3	2E-02	-1.6	1E-03	-1.4
NM_176972	Usp37	-1.4	1E-02	-1.5	5E-03	-1.4
NM_011884	Rngtt	-1.4	8E-04	-1.4	1E-03	-1.4
NM_147153	Vps39	-1.5	4E-04	-1.4	8E-04	-1.4
NM_019410	Pfn2	-1.5	1E-02	-1.4	3E-02	-1.4
NM_178599	Commd8	-1.4	3E-04	-1.4	2E-04	-1.4
NM_001081280	Nlrc3	-1.5	7E-03	-1.3	2E-02	-1.4
NM_013469	Anxa11	-1.3	1E-03	-1.5	2E-04	-1.4
NM_030165	Csgalnact2	-1.4	6E-05	-1.4	7E-05	-1.4
NM_146319	Olfr727	-1.4	2E-02	-1.4	1E-02	-1.4
NM_146224	Zfp280d	-1.5	5E-04	-1.4	1E-03	-1.4
NM_011818	Gmcl1	-1.3	1E-03	-1.5	2E-04	-1.4
NM_028315	Dis3	-1.4	8E-03	-1.4	1E-02	-1.4
NM_177381	Cog3	-1.4	3E-03	-1.4	2E-03	-1.4
NM_027514	Pvr	-1.3	5E-02	-1.5	1E-02	-1.4
NM_023824	Paqr4	-1.3	1E-02	-1.5	2E-03	-1.4
NM_028221	Fam192a	-1.4	3E-03	-1.4	3E-03	-1.4
BC064812	Zfp433	-1.4	4E-03	-1.4	7E-03	-1.4
ENSMUST00000034388	Vps4a	-1.3	5E-03	-1.5	1E-03	-1.4
ENSMUST00000031069	Sepsecs	-1.5	3E-03	-1.4	8E-03	-1.4
NM_027236	Eif1ad	-1.4	1E-03	-1.4	6E-04	-1.4
NM_027127	Gpx8	-1.6	2E-04	-1.3	9E-03	-1.4
ENSMUST00000052164	Ppyr1	-1.6	2E-04	-1.2	1E-02	-1.4
NM_010344	Gsr	-1.5	4E-04	-1.3	3E-03	-1.4
NM_026343	Stx17	-1.3	3E-02	-1.5	5E-03	-1.4
NM_027722	Nudt4	-1.5	3E-04	-1.3	4E-03	-1.4

NR_028385	Gm10190	-1.3	4E-02	-1.5	6E-03	-1.4
NM_023844	Jam2	-1.5	6E-04	-1.3	8E-03	-1.4
ENSMUST00000034405	Mre11a	-1.3	1E-02	-1.5	9E-04	-1.4
NM_181410	Gtf2h3	-1.4	1E-02	-1.4	2E-02	-1.4
NM_027231	Polr2f	-1.4	5E-03	-1.4	1E-02	-1.4
NM_001005423	Mreg	-1.4	3E-03	-1.4	3E-03	-1.4
NM_144802	Hnrpll	-1.3	1E-03	-1.5	1E-04	-1.4
NM_021512	Nup160	-1.4	4E-03	-1.4	3E-03	-1.4
ENSMUST00000148960	Myl12a	-1.4	1E-02	-1.5	7E-03	-1.4
NM_183140	Zfp691	-1.3	2E-03	-1.6	4E-05	-1.4
ENSMUST00000109249	Sulf2	-1.3	9E-03	-1.5	2E-03	-1.4
NM_145452	Rasa1	-1.5	3E-05	-1.4	1E-04	-1.4
BC099503	Gm527	-1.5	2E-02	-1.4	4E-02	-1.4
NM_026816	Gtf2f2	-1.5	9E-03	-1.3	3E-02	-1.4
NM_173783	Mageb18	-1.5	7E-03	-1.3	3E-02	-1.4
NM_011399	Slc25a17	-1.4	4E-03	-1.4	8E-03	-1.4
ENSMUST00000029477	Slc25a24	-1.5	4E-03	-1.4	1E-02	-1.4
NM_001159612	Lrrc57	-1.4	4E-03	-1.4	5E-03	-1.4
NR_029649	Mir298	-1.5	1E-02	-1.4	2E-02	-1.4
ENSMUST00000026859	Mfsd8	-1.4	6E-03	-1.5	2E-03	-1.4
NM_001033222	Pdzd8	-1.3	9E-03	-1.5	2E-03	-1.4
ENSMUST00000102946	Exoc2	-1.5	6E-05	-1.4	2E-04	-1.4
NR_037998	D030040B21Rik	-1.6	2E-03	-1.3	5E-02	-1.4
NR_015529	R74862	-1.4	2E-02	-1.4	2E-02	-1.4
NM_033618	Supt16h	-1.5	4E-03	-1.3	3E-02	-1.4
NM_178617	Necab1	-1.4	2E-02	-1.4	1E-02	-1.4
NM_009592	Abcb7	-1.3	1E-02	-1.5	2E-03	-1.4
NM_148924	Zfp263	-1.5	1E-02	-1.3	4E-02	-1.4
NM_008885	Pmp22	-1.5	2E-03	-1.3	3E-02	-1.4
NM_001113474	Lair1	-1.5	1E-02	-1.4	4E-02	-1.4
NM_175451	Ckap4	-1.3	4E-02	-1.5	1E-02	-1.4
NM_001034851	Fam134b	-1.5	4E-04	-1.3	4E-03	-1.4
ENSMUST00000049005	Bmpr1a	-1.6	3E-04	-1.2	2E-02	-1.4
ENSMUST00000059667	Hpcal4	-1.5	4E-03	-1.3	1E-02	-1.4
NM_026046	Zfp329	-1.4	1E-03	-1.4	3E-03	-1.4
NM_080795	Lnx2	-1.4	2E-03	-1.5	6E-04	-1.4
NM_144915	Daglb	-1.3	3E-02	-1.5	5E-03	-1.4
ENSMUST00000120461	Lman1	-1.4	7E-04	-1.4	5E-04	-1.4
NM_026144	Dhdds	-1.4	4E-03	-1.4	3E-03	-1.4
NM_026283	Samd8	-1.3	8E-04	-1.5	3E-05	-1.4
NM_019698	Aldh18a1	-1.5	5E-04	-1.3	4E-03	-1.4
NM_011758	Zfp39	-1.6	2E-04	-1.2	1E-02	-1.4
NM_023224	Cblc	-1.4	2E-02	-1.4	1E-02	-1.4
NM_001033550	Lrrc8b	-1.3	1E-02	-1.5	1E-03	-1.4
NM_001164071	Tank	-1.3	5E-02	-1.5	4E-03	-1.4
NM_008468	Kpna6	-1.4	8E-04	-1.4	5E-04	-1.4

NM_001177843	Frmd4a	-1.4	3E-02	-1.4	1E-02	-1.4
NM_198607	4930572J05Rik	-1.4	3E-02	-1.4	2E-02	-1.4
NM_145480	Rfc4	-1.5	7E-04	-1.3	8E-03	-1.4
NM_001199494	Ei24	-1.4	7E-04	-1.4	8E-04	-1.4
NM_145707	Obox3	-1.5	9E-03	-1.3	4E-02	-1.4
NM_026576	Etaa1	-1.4	3E-02	-1.4	2E-02	-1.4
NM_001009947	Dock11	-1.5	1E-03	-1.3	2E-02	-1.4
NM_145629	Pls3	-1.5	3E-03	-1.3	1E-02	-1.4
NM_009127	Scd1	-1.5	1E-03	-1.3	8E-03	-1.4
NM_009418	Tpp2	-1.3	2E-04	-1.5	5E-05	-1.4
ENSMUST00000162554	Ttc26	-1.4	4E-03	-1.4	2E-03	-1.4
NM_027402	Fndc5	-1.5	1E-03	-1.3	4E-03	-1.4
NM_009682	Ap3s2	-1.3	2E-02	-1.5	5E-03	-1.4
NM_029956	Mmab	-1.2	3E-02	-1.6	9E-04	-1.4
ENSMUST00000153360	Ino80c	-1.4	3E-02	-1.4	1E-02	-1.4
ENSMUST00000035930	Zfp800	-1.4	6E-03	-1.4	9E-03	-1.4
NM_172967	4930503L19Rik	-1.4	6E-03	-1.4	5E-03	-1.4
ENSMUST00000076313	Prpf40a	-1.4	2E-03	-1.4	2E-03	-1.4
ENSMUST00000047404	Dync1li1	-1.3	1E-03	-1.5	2E-04	-1.4
ENSMUST00000028081	Plxdc2	-1.4	3E-02	-1.4	4E-02	-1.4
NM_013768	Prmt5	-1.5	1E-03	-1.3	2E-02	-1.4
NM_025887	Rab5a	-1.6	2E-04	-1.2	2E-02	-1.4
NM_009070	Rnps1	-1.5	1E-03	-1.3	3E-02	-1.4
BC023359	3632451O06Rik	-1.5	1E-03	-1.2	3E-02	-1.4
ENSMUST00000174545	Xpo4	-1.4	2E-03	-1.4	2E-03	-1.4
NM_153538	Zcchc6	-1.4	4E-03	-1.4	3E-03	-1.4
ENSMUST00000045368	BC026590	-1.3	1E-02	-1.5	1E-03	-1.4
NR_045578	St7l	-1.5	2E-03	-1.3	2E-02	-1.4
NM_152816	Dnm1l	-1.3	2E-03	-1.5	3E-04	-1.4
NM_029976	Cdkn2aipnl	-1.4	5E-04	-1.4	7E-04	-1.4
ENSMUST00000041516	Epgn	-1.3	1E-02	-1.4	4E-03	-1.4
ENSMUST00000066632	Angel2	-1.4	8E-03	-1.4	6E-03	-1.4
ENSMUST00000029451	Tspan2	-1.3	1E-02	-1.5	2E-03	-1.4
NM_001099637	Cep170	-1.4	3E-02	-1.4	3E-02	-1.4
ENSMUST00000114848	Taf11	-1.6	2E-05	-1.2	2E-03	-1.4
NM_001146707	Nap1l1	-1.5	2E-03	-1.3	1E-02	-1.4
NM_001081105	Rhoh	-1.5	3E-03	-1.3	4E-02	-1.4
NM_001177602	Ak4	-1.3	1E-02	-1.5	1E-03	-1.4
NM_001164362	Cep55	-1.4	3E-02	-1.4	1E-02	-1.4
ENSMUST00000114666	Atp6v1a	-1.4	2E-03	-1.4	2E-03	-1.4
NM_177429	Ofd1	-1.4	8E-03	-1.4	2E-02	-1.4
NM_008740	Nsf	-1.3	5E-02	-1.5	7E-03	-1.4
NM_172988	Fbxl4	-1.2	5E-02	-1.6	1E-03	-1.4
NM_011826	Hax1	-1.4	1E-03	-1.4	8E-04	-1.4
NM_001142647	Tmem194b	-1.4	2E-02	-1.4	2E-02	-1.4
NM_028173	Tram1	-1.4	6E-04	-1.3	2E-03	-1.4

NM_001081274	Pgd	-1.3	1E-02	-1.5	9E-04	-1.4
NM_134052	Adi1	-1.3	3E-02	-1.5	7E-03	-1.4
NM_007466	Api5	-1.4	6E-03	-1.4	6E-03	-1.4
ENSMUST00000058577	2810046L04Rik	-1.3	7E-03	-1.5	7E-04	-1.4
NM_026604	Fam135a	-1.4	2E-03	-1.4	4E-03	-1.4
NM_173014	Lpcat2	-1.4	8E-03	-1.4	9E-03	-1.4
ENSMUST00000151952	Mgat4a	-1.4	1E-02	-1.4	1E-02	-1.4
NM_008565	Mcm4	-1.5	1E-04	-1.3	2E-03	-1.4
ENSMUST00000047131	Ipo4	-1.4	8E-04	-1.4	1E-03	-1.4
NM_145609	8430410K20Rik	-1.4	4E-03	-1.4	3E-03	-1.4
NM_011960	Parg	-1.4	5E-03	-1.3	2E-02	-1.4
NM_029909	C330018D20Rik	-1.4	2E-03	-1.4	9E-04	-1.4
NM_144551	Trib2	-1.3	1E-03	-1.4	3E-04	-1.4
ENSMUST00000111246	Accs	-1.4	2E-02	-1.3	5E-02	-1.4
NM_001003934	Rtn3	-1.3	7E-04	-1.4	2E-04	-1.4
NM_010901	Nfatc3	-1.4	4E-04	-1.4	2E-04	-1.4
NM_029278	Nop14	-1.3	2E-03	-1.5	2E-04	-1.4
NM_001037917	Gm6377	-1.4	2E-02	-1.3	4E-02	-1.4
BC043120	Fam76b	-1.4	1E-03	-1.4	3E-03	-1.4
NM_008602	Pias2	-1.3	9E-03	-1.5	1E-03	-1.4
NM_153055	Sec63	-1.4	5E-04	-1.4	9E-04	-1.4
NM_008999	Rab23	-1.4	2E-02	-1.3	5E-02	-1.4
ENSMUST00000050735	Zfp260	-1.5	5E-03	-1.3	2E-02	-1.4
NM_026173	Poc5	-1.3	4E-02	-1.5	8E-03	-1.4
NM_183250	Ccdc72	-1.5	8E-04	-1.2	3E-02	-1.4
NM_207705	Elmo2	-1.4	4E-03	-1.4	4E-03	-1.4
NM_001252292	Mest	-1.3	2E-02	-1.4	4E-03	-1.4
NM_207221	Jmjd1c	-1.4	5E-03	-1.4	6E-03	-1.4
NM_177103	Senp5	-1.3	2E-03	-1.4	6E-04	-1.4
NM_026062	Fam69a	-1.4	2E-02	-1.4	2E-02	-1.4
NM_027192	Ttl	-1.4	1E-02	-1.4	8E-03	-1.4
NM_001081080	Phf3	-1.3	2E-02	-1.5	4E-03	-1.4
ENSMUST00000028386	Nckap1	-1.5	8E-04	-1.2	2E-02	-1.4
NM_001039482	Klhl20	-1.4	1E-02	-1.4	1E-02	-1.4
NM_026276	Aasdhppt	-1.3	4E-02	-1.5	9E-03	-1.4
NM_175317	Eftud1	-1.2	4E-02	-1.6	8E-04	-1.4
NM_053089	Naa15	-1.4	3E-03	-1.4	4E-03	-1.4
NM_001081286	Fat1	-1.3	2E-02	-1.5	4E-03	-1.4
NM_027159	Ccdc115	-1.4	9E-03	-1.4	2E-02	-1.4
NM_027156	Ddx51	-1.4	3E-03	-1.4	2E-03	-1.4
ENSMUST00000102585	Fgf11	-1.3	1E-02	-1.4	4E-03	-1.4
NM_023179	Atp6v1g2	-1.3	2E-02	-1.4	6E-03	-1.4
NM_172587	Cdc14b	-1.3	3E-02	-1.5	2E-03	-1.4
NM_021310	Jmy	-1.3	1E-02	-1.5	7E-04	-1.4
NM_001024950	Zfp563	-1.5	1E-02	-1.3	4E-02	-1.4
ENSMUST00000116375	Cstf1	-1.2	1E-02	-1.5	2E-04	-1.4

NM_178746	Slc38a9	-1.3	2E-02	-1.4	6E-03	-1.4
NM_025707	Klhl28	-1.3	8E-04	-1.4	2E-04	-1.4
NR_003555	Vmn2r29	-1.4	4E-03	-1.4	2E-03	-1.4
ENSMUST00000121979	Cep135	-1.3	2E-02	-1.4	8E-03	-1.4
NM_080575	Acss1	-1.4	1E-02	-1.4	1E-02	-1.4
NM_001163263	Rnf20	-1.3	3E-02	-1.5	8E-03	-1.4
ENSMUST00000019064	Cxcl16	-1.5	3E-03	-1.3	3E-02	-1.4
NM_198899	Uggt1	-1.4	1E-04	-1.3	7E-04	-1.4
BC059213	4732418C07Rik	-1.3	1E-03	-1.4	3E-04	-1.4
NM_001039521	Rrn3	-1.3	4E-02	-1.5	9E-03	-1.4
NM_026579	D10Wsu102e	-1.3	4E-02	-1.4	2E-02	-1.4
NM_023680	Tnfrsf22	-1.5	9E-03	-1.3	4E-02	-1.4
ENSMUST00000029881	Mmp16	-1.3	4E-02	-1.4	2E-02	-1.4
NM_009033	Rbmxl1	-1.4	8E-04	-1.3	3E-03	-1.4
NM_001177833	Smox	-1.4	2E-02	-1.4	1E-02	-1.4
BC048158	Fam177a	-1.5	4E-03	-1.3	2E-02	-1.4
NM_194339	Bms1	-1.3	4E-04	-1.5	4E-05	-1.4
NM_134163	Mbnl3	-1.5	8E-04	-1.3	1E-02	-1.4
NM_019678	Tfg	-1.4	2E-03	-1.4	2E-03	-1.4
NM_172836	9930021J03Rik	-1.3	5E-02	-1.5	3E-03	-1.4
NM_030241	Setd8	-1.4	9E-03	-1.4	7E-03	-1.4
ENSMUST00000072080	Lrrc40	-1.3	2E-03	-1.4	4E-04	-1.4
NM_020270	Scamp5	-1.3	3E-03	-1.4	1E-03	-1.4
NM_212484	Cnot6	-1.3	3E-02	-1.5	2E-03	-1.4
ENSMUST00000034879	Hmg20a	-1.3	2E-02	-1.5	4E-03	-1.4
NM_146892	Olfr1223	-1.3	3E-03	-1.5	2E-04	-1.4
NM_001195097	3425401B19Rik	-1.4	2E-03	-1.3	1E-02	-1.4
NM_030261	Sesn3	-1.3	2E-03	-1.5	5E-05	-1.4
NM_025546	Rsl1d1	-1.5	7E-04	-1.3	9E-03	-1.4
ENSMUST00000061568	Slc36a4	-1.4	1E-02	-1.4	9E-03	-1.4
NM_172593	Mier3	-1.2	3E-02	-1.5	6E-04	-1.4
NM_011749	Zfp148	-1.5	4E-03	-1.3	3E-02	-1.4
NM_028151	Skiv2l2	-1.3	8E-04	-1.4	3E-04	-1.4
ENSMUST00000030427	Tceb3	-1.5	1E-04	-1.3	4E-03	-1.4
NM_001110826	Ddx6	-1.5	5E-05	-1.3	9E-04	-1.4
BC125500	E130311K13Rik	-1.4	9E-03	-1.4	9E-03	-1.4
NM_019480	Ebag9	-1.3	4E-02	-1.5	5E-03	-1.4
NM_019961	Pex3	-1.4	4E-04	-1.4	2E-04	-1.4
NR_037271	Mir3088	-1.5	4E-03	-1.3	4E-02	-1.4
NM_001110017	Dzip3	-1.4	5E-04	-1.4	6E-04	-1.4
NM_033560	Vps37a	-1.4	2E-03	-1.4	1E-03	-1.4
NM_144835	Heatr1	-1.5	6E-05	-1.2	8E-03	-1.4
NM_001195031	Pag1	-1.3	2E-02	-1.4	6E-03	-1.4
NM_009811	Casp6	-1.4	3E-02	-1.3	5E-02	-1.4
NM_145972	BC027231	-1.4	9E-03	-1.3	3E-02	-1.4
NM_011273	Xpr1	-1.4	4E-03	-1.3	2E-02	-1.4

ENSMUST00000091752	Hist2h3b	-1.3	2E-02	-1.4	1E-02	-1.4
NM_011889	Septin3	-1.5	6E-04	-1.3	6E-03	-1.4
NM_134138	Psmg2	-1.4	4E-03	-1.3	7E-03	-1.4
NM_177208	Dopey1	-1.5	8E-04	-1.3	2E-02	-1.4
NM_176916	Pld5	-1.4	3E-02	-1.4	3E-02	-1.4
NM_199025	Zbtb26	-1.5	4E-03	-1.3	3E-02	-1.4
NM_001081152	Npat	-1.3	2E-04	-1.4	5E-05	-1.4
ENSMUST00000039990	Leprel1	-1.3	1E-03	-1.4	5E-04	-1.4
NM_207272	Tdpoz4	-1.4	4E-02	-1.4	4E-02	-1.4
NM_173734	Tmem87a	-1.4	9E-03	-1.4	9E-03	-1.4
NM_001081265	Heatr2	-1.4	1E-02	-1.4	1E-02	-1.4
NM_007950	Ereg	-1.3	5E-02	-1.4	1E-02	-1.4
NM_026532	Nutf2	-1.4	2E-03	-1.4	2E-03	-1.4
NM_001081073	Cep76	-1.3	8E-03	-1.5	5E-04	-1.4
ENSMUST00000111117	Cdc42bpa	-1.3	2E-02	-1.4	1E-02	-1.4
NM_019914	Mllt11	-1.4	5E-03	-1.3	2E-02	-1.4
NM_177632	Fam43a	-1.4	2E-02	-1.4	2E-02	-1.4
NM_026504	Coq5	-1.2	8E-03	-1.5	1E-04	-1.4
ENSMUST00000135431	Lrrc4c	-1.4	5E-03	-1.3	1E-02	-1.4
NM_007561	Bmpr2	-1.3	2E-02	-1.5	9E-04	-1.4
NM_001163314	Pgap1	-1.2	1E-02	-1.5	3E-04	-1.4
BC031465	Fam13b	-1.4	3E-02	-1.4	3E-02	-1.4
NM_028355	Tmem48	-1.3	5E-02	-1.4	2E-02	-1.4
NM_011135	Cnot7	-1.3	6E-03	-1.4	1E-03	-1.4
NM_133705	Pycr2	-1.4	1E-02	-1.4	7E-03	-1.4
NM_001080814	Fat3	-1.3	3E-02	-1.4	1E-02	-1.4
NM_016856	Cpsf2	-1.5	4E-04	-1.2	9E-03	-1.4
NM_011186	Psmb5	-1.4	3E-04	-1.3	6E-04	-1.4
NM_175386	Lhfp	-1.5	8E-03	-1.3	4E-02	-1.4
NM_178907	Mapkapk3	-1.5	4E-04	-1.2	1E-02	-1.4
NM_173037	Tmco7	-1.3	2E-02	-1.4	5E-03	-1.4
NM_001190409	Magt1	-1.3	4E-02	-1.5	4E-03	-1.4
NM_009302	Swap70	-1.3	3E-02	-1.5	5E-03	-1.4
ENSMUST00000098873	Gabpb2	-1.4	3E-02	-1.4	4E-02	-1.4
ENSMUST00000021356	1110034A24Rik	-1.4	2E-02	-1.4	2E-02	-1.4
NM_016741	Scarb1	-1.3	4E-02	-1.4	1E-02	-1.4
NM_145952	Tbc1d12	-1.3	2E-02	-1.5	2E-03	-1.4
NM_021326	Rbak	-1.4	2E-02	-1.4	3E-02	-1.4
NM_148673	Slu7	-1.5	1E-03	-1.2	5E-02	-1.4
NM_011804	Creg1	-1.4	3E-03	-1.3	5E-03	-1.4
NM_133229	Ripply3	-1.4	2E-02	-1.4	1E-02	-1.4
ENSMUST00000054344	Eogt	-1.4	8E-03	-1.3	2E-02	-1.4
ENSMUST00000037585	Fam116a	-1.3	3E-03	-1.4	2E-03	-1.4
ENSMUST00000067298	Mrps21	-1.3	1E-02	-1.4	3E-03	-1.4
NM_133927	Itfg2	-1.4	5E-03	-1.3	1E-02	-1.4
NM_001040690	Rap1gds1	-1.4	6E-03	-1.3	1E-02	-1.4

ENSMUST00000028062	Vim	-1.4	2E-02	-1.3	3E-02	-1.4
NM_020042	Mocs1	-1.3	3E-02	-1.4	6E-03	-1.4
NM_026116	Bbs2	-1.3	4E-03	-1.4	4E-04	-1.4
NM_030690	Rai14	-1.2	5E-02	-1.5	3E-03	-1.4
NM_009386	Tjp1	-1.3	2E-02	-1.5	1E-03	-1.4
ENSMUST00000091903	Sh3bp5	-1.5	3E-03	-1.3	2E-02	-1.4
NM_029492	Zdhhc20	-1.4	1E-02	-1.3	2E-02	-1.4
ENSMUST00000044681	Arl6ip5	-1.4	1E-03	-1.3	4E-03	-1.4
NM_001081198	Tmem182	-1.3	1E-03	-1.4	3E-04	-1.4
NM_031181	Siglece	-1.4	6E-03	-1.4	8E-03	-1.4
NM_019868	Hnrnph2	-1.3	3E-03	-1.4	7E-04	-1.4
NM_013749	Tnfrsf12a	-1.3	3E-02	-1.4	9E-03	-1.4
ENSMUST00000150990	Hdhd2	-1.3	2E-02	-1.4	1E-02	-1.4
NM_028982	8430419L09Rik	-1.5	1E-03	-1.3	1E-02	-1.4
NM_177694	Ano5	-1.4	1E-02	-1.3	3E-02	-1.4
NM_010431	Hif1a	-1.4	1E-02	-1.3	2E-02	-1.4
NM_029545	6530401N04Rik	-1.5	8E-04	-1.2	3E-02	-1.4
ENSMUST00000027688	Rassf5	-1.4	4E-03	-1.3	2E-02	-1.4
NM_145599	Tmem184c	-1.5	7E-04	-1.2	1E-02	-1.4
NM_207246	Rasgrp3	-1.4	8E-04	-1.3	3E-03	-1.4
NM_025645	Snrnp40	-1.3	9E-03	-1.4	3E-03	-1.4
ENSMUST00000090178	Dnajb14	-1.3	1E-02	-1.4	7E-03	-1.4
NM_053086	Nolc1	-1.2	3E-02	-1.5	1E-03	-1.4
ENSMUST00000018625	Appbp2	-1.3	1E-04	-1.4	6E-05	-1.4
NM_178696	Slc25a44	-1.4	1E-02	-1.3	3E-02	-1.4
NR_039590	Mir5128	-1.3	2E-02	-1.4	3E-03	-1.4
NM_145483	Zfp160	-1.4	5E-03	-1.3	7E-03	-1.4
BC064033	Fam180a	-1.4	3E-02	-1.4	3E-02	-1.4
NM_007550	Blm	-1.5	2E-03	-1.2	4E-02	-1.4
NM_001110015	Wdr36	-1.4	2E-04	-1.3	3E-04	-1.4
NM_026217	Atg12	-1.4	1E-03	-1.3	8E-03	-1.4
NM_029929	Vps33a	-1.3	1E-02	-1.4	1E-03	-1.4
NM_009851	Cd44	-1.4	9E-03	-1.3	2E-02	-1.4
NM_011739	Ywhaq	-1.4	2E-03	-1.3	3E-03	-1.4
ENSMUST00000012540	Nanog	-1.3	2E-02	-1.4	2E-03	-1.4
NM_172616	C330027C09Rik	-1.4	2E-02	-1.3	3E-02	-1.4
NM_026067	Eri1	-1.4	1E-02	-1.3	2E-02	-1.4
ENSMUST00000022429	Arf4	-1.4	4E-04	-1.3	1E-03	-1.4
NM_018732	Scn3a	-1.4	3E-03	-1.3	2E-02	-1.4
ENSMUST00000166032	Tdrkh	-1.2	7E-03	-1.5	2E-04	-1.4
NM_026482	Atp2b1	-1.3	4E-04	-1.4	3E-04	-1.4
NM_009158	Mapk10	-1.4	2E-03	-1.3	3E-03	-1.4
ENSMUST00000007602	M6pr	-1.3	2E-04	-1.4	2E-04	-1.4
NM_153532	Zfp280c	-1.2	1E-02	-1.5	5E-04	-1.4
NM_007566	Birc6	-1.4	9E-05	-1.3	7E-04	-1.4
BC044749	2310057M21Rik	-1.3	7E-03	-1.4	7E-04	-1.4

NM_030096	Ddx52	-1.4	4E-04	-1.3	6E-04	-1.4
NM_145466	A2ld1	-1.3	9E-03	-1.4	4E-03	-1.4
NM_172591	Fcho2	-1.4	5E-03	-1.3	2E-02	-1.4
NM_009361	Tfdp1	-1.4	1E-03	-1.3	2E-03	-1.4
NM_013719	Eif2ak4	-1.3	3E-02	-1.4	4E-03	-1.4
NM_177338	Hmbox1	-1.4	3E-03	-1.3	4E-03	-1.4
ENSMUST00000105520	Enpp1	-1.3	2E-02	-1.4	2E-03	-1.4
NM_001205043	Jarid2	-1.3	9E-03	-1.4	5E-03	-1.4
NM_001033242	Cln5	-1.3	2E-03	-1.4	3E-04	-1.4
NM_027769	Cpne3	-1.5	3E-03	-1.2	4E-02	-1.4
NM_001081118	Phrf1	-1.3	3E-02	-1.4	1E-02	-1.4
NM_001033213	Ttc7b	-1.2	4E-03	-1.5	2E-04	-1.4
NM_133994	Gstt3	-1.5	4E-04	-1.2	7E-03	-1.4
NM_001167750	Ccdc132	-1.3	2E-02	-1.4	2E-02	-1.4
NM_021504	Ngly1	-1.3	2E-02	-1.4	2E-02	-1.4
NM_011764	Zfp90	-1.3	3E-02	-1.4	8E-03	-1.4
ENSMUST00000103005	Shroom4	-1.4	1E-02	-1.3	1E-02	-1.4
NM_009316	Map3k7	-1.3	3E-03	-1.4	2E-03	-1.4
NM_023311	Yipf5	-1.4	1E-04	-1.3	3E-04	-1.3
NM_008105	Gcnt2	-1.3	3E-02	-1.4	3E-02	-1.3
ENSMUST00000028059	Rsu1	-1.5	2E-03	-1.2	3E-02	-1.3
ENSMUST00000027444	Pde6d	-1.3	8E-03	-1.4	7E-03	-1.3
NM_080554	Psmd5	-1.4	7E-04	-1.3	7E-04	-1.3
NM_001013389	Mrs2	-1.3	5E-03	-1.4	4E-03	-1.3
NM_026277	Nob1	-1.4	2E-03	-1.3	3E-03	-1.3
ENSMUST00000046520	Fkbp14	-1.3	4E-02	-1.4	8E-03	-1.3
NM_201360	Cyp2d12	-1.4	2E-02	-1.3	4E-02	-1.3
NM_028228	Pinx1	-1.4	1E-02	-1.3	2E-02	-1.3
NM_008862	Pkia	-1.3	3E-02	-1.4	1E-02	-1.3
NM_001082485	Zfp266	-1.4	9E-03	-1.3	2E-02	-1.3
NM_016802	Rhoa	-1.3	2E-05	-1.4	4E-06	-1.3
NM_133227	Nup155	-1.4	2E-02	-1.3	2E-02	-1.3
NM_146096	Fam108b	-1.2	3E-03	-1.4	1E-04	-1.3
NM_001142731	Kctd1	-1.3	3E-02	-1.4	1E-02	-1.3
NM_029788	Rnft1	-1.4	9E-03	-1.3	3E-02	-1.3
NM_025483	Senp7	-1.4	3E-02	-1.3	4E-02	-1.3
NM_145076	Trim24	-1.4	8E-03	-1.3	1E-02	-1.3
ENSMUST00000007708	Ppp2r1a	-1.3	3E-03	-1.4	2E-04	-1.3
ENSMUST00000048774	Copg2	-1.3	4E-03	-1.4	3E-03	-1.3
NM_001081251	Pbrm1	-1.3	7E-05	-1.4	4E-05	-1.3
ENSMUST00000160285	Wdr63	-1.3	5E-02	-1.4	2E-02	-1.3
ENSMUST00000052368	Kdm6a	-1.3	4E-04	-1.4	7E-05	-1.3
NM_001004154	Rragb	-1.3	4E-02	-1.4	2E-02	-1.3
NM_013814	Galnt1	-1.4	2E-05	-1.2	4E-04	-1.3
ENSMUST00000063191	Serpinb9	-1.3	3E-02	-1.4	3E-03	-1.3
ENSMUST00000020113	Poc1b	-1.3	3E-02	-1.4	5E-03	-1.3

NM_174875	Atg4a	-1.4	3E-03	-1.3	6E-03	-1.3
ENSMUST00000071650	Idua	-1.4	6E-03	-1.3	9E-03	-1.3
NM_172585	Larp4b	-1.4	5E-04	-1.3	6E-04	-1.3
ENSMUST00000029569	Slc35a3	-1.3	3E-03	-1.4	6E-04	-1.3
ENSMUST00000077273	Ext1	-1.2	2E-02	-1.5	6E-04	-1.3
NM_013652	Ccl4	-1.4	7E-03	-1.3	5E-02	-1.3
NM_015806	Mapk6	-1.2	2E-02	-1.5	9E-04	-1.3
NM_145458	Pxk	-1.4	2E-03	-1.3	5E-03	-1.3
NM_019498	Olfm1	-1.3	2E-02	-1.4	5E-03	-1.3
NM_011132	Pole	-1.4	6E-03	-1.3	2E-02	-1.3
ENSMUST00000085573	Traf5	-1.4	4E-03	-1.3	3E-02	-1.3
ENSMUST00000114025	Prrg1	-1.4	4E-03	-1.2	5E-02	-1.3
NM_001103157	Steap2	-1.4	8E-03	-1.3	1E-02	-1.3
NM_027113	Wdr5b	-1.3	3E-02	-1.3	4E-02	-1.3
NM_145552	Gnl2	-1.3	2E-02	-1.3	2E-02	-1.3
NM_025356	Ube2d3	-1.3	5E-04	-1.3	5E-04	-1.3
ENSMUST00000125716	Gnao1	-1.4	9E-04	-1.3	3E-03	-1.3
NM_024452	Luzp1	-1.4	6E-04	-1.3	4E-03	-1.3
NM_018742	Bet1l	-1.4	4E-03	-1.2	4E-02	-1.3
NM_178647	Cggbp1	-1.3	2E-02	-1.4	1E-02	-1.3
NM_001081113	Ipo8	-1.3	2E-02	-1.4	9E-03	-1.3
NM_001162921	Zc3h12c	-1.2	4E-02	-1.4	3E-03	-1.3
ENSMUST00000081339	Rrp1b	-1.3	2E-02	-1.4	6E-03	-1.3
NM_015751	Abce1	-1.4	1E-03	-1.3	7E-03	-1.3
ENSMUST00000032735	Mphosph10	-1.4	1E-02	-1.3	5E-02	-1.3
NM_029942	Prelid2	-1.4	2E-02	-1.3	3E-02	-1.3
NM_001085495	Arfgef2	-1.4	6E-03	-1.3	3E-02	-1.3
ENSMUST00000000153	Gna12	-1.3	2E-03	-1.3	2E-03	-1.3
NM_009829	Ccnd2	-1.3	4E-02	-1.4	2E-02	-1.3
NM_027045	Gcap14	-1.4	4E-04	-1.3	3E-03	-1.3
NM_013841	Vps45	-1.4	1E-02	-1.3	3E-02	-1.3
NR_027008	Gt(ROSA)26Sor	-1.4	3E-02	-1.3	4E-02	-1.3
NM_009461	Ubr1	-1.3	3E-03	-1.4	1E-03	-1.3
NM_001081300	Tshz1	-1.2	1E-02	-1.4	9E-04	-1.3
NM_173757	Mrps27	-1.3	3E-02	-1.4	2E-02	-1.3
NM_008466	Kpna3	-1.4	3E-03	-1.3	8E-03	-1.3
NM_138667	Tab2	-1.3	2E-04	-1.4	6E-05	-1.3
NM_138685	Wfdc15b	-1.3	2E-02	-1.3	3E-02	-1.3
NM_025791	Tmem223	-1.3	2E-02	-1.3	2E-02	-1.3
NM_010608	Kcnk3	-1.3	3E-03	-1.4	4E-04	-1.3
NM_177670	Tmem69	-1.3	4E-02	-1.4	7E-03	-1.3
NM_175094	Pdhx	-1.3	1E-02	-1.4	2E-03	-1.3
NM_009677	Ap1g1	-1.3	2E-04	-1.3	3E-04	-1.3
ENSMUST00000015100	Ppp1cb	-1.3	3E-03	-1.4	3E-04	-1.3
NM_133906	Zkscan1	-1.3	3E-02	-1.3	4E-02	-1.3
NR_028109	Fam172a	-1.4	4E-03	-1.3	2E-02	-1.3

ENSMUST00000027997	Rgs5	-1.5	1E-03	-1.2	4E-02	-1.3
NM_001033270	Slc4a7	-1.3	2E-03	-1.3	1E-03	-1.3
ENSMUST00000006435	Atp6v1b2	-1.3	8E-04	-1.4	2E-04	-1.3
ENSMUST00000154617	Rps6kb1	-1.3	2E-02	-1.4	5E-03	-1.3
ENSMUST00000063084	Xbp1	-1.4	4E-03	-1.3	2E-02	-1.3
NM_001033194	Gtf3c3	-1.3	6E-03	-1.3	9E-03	-1.3
NM_001159965	Ralgps2	-1.3	7E-04	-1.3	6E-04	-1.3
NM_026040	Srfbp1	-1.4	1E-02	-1.3	3E-02	-1.3
NM_001042438	Zhx1	-1.4	2E-03	-1.2	3E-02	-1.3
NM_175025	Atp2c1	-1.3	6E-04	-1.3	8E-04	-1.3
NM_001146060	Als2cl	-1.4	1E-02	-1.3	3E-02	-1.3
NM_001004190	Zfp560	-1.5	2E-03	-1.2	4E-02	-1.3
NM_026876	1190005F20Rik	-1.3	4E-03	-1.4	1E-03	-1.3
ENSMUST00000108156	Bbs7	-1.3	2E-02	-1.3	2E-02	-1.3
NM_172926	Snx14	-1.4	3E-03	-1.3	1E-02	-1.3
NM_008566	Mcm5	-1.3	3E-02	-1.4	2E-02	-1.3
NM_029094	Pik3cb	-1.2	6E-03	-1.4	3E-04	-1.3
NM_030262	Pofut2	-1.2	3E-03	-1.4	2E-04	-1.3
NM_025999	Rnf141	-1.4	2E-05	-1.3	2E-04	-1.3
NM_008133	Glud1	-1.3	3E-03	-1.4	7E-04	-1.3
ENSMUST00000040881	Cluap1	-1.4	3E-03	-1.3	2E-02	-1.3
NM_197997	4930422G04Rik	-1.3	3E-02	-1.4	7E-03	-1.3
NM_146208	Neil3	-1.4	7E-03	-1.3	4E-02	-1.3
NM_025932	Syap1	-1.3	2E-04	-1.3	2E-04	-1.3
NM_134188	Acot2	-1.4	1E-02	-1.3	2E-02	-1.3
NM_011930	Clcn7	-1.3	3E-02	-1.4	5E-03	-1.3
NM_007499	Atm	-1.3	4E-05	-1.3	2E-05	-1.3
NM_018819	Brp44l	-1.3	4E-03	-1.4	8E-04	-1.3
ENSMUST00000057438	Vcpip1	-1.3	4E-03	-1.4	2E-03	-1.3
NM_172695	Plaa	-1.3	1E-02	-1.4	8E-03	-1.3
NM_024190	Chmp1b	-1.3	2E-02	-1.4	6E-03	-1.3
ENSMUST00000057792	Pon2	-1.3	2E-02	-1.3	1E-02	-1.3
NM_008943	Psen1	-1.4	2E-03	-1.3	1E-02	-1.3
NM_028136	Dhx36	-1.3	3E-04	-1.3	6E-04	-1.3
NM_025443	Pno1	-1.4	3E-03	-1.3	2E-02	-1.3
NM_001146311	Cln3	-1.3	3E-02	-1.3	3E-02	-1.3
NR_046281	4930502E09Rik	-1.3	8E-03	-1.3	1E-02	-1.3
ENSMUST00000021920	Sptlc1	-1.3	6E-03	-1.3	4E-03	-1.3
NM_178870	Hs3st3a1	-1.4	1E-02	-1.3	3E-02	-1.3
NM_145475	Cerk	-1.4	1E-02	-1.3	2E-02	-1.3
NM_017472	Snx3	-1.2	4E-02	-1.4	2E-03	-1.3
NM_172937	Shprh	-1.3	9E-04	-1.4	2E-04	-1.3
NM_134076	Abhd4	-1.4	1E-03	-1.2	2E-02	-1.3
NM_001081056	Xpot	-1.4	2E-03	-1.3	6E-03	-1.3
NM_029653	Dapk1	-1.3	2E-02	-1.3	1E-02	-1.3
NM_146005	Ank3	-1.2	3E-02	-1.4	6E-03	-1.3

NM_018749	Eif3d	-1.4	5E-03	-1.3	1E-02	-1.3
NM_025942	Ola1	-1.3	2E-02	-1.4	6E-03	-1.3
ENSMUST00000110109	Plcb4	-1.3	4E-03	-1.3	4E-03	-1.3
ENSMUST00000092984	Myh10	-1.3	2E-04	-1.3	1E-04	-1.3
ENSMUST00000002790	Cse1l	-1.3	1E-03	-1.4	3E-04	-1.3
ENSMUST00000031680	Ing3	-1.3	1E-02	-1.3	9E-03	-1.3
NM_001077495	Pik3r1	-1.3	7E-03	-1.4	1E-03	-1.3
ENSMUST00000043760	Mvk	-1.3	1E-02	-1.4	4E-03	-1.3
NM_207671	Zfp318	-1.3	3E-02	-1.4	1E-02	-1.3
NM_178407	Arap2	-1.4	4E-03	-1.3	1E-02	-1.3
NM_025481	Smurf2	-1.3	2E-04	-1.3	3E-04	-1.3
NM_201519	Map4k5	-1.3	2E-03	-1.3	4E-03	-1.3
NM_011240	Ranbp2	-1.3	1E-03	-1.4	3E-04	-1.3
NM_011916	Xrn1	-1.3	5E-03	-1.3	3E-03	-1.3
NM_026647	Zdhhc21	-1.3	7E-03	-1.3	5E-03	-1.3
ENSMUST00000165563	Micu1	-1.4	1E-03	-1.2	4E-02	-1.3
NM_175647	Dmrta1	-1.2	1E-02	-1.4	5E-04	-1.3
NM_022979	Nup98	-1.3	2E-03	-1.4	1E-03	-1.3
NM_001122982	Ccnc	-1.3	5E-02	-1.4	2E-02	-1.3
NM_027513	Nup205	-1.3	9E-03	-1.3	7E-03	-1.3
NM_144917	Elmod3	-1.3	3E-02	-1.3	2E-02	-1.3
NM_144826	Utp6	-1.4	2E-03	-1.2	4E-02	-1.3
NM_178377	Commd10	-1.3	3E-02	-1.4	1E-02	-1.3
NM_033320	Glce	-1.4	2E-03	-1.3	1E-02	-1.3
NM_029884	Hgsnat	-1.4	3E-04	-1.3	1E-03	-1.3
ENSMUST00000081636	Prkag3	-1.3	3E-02	-1.3	2E-02	-1.3
ENSMUST00000152954	Hars2	-1.2	4E-02	-1.4	2E-03	-1.3
NM_001029934	Usp32	-1.3	8E-03	-1.3	4E-03	-1.3
NM_152812	Otud6b	-1.4	2E-03	-1.3	6E-03	-1.3
NM_001162532	Fam174b	-1.3	5E-02	-1.4	2E-02	-1.3
NM_026390	Ubxn4	-1.4	5E-04	-1.2	2E-02	-1.3
ENSMUST00000065562	Socs4	-1.3	6E-03	-1.3	7E-03	-1.3
ENSMUST00000053771	Phkb	-1.3	2E-02	-1.3	2E-02	-1.3
ENSMUST00000046306	Ikzf5	-1.3	2E-02	-1.4	5E-03	-1.3
NM_001252494	Rapgef6	-1.4	1E-02	-1.3	4E-02	-1.3
NM_001110013	Tmtc3	-1.4	3E-04	-1.2	3E-03	-1.3
NM_172307	Mbtps2	-1.3	8E-03	-1.3	9E-03	-1.3
NM_177705	4932411N23Rik	-1.3	3E-02	-1.3	5E-02	-1.3
NM_001114332	Slc16a10	-1.2	4E-02	-1.4	6E-03	-1.3
NM_177003	9630033F20Rik	-1.3	1E-02	-1.3	7E-03	-1.3
ENSMUST00000128909	Tmem56	-1.3	3E-02	-1.3	3E-02	-1.3
ENSMUST00000033609	Cstf2	-1.4	2E-02	-1.3	4E-02	-1.3
NM_138677	Edem1	-1.3	3E-02	-1.3	4E-02	-1.3
ENSMUST00000059899	Mmgt1	-1.3	4E-03	-1.4	8E-04	-1.3
ENSMUST00000084838	Cd47	-1.4	1E-03	-1.2	1E-02	-1.3
NM_207214	Exoc5	-1.3	3E-02	-1.4	8E-03	-1.3

NM_027270	Exoc1	-1.3	5E-02	-1.4	2E-02	-1.3
NM_001081058	Cdk13	-1.3	2E-02	-1.3	9E-03	-1.3
NM_152895	Kdm5b	-1.3	1E-02	-1.4	3E-03	-1.3
NM_175312	B630005N14Rik	-1.3	3E-03	-1.3	2E-03	-1.3
NM_001160214	Smek1	-1.3	1E-02	-1.4	5E-03	-1.3
NM_009057	Slc50a1	-1.4	3E-04	-1.2	4E-03	-1.3
NM_027892	Ppp1r12a	-1.2	8E-03	-1.4	4E-04	-1.3
ENSMUST00000070607	Haus6	-1.3	5E-02	-1.3	4E-02	-1.3
NM_027315	Ube2q1	-1.4	7E-06	-1.3	7E-05	-1.3
AK042831	A730028G07Rik	-1.3	2E-02	-1.4	5E-03	-1.3
NM_025447	Dimt1	-1.3	1E-02	-1.3	2E-02	-1.3
ENSMUST00000112925	Ift122	-1.4	5E-04	-1.2	1E-02	-1.3
ENSMUST00000028619	Hsd17b12	-1.3	1E-02	-1.3	1E-02	-1.3
NM_020588	Tmem183a	-1.3	7E-03	-1.3	1E-02	-1.3
NM_177767	Ogfod1	-1.3	3E-02	-1.3	2E-02	-1.3
NM_026401	Mrp63	-1.3	2E-02	-1.3	9E-03	-1.3
NM_178912	Fancm	-1.4	8E-03	-1.3	3E-02	-1.3
NM_026879	Chmp2b	-1.3	3E-03	-1.3	7E-03	-1.3
NM_177692	Bloc1s3	-1.4	2E-03	-1.3	1E-02	-1.3
NM_011740	Ywhaz	-1.3	6E-03	-1.4	2E-03	-1.3
BC016084	BC031181	-1.4	3E-03	-1.2	2E-02	-1.3
NM_028887	Smchd1	-1.3	2E-04	-1.3	5E-04	-1.3
NM_028994	Pck2	-1.3	4E-03	-1.3	2E-03	-1.3
NM_178380	Dhx38	-1.3	4E-03	-1.3	5E-03	-1.3
ENSMUST00000095070	Dnajc25	-1.3	2E-02	-1.4	4E-03	-1.3
ENSMUST00000031693	Spam1	-1.3	8E-03	-1.3	1E-02	-1.3
NM_025872	Golt1b	-1.3	4E-03	-1.3	2E-03	-1.3
NM_001026214	Entpd5	-1.3	1E-02	-1.3	6E-03	-1.3
NM_011416	Smarca2	-1.3	2E-03	-1.3	2E-03	-1.3
NM_011030	P4ha1	-1.4	2E-03	-1.2	2E-02	-1.3
NM_019588	Plce1	-1.3	4E-04	-1.3	2E-04	-1.3
NM_007769	Dmbt1	-1.3	4E-02	-1.3	2E-02	-1.3
NM_153164	Cnot1	-1.3	3E-04	-1.3	9E-05	-1.3
NM_027462	Wars2	-1.4	8E-04	-1.3	4E-03	-1.3
NM_001004362	2610008E11Rik	-1.3	4E-02	-1.3	5E-02	-1.3
ENSMUST00000109790	Asxl1	-1.4	1E-02	-1.3	4E-02	-1.3
BC031379	2610034B18Rik	-1.4	5E-04	-1.2	4E-03	-1.3
ENSMUST00000112194	Trappc2	-1.3	5E-02	-1.3	4E-02	-1.3
NM_026364	Prpsap1	-1.3	5E-02	-1.3	3E-02	-1.3
ENSMUST00000035635	Bmp2k	-1.3	1E-02	-1.3	9E-03	-1.3
NM_029090	Nat15	-1.3	1E-02	-1.3	8E-03	-1.3
NM_007983	Faf1	-1.3	2E-03	-1.3	1E-03	-1.3
NM_019920	Lamtor3	-1.3	9E-03	-1.3	7E-03	-1.3
NM_022989	Arl6ip6	-1.3	2E-02	-1.4	4E-03	-1.3
NM_026028	Ccdc77	-1.3	9E-03	-1.3	2E-02	-1.3
NM_146671	Olfr822	-1.3	4E-02	-1.3	3E-02	-1.3

NM_001025392	Bclaf1	-1.3	1E-02	-1.3	1E-02	-1.3
NM_007690	Chd1	-1.2	6E-03	-1.4	1E-03	-1.3
BC034664	Lyrm4	-1.3	5E-04	-1.3	8E-04	-1.3
NM_172595	Arl15	-1.3	3E-02	-1.3	2E-02	-1.3
NM_019658	Shoc2	-1.3	5E-04	-1.3	3E-04	-1.3
ENSMUST00000028384	Dusp19	-1.3	4E-02	-1.3	4E-02	-1.3
ENSMUST00000150434	Brwd3	-1.3	1E-02	-1.3	2E-02	-1.3
NM_177240	D030016E14Rik	-1.2	8E-03	-1.4	8E-04	-1.3
ENSMUST00000004054	Kpna1	-1.3	4E-02	-1.3	2E-02	-1.3
NM_021393	Cobra1	-1.2	4E-02	-1.4	6E-03	-1.3
NM_146054	Fermt2	-1.3	5E-03	-1.3	2E-03	-1.3
NM_026234	Pigm	-1.2	3E-02	-1.4	4E-03	-1.3
NM_007481	Arf6	-1.2	5E-03	-1.4	5E-04	-1.3
ENSMUST00000063318	Slc16a7	-1.3	2E-02	-1.3	3E-02	-1.3
NM_027375	Gcc2	-1.4	5E-03	-1.2	4E-02	-1.3
NM_172393	Aim1	-1.3	5E-02	-1.3	2E-02	-1.3
ENSMUST00000044359	Ankrd44	-1.3	3E-02	-1.3	9E-03	-1.3
NM_029010	Glb1l	-1.3	4E-02	-1.3	3E-02	-1.3
NM_133702	Nol11	-1.3	4E-03	-1.3	1E-02	-1.3
NM_011734	Siae	-1.4	6E-04	-1.2	8E-03	-1.3
NM_172666	Agps	-1.3	8E-03	-1.3	9E-03	-1.3
NM_053084	Trim32	-1.2	4E-02	-1.4	5E-03	-1.3
NM_126166	Tlr3	-1.3	7E-03	-1.3	3E-02	-1.3
NM_001112714	Ralgapa1	-1.3	4E-03	-1.3	9E-04	-1.3
NM_008640	Laptm4a	-1.2	9E-04	-1.4	5E-05	-1.3
NM_026033	Gatad1	-1.3	2E-02	-1.3	2E-02	-1.3
NM_001163759	Dhx57	-1.2	6E-03	-1.4	9E-04	-1.3
NM_173444	Nbeal1	-1.3	2E-04	-1.3	3E-04	-1.3
NM_145962	Pank3	-1.3	1E-02	-1.3	3E-02	-1.3
ENSMUST00000080215	Chst15	-1.3	5E-03	-1.3	4E-03	-1.3
NR_040680	LOC100503496	-1.3	1E-02	-1.3	2E-02	-1.3
ENSMUST00000029358	Nmd3	-1.3	4E-02	-1.3	3E-02	-1.3
NM_008142	Gnb1	-1.3	8E-03	-1.3	1E-02	-1.3
NM_001039089	Sel1l	-1.3	4E-04	-1.3	1E-04	-1.3
NM_027642	Phf6	-1.3	3E-03	-1.3	7E-03	-1.3
NM_008564	Mcm2	-1.3	3E-03	-1.3	3E-03	-1.3
NM_008702	Nlk	-1.2	3E-02	-1.3	1E-02	-1.3
NM_026396	Brix1	-1.3	2E-02	-1.3	2E-02	-1.3
NM_009680	Ap3b1	-1.3	3E-04	-1.3	2E-03	-1.3
NM_001081008	Taf1	-1.2	8E-03	-1.4	4E-04	-1.3
NR_046064	Gm10373	-1.4	9E-04	-1.2	1E-02	-1.3
NM_009535	Yes1	-1.3	5E-02	-1.3	2E-02	-1.3
NM_018759	Zfp326	-1.3	4E-02	-1.3	2E-02	-1.3
XM_994335	Gm8978	-1.3	3E-02	-1.3	4E-02	-1.3
ENSMUST00000164568	Pde8a	-1.3	1E-02	-1.3	6E-03	-1.3
NM_001081161	Fam171a1	-1.3	2E-02	-1.3	5E-02	-1.3

ENSMUST00000002848	Grin2d	-1.2	8E-03	-1.4	7E-04	-1.3
NM_026307	Cuta	-1.3	4E-02	-1.3	4E-02	-1.3
NM_011101	Prkca	-1.3	4E-02	-1.3	2E-02	-1.3
NM_026453	Mak16	-1.3	1E-02	-1.2	3E-02	-1.3
NM_018851	Samhd1	-1.3	2E-02	-1.3	2E-02	-1.3
NM_008914	Ppp3cb	-1.2	1E-02	-1.4	9E-04	-1.3
NM_001128096	Atp13a3	-1.4	5E-03	-1.2	2E-02	-1.3
ENSMUST00000081933	Dtx3l	-1.3	1E-02	-1.2	4E-02	-1.3
NM_019553	Ddx21	-1.3	6E-03	-1.2	2E-02	-1.3
ENSMUST00000029770	Abcd3	-1.2	2E-02	-1.4	1E-03	-1.3
NM_011112	Papola	-1.3	2E-03	-1.2	1E-02	-1.3
NM_025938	Rpp14	-1.3	9E-03	-1.2	3E-02	-1.3
NM_001007573	Maneal	-1.2	1E-02	-1.4	9E-04	-1.3
NM_173185	Csnk1g1	-1.3	5E-03	-1.3	1E-02	-1.3
NM_007971	Ezh2	-1.3	7E-03	-1.3	2E-03	-1.3
ENSMUST00000059319	Tmem17	-1.3	2E-02	-1.3	3E-02	-1.3
NM_133766	Efr3a	-1.3	4E-03	-1.3	1E-02	-1.3
ENSMUST00000112990	Mid2	-1.3	4E-04	-1.3	1E-04	-1.3
ENSMUST00000030138	Nol6	-1.3	6E-03	-1.3	2E-03	-1.3
ENSMUST00000026710	Usp16	-1.3	2E-04	-1.3	3E-04	-1.3
ENSMUST00000022451	Capn7	-1.4	3E-03	-1.2	2E-02	-1.3
NM_001199177	Opa1	-1.2	3E-02	-1.4	5E-03	-1.3
NM_027423	Polr3b	-1.3	3E-03	-1.3	2E-03	-1.3
NM_008379	Kpnb1	-1.3	7E-03	-1.3	2E-03	-1.3
NM_015827	Copb2	-1.3	5E-03	-1.3	1E-02	-1.3
XM_003688829	LOC100862151	-1.3	7E-03	-1.3	6E-03	-1.3
ENSMUST00000102956	Slmap	-1.3	6E-03	-1.3	5E-03	-1.3
NM_010820	Mpdz	-1.3	4E-05	-1.3	2E-04	-1.3
NM_023662	Pcm1	-1.3	2E-03	-1.3	1E-03	-1.3
NM_030886	Ankrd17	-1.3	1E-04	-1.3	1E-04	-1.3
ENSMUST00000039247	Dsc2	-1.2	2E-02	-1.3	4E-03	-1.3
NM_024185	Fam188a	-1.3	2E-02	-1.3	7E-03	-1.3
ENSMUST00000079996	Zfand2a	-1.3	4E-02	-1.3	3E-02	-1.3
NM_012032	Serinc3	-1.3	2E-03	-1.3	8E-04	-1.3
ENSMUST00000122424	Fam20b	-1.3	7E-03	-1.3	4E-03	-1.3
ENSMUST00000054442	N6amt1	-1.3	3E-02	-1.3	4E-02	-1.3
ENSMUST00000002418	2310045N01Rik	-1.2	4E-03	-1.4	2E-04	-1.3
NM_030711	Erap1	-1.3	2E-03	-1.3	2E-03	-1.3
NM_007668	Cdk5	-1.2	2E-02	-1.3	4E-03	-1.3
ENSMUST00000000776	Tubgcp3	-1.3	8E-03	-1.3	1E-02	-1.3
NM_199016	Enpp4	-1.3	2E-02	-1.3	2E-02	-1.3
ENSMUST00000035766	Wdr44	-1.3	2E-02	-1.3	4E-02	-1.3
NM_177151	Vps13b	-1.3	5E-03	-1.3	1E-02	-1.3
NM_026181	Gpatch1	-1.4	4E-03	-1.2	3E-02	-1.3
NM_024459	Ppp3r1	-1.3	2E-03	-1.3	4E-03	-1.3
NM_178246	Smg5	-1.2	4E-03	-1.3	1E-03	-1.3

NM_001163635	Tnks2	-1.3	6E-03	-1.3	4E-03	-1.3
NM_001161362	Ppp2r3a	-1.2	2E-02	-1.3	7E-03	-1.3
ENSMUST00000033910	Leprotl1	-1.2	3E-02	-1.4	4E-03	-1.3
NM_133807	Lrrc59	-1.3	5E-03	-1.3	5E-03	-1.3
NM_009806	Cask	-1.3	2E-02	-1.3	1E-02	-1.3
NM_134015	Fbxw11	-1.2	3E-02	-1.3	8E-03	-1.3
NR_027488	Senp2	-1.3	2E-02	-1.3	2E-02	-1.3
NM_001193303	Lims1	-1.3	1E-02	-1.3	9E-03	-1.3
NM_009443	Tgoln1	-1.3	2E-04	-1.2	2E-03	-1.3
NM_010918	Nktr	-1.2	4E-02	-1.3	6E-03	-1.3
NM_029988	Pigh	-1.3	2E-02	-1.2	5E-02	-1.3
NM_177608	Secisbp2l	-1.3	3E-02	-1.3	2E-02	-1.3
NM_001004164	Gnptab	-1.2	5E-02	-1.4	5E-03	-1.3
ENSMUST00000111665	Tmx2	-1.3	2E-02	-1.3	1E-02	-1.3
NM_144870	Ndufs8	-1.3	1E-02	-1.3	2E-02	-1.3
NM_175375	Ankhd1	-1.3	7E-04	-1.3	7E-04	-1.3
ENSMUST00000140299	Tmem5	-1.3	3E-02	-1.3	3E-02	-1.3
NM_001164677	Pdcd6ip	-1.2	4E-03	-1.3	8E-04	-1.3
ENSMUST00000108277	Tnfaip1	-1.3	1E-02	-1.3	9E-03	-1.3
NM_001205361	Dcun1d1	-1.3	1E-02	-1.2	4E-02	-1.3
NM_019816	Aatf	-1.3	4E-03	-1.2	9E-03	-1.3
NM_029478	Vmp1	-1.2	5E-02	-1.3	1E-02	-1.3
NM_001163026	Dnajc13	-1.3	4E-03	-1.3	7E-03	-1.3
ENSMUST00000127247	Tmem229a	-1.3	9E-03	-1.3	4E-03	-1.3
ENSMUST00000041374	Manea	-1.3	1E-04	-1.3	4E-05	-1.3
NM_011278	Rnf4	-1.3	6E-04	-1.2	8E-03	-1.3
NM_177780	Dock5	-1.2	2E-03	-1.3	4E-04	-1.3
NM_001166416	Med23	-1.3	1E-02	-1.2	3E-02	-1.3
NM_001114119	Qrich1	-1.3	5E-04	-1.2	3E-03	-1.3
NM_172285	Plcg2	-1.2	2E-02	-1.3	6E-03	-1.3
NR_030523	Mir652	-1.3	4E-02	-1.3	3E-02	-1.3
NM_001024926	Cyb5d2	-1.3	2E-02	-1.3	1E-02	-1.3
NM_024244	Fam13c	-1.3	6E-03	-1.3	1E-02	-1.3
NM_026574	Ino80	-1.2	4E-02	-1.3	6E-03	-1.3
NM_025965	Ssr1	-1.3	2E-03	-1.2	2E-02	-1.3
NM_001042523	Txnrd1	-1.3	4E-02	-1.3	3E-02	-1.3
NR_033173	Krit1	-1.3	9E-04	-1.3	1E-03	-1.3
NM_011585	Tia1	-1.2	3E-02	-1.3	4E-03	-1.3
NM_029320	Pibf1	-1.3	1E-02	-1.2	4E-02	-1.3
NM_147005	Olfr395	-1.2	3E-02	-1.3	1E-02	-1.3
ENSMUST00000028251	Rbm18	-1.3	6E-03	-1.2	2E-02	-1.3
NM_173363	Eif5	-1.3	8E-04	-1.2	9E-03	-1.3
ENSMUST00000107545	Med1	-1.2	7E-03	-1.3	1E-03	-1.3
NM_199196	Suz12	-1.3	5E-02	-1.3	4E-02	-1.3
NM_001024952	Rc3h1	-1.3	3E-03	-1.3	4E-03	-1.3
NM_029074	Tmem188	-1.3	3E-02	-1.3	2E-02	-1.3

NM_026417	Yipf4	-1.3	1E-02	-1.3	2E-02	-1.3
NM_175353	Exoc6	-1.3	1E-03	-1.2	7E-03	-1.3
NM_144861	Rprd1a	-1.3	3E-03	-1.2	6E-03	-1.3
NM_027807	Cul5	-1.2	3E-02	-1.3	2E-02	-1.3
NM_001253391	Mtfr1	-1.3	9E-03	-1.2	4E-02	-1.3
NM_008246	Hiat1	-1.3	2E-03	-1.2	1E-02	-1.3
NM_001013770	Lipo1	-1.2	9E-03	-1.3	2E-03	-1.3
NM_001013370	Sesn1	-1.2	5E-02	-1.3	2E-02	-1.3
NM_172771	Dmxl2	-1.3	3E-04	-1.2	1E-03	-1.3
ENSMUST00000016639	5033414D02Rik	-1.3	4E-02	-1.3	3E-02	-1.3
NM_011462	Spin1	-1.3	5E-03	-1.3	7E-03	-1.3
ENSMUST00000077642	Kifap3	-1.2	3E-03	-1.3	1E-03	-1.3
NM_001039483	Tmco1	-1.3	6E-03	-1.2	2E-02	-1.3
NM_172310	Tarsl2	-1.2	1E-02	-1.3	3E-03	-1.3
NM_026353	Slc48a1	-1.3	3E-03	-1.2	2E-02	-1.3
NM_146067	Cpped1	-1.3	4E-02	-1.3	4E-02	-1.3
BC051430	B230219D22Rik	-1.2	3E-04	-1.3	1E-04	-1.3
NM_144842	Zmym5	-1.3	1E-04	-1.3	2E-04	-1.3
NM_173762	Cenpe	-1.3	6E-03	-1.2	2E-02	-1.3
ENSMUST00000028610	Cat	-1.3	4E-04	-1.3	3E-04	-1.3
NM_153391	Wdr19	-1.2	2E-02	-1.3	6E-03	-1.3
NM_153542	Lrrc20	-1.3	1E-02	-1.3	7E-03	-1.3
ENSMUST00000022720	Fbxl3	-1.2	1E-03	-1.3	2E-04	-1.3
NM_025824	Bzw1	-1.3	9E-05	-1.2	3E-04	-1.3
NM_001081371	Dmxl1	-1.3	4E-04	-1.3	4E-04	-1.3
NM_133247	Usp33	-1.3	4E-04	-1.2	1E-03	-1.3
NM_001111028	Kctd9	-1.2	3E-02	-1.3	2E-02	-1.3
NM_011715	Wdr1	-1.3	1E-02	-1.3	9E-03	-1.3
ENSMUST00000161859	Rnf6	-1.3	5E-03	-1.3	6E-03	-1.3
NM_027860	0610010F05Rik	-1.2	3E-03	-1.3	2E-03	-1.3
NM_029334	Zc3h14	-1.3	1E-02	-1.2	3E-02	-1.3
NM_013761	Srr	-1.3	2E-02	-1.2	4E-02	-1.3
NM_133242	Rbm39	-1.2	1E-02	-1.3	5E-03	-1.3
ENSMUST00000067327	Cdkn1b	-1.3	2E-03	-1.2	4E-03	-1.3
NM_134058	Pelo	-1.3	3E-02	-1.3	3E-02	-1.3
NM_001114385	Chrdl1	-1.2	2E-02	-1.3	3E-03	-1.3
NM_031251	Ctns	-1.3	4E-02	-1.3	3E-02	-1.3
ENSMUST00000097680	Rab18	-1.2	2E-02	-1.3	6E-03	-1.3
ENSMUST00000004784	Cnn2	-1.2	4E-02	-1.3	2E-02	-1.3
NR_024093	U05342	-1.3	4E-03	-1.2	9E-03	-1.3
NM_053098	Lmod2	-1.2	8E-03	-1.3	5E-03	-1.3
ENSMUST00000102944	Creb3	-1.3	2E-03	-1.2	1E-02	-1.3
NM_029572	Erp44	-1.2	2E-02	-1.3	7E-03	-1.3
NM_021500	Maea	-1.2	2E-02	-1.3	6E-03	-1.3
NM_025517	Rtcd1	-1.3	3E-02	-1.3	4E-02	-1.3
ENSMUST00000101506	BC023829	-1.3	1E-02	-1.3	1E-02	-1.3

NR_045306	4931403E22Rik	-1.3	2E-02	-1.2	4E-02	-1.3
NM_016701	Nes	-1.2	5E-02	-1.3	2E-02	-1.3
ENSMUST00000174252	Scyl2	-1.2	2E-02	-1.3	3E-03	-1.3
NM_026375	Ahctf1	-1.2	1E-02	-1.3	6E-03	-1.3
ENSMUST00000159692	Ermp1	-1.3	2E-03	-1.2	2E-02	-1.3
NM_138593	Larp7	-1.3	8E-03	-1.2	3E-02	-1.3
ENSMUST00000163119	Cul3	-1.2	1E-03	-1.3	2E-04	-1.3
NM_027415	Tmem70	-1.2	5E-03	-1.3	3E-03	-1.3
NM_010879	Nck2	-1.3	2E-02	-1.2	3E-02	-1.3
ENSMUST00000103241	Sema6d	-1.3	1E-03	-1.2	7E-03	-1.3
NM_027604	Usp15	-1.3	7E-03	-1.2	4E-02	-1.3
NM_080452	Mrps2	-1.3	2E-02	-1.2	4E-02	-1.3
NM_173764	Tapt1	-1.2	3E-02	-1.3	2E-02	-1.3
NM_177407	Camk2a	-1.3	3E-02	-1.2	5E-02	-1.3
NM_007404	Adam9	-1.3	5E-04	-1.2	3E-03	-1.3
NM_020584	Terf2ip	-1.3	3E-02	-1.2	4E-02	-1.3
NM_016906	Sec61a1	-1.2	4E-02	-1.3	1E-02	-1.3
NM_001177771	Clcc1	-1.3	2E-03	-1.3	2E-03	-1.3
NM_007889	Dvl3	-1.2	5E-02	-1.3	1E-02	-1.3
NM_027355	Rnf168	-1.3	1E-03	-1.2	8E-03	-1.3
ENSMUST00000102800	Gapvd1	-1.3	4E-03	-1.2	8E-03	-1.3
ENSMUST00000102759	Stam2	-1.3	2E-02	-1.2	2E-02	-1.3
NR_030694	1110002L01Rik	-1.3	2E-02	-1.2	3E-02	-1.3
NM_027475	Ecd	-1.2	1E-02	-1.3	6E-03	-1.3
NM_178114	Amigo2	-1.2	3E-02	-1.3	2E-02	-1.3
NM_080788	Ttbk2	-1.3	8E-03	-1.3	8E-03	-1.3
ENSMUST00000151287	Tcp1	-1.3	6E-03	-1.2	3E-02	-1.3
NM_001130408	Arf1	-1.2	9E-03	-1.3	1E-03	-1.3
NM_172513	Fam126b	-1.2	5E-02	-1.3	2E-02	-1.3
ENSMUST00000036570	Appl1	-1.3	3E-03	-1.2	2E-02	-1.3
NM_026120	2410127L17Rik	-1.3	1E-02	-1.2	4E-02	-1.3
NM_028360	Ttc19	-1.2	2E-02	-1.3	4E-03	-1.3
NM_021041	Abcc9	-1.2	2E-03	-1.3	9E-04	-1.2
ENSMUST00000025236	Stard4	-1.3	4E-02	-1.2	5E-02	-1.2
NM_173006	Pon3	-1.2	7E-03	-1.3	5E-03	-1.2
NM_029745	Tbc1d9b	-1.3	2E-02	-1.2	2E-02	-1.2
NM_007451	Slc25a5	-1.2	6E-03	-1.3	2E-03	-1.2
ENSMUST00000018875	Ap2b1	-1.2	5E-03	-1.3	9E-04	-1.2
NM_178699	B930041F14Rik	-1.2	5E-02	-1.3	2E-02	-1.2
NM_146062	Pphln1	-1.2	2E-02	-1.2	2E-02	-1.2
NM_025333	Sdhaf2	-1.2	3E-02	-1.3	2E-02	-1.2
ENSMUST00000041993	Iapp	-1.3	6E-04	-1.2	2E-03	-1.2
NM_173028	Vps13a	-1.2	3E-02	-1.3	2E-02	-1.2
NM_026236	Wdr48	-1.3	3E-03	-1.2	1E-02	-1.2
ENSMUST00000068856	Snupn	-1.2	3E-02	-1.2	3E-02	-1.2
NM_011655	Tubb5	-1.2	5E-02	-1.2	5E-02	-1.2

NM_019491	Rala	-1.2	2E-02	-1.2	2E-02	-1.2
NM_001014973	Snx13	-1.2	2E-02	-1.2	2E-02	-1.2
NM_134114	Sft2d1	-1.3	5E-03	-1.2	9E-03	-1.2
NM_027879	Cdc40	-1.3	3E-04	-1.2	7E-04	-1.2
NM_009289	Slk	-1.2	7E-04	-1.3	1E-04	-1.2
NM_053242	Foxp2	-1.2	9E-03	-1.3	6E-03	-1.2
ENSMUST00000120381	Stt3a	-1.2	2E-02	-1.2	3E-02	-1.2
NM_001081061	Bdp1	-1.2	1E-03	-1.3	6E-04	-1.2
NM_080556	Tm9sf2	-1.2	5E-04	-1.3	1E-04	-1.2
NM_019737	B4galt6	-1.2	4E-02	-1.3	2E-02	-1.2
NM_001198968	Itsn2	-1.3	1E-02	-1.2	3E-02	-1.2
NM_028584	Marveld3	-1.3	1E-02	-1.2	3E-02	-1.2
NM_029665	Ipo11	-1.2	2E-03	-1.3	2E-03	-1.2
NM_172626	Rbm27	-1.3	1E-03	-1.2	5E-03	-1.2
NM_025417	Commd4	-1.3	2E-03	-1.2	8E-03	-1.2
NM_007712	Clk2	-1.2	1E-02	-1.3	3E-03	-1.2
NM_019490	Uso1	-1.2	9E-04	-1.2	1E-03	-1.2
NM_178601	Imp4	-1.2	8E-03	-1.3	5E-03	-1.2
NM_008370	Il5ra	-1.3	3E-02	-1.2	4E-02	-1.2
NM_001039048	Trim63	-1.2	4E-02	-1.2	4E-02	-1.2
NM_134071	Ankrd32	-1.3	1E-02	-1.2	4E-02	-1.2
NM_013718	Trappc3	-1.2	3E-03	-1.3	7E-04	-1.2
NM_007462	Apc	-1.3	1E-02	-1.2	2E-02	-1.2
NM_028719	Cpne4	-1.2	2E-02	-1.2	2E-02	-1.2
NM_030179	Clip4	-1.2	7E-03	-1.3	4E-03	-1.2
ENSMUST00000118592	A630007B06Rik	-1.3	1E-03	-1.2	4E-03	-1.2
NM_029409	Mff	-1.3	3E-02	-1.2	4E-02	-1.2
NM_183046	Kif20b	-1.2	5E-02	-1.3	3E-02	-1.2
NM_026664	Vps53	-1.2	5E-03	-1.2	4E-03	-1.2
ENSMUST00000091731	Vmn1r200	-1.2	2E-02	-1.3	9E-03	-1.2
NM_011816	G3bp2	-1.2	2E-03	-1.2	4E-03	-1.2
NR_033593	4930529C04Rik	-1.2	5E-02	-1.3	3E-02	-1.2
NM_172609	Tomm22	-1.2	2E-02	-1.2	3E-02	-1.2
NM_026696	0610030E20Rik	-1.2	3E-02	-1.2	3E-02	-1.2
ENSMUST00000164744	Uba3	-1.2	4E-02	-1.3	2E-02	-1.2
NM_023647	Nipa2	-1.2	3E-02	-1.3	1E-02	-1.2
NM_011743	Zfp106	-1.3	1E-02	-1.2	3E-02	-1.2
NM_019464	Sh3glb1	-1.3	2E-03	-1.2	8E-03	-1.2
NM_016792	Txnl1	-1.2	2E-03	-1.2	9E-04	-1.2
NM_028074	Ddx42	-1.2	2E-02	-1.2	3E-02	-1.2
NM_001033420	Dock1	-1.2	3E-02	-1.2	3E-02	-1.2
NM_183016	Cdc42bpb	-1.2	5E-02	-1.2	3E-02	-1.2
NM_008569	Anapc1	-1.2	3E-03	-1.2	2E-03	-1.2
NM_025630	Aggf1	-1.3	7E-03	-1.2	2E-02	-1.2
NM_177658	Ralgapb	-1.2	1E-02	-1.2	2E-02	-1.2
NM_021450	Trpm7	-1.2	4E-03	-1.2	6E-03	-1.2

NM_009530	Atrx	-1.2	3E-02	-1.3	2E-02	-1.2
NM_025494	Atp6v1c1	-1.2	1E-02	-1.2	7E-03	-1.2
NM_133907	Ube3c	-1.2	2E-02	-1.2	9E-03	-1.2
NM_026519	Tmem85	-1.3	9E-04	-1.2	3E-03	-1.2
NM_172767	Vwa5a	-1.3	2E-02	-1.2	3E-02	-1.2
NM_145937	Sumf1	-1.2	5E-02	-1.3	2E-02	-1.2
ENSMUST00000002121	Supt6h	-1.2	6E-03	-1.3	2E-03	-1.2
NM_175935	G6pc3	-1.2	3E-02	-1.3	1E-02	-1.2
NM_177633	Ubxn7	-1.2	7E-05	-1.2	4E-05	-1.2
NM_144931	Nae1	-1.3	2E-02	-1.2	4E-02	-1.2
NM_178722	Zfp438	-1.2	3E-02	-1.2	4E-02	-1.2
NM_023290	Mkrn2	-1.2	2E-02	-1.2	3E-02	-1.2
ENSMUST00000096433	Deptor	-1.2	4E-03	-1.2	4E-03	-1.2
NM_001166506	Sec14l1	-1.2	3E-02	-1.2	4E-02	-1.2
ENSMUST00000022901	Rrm2b	-1.2	2E-02	-1.2	9E-03	-1.2
NM_207110	Rnf216	-1.2	2E-03	-1.2	5E-03	-1.2
NM_007551	Cxcr5	-1.2	2E-02	-1.2	2E-02	-1.2
NM_145465	Stk24	-1.2	1E-02	-1.2	2E-02	-1.2
ENSMUST00000130916	Becn1	-1.2	5E-03	-1.2	9E-03	-1.2
ENSMUST00000112932	Zbtb6	-1.2	1E-02	-1.2	6E-03	-1.2
ENSMUST00000060608	Cyp20a1	-1.2	3E-02	-1.2	3E-02	-1.2
NM_011682	Utrn	-1.2	2E-02	-1.2	2E-02	-1.2
NM_025673	Golph3	-1.2	2E-02	-1.2	4E-02	-1.2
NM_023429	Ociad1	-1.2	4E-02	-1.2	4E-02	-1.2
ENSMUST00000110855	Lpgat1	-1.2	7E-03	-1.2	1E-02	-1.2
NM_009163	Sgpl1	-1.2	2E-02	-1.2	8E-03	-1.2
ENSMUST00000101066	Ncor1	-1.2	2E-03	-1.2	2E-03	-1.2
NM_177077	Exoc6b	-1.2	3E-02	-1.2	4E-02	-1.2
NM_133803	Dpp3	-1.2	2E-02	-1.2	1E-02	-1.2
NM_016985	Mtmr1	-1.2	2E-02	-1.2	1E-02	-1.2
NM_020003	0610031J06Rik	-1.2	4E-04	-1.2	4E-04	-1.2
NM_175226	Rnf139	-1.2	3E-03	-1.2	2E-03	-1.2
NM_026281	Tm7sf3	-1.2	3E-02	-1.2	2E-02	-1.2
NM_023743	Eif4enif1	-1.2	4E-02	-1.2	4E-02	-1.2
NM_001081357	Map4k3	-1.2	2E-02	-1.2	3E-02	-1.2
NM_173187	2310035C23Rik	-1.2	1E-02	-1.2	9E-03	-1.2
NM_172280	2210018M11Rik	-1.2	4E-03	-1.2	5E-03	-1.2
NM_139061	Vps54	-1.2	8E-03	-1.2	7E-03	-1.2
NM_033521	Laptm4b	-1.2	3E-02	-1.2	4E-02	-1.2
NM_013830	Prpf4b	-1.2	3E-02	-1.2	3E-02	-1.2

Supplementary Table 4: Protein-coding genes differentially-expressed (either induced or repressed) in *both* S/A^{18} and S/E^{18} peptide-treated animals in a *discordant* manner relative to saline-treated controls. Black type-face denotes genes induced and repressed in S/A^{18} and S/E^{18} peptide-treated animals, respectively whilst blue type-face denotes genes repressed and induced in S/A^{18} and S/E^{18} peptide-treated animals, respectively. Only genes exhibiting a fold-change (FC) $\geq$ 1.2 and p value (P) $\leq$0.05 are considered. In cases of multiple probes mapping to the same transcript, the greatest FC is reported. Genes are ranked according to mean divergence.

		SA v Control		SE v Control		
**RefSeq**	**Gene Symbol**	**FC**	**P**	**FC**	**P**	**Mean divergence**
NM_001013373	Tmprss13	2.3	5E-03	-1.6	5E-02	2.0
ENSMUST00000028166	Nr4a2	1.9	4E-03	-1.9	4E-03	1.9
NM_144515	Zfp52	-1.9	2E-04	1.3	3E-02	1.6
ENSMUST00000060989	Sorl1	1.7	2E-04	-1.3	2E-02	1.5
AY140896	Gm3579	-1.6	3E-03	1.3	2E-02	1.5
ENSMUST00000095529	Fgf14	1.6	4E-03	-1.4	2E-02	1.5
NM_008725	Nppa	1.5	1E-02	-1.4	4E-02	1.4
NM_013512	Epb4.1l4a	1.6	1E-04	-1.3	7E-03	1.4
NM_146106	Lyplal1	1.4	8E-03	-1.5	1E-03	1.4
NM_173447	Ephb1	1.4	1E-02	-1.3	5E-02	1.4
ENSMUST00000023605	Masp1	1.3	2E-02	-1.4	9E-03	1.4
NM_029229	4930557A04Rik	-1.4	4E-02	1.3	5E-02	1.3
NM_013470	Anxa3	-1.4	2E-03	1.3	1E-02	1.3
NM_028222	Cdkn3	-1.3	2E-02	1.3	1E-02	1.3
NM_009834	Ccrn4l	1.3	9E-04	-1.3	2E-03	1.3
NM_145140	Abcc10	1.3	1E-02	-1.3	8E-03	1.3
NM_001083890	4932441B19Rik	-1.4	5E-03	1.2	4E-02	1.3
NM_019514	Astn2	1.3	3E-02	-1.2	5E-02	1.3
ENSMUST00000107636	Prr15l	-1.2	3E-02	1.2	2E-02	1.2
NM_001163456	Cox18	1.2	2E-02	-1.2	3E-02	1.2
AK019797	4930570E03Rik	1.2	5E-03	-1.2	3E-03	1.2

Supplementary Table 5: Protein-coding genes *exclusively* differentially-expressed (either induced (black type-face) or repressed (blue type-face)) in S/A[18] peptide-treated animals relative to saline-treated controls. Only genes exhibiting a fold-change (FC) $\geq$ 1.2 and p value (P) $\leq$0.05 are considered. In cases of multiple probes mapping to the same transcript, the greatest FC is reported. Genes are ranked according to fold-change.

		SA v Control	
**RefSeq/Ensembl**	**Gene Symbol**	**FC**	**P**
NM_207254	Olfr1286	6.1	2E-05
NM_009813	Casq1	3.8	3E-04
BC057661	Rabggtb	3.7	7E-04
NM_133362	Erdr1	3.6	1E-03
NM_001166723	Vmn1r94	3.4	3E-02
XR_141817	LOC100862171	3.2	4E-03
EU052291	Srsy	2.9	5E-02
NM_001114754	Gm6121	2.8	1E-02
NM_023546	Ssty2	2.7	4E-02
NM_001017393	MGC107098	2.6	4E-02
NM_010444	Nr4a1	2.6	1E-03
NR_035454	Mir1933	2.5	5E-02
NM_001123370	9030025P20Rik	2.5	8E-05
AK030561	Fam188b2	2.4	1E-02
ENSMUST00000096148	Gm10338	2.4	5E-03
AF118128	Ddx39b	2.3	3E-03
NM_175490	Gpr75	2.3	2E-02
NM_008720	Npc1	2.3	6E-05
NM_001081212	Irs2	2.2	5E-03
BC100414	LOC100040744	2.2	5E-02
NM_009252	Serpina3n	2.2	1E-02
XR_108081	LOC100504898	2.2	2E-02
NR_046286	H2-T10	2.1	2E-02
XM_003085641	LOC100504515	2.1	3E-02
NR_045946	B020014A21Rik	2.1	3E-02
XR_141834	LOC100862363	2.1	3E-02
NM_010831	Sik1	2.1	3E-03
NM_011215	Ptprn2	2.0	3E-03
NR_040280	Gm12295	2.0	9E-03
NM_025759	Speer4d	2.0	1E-02
ENSMUST00000035061	Ngp	2.0	2E-02
NR_003513	Neat1	2.0	3E-02
NM_146797	Olfr1502	2.0	3E-02
NM_021301	Slc15a2	2.0	5E-02
NM_009026	Rasd1	1.9	2E-02
XM_003086798	Gm6445	1.9	4E-02
ENSMUST00000061829	Cd14	1.9	2E-04
XM_001477670	LOC100041824	1.9	5E-02
XR_107093	Gm19773	1.9	5E-03

NM_010637	Klf4	1.9	7E-03
NM_022326	Ctsm	1.9	2E-02
NR_001585	Speer7-ps1	1.9	2E-02
NM_153142	Slc35e4	1.9	2E-02
NM_146504	Olfr961	1.9	2E-02
NM_013670	Snrpn	1.9	1E-03
AJ005350	Zfp125	1.9	3E-02
NM_027881	Osbpl3	1.9	3E-04
ENSMUST00000096953	Gm10354	1.9	4E-02
ENSMUST00000167923	Gm3696	1.9	6E-03
NM_001256886	Gm3500	1.9	3E-02
NM_146497	Olfr492	1.9	3E-02
NR_035472	Mir1949	1.9	4E-02
NM_001011797	Olfr782	1.9	5E-02
NR_033497	D830015G02Rik	1.9	7E-03
ENSMUST00000030025	Nr4a3	1.8	2E-02
NM_001099333	Gm2863	1.8	9E-03
NR_040417	1810021B22Rik	1.8	5E-03
NM_146357	Olfr168	1.8	2E-02
NR_030607	Mir208b	1.8	2E-02
NM_008631	Mt4	1.8	3E-02
NM_011517	Sycp3	1.8	5E-03
NM_023456	Npy	1.8	1E-02
NM_010651	Klra9	1.8	3E-02
NR_039561	Mir5102	1.8	2E-02
BC048594	BC048594	1.8	1E-02
ENSMUST00000162387	LOC100861621	1.8	2E-03
ENSMUST00000100895	Gm10406	1.8	3E-03
ENSMUST00000117167	S100a9	1.8	3E-02
ENSMUST00000167753	Gm4450	1.8	3E-02
NM_145834	Il17c	1.8	2E-02
NM_010234	Fos	1.8	5E-02
NM_026037	Mboat2	1.8	2E-03
NR_030571	Mir467c	1.7	4E-02
NM_008452	Klf2	1.7	4E-02
NR_030254	Mir486	1.7	3E-02
NR_030570	Mir466h	1.7	2E-02
ENSMUST00000102551	Olfr1289	1.7	2E-02
NM_001024138	Gpr139	1.7	4E-02
NM_010402	Hand2	1.7	5E-03
BC059060	Gm3893	1.7	5E-03
ENSMUST00000059524	Ahcy	1.7	2E-02
XR_141075	LOC100862332	1.7	5E-02
NR_033637	Gm4349	1.7	1E-02
NM_183281	2310005G13Rik	1.7	3E-03
ENSMUST00000097570	Gm8618	1.7	4E-02

XM_003085853	Gm8674	1.7	7E-03
BC086484	Oog1	1.7	2E-02
X01134	Trav9d-3	1.7	3E-02
NM_008249	Tfb2m	1.7	1E-02
NM_178653	Sccpdh	1.7	3E-05
NM_025634	2310042E22Rik	1.7	1E-02
NM_001029930	Gm5796	1.7	3E-02
NM_027559	Wdr96	1.7	4E-03
NM_010305	Gnai1	1.7	2E-03
X65588	Ppnr	1.7	5E-02
NM_001142804	Acss3	1.7	3E-02
NM_001256161	9430069I07Rik	1.7	2E-02
NM_008333	Ifna11	1.7	3E-02
NM_009920	Cnih2	1.7	4E-03
NR_015456	D7Ertd715e	1.6	8E-03
NM_029639	1600029D21Rik	1.6	8E-04
NM_011467	Spr	1.6	5E-03
NR_033570	Gm7104	1.6	2E-02
NM_028440	3110003A17Rik	1.6	1E-02
NR_037691	Gm4776	1.6	8E-05
NM_011315	Saa3	1.6	1E-02
NM_001177775	H60b	1.6	1E-02
ENSMUST00000005509	Stx1a	1.6	3E-02
NM_017370	Hp	1.6	3E-02
NM_025980	Nrarp	1.6	1E-02
ENSMUST00000024706	Pla2g7	1.6	1E-02
NM_181593	Itpkc	1.6	7E-03
ENSMUST00000156702	Crat	1.6	4E-02
ENSMUST00000105823	Sh2d5	1.6	5E-03
AK008598	2010320O07Rik	1.6	4E-02
NR_033476	Gm10789	1.6	4E-02
ENSMUST00000080669	Gm4559	1.6	2E-02
BC023886	0610009E02Rik	1.6	1E-02
XR_105512	Gm20251	1.6	4E-03
NM_027120	Nmrk2	1.6	1E-02
NM_019540	Pfpl	1.6	1E-02
NR_045170	Gm10336	1.6	3E-03
ENSMUST00000092990	Agpat9	1.6	9E-04
NR_033554	D330041H03Rik	1.6	2E-02
XR_141206	Gm3591	1.6	3E-02
NM_198250	Lrrc4b	1.6	2E-03
NR_046040	1700009C05Rik	1.6	3E-03
NM_001034905	Spag11b	1.6	1E-02
NM_153677	Ush1c	1.6	1E-02
NR_028417	Gm7854	1.6	8E-03
NM_001039368	Polr2k	1.6	1E-03

NM_001011839	Olfr1437	1.6	4E-02
NM_029361	Wnk2	1.6	4E-04
NM_001039689	Rhox4c	1.6	2E-02
NM_020486	Bcam	1.6	9E-04
NM_024166	Chchd2	1.6	3E-02
NM_008483	Lamb2	1.6	1E-04
BC003284	Dcaf4	1.6	5E-02
NM_001256309	Gm933	1.6	6E-03
NM_053131	Pcdhb6	1.6	2E-02
NM_009622	Adcy1	1.6	1E-02
NR_045113	Gm2027	1.6	3E-02
NM_146326	Olfr943	1.6	5E-02
ENSMUST00000025840	Mtl5	1.6	3E-03
X04330	Trav12-2	1.6	5E-02
NM_008102	Gch1	1.6	2E-02
NM_029960	9230104L09Rik	1.6	5E-02
NM_199013	Irgc1	1.6	3E-03
XM_003086877	Gm3141	1.6	2E-02
DQ080431	1700003F12Rik	1.6	3E-02
NM_023580	Epha1	1.6	4E-02
NM_009052	Bex1	1.6	2E-02
NR_038182	4930425K10Rik	1.6	2E-02
NM_001042660	Smad7	1.6	3E-03
NM_153598	Ugt2b34	1.6	3E-02
NM_146351	Olfr1133	1.6	5E-02
NM_172961	Abat	1.6	4E-02
NM_177039	A530016L24Rik	1.6	2E-04
NM_146628	Olfr344	1.6	3E-02
NR_030645	Mir467e	1.6	2E-02
NM_134236	Vmn1r203	1.6	1E-02
NM_001160414	Wfdc9	1.6	4E-02
NM_183368	Sytl3	1.5	7E-03
NM_145473	Csdc2	1.5	2E-03
NM_007913	Egr1	1.5	1E-02
NR_045346	Gm11648	1.5	3E-03
NM_033567	Cecr6	1.5	2E-02
NM_011937	Gnpda1	1.5	4E-02
NM_008208	H2-T3	1.5	8E-03
NM_010835	Msx1	1.5	1E-02
NM_013741	Smok2a	1.5	9E-03
ENSMUST00000033930	Dusp4	1.5	3E-02
NR_045143	AA536875	1.5	9E-03
NM_175219	C130026I21Rik	1.5	6E-03
AK077079	Gm19386	1.5	2E-02
NM_025420	Lce1m	1.5	4E-02
NM_001081425	Rbm24	1.5	3E-04

NM_001173460	Sirpb1b	1.5	8E-04
NM_001163525	S100a14	1.5	3E-04
NM_008097	Gcdh	1.5	5E-03
NM_009061	Rgs2	1.5	2E-02
ENSMUST00000165723	Nkx3-2	1.5	1E-02
NM_176828	C030006K11Rik	1.5	1E-03
NM_030047	4930458L03Rik	1.5	1E-02
NM_139225	Defb10	1.5	4E-02
NM_026812	Hddc3	1.5	9E-03
NM_173786	Apol9a	1.5	4E-02
NM_009918	Cnga3	1.5	2E-02
BC150842	Unkl	1.5	4E-02
NM_013917	Pttg1	1.5	1E-02
NR_033784	1700041C23Rik	1.5	2E-03
ENSMUST00000172388	Rgs1	1.5	8E-03
NM_027697	4933413G19Rik	1.5	4E-03
ENSMUST00000087822	Olfr76	1.5	2E-02
AY522648	B430306N03Rik	1.5	7E-03
NM_001013823	Krtap4-16	1.5	4E-02
NM_207208	Clca6	1.5	3E-02
NM_008002	Fgf10	1.5	4E-02
NM_033570	Cnnm4	1.5	1E-02
NM_009737	Bcat2	1.5	3E-03
ENSMUST00000029140	Procr	1.5	3E-02
NM_146471	Olfr1393	1.5	3E-02
NM_175393	4930555G01Rik	1.5	9E-03
ENSMUST00000056571	Syne1	1.5	1E-03
ENSMUST00000003444	Ccdc65	1.5	3E-03
ENSMUST00000005364	G6pc2	1.5	2E-03
XM_003086259	Gm14025	1.5	2E-02
NM_008475	Krt4	1.5	3E-02
ENSMUST00000167273	Ppp1r15a	1.5	2E-02
NM_181344	C1rl	1.5	5E-03
NM_020027	Prrc2a	1.5	2E-04
NM_027857	Acy3	1.5	4E-03
NR_003619	6330549D23Rik	1.5	2E-02
ENSMUST00000165255	Vmn1r238	1.5	3E-02
ENSMUST00000031304	Tesc	1.5	3E-04
AK084734	Gm20328	1.5	1E-02
ENSMUST00000099992	Pde11a	1.5	1E-02
BC147228	2610042L04Rik	1.5	2E-02
AK086006	Gm3916	1.5	5E-02
NM_029594	Il31	1.5	7E-03
NM_001115075	H2-M5	1.5	4E-02
ENSMUST00000063204	1700025K23Rik	1.5	3E-02
ENSMUST00000030942	Mrpl20	1.5	7E-03

ENSMUST00000121339	Mrps17	1.5	4E-02
NR_046195	A930041C12Rik	1.5	2E-02
NM_028030	Rbpms2	1.5	6E-04
NM_009174	Siah2	1.5	3E-03
XR_104965	Gm20120	1.5	3E-02
ENSMUST00000025025	Dusp1	1.5	2E-02
ENSMUST00000087211	Ppp1r10	1.5	5E-03
NM_175118	Dusp28	1.5	2E-02
NM_013610	Ninj1	1.5	2E-03
NM_027198	Zswim7	1.5	7E-03
NM_001164044	Ccl27a	1.5	3E-02
NM_001085530	Gm13298	1.5	5E-03
NM_016970	Klrg1	1.5	2E-02
NM_013880	Plcl2	1.5	5E-03
NM_019864	Atr	1.5	3E-02
NM_008416	Junb	1.5	2E-02
AK134852	5730409K12Rik	1.5	3E-03
NM_198601	Trim52	1.5	4E-02
XM_141933	Mageb17-ps	1.5	1E-02
NM_011948	Map3k4	1.5	2E-02
NM_175276	Fhod3	1.5	1E-04
XM_916257	Gm4802	1.5	2E-02
NR_028311	Gm4371	1.5	3E-02
NM_145158	Emilin2	1.5	2E-04
NM_025529	Nudt8	1.5	1E-02
NM_145403	Tmprss4	1.5	8E-03
NM_008020	Fkbp2	1.5	6E-03
NM_026689	Mul1	1.5	3E-02
BC027570	Gm3086	1.5	1E-02
AK145728	Gm20325	1.5	2E-02
NM_008700	Nkx2-5	1.5	3E-03
NM_030180	Usp54	1.5	4E-02
NM_011915	Wif1	1.5	3E-02
NR_015571	4930471G03Rik	1.5	3E-03
NM_007828	Dapk3	1.5	8E-03
NR_015477	2310050B05Rik	1.5	5E-02
NM_022331	Herpud1	1.5	9E-03
XR_107349	Gm13187	1.5	1E-02
NR_040366	LOC100642166	1.5	5E-02
NM_177794	Tmem26	1.5	4E-02
ENSMUST00000168579	Slc16a3	1.5	7E-03
ENSMUST00000129421	Hal	1.5	6E-03
ENSMUST00000025249	Apom	1.5	4E-02
ENSMUST00000108105	Gm5148	1.5	5E-03
NM_001013824	Gm5662	1.5	4E-02
NM_021565	Midn	1.5	3E-03

NM_024441	Hspb2	1.5	1E-02
NM_026646	Slc25a22	1.5	7E-03
NM_001167793	Gm3286	1.5	8E-03
NM_144943	Cd207	1.5	4E-02
ENSMUST00000031766	Asns	1.5	8E-03
NM_177544	Ang4	1.5	1E-02
BC083134	Rpl3	1.5	3E-03
ENSMUST00000099201	Sirpb1a	1.5	3E-02
NM_027457	Tmem242	1.5	1E-02
NR_040430	Gm15787	1.4	2E-02
XR_140622	Gm8273	1.4	9E-03
NM_008946	Psmb6	1.4	3E-03
NM_007792	Csrp2	1.4	2E-02
NM_146754	Olfr556	1.4	8E-03
NM_027399	Steap1	1.4	9E-03
NM_177078	Adrbk2	1.4	8E-04
NM_172913	Tox3	1.4	3E-02
NR_045879	Gm10532	1.4	3E-03
ENSMUST00000051209	Peg3	1.4	2E-02
NM_001037248	Gm4567	1.4	1E-02
NM_025631	Bpifb2	1.4	8E-03
NM_011963	Psg18	1.4	4E-02
NM_013614	Odc1	1.4	3E-03
NM_011724	Xirp1	1.4	1E-02
NM_016696	Gpc1	1.4	4E-02
NM_027222	2010001M09Rik	1.4	2E-02
NM_007584	Ddr1	1.4	1E-03
NR_027905	LOC106740	1.4	5E-02
AK081400	Gm12371	1.4	6E-03
NM_007529	Bcan	1.4	4E-02
NM_001081191	Eml5	1.4	2E-03
NM_001160378	Fam46a	1.4	3E-02
NM_016694	Park2	1.4	4E-03
ENSMUST00000050899	Tmem198	1.4	2E-02
ENSMUST00000006718	Wnt10a	1.4	4E-02
NR_024513	Gm5	1.4	2E-02
ENSMUST00000078890	Vmn1r30	1.4	1E-02
NM_146961	Olfr539	1.4	6E-04
NM_028325	Zcchc12	1.4	3E-02
NM_134147	Macrod1	1.4	8E-04
NR_045317	4930526L06Rik	1.4	4E-02
NM_010813	Mnt	1.4	2E-02
AK007073	1700095A13Rik	1.4	1E-02
NM_134170	Vmn1r32	1.4	7E-03
ENSMUST00000112900	Zfand4	1.4	2E-03
NR_040477	4930503O07Rik	1.4	1E-02

NM_029006	Kcnk16	1.4	2E-02
NM_013844	Zfp68	1.4	4E-02
BC006907	Ralb	1.4	2E-02
NR_033117	Gm3020	1.4	2E-02
NM_010603	Kcnj12	1.4	1E-03
ENSMUST00000142090	Dgkz	1.4	3E-02
ENSMUST00000026296	Fgd1	1.4	3E-02
ENSMUST00000068641	Sertad3	1.4	2E-04
ENSMUST00000169395	LOC100039659	1.4	3E-02
ENSMUST00000108689	Myh3	1.4	5E-02
NM_011897	Spry2	1.4	3E-02
NM_028629	Kprp	1.4	7E-03
NM_010655	Kpna2	1.4	6E-03
XR_142007	LOC100862165	1.4	1E-02
NM_016899	Rab25	1.4	3E-02
ENSMUST00000034830	Crabp1	1.4	1E-02
XM_003689295	Gm19570	1.4	1E-02
NM_025835	Pccb	1.4	8E-05
NM_012052	Rps3	1.4	2E-02
NM_001164739	Fam47c	1.4	2E-02
NM_013863	Bag3	1.4	5E-04
NM_011220	Pts	1.4	4E-02
NR_046044	AI463170	1.4	2E-02
ENSMUST00000105336	Dot1l	1.4	2E-02
NR_045922	1700051A21Rik	1.4	3E-02
NM_021793	Tmem8	1.4	1E-02
NM_175381	2700081O15Rik	1.4	2E-02
NM_027046	Ccdc54	1.4	8E-03
NM_172151	Zdhhc8	1.4	9E-03
NM_025960	Trappc6a	1.4	3E-02
NM_007601	Capn3	1.4	3E-02
XR_107803	Gm19462	1.4	4E-02
NM_001195767	Gm6772	1.4	2E-02
NM_144815	Cecr5	1.4	1E-02
NM_028312	Ccdc12	1.4	1E-02
AF011425	Vmn2r30	1.4	3E-02
NM_177350	Gldn	1.4	4E-02
NM_175333	Slc25a41	1.4	3E-02
ENSMUST00000090839	Selenbp1	1.4	2E-02
NR_045438	Mospd4	1.4	4E-02
NM_009314	Tacr2	1.4	4E-02
NM_001004468	Tacc2	1.4	5E-03
NR_040370	Gm13752	1.4	5E-02
NR_027915	1700120K04Rik	1.4	2E-02
NM_008412	Ivl	1.4	3E-02
NM_008454	Klk1b16	1.4	4E-02

NM_030098	Rnase6	1.4	1E-03
NM_021423	Shank3	1.4	1E-02
NM_001177715	Gm2897	1.4	2E-02
ENSMUST00000057866	Nrsn1	1.4	2E-02
NM_177075	C030019I05Rik	1.4	3E-02
NR_029470	Gm10863	1.4	5E-02
NM_146501	Olfr318	1.4	3E-02
NM_007931	Endog	1.4	3E-02
ENSMUST00000172775	Msx3	1.4	1E-02
NM_177464	D19Ertd386e	1.4	5E-02
NM_001018063	Cxx1b	1.4	4E-03
NM_008343	Igfbp3	1.4	3E-02
NR_045069	Gm16576	1.4	1E-02
NM_146555	Olfr805	1.4	4E-02
NM_013903	Mmp20	1.4	1E-02
NM_001083884	2210415F13Rik	1.4	4E-02
NM_001048207	Gypc	1.4	5E-03
NM_008738	Nrtn	1.4	4E-02
NM_001195094	Ccdc42b	1.4	3E-02
NM_146016	Eml6	1.4	2E-02
NM_008780	Pax1	1.4	1E-02
NM_178378	Iqcg	1.4	3E-02
BC120716	Rpl22l1	1.4	2E-02
NM_021566	Jph2	1.4	4E-03
NM_172994	Ppp2r2c	1.4	2E-02
NM_001017426	Kdm6b	1.4	3E-02
ENSMUST00000031876	Stra8	1.4	3E-02
NM_181039	Lphn1	1.4	9E-04
NM_033073	Krt7	1.4	4E-02
NM_001267622	Ttc28	1.4	4E-02
NM_001171512	Obscn	1.4	2E-03
ENSMUST00000154376	Cpeb3	1.4	8E-04
GU269230	Gm5325	1.4	2E-02
NM_145430	BC017647	1.4	7E-04
NM_007427	Agrp	1.4	4E-02
NM_053245	Aipl1	1.4	1E-03
ENSMUST00000040536	Batf	1.4	5E-02
ENSMUST00000057090	Synpo2l	1.4	3E-02
NM_174995	Mgst2	1.4	4E-02
NM_025396	Pgls	1.4	1E-02
NM_133900	Psph	1.4	2E-03
NM_001030293	Spry3	1.4	1E-02
NR_040737	4930502A04Rik	1.4	5E-02
NM_029582	Txndc11	1.4	3E-02
NM_010171	F3	1.4	2E-02
NM_001033215	Zfp957	1.4	2E-02

NM_007911	Efnb3	1.4	4E-02
NM_010919	Nkx2-2	1.4	2E-02
NM_134096	Fam19a5	1.4	5E-02
NM_024124	Hdac9	1.4	2E-03
NM_019684	Srpk3	1.4	2E-02
ENSMUST00000140653	D5Ertd579e	1.4	4E-02
NM_007881	Atn1	1.4	1E-02
ENSMUST00000119080	Gjb1	1.4	1E-02
ENSMUST00000023593	Adipoq	1.4	2E-02
ENSMUST00000173472	Olfr103	1.4	4E-02
XR_141809	Gm19270	1.4	2E-02
NM_023304	Fgf22	1.4	5E-02
XR_140804	LOC100862260	1.4	3E-02
NM_145612	Zfp810	1.4	8E-03
NR_045368	1700120E14Rik	1.4	2E-02
NM_030064	Phf23	1.4	8E-04
NM_009603	Chrne	1.4	2E-02
ENSMUST00000025062	Clps	1.4	5E-02
AK129406	BC034090	1.4	4E-02
NM_016742	Cdc37	1.4	8E-03
ENSMUST00000103037	Ush1g	1.4	2E-02
XR_140624	Gm2401	1.4	1E-02
NM_172802	Fscn2	1.4	3E-02
NM_030251	Abtb1	1.4	2E-02
AK020082	6030471H07Rik	1.4	5E-02
ENSMUST00000106068	Adc	1.4	3E-02
ENSMUST00000024802	Ppil1	1.4	3E-02
ENSMUST00000032111	Wbp1	1.4	1E-02
NM_008291	Hsd17b3	1.4	3E-02
NR_045966	4930511A02Rik	1.4	2E-02
NM_007837	Ddit3	1.4	2E-02
NM_203319	Dhx37	1.4	1E-02
ENSMUST00000108723	Lrrc48	1.4	2E-03
ENSMUST00000073251	Gm1568	1.4	4E-02
NM_011697	Vegfb	1.4	7E-05
NM_182930	Plekha6	1.4	2E-02
NR_040427	Gm9899	1.4	6E-03
NR_040327	E130018N17Rik	1.4	4E-02
NM_025687	Tex12	1.4	4E-02
XM_905568	Gm4801	1.4	5E-02
ENSMUST00000025915	Dnajc4	1.4	9E-03
NM_001160239	Fau	1.4	3E-03
XM_891465	Gm6713	1.4	4E-02
NR_033813	Dlk1	1.4	1E-02
NM_009224	Snrnp70	1.4	1E-02
NR_023357	Gm6981	1.4	1E-02

ENSMUST00000063663	B3gat2	1.4	9E-03
NR_027360	5930416I19Rik	1.4	3E-02
NM_178005	Lrrtm2	1.4	3E-02
ENSMUST00000093969	Myo19	1.4	3E-02
NM_001077661	Urgcp	1.4	3E-02
NM_172293	Fam113b	1.4	3E-02
AK019847	Nub1	1.4	1E-02
NR_024068	Snhg7	1.4	4E-02
ENSMUST00000115344	Crisp4	1.4	2E-02
ENSMUST00000034243	Mmp15	1.4	6E-03
NM_183104	4931429L15Rik	1.4	7E-03
ENSMUST00000019135	Gga3	1.4	4E-02
NM_028439	3110009E18Rik	1.4	9E-03
NM_134099	Fbxo4	1.4	2E-03
NM_206822	Olfr10	1.4	2E-02
AK006706	1700047F07Rik	1.4	1E-02
NM_001033039	Klhdc9	1.4	4E-02
NR_045521	Prmt1	1.4	4E-03
NM_018796	Eef1b2	1.4	1E-02
NM_023627	Isyna1	1.4	5E-03
NM_001170591	Nfu1	1.3	9E-03
BC023421	Fam98c	1.3	6E-03
NM_001145972	Gpr114	1.3	4E-02
NM_001085492	Rere	1.3	9E-03
NM_001010836	Ppp1r13l	1.3	2E-02
ENSMUST00000043459	Ankrd9	1.3	3E-02
NR_036572	Rps15a-ps4	1.3	1E-02
NM_026815	Upk1a	1.3	2E-02
NM_144939	Frs3	1.3	2E-02
ENSMUST00000055990	Eef1a2	1.3	5E-04
NR_015527	4930417O13Rik	1.3	4E-02
ENSMUST00000071093	Rims3	1.3	2E-02
ENSMUST00000035218	Nckipsd	1.3	7E-03
NM_198622	H1fx	1.3	3E-02
NM_147011	Olfr1044	1.3	4E-02
NM_028219	Cnfn	1.3	6E-03
NM_181820	Tmc4	1.3	2E-02
NM_025538	Alkbh7	1.3	3E-02
ENSMUST00000023572	Cxadr	1.3	3E-02
NM_001163006	Minos1	1.3	5E-02
NM_007675	Ceacam10	1.3	2E-02
NR_045738	8030423F21Rik	1.3	2E-03
ENSMUST00000023390	Drd3	1.3	1E-03
ENSMUST00000016088	Gatsl2	1.3	7E-03
ENSMUST00000106437	Hs3st4	1.3	4E-02
NM_001034115	Shank1	1.3	3E-02

NM_007728	Coch	1.3	2E-03
NM_001200038	1700021K19Rik	1.3	2E-02
NM_175432	Tmem132c	1.3	4E-02
ENSMUST00000017142	Svs4	1.3	4E-02
NM_001100455	Lcn11	1.3	4E-02
NM_028733	Pacsin3	1.3	1E-02
NM_026725	Dusp23	1.3	3E-03
NM_001013826	Dupd1	1.3	4E-02
NM_027093	2310003L22Rik	1.3	4E-02
AK006338	1700025H01Rik	1.3	4E-02
BC100410	1700084M14Rik	1.3	2E-02
ENSMUST00000108674	Ntn1	1.3	5E-03
NM_011164	Prl	1.3	1E-02
NM_029906	9530003J23Rik	1.3	2E-03
NM_030256	Bcl9l	1.3	1E-02
ENSMUST00000036181	Car14	1.3	1E-02
NM_001177522	Gm14850	1.3	1E-02
NM_025431	Llph	1.3	7E-03
ENSMUST00000099619	Olfr1277	1.3	1E-02
NM_001164376	Ctnna3	1.3	9E-03
NM_001167581	Gsdmcl1	1.3	2E-02
ENSMUST00000103181	Cds2	1.3	9E-03
NM_019993	Aldh9a1	1.3	8E-03
NM_008746	Ntrk3	1.3	5E-02
ENSMUST00000004173	5730403B10Rik	1.3	9E-03
NM_007681	Cenpa	1.3	5E-03
NM_010358	Gstm1	1.3	1E-02
NM_001114664	Iqsec2	1.3	1E-03
ENSMUST00000098142	Olfr703	1.3	1E-02
NM_178764	Fam168a	1.3	8E-03
NM_008826	Pfkl	1.3	2E-03
NM_001162880	Syce3	1.3	3E-02
BC100475	1110034G24Rik	1.3	1E-02
NM_009503	Vcp	1.3	4E-02
NM_134224	Vmn1r202	1.3	2E-02
NM_172132	Kdm4b	1.3	3E-02
ENSMUST00000033157	Ndufab1	1.3	4E-02
ENSMUST00000109184	Nfatc2	1.3	5E-02
NR_040342	A330093E20Rik	1.3	2E-02
NM_001199961	Ccl27b	1.3	2E-02
ENSMUST00000149046	Rtl1	1.3	4E-02
ENSMUST00000046506	Clcf1	1.3	4E-02
ENSMUST00000106630	Sstr2	1.3	4E-02
NM_008254	Hmgcl	1.3	2E-02
ENSMUST00000048572	Hlx	1.3	2E-02
BC116839	AI606181	1.3	2E-02

NM_001033343	Sec31b	1.3	1E-02
XR_107767	Gm16055	1.3	1E-02
NM_009665	Amd1	1.3	4E-02
NM_001256055	Gm9781	1.3	3E-02
NR_033499	Gm8773	1.3	2E-02
NM_001033875	Ctrc	1.3	4E-03
ENSMUST00000051757	Slc26a1	1.3	1E-02
NR_045457	1700030A11Rik	1.3	5E-02
NM_172665	Pdk1	1.3	1E-04
NR_030417	Mir744	1.3	8E-03
NM_001164607	Maf1	1.3	2E-02
NM_145546	Gtf2b	1.3	3E-02
NM_001081372	Ces1b	1.3	3E-02
NM_175307	Fam46b	1.3	3E-02
NM_001013616	Trim6	1.3	5E-02
ENSMUST00000025904	Prdx5	1.3	5E-03
NM_153389	Atp10d	1.3	3E-02
NM_153136	Nudt18	1.3	4E-02
NM_021428	Dexi	1.3	7E-03
ENSMUST00000152673	Lsr	1.3	2E-02
NR_045708	Gm11944	1.3	2E-02
NM_178873	Adck2	1.3	6E-03
ENSMUST00000079738	1700011I03Rik	1.3	4E-02
ENSMUST00000071798	Tnnt1	1.3	3E-02
BC094623	Gstp2	1.3	9E-03
ENSMUST00000107894	Dgke	1.3	2E-02
NM_010357	Gsta4	1.3	1E-02
NR_027959	B830017H08Rik	1.3	4E-02
NM_024227	Mrpl28	1.3	3E-03
NR_040587	1700010I02Rik	1.3	3E-02
XR_141677	LOC100862592	1.3	5E-02
NM_001146325	Tigit	1.3	2E-02
NR_045997	1700113A16Rik	1.3	2E-02
NM_138677	Edem1	1.3	3E-02
NR_038081	Raver1-fdx1l	1.3	1E-02
ENSMUST00000037843	Fam100a	1.3	3E-02
NM_153585	Cnot10	1.3	3E-02
NM_016960	Ccl20	1.3	2E-02
NR_040323	4930500J02Rik	1.3	1E-02
NM_001011819	Olfr312	1.3	2E-02
ENSMUST00000079306	Lypd4	1.3	3E-02
XM_979107	Gm7762	1.3	4E-02
NM_026486	Tctn2	1.3	3E-03
NM_009420	Crisp2	1.3	2E-02
NM_001081188	Exosc7	1.3	4E-02
ENSMUST00000079431	Kcnip2	1.3	7E-03

NM_177564	Dhrs11	1.3	2E-02
ENSMUST00000038287	Dusp5	1.3	3E-02
NM_146155	Ahdc1	1.3	4E-02
ENSMUST00000099557	Pak6	1.3	2E-02
ENSMUST00000162731	Zfp579	1.3	3E-02
ENSMUST00000107864	Tmem8b	1.3	2E-02
NM_021899	Foxj2	1.3	2E-02
NM_009818	Ctnna1	1.3	5E-04
NM_001145198	1500009L16Rik	1.3	1E-02
NM_172417	2310042D19Rik	1.3	2E-02
NM_007874	Reep5	1.3	1E-02
NM_028680	Ift57	1.3	2E-02
NM_001005767	Parl	1.3	6E-03
BC016232	Capza1	1.3	4E-02
NM_011658	Twist1	1.3	2E-02
NM_001038230	Anapc11	1.3	7E-03
ENSMUST00000005647	Ndufs3	1.3	3E-03
NM_009088	Polr1a	1.3	8E-05
NM_007533	Bckdha	1.3	1E-02
NR_045737	9430014N10Rik	1.3	2E-02
XR_141166	Gm3897	1.3	3E-02
ENSMUST00000037872	Dnajb5	1.3	3E-02
NM_001081171	Lama5	1.3	4E-02
ENSMUST00000107773	Tmod1	1.3	3E-02
NM_057171	Bag6	1.3	4E-03
NM_023637	Sars2	1.3	1E-02
NM_009951	Igf2bp1	1.3	2E-03
NR_033546	Gm15348	1.3	1E-02
NM_009343	Phf1	1.3	2E-03
NM_011480	Srebf1	1.3	2E-02
NM_147052	Olfr589	1.3	3E-02
NM_008310	Htr1f	1.3	2E-02
NM_020014	Gfra4	1.3	1E-02
AK085606	LOC621549	1.3	4E-02
ENSMUST00000172602	Fam161a	1.3	4E-02
ENSMUST00000026538	Echs1	1.3	3E-02
NR_029553	Mir140	1.3	2E-02
XR_140944	Gm6202	1.3	1E-02
NR_045290	4732491K20Rik	1.3	2E-02
NM_029197	4930528F23Rik	1.3	4E-02
ENSMUST00000006136	Dnase1	1.3	4E-02
ENSMUST00000113530	Klf15	1.3	2E-02
NM_001166399	Rab3a	1.3	2E-03
ENSMUST00000111507	Olfr1263	1.3	4E-02
NM_031405	Srrt	1.3	2E-02
NR_015547	1700009J07Rik	1.3	2E-02

NM_020568	Plin4	1.3	5E-03
NM_010827	Msc	1.3	1E-02
NM_024464	Pigx	1.3	7E-04
NM_001003953	Kdm2b	1.3	1E-02
ENSMUST00000097495	Dok6	1.3	9E-03
NM_001033535	Tnfaip8l3	1.3	3E-02
AK043908	Dedd2	1.3	1E-02
NM_173016	Vat1l	1.3	3E-02
NM_178576	Cpsf4	1.3	2E-02
NM_011850	Nr0b2	1.3	3E-02
BC051019	BC051019	1.3	4E-02
XM_003085142	Gm19399	1.3	2E-02
NM_018761	Ctnnal1	1.3	7E-03
NM_001081365	0610010O12Rik	1.3	4E-02
ENSMUST00000167334	Sis	1.3	7E-03
NM_001243017	Gm15140	1.3	2E-02
NM_198423	Bahcc1	1.3	7E-03
NM_009010	Rad23a	1.3	2E-02
AK054201	E230028L10Rik	1.3	4E-02
NM_001082975	Sdr39u1	1.3	2E-02
NM_199222	Lman1l	1.3	4E-02
NM_025600	Dda1	1.3	4E-03
NM_026768	Mrps18a	1.3	9E-03
NR_033707	Zscan4a	1.3	2E-02
BC042473	Rtcd1	1.3	3E-02
NM_025954	Pgp	1.3	4E-02
NM_133722	Fam108c	1.3	4E-02
NM_009455	Ube2e1	1.3	1E-02
NM_030728	9930013L23Rik	1.3	5E-02
NM_025363	1110001J03Rik	1.3	9E-03
AK034016	9030407P20Rik	1.3	4E-02
NM_172276	Sfswap	1.3	4E-02
NM_175109	Rps19bp1	1.3	4E-02
ENSMUST00000117782	Adamtsl4	1.3	2E-02
NM_028202	Kbtbd5	1.3	2E-02
NR_028593	Gm10069	1.3	3E-02
NM_021028	Tk2	1.3	2E-02
XR_141915	Gm3878	1.3	4E-03
XM_910275	H2afb2-ps	1.3	4E-02
ENSMUST00000033756	Asb9	1.3	2E-02
NM_177994	C030046I01Rik	1.3	3E-02
NR_015349	Etohd2	1.3	3E-02
NR_038023	Gm15910	1.3	1E-02
NM_025848	Sdhd	1.3	3E-02
NM_001078649	Tmem134	1.3	1E-02
NM_025615	2810004N23Rik	1.3	5E-02

ENSMUST00000114143	Fam3a	1.3	1E-02
NM_175638	Wnk4	1.3	4E-02
NM_134148	Carns1	1.3	1E-02
NM_024241	Kif24	1.3	3E-02
NM_133765	Fbxo31	1.3	1E-03
XR_142318	LOC100862575	1.3	4E-02
NM_001017955	Zscan18	1.3	3E-02
ENSMUST00000112066	Sirt4	1.3	2E-02
NM_032418	Dmpk	1.3	5E-04
NM_183285	Kctd2	1.3	3E-02
NM_001013780	Slc25a34	1.3	1E-02
NM_011716	Wfs1	1.3	1E-02
NM_152803	Hpse	1.3	2E-02
AK135804	Gm10839	1.3	2E-02
NM_029372	1700011F14Rik	1.3	3E-02
NM_010659	Krt31	1.3	2E-02
NM_010027	Ddt	1.3	2E-02
NM_010893	Neu1	1.3	3E-02
NM_018792	Hils1	1.3	2E-03
NM_001163787	Ccdc151	1.3	4E-02
NM_172945	Ankrd13b	1.3	2E-02
NM_001080819	Arid1a	1.3	3E-02
NM_134011	Tbrg4	1.3	2E-02
NM_198619	Zfp933	1.3	4E-02
NM_001085548	Krtap4-9	1.3	2E-02
NM_009055	Rfx1	1.3	1E-02
NR_040472	1700112H15Rik	1.3	3E-02
ENSMUST00000061019	Kif2b	1.3	3E-02
ENSMUST00000040971	Capn5	1.3	3E-03
NM_009781	Cacna1c	1.3	1E-02
NM_026596	4930591A17Rik	1.3	4E-02
AK015283	4930432B10Rik	1.3	3E-02
NM_007463	Speg	1.3	8E-03
NM_001253694	Zfp219	1.3	4E-02
NM_001013381	Rsad1	1.3	3E-02
BC058791	E030002O03Rik	1.3	3E-02
NM_027327	2210013O21Rik	1.3	3E-02
NM_001205070	Josd2	1.3	3E-02
NM_172772	Fam63b	1.3	4E-02
NR_028053	Ngrn	1.3	2E-03
ENSMUST00000102584	Ung	1.3	2E-02
ENSMUST00000026476	Mbd6	1.3	5E-02
ENSMUST00000060833	Gpa33	1.3	4E-02
AK133679	Gm20255	1.3	8E-03
NM_010473	Hrc	1.3	3E-02
XM_621386	Gm5958	1.3	1E-02

NR_035435	Mir1895	1.3	8E-03
NM_146871	Olfr898	1.3	4E-02
ENSMUST00000034230	Cx3cl1	1.3	9E-03
NM_019479	Hes6	1.3	2E-02
NM_029428	Adad2	1.3	5E-02
NM_001013012	Zfp787	1.2	2E-02
NM_028638	Gadl1	1.2	4E-02
NM_175164	Arhgap26	1.2	3E-02
ENSMUST00000043415	Tekt5	1.2	3E-02
NM_001163819	Fance	1.2	5E-02
NM_030749	Sil1	1.2	1E-02
NM_144528	Rnf126	1.2	5E-03
NM_133667	Pdk2	1.2	5E-03
NM_153082	Dnajc27	1.2	4E-02
ENSMUST00000029128	Map1lc3a	1.2	4E-02
NM_020036	Calm4	1.2	5E-02
NM_026782	1110021J02Rik	1.2	3E-02
ENSMUST00000108684	Myh13	1.2	3E-02
NM_001171026	Os9	1.2	3E-02
NM_175366	Mex3b	1.2	4E-02
NM_008299	Dnajb3	1.2	2E-02
NM_027884	Tns1	1.2	5E-02
ENSMUST00000123437	Lmo2	1.2	4E-02
NM_138582	D17H6S56E-3	1.2	7E-03
NM_018870	Pgam2	1.2	1E-02
NM_019913	Txn2	1.2	1E-02
NM_029720	Creld2	1.2	1E-02
NM_030726	Mrgprh	1.2	3E-02
NM_027937	Caskin1	1.2	4E-02
NM_153514	Rhobtb2	1.2	4E-02
ENSMUST00000144177	Adhfe1	1.2	3E-02
NM_009448	Tuba1c	1.2	3E-02
NR_045919	1700054M17Rik	1.2	2E-02
NM_010147	Epn1	1.2	8E-04
NM_029186	Tmem180	1.2	2E-02
NM_201359	Tmem106c	1.2	4E-02
NM_026938	Tmem160	1.2	2E-02
NM_011099	Pkm	1.2	2E-02
NM_146705	Olfr1451	1.2	5E-02
NM_001029990	Mettl17	1.2	3E-02
ENSMUST00000073945	Vkorc1l1	1.2	4E-02
NM_175457	Heatr7a	1.2	4E-02
NM_008650	Mut	1.2	4E-02
NM_011680	Usf2	1.2	1E-02
NM_198671	Gse1	1.2	4E-02
NM_007442	Alx4	1.2	3E-02

XR_106366	Gm11702	1.2	3E-02
NM_010330	Emb	1.2	5E-02
NM_172148	B9d2	1.2	4E-02
NM_023814	Tbx18	1.2	2E-02
NM_011508	Eif1	1.2	7E-03
NM_010135	Enah	1.2	3E-02
NR_002700	Gm7334	1.2	2E-02
NM_028005	2310047M10Rik	1.2	2E-02
NM_023475	Serhl	1.2	2E-02
NM_009393	Tnnc1	1.2	1E-02
NM_152808	Slc44a2	1.2	1E-02
ENSMUST00000116563	Klc2	1.2	3E-02
NM_172835	Peli3	1.2	3E-02
ENSMUST00000028852	Mrps5	1.2	2E-02
NM_001163662	Nucb1	1.2	2E-02
NM_001039126	Asb1	1.2	3E-02
NM_001083618	Ttll9	1.2	3E-02
NM_001029938	Rilp	1.2	1E-02
ENSMUST00000041830	Mettl11a	1.2	5E-02
ENSMUST00000090750	Pde4dip	1.2	1E-02
NM_139153	Agap3	1.2	2E-02
ENSMUST00000080058	Egln2	1.2	3E-02
NM_053100	Trim8	1.2	2E-02
NM_148932	Pom121	1.2	4E-03
NM_134168	Vmn1r38	1.2	2E-02
NR_030700	4831440E17Rik	1.2	4E-02
NM_018867	Cpxm2	1.2	5E-02
NM_025839	Nudt16l1	1.2	2E-02
AK040725	A530020G20Rik	1.2	2E-02
NM_001033205	Zfp575	1.2	3E-02
NM_001171582	Mars	1.2	1E-02
NM_019776	Snd1	1.2	7E-03
NM_183208	Zmiz1	1.2	3E-02
NM_173384	Sox30	1.2	2E-02
NM_007421	Adssl1	1.2	3E-02
NM_007851	Defa5	1.2	3E-02
NM_033561	Eif4h	1.2	5E-03
ENSMUST00000001455	Mef2d	1.2	3E-02
NM_027687	Cabyr	1.2	2E-02
NM_001111111	Atg16l2	1.2	5E-02
NM_146104	Aph1a	1.2	2E-02
NR_045458	4933433H22Rik	1.2	3E-02
NM_175022	Prr12	1.2	4E-02
NM_029872	Hnrnpa0	1.2	5E-02
NM_080635	Eif3h	1.2	2E-02
NM_001033548	6030429G01Rik	1.2	4E-02

NM_009664	Ambn	1.2	5E-02
NM_178035	Fads6	1.2	3E-02
NM_007383	Acads	1.2	8E-04
ENSMUST00000102697	Flcn	1.2	5E-02
BC071212	1700037H04Rik	1.2	5E-02
NM_019733	Rbpms	1.2	7E-03
NM_001199677	Vps37d	1.2	3E-02
NM_008492	Ldhb	1.2	2E-02
NM_177696	Gdpd4	1.2	4E-02
ENSMUST00000015889	Plekho1	1.2	4E-02
NM_008155	Gpi1	1.2	3E-03
NM_183263	Rnmtl1	1.2	4E-02
ENSMUST00000118411	Mab21l3	1.2	5E-02
NM_054097	Pip4k2c	1.2	3E-02
ENSMUST00000027726	Cyb5r1	1.2	4E-02
NM_007405	Adcy6	1.2	2E-02
NM_015731	Atp9a	1.2	3E-04
NM_020580	Th1l	1.2	3E-02
NM_145614	Dlat	1.2	2E-02
NM_028958	Taf7l	1.2	4E-02
NM_024208	Echdc3	1.2	5E-03
NM_133982	Rpp25	1.2	4E-02
ENSMUST00000113553	Stk11ip	1.2	3E-02
NM_203280	Sphk2	1.2	4E-02
NR_040686	8430423G03Rik	1.2	4E-02
NM_027883	Dhx34	1.2	8E-03
NM_139301	Catsper1	1.2	3E-02
NR_028428	2610005L07Rik	1.2	9E-03
BC147129	C130023O10Rik	1.2	1E-02
NM_016781	Prkag1	1.2	9E-03
NM_001134741	Tdrd5	1.2	5E-02
NM_009228	Snta1	1.2	3E-02
NM_013809	Cyp2g1	1.2	1E-02
NM_177828	Arhgef37	1.2	5E-02
NM_023774	4930550L24Rik	1.2	4E-02
NM_020493	Srf	1.2	6E-04
NM_007620	Cbr1	1.2	4E-02
ENSMUST00000026495	Atp5a1	1.2	1E-02
NM_001081415	Samd1	1.2	8E-03
NM_001130868	Kars	1.2	2E-02
NM_019837	Nudt3	1.2	1E-02
NM_001110252	Hpn	1.2	4E-02
XR_104972	Gm20186	-15.5	1E-06
NM_010381	H2-Ea-ps	-14.4	8E-07
NM_001081130	Ogdhl	-11.3	5E-04
NM_016957	Hmgn2	-7.0	2E-04

NM_010397	H2-T22	-6.9	3E-03
NM_018859	Akr1e1	-6.3	1E-03
NM_010382	H2-Eb1	-6.3	4E-05
NM_001243837	C7	-6.1	3E-05
ENSMUST00000058856	Scd4	-4.9	3E-04
NM_008599	Cxcl9	-4.8	6E-04
ENSMUST00000060125	Scn4b	-4.4	2E-06
NR_028061	Gm8615	-3.6	1E-03
NM_009082	Rpl29	-3.5	4E-06
NM_010259	Gbp1	-3.5	7E-06
NM_008331	Ifit1	-3.3	3E-03
ENSMUST00000045075	Cd300ld	-3.2	9E-04
AK035387	Gm20559	-3.2	1E-03
ENSMUST00000041045	H2afz	-3.0	8E-04
ENSMUST00000159679	Mettl11b	-3.0	8E-04
NM_007609	Casp4	-2.9	7E-04
NM_008329	Ifi204	-2.9	3E-03
NM_001083918	Gm13139	-2.9	7E-03
NM_172980	Slc28a2	-2.9	3E-04
ENSMUST00000040655	H2-Aa	-2.9	1E-03
NM_007753	Cpa3	-2.8	1E-03
NM_138648	Olr1	-2.7	2E-02
NM_177066	Tnni3k	-2.7	1E-02
NM_001033450	Mnda	-2.7	1E-02
AK136967	Gm10808	-2.6	8E-04
NM_172777	Gbp9	-2.6	4E-04
XR_107257	LOC100503226	-2.6	2E-02
NM_010476	Hsd17b7	-2.6	1E-02
NM_176962	Zfp944	-2.5	3E-03
NM_001034859	Gm4841	-2.5	2E-02
NR_030675	D730005E14Rik	-2.5	8E-04
NM_001048204	Zfp455	-2.5	2E-02
NM_001025373	Zfp943	-2.4	4E-05
ENSMUST00000112170	Tlr8	-2.4	6E-03
NR_028300	5330426P16Rik	-2.4	8E-04
NM_205820	Tlr13	-2.4	1E-03
NM_010745	Ly86	-2.4	1E-03
NM_027571	P2ry12	-2.4	4E-03
NR_040766	9430037G07Rik	-2.4	5E-03
NM_010394	H2-Q7	-2.3	2E-04
NM_001204910	AI607873	-2.3	3E-03
NM_021378	Klrc3	-2.3	3E-02
NM_181348	Prune2	-2.3	1E-03
ENSMUST00000027603	Rgs18	-2.3	2E-02
NM_172648	Ifi205	-2.3	4E-02
BC027314	D17H6S56E-5	-2.3	9E-05

NR_030718	F630028O10Rik	-2.3	7E-04
NM_001163004	Pkd2l2	-2.2	2E-04
ENSMUST00000112161	Tlr7	-2.2	2E-04
NM_145227	Oas2	-2.2	3E-03
ENSMUST00000102678	H2-T23	-2.2	4E-03
NM_177600	Ccdc73	-2.2	4E-04
AB097847	Ighg	-2.2	1E-03
NM_001143689	H2-Q4	-2.2	3E-02
NR_040556	Gm6634	-2.2	1E-02
NM_001081117	Mki67	-2.2	1E-03
NM_001039554	Angptl7	-2.2	4E-03
NM_053247	Lyve1	-2.2	1E-03
NR_045766	Gm10509	-2.2	2E-02
NR_046305	LOC100861571	-2.1	2E-02
NM_010501	Ifit3	-2.1	2E-03
NM_029747	2410137M14Rik	-2.1	1E-03
ENSMUST00000106157	Zranb1	-2.1	3E-04
NM_025465	1810029B16Rik	-2.1	1E-02
NM_010104	Edn1	-2.1	3E-04
AK143064	Gm7072	-2.1	3E-06
NM_023386	Rtp4	-2.1	4E-04
NM_001195431	Islr	-2.1	4E-04
AB010352	AB010352	-2.1	5E-02
NM_012008	Ddx3y	-2.1	3E-05
NM_181075	2610524H06Rik	-2.1	5E-02
NM_029733	2010005H15Rik	-2.0	6E-03
ENSMUST00000026360	Itgb8	-2.0	8E-03
NM_172796	Slfn9	-2.0	2E-05
ENSMUST00000080511	Hist1h1b	-2.0	1E-02
ENSMUST00000034260	B3gnt3	-2.0	1E-02
NM_001160415	Apobec3	-2.0	3E-03
NM_029499	Ms4a4c	-2.0	5E-02
NM_007962	Mpzl2	-2.0	8E-03
NM_012054	Aoah	-2.0	1E-02
ENSMUST00000018186	Cyb5r3	-2.0	1E-03
NM_172486	Zfp677	-2.0	2E-02
NR_038044	Gm10033	-2.0	6E-03
NM_008682	Nedd1	-2.0	3E-04
NM_008326	Irgm1	-2.0	9E-03
NM_007651	Cd53	-2.0	4E-03
ENSMUST00000091436	Cubn	-2.0	4E-04
NM_207648	H2-Q6	-2.0	3E-03
NM_146760	Olfr672	-2.0	3E-02
NM_025779	Ccdc109b	-2.0	2E-03
NM_011784	Aplnr	-2.0	1E-03
NM_177909	Slc9a9	-2.0	3E-03

NM_025374	Glo1	-2.0	7E-04
NM_001093749	Mpzl3	-2.0	3E-03
NM_001042605	Cd74	-1.9	1E-03
NM_207105	H2-Ab1	-1.9	5E-03
NR_003568	Gpr137b-ps	-1.9	4E-03
NR_040453	Gm17757	-1.9	2E-02
NM_001011807	Olfr191	-1.9	4E-02
NM_197999	Ces2g	-1.9	1E-02
NM_011163	Eif2ak2	-1.9	6E-03
NM_001113326	Msr1	-1.9	2E-03
NM_001111279	Wdfy1	-1.9	3E-04
AK080053	2310058D17Rik	-1.9	2E-02
NM_001080780	Ret	-1.9	2E-02
NM_153168	Lars2	-1.9	1E-04
NM_008615	Me1	-1.9	4E-04
NM_207229	Plac9	-1.9	4E-04
ENSMUST00000102979	LOC100862646	-1.9	4E-02
NM_016972	Slc7a8	-1.9	3E-03
ENSMUST00000060521	Vmn1r28	-1.9	6E-03
ENSMUST00000159572	Nmrk1	-1.9	8E-03
NM_025415	Cks2	-1.9	3E-03
NR_045627	1700120C14Rik	-1.9	8E-04
NR_040401	C920006O11Rik	-1.9	9E-05
NM_172919	Zfp846	-1.9	1E-02
NM_153100	Rtp3	-1.9	2E-03
NM_172632	Mapk4	-1.9	1E-04
NR_015491	A630089N07Rik	-1.9	4E-02
NM_146566	Olfr830	-1.9	6E-03
NM_001164838	Lrrfip2	-1.9	4E-05
NM_026679	D14Ertd449e	-1.9	1E-04
NM_144539	Slamf7	-1.9	4E-03
BC119072	AK129341	-1.9	5E-03
NM_177712	Zfp874a	-1.9	4E-03
ENSMUST00000087626	Gm5945	-1.9	3E-02
ENSMUST00000078673	Samd12	-1.9	9E-03
NM_011337	Ccl3	-1.9	2E-02
NM_025992	Herc6	-1.9	1E-02
NR_045336	Gm10635	-1.9	9E-03
NM_011854	Oasl2	-1.9	1E-02
NM_001163246	AA987161	-1.9	4E-03
NM_013831	Pstpip2	-1.9	3E-04
NM_153510	Pilra	-1.9	6E-03
NM_008207	H2-T24	-1.9	9E-05
ENSMUST00000028668	Eif3j	-1.9	5E-02
NM_013467	Aldh1a1	-1.8	2E-02
NM_144559	Fcgr4	-1.8	3E-02

NM_001081746	Gm7609	-1.8	5E-04
NM_028572	Vgll3	-1.8	3E-02
ENSMUST00000029935	Gbp3	-1.8	2E-02
NM_145362	Alg1	-1.8	3E-03
NM_001039185	Ceacam1	-1.8	1E-03
NM_021415	Cacna1h	-1.8	1E-03
NM_025644	Exosc1	-1.8	8E-05
ENSMUST00000085050	Serpina3c	-1.8	2E-02
NM_019963	Stat2	-1.8	1E-02
NM_010130	Emr1	-1.8	3E-03
NM_020252	Nrxn1	-1.8	6E-03
XM_003688905	LOC100861626	-1.8	2E-02
NR_027380	Gm3219	-1.8	2E-03
NR_046027	LOC627800	-1.8	2E-02
ENSMUST00000046383	Tnfsf10	-1.8	4E-02
ENSMUST00000031402	Cct6a	-1.8	3E-05
NM_133851	Nusap1	-1.8	1E-02
ENSMUST00000091706	Hfe	-1.8	4E-04
NM_007706	Socs2	-1.8	2E-02
NR_003507	Oas1b	-1.8	1E-02
NM_008008	Fgf7	-1.8	4E-04
NM_021893	Cd274	-1.8	7E-03
NM_011693	Vcam1	-1.8	9E-03
ENSMUST00000169878	Lcp2	-1.8	7E-03
NM_027091	Nup35	-1.8	3E-02
NR_040438	3110045C21Rik	-1.8	3E-02
ENSMUST00000066427	Sp100	-1.8	2E-03
NM_001243039	Gm4070	-1.8	8E-03
NM_011352	Sema7a	-1.8	4E-02
NM_029844	Mrap	-1.8	6E-04
NM_130449	Colec12	-1.8	2E-03
ENSMUST00000033775	Mpp1	-1.8	1E-03
NM_011495	Plk4	-1.8	2E-02
NM_001077189	Fcgr2b	-1.8	1E-03
ENSMUST00000103132	Krt222	-1.8	1E-02
ENSMUST00000030651	Sh3bgrl3	-1.8	7E-04
NM_001081416	Fndc1	-1.8	7E-03
NM_177686	Clec12a	-1.8	2E-03
NM_031159	Apobec1	-1.8	4E-03
Z17401	Igkv1-133	-1.8	5E-02
ENSMUST00000001507	Cyp51	-1.8	7E-03
NM_001033163	Ephx3	-1.8	9E-03
ENSMUST00000029502	Slc16a4	-1.8	5E-03
NM_028035	Snx10	-1.8	3E-02
NM_177591	Igsf1	-1.8	4E-04
NM_008971	Twf1	-1.8	2E-04

ENSMUST00000034808	Nnmt	-1.8	1E-02
NR_030720	Gm8989	-1.8	2E-02
NM_153197	Clec4a3	-1.8	2E-02
NM_001033337	Ttc38	-1.8	3E-04
NM_001083316	Pdgfra	-1.8	1E-02
NM_026878	Rasl11b	-1.8	9E-03
ENSMUST00000062862	Igf1	-1.8	1E-03
NM_001105058	Vmn2r61	-1.8	3E-02
ENSMUST00000165774	Gbp2	-1.7	4E-02
NM_001042611	Cp	-1.7	6E-05
NM_009779	C3ar1	-1.7	2E-03
XR_104918	Gm12676	-1.7	2E-02
NM_016681	Chek2	-1.7	1E-02
NM_009288	Stk10	-1.7	1E-03
NM_001081342	Gpr133	-1.7	3E-02
NM_010874	Nat2	-1.7	1E-03
NM_001110320	Cd72	-1.7	2E-03
NR_028262	Rmst	-1.7	2E-02
NM_173401	Fbxo44	-1.7	1E-03
ENSMUST00000091197	Eif2s3y	-1.7	1E-03
NM_011654	Tuba1b	-1.7	2E-02
NM_012050	Omd	-1.7	3E-02
ENSMUST00000032909	Pde3b	-1.7	3E-02
AK132205	Gm10554	-1.7	9E-03
XM_003688821	Klra11	-1.7	2E-02
NM_025273	Pcbd1	-1.7	9E-03
NM_001001488	Atp8b1	-1.7	9E-03
NM_153408	Neurl3	-1.7	1E-02
NM_018738	Igtp	-1.7	1E-02
NM_001205369	Casc4	-1.7	4E-03
NM_007922	Elk1	-1.7	3E-03
NM_001164327	Gm4902	-1.7	6E-03
NR_037982	9330159M07Rik	-1.7	7E-03
NM_026347	Iah1	-1.7	3E-03
NM_028130	Zfp157	-1.7	9E-04
NM_009917	Ccr5	-1.7	1E-02
NM_145624	Zfp709	-1.7	8E-04
NM_019985	Clec1b	-1.7	4E-02
NM_001177956	Gpm6b	-1.7	2E-03
ENSMUST00000066465	Acsm5	-1.7	6E-03
AK014162	3110040M04Rik	-1.7	4E-02
NM_001159417	Irf9	-1.7	1E-02
NM_009277	Trim21	-1.7	5E-03
NM_008607	Mmp13	-1.7	1E-02
NM_153505	Nckap1l	-1.7	7E-03
NM_178675	Slc35f1	-1.7	3E-02

NM_023043	Prnd	-1.7	2E-02
NM_007778	Csf1	-1.7	2E-02
NM_001161432	Eda2r	-1.7	1E-02
NM_146015	Efemp1	-1.7	3E-03
NM_175674	Vmn2r7	-1.7	3E-02
AK161112	4732440D04Rik	-1.7	2E-02
ENSMUST00000035158	Trf	-1.7	3E-03
NM_028390	Anln	-1.7	5E-03
NM_172759	Ces2e	-1.7	4E-02
ENSMUST00000146439	Tgtp2	-1.7	4E-02
NM_028838	Lrrc2	-1.7	6E-05
XR_106109	Gm13842	-1.7	3E-03
NR_002870	Dnm3os	-1.7	3E-02
ENSMUST00000007248	Hspa1l	-1.7	2E-02
XM_357051	Ms4a14	-1.7	3E-03
NM_016917	Slc40a1	-1.7	1E-02
BC023105	BC023105	-1.7	5E-02
NM_013568	Kcna6	-1.7	7E-03
ENSMUST00000114701	Pi16	-1.7	2E-03
NM_021472	Rnase4	-1.7	2E-02
NM_133226	Pdzd3	-1.7	2E-03
NM_001005358	Zfp960	-1.7	1E-02
NM_152817	Ttc27	-1.7	8E-04
NM_001244651	Gm9041	-1.7	2E-02
NM_144546	Zfp119a	-1.7	4E-02
NM_008362	Il1r1	-1.7	2E-02
ENSMUST00000119047	Tmem128	-1.7	3E-02
NR_045619	Gm9776	-1.7	1E-02
NM_030691	Igsf6	-1.7	3E-03
NM_008517	Lta4h	-1.6	2E-04
ENSMUST00000040128	Atp8b4	-1.6	4E-02
NR_015483	2610203C20Rik	-1.6	3E-02
ENSMUST00000112143	Oasl1	-1.6	1E-03
ENSMUST00000014290	Apbb1ip	-1.6	4E-03
NM_031376	Pik3ap1	-1.6	4E-03
NM_173385	Cilp	-1.6	2E-02
NM_001003948	Pid1	-1.6	2E-03
ENSMUST00000016105	Adss	-1.6	6E-03
NM_207624	Ace	-1.6	2E-02
NM_001004139	Zfp619	-1.6	1E-02
NM_009397	Tnfaip3	-1.6	4E-02
NM_001033228	Itga1	-1.6	1E-03
NM_001085522	Gm13251	-1.6	5E-04
NM_018813	Cpsf3	-1.6	3E-03
ENSMUST00000171811	Vmn2r85	-1.6	1E-02
ENSMUST00000046739	H28	-1.6	9E-03

ENSMUST00000037998	Tram2	-1.6	1E-03
NM_009735	B2m	-1.6	8E-04
ENSMUST00000089926	Mfap1b	-1.6	3E-02
ENSMUST00000067621	Nlrp4g	-1.6	3E-02
ENSMUST00000026571	Irf7	-1.6	8E-03
ENSMUST00000027830	Slamf9	-1.6	8E-03
NM_146896	Olfr1205	-1.6	4E-02
NM_011426	Siglec1	-1.6	9E-03
NM_029495	Epsti1	-1.6	2E-03
NM_001177505	Zfp167	-1.6	1E-02
ENSMUST00000067218	Parp3	-1.6	9E-04
ENSMUST00000012281	Bmp5	-1.6	1E-02
NM_026044	Wdr85	-1.6	3E-02
NM_001170746	Magi2	-1.6	2E-04
NM_001146007	Trim12c	-1.6	4E-02
NM_028756	Slc35a5	-1.6	5E-03
NM_175561	Pcnxl2	-1.6	2E-03
NM_010016	Cd55	-1.6	3E-02
NM_024495	Car13	-1.6	2E-02
NM_007883	Dsg2	-1.6	6E-04
NM_011606	Clec3b	-1.6	9E-03
NM_001142411	Zfp937	-1.6	4E-02
NR_003363	Gm6548	-1.6	4E-03
NM_009728	Atp10a	-1.6	3E-02
NM_011459	Serpinb8	-1.6	5E-02
NM_009515	Was	-1.6	1E-02
NM_026528	2700060E02Rik	-1.6	2E-02
NM_026817	Rabl2	-1.6	8E-03
NM_028813	Vit	-1.6	2E-02
NM_027835	Ifih1	-1.6	5E-03
NM_008035	Folr2	-1.6	1E-02
ENSMUST00000076939	C1qtnf7	-1.6	4E-02
NM_001243943	4930525M21Rik	-1.6	2E-02
ENSMUST00000093802	4922501C03Rik	-1.6	5E-03
NM_027238	Ttc39b	-1.6	5E-02
AK084170	D230004N17Rik	-1.6	3E-02
ENSMUST00000046332	C1qc	-1.6	9E-03
NM_133228	Zfp87	-1.6	4E-03
NR_033498	AI504432	-1.6	5E-02
NM_001034862	Erich1	-1.6	7E-03
NM_174848	Crybg3	-1.6	2E-03
NM_001101471	Akap5	-1.6	6E-03
NM_173381	6720489N17Rik	-1.6	2E-02
NM_172508	Dse	-1.6	1E-02
ENSMUST00000115443	Met	-1.6	1E-02
NM_138674	Pkhd1l1	-1.6	3E-02

NM_010779	Mcpt4	-1.6	5E-02
NM_010917	Nid1	-1.6	4E-03
NM_145585	Thumpd1	-1.6	1E-02
NR_040630	LOC626049	-1.6	5E-02
AK138253	A230045G11Rik	-1.6	1E-02
NM_026174	Entpd4	-1.6	2E-03
NM_001164578	Tsr2	-1.6	5E-02
NM_145494	Me2	-1.6	5E-03
NM_021529	Ppp2r3c	-1.6	7E-03
NR_028261	Rian	-1.6	2E-02
NM_183168	P2ry6	-1.6	7E-03
XR_141558	Gm19624	-1.6	4E-03
NM_011271	Rnase1	-1.6	6E-03
NR_029567	Mir129-1	-1.6	2E-02
NM_134246	Acot3	-1.6	4E-02
NR_040463	9230112J17Rik	-1.6	6E-03
NM_026644	Agpat4	-1.6	3E-03
NM_025341	Abhd6	-1.6	4E-03
NM_011610	Tnfrsf1b	-1.6	3E-03
NM_011758	Zfp39	-1.6	2E-04
NM_010511	Ifngr1	-1.6	5E-03
ENSMUST00000117194	Cep164	-1.6	2E-02
NM_146391	Olfr1058	-1.6	7E-03
NM_001163020	Klhl32	-1.6	4E-03
NM_011782	Adamts5	-1.6	3E-02
ENSMUST00000056398	Naa38	-1.6	3E-04
BC022960	BC022960	-1.6	5E-04
ENSMUST00000044155	Ubash3b	-1.6	2E-02
NM_001039088	Seh1l	-1.6	5E-05
NM_025934	Riok2	-1.6	5E-02
NM_001190466	Dact1	-1.6	9E-03
NM_139138	Emr4	-1.6	1E-02
NM_001243032	Gm16445	-1.6	5E-02
NM_144538	Rab3il1	-1.6	1E-03
NR_045001	Gm6936	-1.6	3E-02
NM_028812	Gtf2e1	-1.6	3E-02
NM_001166709	Vmn1r207-ps	-1.6	1E-02
NR_029916	Mir411	-1.6	3E-02
NM_145418	BC013529	-1.6	7E-03
NM_027060	Btbd9	-1.6	4E-03
NM_010791	Meox1	-1.6	5E-02
NR_003960	Gm5478	-1.6	4E-02
NM_001005421	Amica1	-1.6	1E-02
NM_001146046	Lrrc49	-1.6	4E-02
NR_015502	E030003E18Rik	-1.6	4E-02
NM_133993	Pwp1	-1.6	2E-03

NM_008985	Ptprn	-1.6	3E-02
NM_011331	Ccl12	-1.6	5E-02
NM_010188	Fcgr3	-1.6	7E-03
NM_177653	F830045P16Rik	-1.6	3E-02
ENSMUST00000090986	Fcrls	-1.6	2E-03
NM_145532	Mall	-1.6	5E-03
NM_146205	Armc5	-1.6	3E-02
NM_001033149	Ttc9	-1.6	3E-02
ENSMUST00000019067	Med11	-1.6	2E-02
NM_199015	D14Ertd668e	-1.6	4E-02
NM_001099217	Ly6c2	-1.6	2E-02
NM_013590	Lyz1	-1.6	4E-02
NM_001012324	Ecm2	-1.6	1E-02
NM_145575	Cald1	-1.6	1E-02
NM_008332	Ifit2	-1.6	1E-02
ENSMUST00000026274	Lztfl1	-1.6	1E-03
NM_177070	Fbxw16	-1.6	5E-02
NM_010070	Dok1	-1.6	2E-02
NM_030731	Trim23	-1.6	2E-02
NM_011774	Slc30a4	-1.6	6E-03
NM_001081249	Vcan	-1.6	3E-02
NR_027875	1810058I24Rik	-1.6	3E-02
NM_001099634	Myof	-1.6	2E-02
NM_147009	Olfr389	-1.6	5E-02
NR_045190	E330017L17Rik	-1.5	4E-03
AK019562	4930404H24Rik	-1.5	3E-02
ENSMUST00000109503	Nfam1	-1.5	2E-02
NM_146144	Usp1	-1.5	7E-03
NR_028420	Zfp809	-1.5	2E-03
ENSMUST00000113480	Cysltr1	-1.5	3E-02
NM_001001185	BC048507	-1.5	4E-02
NM_080288	Elmo1	-1.5	3E-02
NM_011660	Txn1	-1.5	3E-02
NM_027976	Acsl5	-1.5	8E-03
NM_175266	Epm2aip1	-1.5	2E-04
NM_011815	Fyb	-1.5	1E-02
NM_001166581	BC005561	-1.5	1E-02
NM_011851	Nt5e	-1.5	2E-03
NR_033729	5730577I03Rik	-1.5	2E-02
ENSMUST00000028045	Mrc1	-1.5	7E-03
NM_133199	Scn4a	-1.5	2E-02
NM_172784	Lrp11	-1.5	3E-02
NM_146758	Olfr678	-1.5	1E-02
NM_001168615	Tifab	-1.5	1E-02
NM_001001152	Zfp458	-1.5	2E-02
BC004016	Uck2	-1.5	4E-02

NM_152821	Purg	-1.5	8E-03
NM_001243118	2010315B03Rik	-1.5	2E-02
BC052634	Wdyhv1	-1.5	4E-02
NM_001113413	Rnf13	-1.5	8E-03
NM_172578	Mis18bp1	-1.5	9E-03
NM_177755	Klhl38	-1.5	7E-03
NM_001111096	Lyn	-1.5	2E-03
NM_181547	Nostrin	-1.5	2E-02
ENSMUST00000080880	Dab2	-1.5	5E-03
NM_009135	Scn7a	-1.5	1E-02
NM_011775	Zp2	-1.5	4E-03
NM_178924	Upk1b	-1.5	5E-02
ENSMUST00000112836	Amot	-1.5	6E-03
ENSMUST00000102942	Psd4	-1.5	3E-02
NM_019549	Plek	-1.5	1E-02
NM_008304	Sdc2	-1.5	2E-03
NM_030715	Polh	-1.5	2E-02
NM_010400	H60a	-1.5	4E-02
BC016429	D6Wsu163e	-1.5	3E-04
NM_008420	Kcnb1	-1.5	7E-04
NM_016886	Gria3	-1.5	4E-02
NM_019759	Dpt	-1.5	2E-03
NM_134080	Flnb	-1.5	2E-02
ENSMUST00000102768	Rbm43	-1.5	1E-02
NM_133198	Pygl	-1.5	1E-03
NM_130448	Pcdh18	-1.5	3E-03
NM_026632	Rpa3	-1.5	7E-03
NM_019656	Tspan6	-1.5	1E-02
NM_177111	Ccdc66	-1.5	7E-03
XR_106549	Gm3515	-1.5	9E-03
NM_025806	Plbd1	-1.5	5E-03
NM_001163548	Cyth3	-1.5	4E-02
NM_010999	Olfr56	-1.5	6E-03
NM_001079844	Gpc6	-1.5	2E-02
AK015294	4930432L08Rik	-1.5	6E-03
NM_134167	Vmn1r35	-1.5	2E-02
NM_008869	Pla2g4a	-1.5	3E-02
ENSMUST00000002360	Angptl4	-1.5	2E-02
NM_025390	Pop4	-1.5	4E-03
ENSMUST00000037086	Fer1l6	-1.5	5E-03
NR_040355	1700029I01Rik	-1.5	4E-03
NM_028634	Cby1	-1.5	3E-03
NM_145531	Spg11	-1.5	4E-04
NM_007630	Ccnb2	-1.5	2E-02
NM_001039039	Kctd21	-1.5	2E-02
NM_023284	Nuf2	-1.5	2E-02

NR_000040	Tyms-ps	-1.5	2E-02
ENSMUST00000034591	Bace1	-1.5	1E-03
NM_023348	Snap29	-1.5	3E-03
NR_040328	4833419F23Rik	-1.5	9E-04
NM_011629	Nr2c1	-1.5	4E-03
NM_173740	Maoa	-1.5	8E-03
NM_001177529	Gm5494	-1.5	1E-02
ENSMUST00000022781	Dad1	-1.5	7E-03
NM_028352	Pgm3	-1.5	3E-02
ENSMUST00000054072	Zfp81	-1.5	4E-02
NM_010738	Ly6a	-1.5	3E-02
NR_040446	C920021L13Rik	-1.5	3E-03
NM_025914	Actr6	-1.5	1E-02
ENSMUST00000032183	Tmem43	-1.5	6E-03
NM_008228	Hdac1	-1.5	2E-02
NM_011748	Zfp14	-1.5	3E-02
ENSMUST00000018610	Nos2	-1.5	3E-02
NM_013594	Mbd1	-1.5	1E-02
NM_178732	Zfp324	-1.5	2E-02
NM_144818	Ncaph	-1.5	2E-04
NM_175328	Slc6a15	-1.5	2E-03
ENSMUST00000002436	Snx9	-1.5	8E-04
NM_001035239	Trpm3	-1.5	2E-03
NM_011074	Cdk14	-1.5	2E-02
NM_146009	Cep290	-1.5	1E-02
ENSMUST00000093193	Dock2	-1.5	2E-03
NR_040741	1700027I24Rik	-1.5	4E-02
NM_010762	Mal	-1.5	2E-02
AK008823	4930555A03Rik	-1.5	4E-02
NR_003643	1700123L14Rik	-1.5	5E-02
AK173199	Rnf213	-1.5	2E-02
ENSMUST00000064667	Rap1b	-1.5	4E-03
NM_007631	Ccnd1	-1.5	4E-02
NM_134420	Slc26a6	-1.5	3E-02
NM_024242	Riok1	-1.5	2E-03
NM_011212	Ptpre	-1.5	2E-03
NM_025442	Alg5	-1.5	6E-03
NM_008760	Ogn	-1.5	2E-02
NM_009801	Car2	-1.5	2E-02
NR_045613	Zfp572	-1.5	2E-02
NM_027000	Gtpbp4	-1.5	1E-02
ENSMUST00000171433	Mid1	-1.5	1E-02
ENSMUST00000042665	Parp14	-1.5	1E-02
NM_053229	Vmn1r46	-1.5	4E-02
NM_026658	Mto1	-1.5	4E-03
NM_175291	Dock10	-1.5	9E-03

NM_028036	Tmco6	-1.5	1E-02
ENSMUST00000028119	Mastl	-1.5	8E-03
NM_010001	Cyp2c37	-1.5	8E-03
NM_146609	Olfr150	-1.5	4E-02
NR_026741	Gm14379	-1.5	3E-02
XM_001477447	Gm9507	-1.5	4E-02
NM_138751	Tmem47	-1.5	2E-03
ENSMUST00000067888	Tpk1	-1.5	2E-02
NM_181588	Cmbl	-1.5	2E-02
NR_045899	AU016765	-1.5	3E-03
NM_010185	Fcer1g	-1.5	4E-02
NM_026805	Svop	-1.5	3E-02
NM_001011684	Nms	-1.5	4E-02
ENSMUST00000025065	Nudt12	-1.5	4E-02
NM_026670	Zmym1	-1.5	1E-02
XR_107558	Gm19708	-1.5	4E-02
ENSMUST00000064783	Mfap4	-1.5	2E-02
NM_010700	Ldlr	-1.5	1E-02
NM_007658	Cdc25a	-1.5	3E-03
ENSMUST00000132967	Fsip2	-1.5	3E-02
NM_015784	Postn	-1.5	3E-02
ENSMUST00000096350	Maff	-1.5	4E-02
NM_009653	Alas2	-1.5	2E-02
NM_019978	Dclk1	-1.5	2E-02
NR_045470	1110028F18Rik	-1.5	3E-02
NM_028889	Efhd1	-1.5	4E-02
NM_027973	Mlf1ip	-1.5	4E-02
NM_146224	Zfp280d	-1.5	5E-04
ENSMUST00000023396	Pmm2	-1.5	3E-02
NM_001177759	Tnfaip8	-1.5	2E-02
NM_008800	Pde1b	-1.5	2E-03
AK046316	B230369F24Rik	-1.5	3E-03
ENSMUST00000165600	Dlg2	-1.5	2E-02
NM_001159518	Igfbp7	-1.5	1E-02
ENSMUST00000112057	Klrc2	-1.5	1E-02
NM_013819	H2-M3	-1.5	4E-03
NM_010877	Ncf2	-1.5	3E-03
NM_054049	Osr2	-1.5	1E-02
NM_025844	Chordc1	-1.5	2E-02
NM_146748	Olfr661	-1.5	4E-03
NM_001177943	Eda	-1.5	3E-02
NM_153069	Leap2	-1.5	4E-02
NM_016710	Hmgn5	-1.5	3E-02
ENSMUST00000102946	Exoc2	-1.5	6E-05
NM_181395	Pxdn	-1.5	2E-02
NR_046075	Gm4251	-1.4	5E-02

ENSMUST00000106364	Coro1a	-1.4	1E-02
ENSMUST00000146165	D3Ertd751e	-1.4	4E-03
NM_028658	Klraq1	-1.4	2E-02
NM_178589	Tnfrsf21	-1.4	4E-02
NM_146133	Golph3l	-1.4	4E-03
NM_001001892	H2-K1	-1.4	9E-03
NM_020614	Taf1b	-1.4	5E-03
ENSMUST00000103234	Fbn1	-1.4	4E-02
NM_011997	Casp8ap2	-1.4	1E-02
NM_029761	Dok5	-1.4	1E-02
NM_021449	Crbn	-1.4	2E-02
ENSMUST00000113422	Hdx	-1.4	7E-03
NM_001033711	Evi2a	-1.4	3E-02
NM_001007583	Best3	-1.4	1E-02
NR_040265	2310001H17Rik	-1.4	6E-03
NM_177474	D19Bwg1357e	-1.4	2E-03
NR_015467	5530601H04Rik	-1.4	1E-02
NM_009202	Slc22a1	-1.4	4E-02
NM_133704	Sec22a	-1.4	1E-02
ENSMUST00000115083	Meig1	-1.4	2E-02
ENSMUST00000096066	Cpa2	-1.4	5E-03
NM_145591	Zfp958	-1.4	4E-02
ENSMUST00000102513	Pla2g5	-1.4	1E-02
NM_001025360	Klc1	-1.4	9E-04
NM_153820	Arhgap15	-1.4	3E-02
ENSMUST00000020448	Irak3	-1.4	4E-02
NM_001081239	Lilra5	-1.4	2E-02
NM_001080818	Cdc14a	-1.4	2E-02
NM_172672	Ganc	-1.4	9E-03
NM_007376	Pzp	-1.4	1E-02
NM_001162870	Spast	-1.4	7E-03
ENSMUST00000035053	Nme6	-1.4	1E-02
NM_001038710	Tmod2	-1.4	2E-02
ENSMUST00000067532	Ms4a7	-1.4	4E-02
NM_009194	Slc12a2	-1.4	9E-03
NM_028195	Cyth4	-1.4	1E-02
NM_144933	Med17	-1.4	2E-03
NM_029631	Abhd14b	-1.4	2E-02
XR_140777	Gm6637	-1.4	5E-02
NM_007473	Aqp7	-1.4	2E-02
NM_009029	Rb1	-1.4	1E-04
NR_046062	Gm10640	-1.4	2E-02
NM_001256100	Mtif3	-1.4	1E-02
NM_001033136	Fam82a2	-1.4	4E-02
NM_019914	Mllt11	-1.4	5E-03
ENSMUST00000045748	Pdk3	-1.4	3E-02

ENSMUST00000023312	Alcam	-1.4	3E-02
NM_001173550	C5ar1	-1.4	3E-02
NM_001033435	Gm885	-1.4	9E-03
NM_178736	Elmod2	-1.4	4E-02
ENSMUST00000067230	Sox4	-1.4	5E-02
NM_201637	Chd8	-1.4	1E-02
NM_026585	D6Wsu116e	-1.4	8E-03
NM_175437	Pion	-1.4	1E-02
ENSMUST00000075452	Chic2	-1.4	2E-02
XM_003688767	Tcf24	-1.4	4E-02
NM_025819	1200016B10Rik	-1.4	5E-02
ENSMUST00000032843	Tmem126b	-1.4	2E-02
NM_198012	Trim68	-1.4	4E-03
NM_029730	Mospd2	-1.4	2E-03
NM_023245	Palmd	-1.4	5E-03
ENSMUST00000177403	Ppp1r3e	-1.4	2E-02
NM_016723	Uchl3	-1.4	3E-02
ENSMUST00000171445	Eps8l1	-1.4	6E-03
NM_001163527	Itpripl1	-1.4	2E-02
NM_175460	Nmnat2	-1.4	2E-02
ENSMUST00000085358	Tex9	-1.4	1E-02
NM_025408	Acer3	-1.4	2E-02
ENSMUST00000027876	Scyl3	-1.4	2E-02
NM_001167988	Taf9b	-1.4	2E-02
ENSMUST00000115243	Opcml	-1.4	2E-03
NM_016658	Galt	-1.4	2E-02
NM_026775	Tmed10	-1.4	2E-04
NM_026327	1810048J11Rik	-1.4	1E-02
NM_172482	Zfp719	-1.4	4E-03
NM_001081402	Wdr70	-1.4	2E-02
NM_001013577	1110054O05Rik	-1.4	1E-02
NM_008216	Has2	-1.4	3E-02
ENSMUST00000016338	Hsd11b1	-1.4	4E-02
NM_153552	Thoc1	-1.4	2E-02
NR_045337	2310039L15Rik	-1.4	2E-02
ENSMUST00000033277	2310008H09Rik	-1.4	3E-03
NM_201361	Fam82a1	-1.4	2E-02
NM_023906	Asb3	-1.4	4E-02
NM_153591	Nars2	-1.4	2E-02
NM_009155	Sepp1	-1.4	4E-02
NM_013666	St8sia5	-1.4	1E-02
NM_134063	BC016423	-1.4	1E-03
NM_001038696	Rnpc3	-1.4	8E-03
ENSMUST00000020081	Zwint	-1.4	3E-02
XR_141546	LOC100862482	-1.4	2E-02
ENSMUST00000174518	Aldh7a1	-1.4	1E-02

ENSMUST00000047131	Ipo4	-1.4	8E-04
NM_001113179	Bub1	-1.4	2E-02
NM_011407	Slfn1	-1.4	4E-03
NM_001081077	Cwf19l1	-1.4	9E-03
NM_025445	Arfgap3	-1.4	3E-02
NR_040740	4930596M17Rik	-1.4	4E-02
ENSMUST00000029907	Ubxn2b	-1.4	4E-02
NR_045889	Gm10791	-1.4	4E-02
NM_009382	Thy1	-1.4	5E-02
ENSMUST00000110003	Eif4e1b	-1.4	2E-02
NM_027017	3300002I08Rik	-1.4	4E-02
NM_025961	Gatm	-1.4	4E-02
NR_037269	Mir466n	-1.4	5E-02
NM_178309	Brip1	-1.4	1E-02
NM_172604	Scara3	-1.4	2E-02
NM_001013758	Lingo3	-1.4	4E-02
NM_008396	Itga2	-1.4	2E-02
NM_029977	Polq	-1.4	3E-02
NM_145511	BC003331	-1.4	6E-03
ENSMUST00000001711	Hoxc6	-1.4	4E-02
ENSMUST00000102915	Smc2	-1.4	1E-02
NM_023788	Mageh1	-1.4	5E-02
NM_172924	C230081A13Rik	-1.4	8E-03
NM_001025386	Olfr243	-1.4	5E-02
NM_145375	Tm6sf1	-1.4	4E-02
ENSMUST00000163336	Ncoa4	-1.4	2E-02
NM_019671	Net1	-1.4	1E-02
NM_172086	Rpl32	-1.4	1E-03
NM_001146022	Wdfy4	-1.4	1E-02
NM_172964	Arhgap28	-1.4	2E-02
NM_146661	Olfr1112	-1.4	1E-02
NM_001102607	Col6a6	-1.4	1E-03
NM_011721	Wrn	-1.4	3E-02
NM_001081350	Nol8	-1.4	3E-03
ENSMUST00000114126	Stx18	-1.4	2E-02
NM_029492	Zdhhc20	-1.4	1E-02
ENSMUST00000108205	Zfp74	-1.4	4E-02
NM_175275	Cntln	-1.4	9E-03
ENSMUST00000110214	Gm6871	-1.4	4E-02
NM_020008	Clec7a	-1.4	2E-02
NM_008055	Fzd4	-1.4	3E-02
NM_029270	Arhgap24	-1.4	5E-03
ENSMUST00000026461	Prim1	-1.4	5E-03
NM_007836	Gadd45a	-1.4	2E-02
ENSMUST00000112063	Klrd1	-1.4	2E-02
NM_009661	Alox8	-1.4	3E-02

NM_134138	Psmg2	-1.4	4E-03
NM_028000	Ppapdc1b	-1.4	9E-03
NM_001039231	Zfp951	-1.4	2E-02
NM_177841	4932418E24Rik	-1.4	3E-02
NM_172420	Ppp1r1c	-1.4	4E-02
ENSMUST00000023760	Gpd1	-1.4	1E-02
NM_001033375	A230046K03Rik	-1.4	3E-03
ENSMUST00000029650	Ints12	-1.4	1E-02
ENSMUST00000112969	Paqr3	-1.4	3E-02
NM_054090	Olfr73	-1.4	4E-02
NM_022563	Ddr2	-1.4	4E-02
NM_001199330	AI987944	-1.4	4E-02
NM_028428	Fut11	-1.4	3E-03
NM_001033534	Layn	-1.4	1E-02
NM_026302	Dctn4	-1.4	4E-04
NM_178638	Tmem108	-1.4	5E-02
NM_023395	Wfdc1	-1.4	3E-02
NR_033609	A930017M01Rik	-1.4	4E-02
NM_010296	Gli1	-1.4	4E-02
BC147292	1810049H13Rik	-1.4	4E-02
NM_001160163	Neu2	-1.4	2E-02
NM_011132	Pole	-1.4	6E-03
ENSMUST00000000299	Itgb2	-1.4	3E-03
NM_001081961	2300005B03Rik	-1.4	2E-02
NM_009663	Alox5ap	-1.4	1E-02
NM_001033261	Zfc3h1	-1.4	2E-02
NM_053126	Pcdhb1	-1.4	3E-02
NM_174990	Gimap4	-1.4	6E-03
NM_007943	Eps15	-1.4	1E-02
ENSMUST00000022429	Arf4	-1.4	4E-04
ENSMUST00000044850	Abca9	-1.4	5E-02
NM_010407	Hck	-1.4	5E-02
NM_026070	Ccdc53	-1.4	4E-04
ENSMUST00000102601	Trappc1	-1.4	3E-02
AK157155	Gm10733	-1.4	4E-02
ENSMUST00000122064	Emcn	-1.4	4E-02
NM_011546	Zeb1	-1.4	2E-02
ENSMUST00000019734	Cyb561	-1.4	9E-03
ENSMUST00000028062	Vim	-1.4	2E-02
ENSMUST00000041708	Gpr34	-1.4	4E-02
NM_001168294	Serpina3f	-1.4	2E-02
NM_177782	Prex1	-1.4	2E-02
NM_011706	Trpv2	-1.4	2E-03
NM_001080811	Bpifa6	-1.4	3E-02
NM_028044	Cnn3	-1.4	4E-02
NM_011737	Ysk4	-1.4	5E-03

NM_033320	Glce	-1.4	2E-03
NM_008403	Itgb1bp1	-1.4	5E-03
ENSMUST00000039557	Arhgap18	-1.4	1E-02
ENSMUST00000094097	Tmem41b	-1.4	1E-02
NM_010431	Hif1a	-1.4	1E-02
NM_024178	Alg14	-1.4	5E-03
ENSMUST00000108112	Spata5	-1.4	7E-03
NM_007825	Cyp7b1	-1.4	4E-02
ENSMUST00000027151	Myl1	-1.4	1E-02
NM_001033249	Zfp583	-1.4	3E-02
ENSMUST00000027139	Wdr75	-1.4	1E-02
NM_010323	Gnrhr	-1.4	4E-02
NM_001025102	2700007P21Rik	-1.4	4E-03
NM_007643	Cd36	-1.4	4E-05
NM_011739	Ywhaq	-1.4	2E-03
NM_145400	Ube4a	-1.4	5E-03
NM_010088	Prl8a2	-1.4	5E-02
NR_045884	Gm10485	-1.4	3E-02
NM_001033416	Gal3st4	-1.4	2E-02
BC035042	2610029G23Rik	-1.4	3E-02
NM_026532	Nutf2	-1.4	2E-03
NM_018797	Plxnc1	-1.4	4E-02
XR_105469	Gm20245	-1.4	3E-02
ENSMUST00000030451	Toe1	-1.4	2E-02
NM_027494	Zcchc8	-1.4	2E-02
NM_007746	Map3k8	-1.4	1E-02
NM_008395	Itch	-1.4	2E-02
NM_145534	Btbd3	-1.4	7E-03
NM_023041	Pex19	-1.4	6E-03
NM_177393	Nalcn	-1.4	1E-02
NM_001113211	Tmem194	-1.4	5E-02
NM_153774	Ipo9	-1.4	3E-03
NM_172575	Zfp277	-1.4	3E-02
NM_172689	Ddx58	-1.4	2E-02
NM_011421	Smpd1	-1.4	1E-02
NM_026040	Srfbp1	-1.4	1E-02
NM_021274	Cxcl10	-1.4	2E-02
NM_207237	Man1c1	-1.4	3E-02
NM_026511	2810002N01Rik	-1.4	4E-02
NM_009794	Capn2	-1.4	3E-02
NM_029842	Jmjd5	-1.4	3E-03
NR_033634	A630075F10Rik	-1.4	2E-02
AK132067	Gm2449	-1.4	4E-02
NM_001039218	Spink14	-1.4	3E-02
NM_021460	Lipa	-1.4	2E-02
ENSMUST00000039165	Golga1	-1.4	5E-03

ENSMUST00000029316	Exosc8	-1.4	2E-02
ENSMUST00000011029	Gm15118	-1.4	4E-02
NR_038040	C730027H18Rik	-1.4	1E-02
ENSMUST00000015901	Ppil4	-1.4	4E-02
NM_013693	Tnf	-1.4	4E-02
XR_105313	Gm19667	-1.4	3E-02
NR_035490	Mir1964	-1.4	2E-02
NM_080445	B3galt6	-1.4	1E-02
NM_172734	Stk38l	-1.4	5E-03
NM_001163640	Chn2	-1.4	1E-02
NM_013896	Timm9	-1.4	2E-02
NM_001247984	Lcp1	-1.4	7E-03
NM_001081111	Tmf1	-1.4	5E-03
NM_146033	Ankmy2	-1.4	1E-02
NM_025514	Anapc16	-1.4	1E-02
NM_001172123	Rbms3	-1.4	9E-03
ENSMUST00000058669	Gnat2	-1.4	6E-03
NM_001005223	Znhit3	-1.4	4E-02
ENSMUST00000028921	Xrn2	-1.4	4E-03
NM_001111107	Zfp322a	-1.4	4E-03
ENSMUST00000026972	Fam20c	-1.4	2E-02
XM_003086086	Gm4701	-1.4	3E-02
ENSMUST00000033609	Cstf2	-1.4	2E-02
NM_001135192	Asap2	-1.4	3E-02
NM_146960	Olfr53	-1.4	2E-02
NM_025433	Rpl7l1	-1.4	1E-02
ENSMUST00000172171	Naalad2	-1.4	1E-02
NM_026552	Arpc4	-1.4	5E-02
NM_019517	Bace2	-1.4	9E-03
NR_015548	5830432E09Rik	-1.4	6E-04
NM_019535	Sh3gl2	-1.4	3E-02
AK006360	1700025N21Rik	-1.4	7E-03
ENSMUST00000109790	Asxl1	-1.4	1E-02
NM_001167963	G2e3	-1.4	1E-02
NM_008861	Pkd2	-1.4	2E-02
NM_001012309	Ccdc55	-1.4	2E-02
NM_009786	Cacybp	-1.4	1E-02
NM_018749	Eif3d	-1.4	5E-03
NM_178087	Pml	-1.4	3E-02
ENSMUST00000074525	Gulp1	-1.4	3E-02
NM_027294	Cmtm8	-1.4	3E-03
NR_003555	Vmn2r29	-1.4	4E-03
AK040372	Gm10002	-1.4	4E-02
NM_172713	Sdad1	-1.4	1E-02
NM_001162922	Zfp931	-1.4	2E-02
XR_106694	Gm4419	-1.4	3E-03

NM_009546	Trim25	-1.4	4E-02
NM_001254745	E030030I06Rik	-1.4	3E-02
NM_009097	Rps6ka1	-1.4	2E-02
NM_001205396	Gprc5d	-1.4	4E-02
NM_011550	Mlx	-1.4	2E-02
NM_027135	Sec24d	-1.4	7E-04
NM_023233	Trim13	-1.4	2E-02
ENSMUST00000102642	Ube2l6	-1.3	1E-03
NM_019773	Rab9	-1.3	4E-02
NM_001081378	Kidins220	-1.3	3E-03
NM_024195	Cyb5r4	-1.3	2E-02
ENSMUST00000091752	Hist2h3b	-1.3	2E-02
NM_183201	Slfn5	-1.3	4E-02
NR_045728	Gm16617	-1.3	3E-02
NR_038017	Gm15915	-1.3	3E-02
NM_026849	Mtmr14	-1.3	8E-03
NM_028841	Tspan17	-1.3	2E-02
NM_027260	Vrk2	-1.3	3E-02
NM_145137	Mgl2	-1.3	1E-02
NM_145933	St6gal1	-1.3	3E-02
NM_001256059	Ccdc149	-1.3	2E-02
BC119608	Phxr1	-1.3	5E-02
ENSMUST00000112386	Rab7l1	-1.3	7E-03
NM_133702	Nol11	-1.3	4E-03
NM_031843	Dpp7	-1.3	5E-02
NM_024250	Phf10	-1.3	5E-03
NR_028265	Mirg	-1.3	4E-02
NM_133762	Ncapg2	-1.3	2E-02
NM_010073	Dpm2	-1.3	4E-02
NM_019553	Ddx21	-1.3	6E-03
NM_008105	Gcnt2	-1.3	3E-02
NM_175501	Adamts12	-1.3	4E-02
AK038602	A930006K02Rik	-1.3	1E-02
NM_009682	Ap3s2	-1.3	2E-02
NM_001170954	A4galt	-1.3	5E-02
XR_142216	Gm6929	-1.3	1E-03
ENSMUST00000107364	Rab5c	-1.3	3E-02
NM_134034	Smek2	-1.3	4E-02
ENSMUST00000033642	Dcx	-1.3	3E-02
NM_013469	Anxa11	-1.3	1E-03
NM_001025365	Miip	-1.3	3E-02
NM_019932	Pf4	-1.3	3E-02
NM_001164087	Homer2	-1.3	3E-02
ENSMUST00000035164	Topbp1	-1.3	2E-02
NM_010128	Emp1	-1.3	4E-02
NM_177814	Erc2	-1.3	3E-02

NM_023738	Uba7	-1.3	2E-02
NM_010217	Ctgf	-1.3	5E-02
NM_009329	Zfp354a	-1.3	1E-02
AK052542	D430040D24Rik	-1.3	2E-02
NM_025539	Nudt2	-1.3	2E-02
NM_133781	Cab39	-1.3	1E-02
NM_025965	Ssr1	-1.3	2E-03
NM_027534	Kdsr	-1.3	1E-02
NM_010946	Ntan1	-1.3	4E-03
NM_016860	Actr1a	-1.3	2E-03
NM_152894	Pop1	-1.3	2E-03
ENSMUST00000090178	Dnajb14	-1.3	1E-02
NM_029879	Rgs7bp	-1.3	1E-02
ENSMUST00000162686	1110034B05Rik	-1.3	2E-02
NM_011755	Zfp35	-1.3	2E-02
NR_040543	4930406D18Rik	-1.3	1E-02
NM_183310	Fam190a	-1.3	2E-02
NM_001166651	Klhl33	-1.3	3E-02
NM_001165980	Dcaf17	-1.3	1E-02
ENSMUST00000038474	Exosc2	-1.3	3E-02
NM_053176	Hrg	-1.3	4E-02
NM_138758	Tmlhe	-1.3	3E-02
ENSMUST00000040917	Rps27l	-1.3	3E-03
NM_019635	Stk3	-1.3	2E-03
NM_024182	Riok3	-1.3	5E-03
ENSMUST00000071077	Spata18	-1.3	1E-02
NM_026611	Rnaset2b	-1.3	3E-04
ENSMUST00000028914	Polr3f	-1.3	7E-03
NM_001033422	Thoc2	-1.3	6E-03
NM_031156	Ide	-1.3	5E-02
NM_008225	Hcls1	-1.3	8E-03
NM_175451	Ckap4	-1.3	4E-02
NM_007683	Cenpc1	-1.3	1E-02
NM_019428	Rpp30	-1.3	2E-02
NM_198017	Fam175b	-1.3	4E-02
AK012238	2700012I20Rik	-1.3	3E-03
AK141242	Gm10856	-1.3	2E-02
NM_026386	Snx2	-1.3	8E-04
NM_133222	Eltd1	-1.3	1E-02
NM_026866	Disp1	-1.3	4E-04
NM_207301	Wrb	-1.3	1E-02
ENSMUST00000033427	Sash3	-1.3	2E-02
ENSMUST00000000058	Cav2	-1.3	3E-02
ENSMUST00000116522	Nck1	-1.3	5E-02
NM_001081316	Dsel	-1.3	1E-02
NM_133200	P2ry14	-1.3	3E-02

ENSMUST00000035264	Pak7	-1.3	4E-02
NM_001081304	Atf6	-1.3	3E-03
NM_001135577	BC024659	-1.3	4E-02
NM_145468	Skp2	-1.3	4E-02
ENSMUST00000003469	Cd79a	-1.3	2E-02
ENSMUST00000165104	Gramd1b	-1.3	3E-02
NM_178644	Oaf	-1.3	4E-02
NM_001205361	Dcun1d1	-1.3	1E-02
NM_001200023	Zfp963	-1.3	5E-02
ENSMUST00000071921	Dmtf1	-1.3	2E-02
NM_001243161	Alg11	-1.3	5E-03
NM_001177544	Hist1h2ao	-1.3	3E-02
NM_010086	Adam24	-1.3	1E-02
NM_025997	Fam103a1	-1.3	1E-02
NM_001204912	2410017P09Rik	-1.3	5E-02
NM_144843	Mtmr6	-1.3	5E-03
NM_001199004	Golga5	-1.3	1E-02
NM_019787	Sec23b	-1.3	2E-02
BC034664	Lyrm4	-1.3	5E-04
ENSMUST00000112736	Vps4b	-1.3	2E-02
NM_001081099	2610002D18Rik	-1.3	4E-02
ENSMUST00000108935	Etohi1	-1.3	2E-02
NM_181277	Col14a1	-1.3	3E-02
NM_027008	Kctd5	-1.3	2E-02
NM_023805	Slc38a3	-1.3	4E-02
NM_177260	Tmem154	-1.3	4E-02
NM_008804	Pde9a	-1.3	7E-03
NM_026964	Ccdc124	-1.3	3E-02
NM_009185	Stil	-1.3	3E-02
AK048710	C230014O12Rik	-1.3	5E-02
NM_028761	Parn	-1.3	3E-03
NM_018737	Ctps2	-1.3	7E-03
NM_010422	Hexb	-1.3	3E-02
NM_010065	Dnm1	-1.3	4E-02
NM_018869	Grk5	-1.3	3E-02
NM_001083319	Ubp1	-1.3	5E-03
ENSMUST00000035721	Prpf18	-1.3	8E-03
NM_001177543	0610010B08Rik	-1.3	3E-03
XR_105528	Gm19865	-1.3	3E-02
NM_019868	Hnrnph2	-1.3	3E-03
ENSMUST00000112221	Rad51ap1	-1.3	4E-02
NM_025286	Slc31a2	-1.3	1E-02
NM_173757	Mrps27	-1.3	3E-02
NM_008729	Ctnnd2	-1.3	5E-02
NR_033518	Gm16039	-1.3	5E-02
NM_177859	Aknad1	-1.3	6E-03

ENSMUST00000036210	Poglut1	-1.3	3E-02
NM_001080129	Tmpo	-1.3	8E-03
NM_172253	Twistnb	-1.3	4E-02
ENSMUST00000055303	Mettl6	-1.3	2E-02
NM_175283	Srd5a1	-1.3	4E-02
NM_001163154	Etv1	-1.3	3E-03
ENSMUST00000030586	Ccdc28b	-1.3	4E-02
NM_027166	Ypel5	-1.3	4E-02
NM_001014423	Abi3bp	-1.3	2E-02
NM_001081695	Dnmt3l	-1.3	1E-02
ENSMUST00000005964	Adh5	-1.3	2E-02
NM_001039514	Dhps	-1.3	2E-02
NM_001077499	Scn8a	-1.3	2E-02
BC080301	2210418O10Rik	-1.3	1E-02
NM_197959	Kif18b	-1.3	4E-02
NM_008960	Pten	-1.3	6E-03
ENSMUST00000023357	0610037P05Rik	-1.3	4E-02
NM_194339	Bms1	-1.3	4E-04
NR_028429	Thap6	-1.3	9E-03
NM_028768	Armc8	-1.3	4E-03
NR_029778	Mir101b	-1.3	4E-02
NM_172049	Tmem18	-1.3	3E-02
ENSMUST00000112676	Zfp161	-1.3	2E-02
NR_039595	Mir5133	-1.3	2E-02
ENSMUST00000039487	Gtf2h5	-1.3	1E-02
NM_026790	Ifi27l1	-1.3	7E-03
NM_023598	Arid5b	-1.3	3E-02
NM_016707	Bcl11a	-1.3	4E-02
NM_011048	Pcsk6	-1.3	4E-02
ENSMUST00000154208	Dhrs3	-1.3	6E-03
NM_015821	Fbxl8	-1.3	4E-02
NM_007950	Ereg	-1.3	5E-02
NM_029169	Rbm6	-1.3	9E-03
ENSMUST00000151287	Tcp1	-1.3	6E-03
ENSMUST00000116234	Arl1	-1.3	4E-02
NM_001081429	Ccdc15	-1.3	6E-03
NR_003373	Gm15772	-1.3	3E-02
NM_053170	Trim33	-1.3	1E-02
NM_011764	Zfp90	-1.3	3E-02
NM_138741	Sdpr	-1.3	4E-02
NM_011122	Plod1	-1.3	3E-02
NM_001025296	Dffa	-1.3	2E-03
NM_026376	Plxnd1	-1.3	4E-02
NM_008747	Ntsr2	-1.3	4E-02
NR_040658	C330046G13Rik	-1.3	3E-03
NM_001177484	Gm11559	-1.3	5E-02

NM_133792	Pla2g15	-1.3	3E-02
NM_016768	Pbx3	-1.3	5E-02
NM_001013380	Dync1li2	-1.3	1E-02
ENSMUST00000004055	Dzip1	-1.3	4E-02
NM_001025375	Wdr61	-1.3	6E-03
XR_105713	Gm4544	-1.3	5E-02
NM_145944	Ccdc25	-1.3	4E-02
NM_153074	Lrrc25	-1.3	1E-02
NM_009281	Zfp143	-1.3	2E-02
NM_019804	B4galt4	-1.3	2E-02
ENSMUST00000006435	Atp6v1b2	-1.3	8E-04
NM_001142957	Zfp955b	-1.3	1E-02
XM_913098	Gm6729	-1.3	4E-02
NM_001081216	Phip	-1.3	3E-03
ENSMUST00000095458	2810008M24Rik	-1.3	3E-02
NM_207636	Fndc3a	-1.3	3E-02
NM_025460	Tmem126a	-1.3	3E-02
ENSMUST00000117648	Ttc3	-1.3	1E-03
NM_024286	Popdc3	-1.3	2E-02
ENSMUST00000037958	Arhgap29	-1.3	8E-03
ENSMUST00000069538	9330182L06Rik	-1.3	4E-02
NM_177806	Prpf39	-1.3	3E-02
NM_019786	Tbk1	-1.3	2E-03
NM_194054	Rtn4	-1.3	7E-03
NM_028724	Rin2	-1.3	2E-02
NM_001252374	Nt5c3	-1.3	1E-02
NM_134133	2010002N04Rik	-1.3	2E-02
NM_029847	Arsk	-1.3	8E-03
ENSMUST00000078691	Bak1	-1.3	2E-02
ENSMUST00000029358	Nmd3	-1.3	4E-02
NM_019953	Cnpy2	-1.3	3E-03
ENSMUST00000030556	Ptpn12	-1.3	5E-02
ENSMUST00000156431	Hacl1	-1.3	2E-02
NM_010811	Ndst2	-1.3	4E-02
NR_030529	Mir804	-1.3	5E-02
ENSMUST00000027322	Rhbdd1	-1.3	2E-02
NM_134110	Kcne2	-1.3	3E-02
NM_138742	Nap1l3	-1.3	2E-02
ENSMUST00000004172	Hmox2	-1.3	4E-02
NM_027455	Qpct	-1.3	4E-02
ENSMUST00000037205	Mcee	-1.3	6E-03
NM_009893	Chrd	-1.3	3E-02
NM_001162532	Fam174b	-1.3	5E-02
NM_008908	Ppic	-1.3	4E-02
NM_030080	Creb3l4	-1.3	9E-03
NM_021392	Ap4m1	-1.3	8E-03

NR_045063	4930556M19Rik	-1.3	4E-02
NM_028310	2810006K23Rik	-1.3	4E-02
NM_023223	Cdc20	-1.3	8E-03
ENSMUST00000160987	Srgn	-1.3	5E-02
NM_001172147	Rbm41	-1.3	9E-03
ENSMUST00000137035	St3gal6	-1.3	2E-02
NM_027994	Cand1	-1.3	2E-02
NM_001177646	Sirpa	-1.3	5E-03
NM_011462	Spin1	-1.3	5E-03
NM_025535	Sar1b	-1.3	2E-02
NM_001162366	Ptk2b	-1.3	3E-02
NM_025936	Rars	-1.3	1E-02
NM_013515	Stom	-1.3	6E-03
NM_010658	Mafb	-1.3	4E-02
NM_028049	Fbxo22	-1.3	4E-02
NM_001127177	Ptpn2	-1.3	3E-02
ENSMUST00000030091	Pole3	-1.3	4E-02
ENSMUST00000043722	Lgals3bp	-1.3	3E-02
NM_178916	Rfesd	-1.3	3E-02
ENSMUST00000021048	Ftsj3	-1.3	1E-02
NM_030742	Vmn1r63	-1.3	3E-02
NM_172517	Rbbp5	-1.3	6E-03
NM_029166	Uhrf1bp1l	-1.3	2E-02
NM_172717	Chfr	-1.3	3E-02
NM_001159396	Irf1	-1.3	4E-02
ENSMUST00000069800	Fut2	-1.3	3E-02
NM_133764	Atp6v0e2	-1.3	4E-02
NM_029150	Spata16	-1.3	4E-02
NR_046039	Gm8179	-1.3	3E-02
NM_177358	Zfp945	-1.3	1E-02
ENSMUST00000093106	Acsl6	-1.3	2E-02
NM_147050	Olfr659	-1.3	1E-02
NM_018819	Brp44l	-1.3	4E-03
NM_009223	Snn	-1.3	7E-03
NM_027276	Cdc16	-1.3	7E-03
ENSMUST00000090678	Rap1a	-1.3	1E-02
NM_021526	Psmd14	-1.3	5E-02
NM_025359	Tspan13	-1.3	5E-02
NR_045527	Rbbp8	-1.3	4E-02
NM_175211	Ralgps1	-1.3	3E-02
NM_145500	Ubtd1	-1.3	3E-02
NM_153164	Cnot1	-1.3	3E-04
NM_010299	Gm2a	-1.3	4E-03
NM_018765	Wbp4	-1.3	5E-02
NM_028031	Zdhhc13	-1.3	4E-02
NM_207214	Exoc5	-1.3	3E-02

ENSMUST00000033673	Nono	-1.3	4E-02
NM_011907	Trex2	-1.3	2E-02
NM_026213	Ttc33	-1.3	3E-02
ENSMUST00000069756	Ocln	-1.3	4E-02
ENSMUST00000086461	Rfc5	-1.3	2E-02
NM_199307	Ece1	-1.3	2E-02
NM_007975	F2rl3	-1.3	2E-02
ENSMUST00000026225	Sema4g	-1.3	4E-02
ENSMUST00000027264	Asnsd1	-1.3	2E-02
NM_001164614	Ccdc159	-1.3	5E-02
NM_134114	Sft2d1	-1.3	5E-03
NM_008477	Ktn1	-1.3	3E-02
NM_001161624	Cdkn1c	-1.3	4E-02
ENSMUST00000114843	Plac1	-1.3	3E-02
NM_011690	Vars	-1.3	2E-02
NM_001099297	Duox1	-1.3	4E-02
NR_045311	6030407O03Rik	-1.3	4E-02
NM_010765	Mapkapk5	-1.3	2E-02
ENSMUST00000003121	Rab8a	-1.3	4E-02
NM_175193	Golim4	-1.3	4E-02
NM_011857	Odz3	-1.3	3E-02
NM_013915	Zfp238	-1.3	2E-02
NR_028264	Dleu2	-1.3	4E-02
NM_181517	Ipo7	-1.3	3E-02
NM_134013	Psme4	-1.3	2E-03
NM_001205239	Parp6	-1.3	2E-03
NR_027804	Nagpa	-1.3	3E-02
NM_011868	Eci2	-1.3	2E-02
NM_001100591	Rc3h2	-1.3	6E-03
NM_134058	Pelo	-1.3	3E-02
NM_011811	Farsb	-1.3	1E-02
NM_197985	Adipor2	-1.3	3E-03
NM_027871	Arhgef3	-1.3	2E-02
NM_133674	Arhgef5	-1.3	5E-02
NM_001111021	Runx1	-1.3	2E-02
NM_001145960	Slc37a2	-1.3	4E-02
NM_010509	Ifnar2	-1.3	1E-02
ENSMUST00000086884	Armcx3	-1.3	3E-02
ENSMUST00000015100	Ppp1cb	-1.3	3E-03
NM_146240	Rassf9	-1.3	5E-02
NM_178883	Gorab	-1.3	1E-02
NM_145356	Zbtb7c	-1.3	6E-03
NM_029271	Mrpl32	-1.3	3E-02
NM_008379	Kpnb1	-1.3	7E-03
BC158088	Gm505	-1.3	3E-02
NM_178626	Cdc42se2	-1.3	1E-02

ENSMUST00000103147	Psmb3	-1.3	3E-02
ENSMUST00000106645	Micalcl	-1.3	6E-03
NM_025706	Tbc1d15	-1.3	1E-03
BC002059	BC002059	-1.3	5E-02
ENSMUST00000107644	Gtf2h1	-1.3	2E-03
NM_001252193	Amz2	-1.3	5E-02
NM_011544	Tcf12	-1.3	1E-02
NM_025509	Ostc	-1.3	9E-03
ENSMUST00000027846	Tada1	-1.3	4E-02
NM_026563	Sdccag3	-1.3	2E-02
NM_009671	Ankfy1	-1.2	9E-03
NM_133826	Atp6v1h	-1.2	3E-02
ENSMUST00000088552	Myl9	-1.2	4E-02
NM_011959	Orc5	-1.2	2E-02
NM_026893	Dcaf12	-1.2	5E-03
NM_011816	G3bp2	-1.2	2E-03
NM_011655	Tubb5	-1.2	5E-02
NM_026496	Grhl2	-1.2	3E-02
NM_177778	Armc7	-1.2	5E-02
NM_008185	Gstt1	-1.2	3E-02
NM_027807	Cul5	-1.2	3E-02
NM_133354	Sumo2	-1.2	2E-03
NM_027164	Lrrc27	-1.2	3E-02
NM_053069	Atg5	-1.2	2E-02
NM_021541	Cryba2	-1.2	7E-03
NM_001001999	Gp1bb	-1.2	6E-03
ENSMUST00000114551	Cetn2	-1.2	2E-02
NM_022889	Pes1	-1.2	4E-03
NM_145469	Nipal2	-1.2	5E-02
NM_177682	Ccz1	-1.2	2E-02
NM_010421	Hexa	-1.2	2E-03
NM_001033347	D430041D05Rik	-1.2	5E-02
NM_025947	Dynlrb1	-1.2	2E-02
NM_007399	Adam10	-1.2	1E-02
NM_001143671	Fam70b	-1.2	4E-02
NM_001037737	Arnt	-1.2	3E-02
NM_001039147	Morf4l1	-1.2	3E-03
NM_025574	Pigy	-1.2	4E-02
NM_201638	Mettl14	-1.2	5E-02
ENSMUST00000028928	Gzf1	-1.2	5E-02
NM_026361	Pkp4	-1.2	1E-02
NM_019699	Fads2	-1.2	2E-02
NM_025840	Bzw2	-1.2	4E-02
NM_134255	Elovl5	-1.2	4E-02
NM_028354	Tdp1	-1.2	2E-02
NR_038030	4930562F07Rik	-1.2	4E-02

NM_148925	Fyco1	-1.2	9E-04
ENSMUST00000130916	Becn1	-1.2	5E-03
ENSMUST00000004910	Eif2b2	-1.2	4E-02
NM_146714	Olfr248	-1.2	2E-02
NM_001198789	Gm5918	-1.2	3E-02
ENSMUST00000081932	Nmt2	-1.2	3E-02
NM_001164099	Add3	-1.2	5E-02
ENSMUST00000030626	Tmem50a	-1.2	2E-02
ENSMUST00000155521	Itgav	-1.2	5E-02
ENSMUST00000047627	Gpbp1	-1.2	3E-02
NM_012026	Rgnef	-1.2	3E-02
ENSMUST00000007993	Rbm28	-1.2	3E-03
NM_001170433	Ppfibp1	-1.2	1E-02
NM_001136104	Abl2	-1.2	4E-02
NM_001163755	Skor1	-1.2	5E-02
NM_015737	Galnt4	-1.2	4E-02
NM_027288	Manba	-1.2	1E-03
XM_003085917	Gm3552	-1.2	1E-02
NM_172255	Wdr11	-1.2	6E-03
NM_011375	St3gal5	-1.2	1E-02
NM_001100415	Gm14430	-1.2	2E-03
AK143696	Gm10374	-1.2	3E-02
NM_007481	Arf6	-1.2	5E-03
NR_033462	Gm10494	-1.2	2E-02
NM_001100110	Srp54c	-1.2	2E-02
NM_001177406	Gm14431	-1.2	4E-02
AK163559	Cyp11a1	-1.2	2E-02
NM_053116	Wnt16	-1.2	4E-02
NM_016706	Coil	-1.2	4E-02
NR_045362	Gm6602	-1.2	3E-02
NM_013661	Sema5b	-1.2	5E-02
NM_019674	Ppp4c	-1.2	3E-02
NM_018776	Crlf3	-1.2	3E-02
NM_025272	Atp6v0e	-1.2	3E-02
NM_016739	Caprin1	-1.2	6E-03
NM_153800	Arhgap22	-1.2	4E-02
NM_010370	Gzma	-1.2	4E-02
NM_030750	Sgpp1	-1.2	1E-03
NM_001109743	Skor2	-1.2	2E-02
NM_016961	Mapk9	-1.2	4E-02
NM_199447	Rrp12	-1.2	4E-02
NM_001024508	Brd9	-1.2	3E-02
NM_026696	0610030E20Rik	-1.2	3E-02
NM_198650	Slc22a20	-1.2	4E-02
NM_021790	Cenpk	-1.2	5E-02
NM_175149	2310022B05Rik	-1.2	5E-02

NM_008915	Ppp3cc	-1.2	1E-02
NM_001172136	Exog	-1.2	2E-02
ENSMUST00000003117	Ap1m1	-1.2	3E-02
NM_172742	Mtmr10	-1.2	2E-02
NM_008302	Hsp90ab1	-1.2	3E-02
ENSMUST00000036647	Ctdspl2	-1.2	2E-02
ENSMUST00000117363	Lsg1	-1.2	4E-02
ENSMUST00000002848	Grin2d	-1.2	8E-03
NM_029478	Vmp1	-1.2	5E-02
ENSMUST00000155676	Ubl4	-1.2	3E-02
NM_001111059	Cd34	-1.2	4E-02
ENSMUST00000126879	Zfp369	-1.2	3E-02
NM_008443	Kif3a	-1.2	3E-02
NM_021437	1700123O20Rik	-1.2	3E-03
ENSMUST00000081104	Timm17a	-1.2	4E-02
NM_001164370	Mipol1	-1.2	5E-02
NM_178928	Afap1l1	-1.2	1E-02
NM_011396	Slc22a5	-1.2	4E-02
NM_019796	Syncrip	-1.2	3E-02
AK016044	Hydin	-1.2	3E-02
ENSMUST00000169156	Abhd12b	-1.2	4E-02
ENSMUST00000080713	Utp14a	-1.2	4E-02
NM_026002	Mtdh	-1.2	1E-02
NM_001005868	Erbb2ip	-1.2	2E-02
NM_009968	Cryz	-1.2	3E-02
NM_011626	Tmem165	-1.2	1E-02
NR_033593	4930529C04Rik	-1.2	5E-02
NM_011876	Twf2	-1.2	5E-02
BC132251	Vmn1r212	-1.2	1E-02
NM_026559	Txndc17	-1.2	2E-02
NM_025736	Ttc35	-1.2	1E-02
NM_183016	Cdc42bpb	-1.2	5E-02
NM_080634	Hps3	-1.2	3E-02
NM_133973	Cog4	-1.2	1E-02
NM_134029	Nt5m	-1.2	4E-02
ENSMUST00000022380	Psmc6	-1.2	4E-02
NM_001017959	Lamp2	-1.2	1E-02
NM_138592	Usp39	-1.2	6E-03
ENSMUST00000040721	Tpst1	-1.2	4E-02
ENSMUST00000118522	Gcfc1	-1.2	5E-02
NM_029606	Ccdc46	-1.2	4E-02
ENSMUST00000029353	Kpna4	-1.2	4E-03
ENSMUST00000111338	Ckap5	-1.2	1E-02
NM_011899	Srp54a	-1.2	2E-02
NM_001190870	Kcne3	-1.2	4E-02
NM_175294	Nucks1	-1.2	1E-02

NM_019393	Exosc9	-1.2	5E-02
NM_007387	Acp2	-1.2	9E-03
NM_010880	Ncl	-1.2	4E-02
ENSMUST00000114124	Tiam1	-1.2	2E-02
ENSMUST00000006353	Cdkal1	-1.2	4E-02
NM_007761	Crcp	-1.2	2E-03
ENSMUST00000045312	Smc1a	-1.2	3E-02
NM_027439	Atp6ap2	-1.2	3E-02
NM_001130408	Arf1	-1.2	9E-03
NM_026626	Efcab2	-1.2	4E-02
NM_133235	Khdrbs2	-1.2	3E-02
NM_175101	Tmem111	-1.2	3E-03
NM_001100109	Srp54b	-1.2	6E-03
NM_020611	Srd5a3	-1.2	4E-02
NM_025349	Lsm7	-1.2	1E-02
NM_019717	Atl2	-1.2	2E-02
NM_026272	Narf	-1.2	5E-02
NM_011889	Septin3	-1.5	6E-04
ENSMUST00000027495	Septin2	-1.2	5E-02

Supplementary Table 6: Protein-coding genes *exclusively* differentially-expressed (either induced (black type-face) or repressed (blue type-face)) in S/E[18] peptide-treated animals relative to saline-treated controls. Only genes exhibiting a fold-change (FC) $\geq$ 1.2 and p value (P) $\leq$0.05 are considered. In cases of multiple probes mapping to the same transcript, the greatest FC is reported. Genes are ranked according to fold-change.

		SE v Control	
**RefSeq/Ensembl ID**	**Gene Symbol**	**FC**	**P**
NM_010240	Ftl1	3.5	9E-03
NM_011354	Serf2	3.3	2E-02
NM_008220	Hbb-b1	3.3	4E-02
NM_009976	Cst3	3.0	1E-02
NR_045304	1700030N03Rik	2.9	9E-04
XM_982603	Gm8005	2.7	5E-03
XM_144599	Gm4963	2.6	1E-02
NM_001005488	Olfr467	2.6	2E-02
NM_001024731	LOC100044193	2.6	3E-03
XR_140674	LOC100862132	2.5	1E-02
XR_141676	LOC100861856	2.5	3E-02
NM_019910	Dcpp1	2.4	2E-02
L37873	Trbv13-2	2.4	4E-03
NM_027955	Gmcl1l	2.4	4E-02
AK076905	1700040F15Rik	2.4	3E-02
NM_009095	Rps5	2.4	2E-02
NM_007894	Ear1	2.4	4E-02
NM_010640	Klk1b11	2.4	2E-03
ENSMUST00000098140	Olfr1532-ps1	2.4	2E-02
XR_106535	Gm19876	2.3	2E-02
ENSMUST00000075382	Gm8213	2.3	1E-02
XR_104955	Gm19617	2.3	2E-03
XM_003689275	LOC100862598	2.3	1E-02
ENSMUST00000075737	Gm5329	2.3	2E-03
NR_040666	2900093L17Rik	2.2	4E-04
ENSMUST00000152407	Gm8842	2.2	1E-03
ENSMUST00000154148	Arfip1	2.2	8E-03
NM_009429	Tpt1	2.2	1E-02
NM_146538	Olfr315	2.2	9E-03
XM_003085279	Srsx	2.2	2E-02
NM_011188	Psmc2	2.2	9E-03
XM_904838	LOC666692	2.1	5E-02
BC147603	Gm732	2.1	1E-02
XM_001474423	Gm2745	2.1	6E-04
NM_001038997	Gm5771	2.1	2E-02
XR_105301	Gm19592	2.1	2E-02
NM_001109970	Gm10486	2.1	6E-03
NM_011296	Rps18	2.0	2E-02
NM_144825	Taok1	2.0	3E-02

NM_001177545	Zfp600	2.0	1E-02
NM_001166743	Vmn1r117	2.0	5E-03
NR_030707	Vmn1r-ps79	2.0	4E-02
NM_001109969	Gm10058	2.0	3E-02
ENSMUST00000098844	Vmn1r55	2.0	2E-02
XR_108082	Gm19447	2.0	3E-02
NM_007606	Car3	2.0	6E-04
ENSMUST00000095561	Gm4666	2.0	2E-02
AK013560	A230006K03Rik	1.9	3E-03
XM_887355	Gm6238	1.9	8E-03
ENSMUST00000084760	Olfr472	1.9	1E-02
NM_029803	Ifi27l2a	1.9	1E-02
NM_001081669	Rhox2d	1.9	3E-02
NM_178598	Tagln2	1.9	4E-02
NM_009139	Ccl6	1.9	3E-02
XM_003085369	Gm6625	1.9	7E-03
NM_010669	Krt6b	1.9	2E-03
NM_175656	Hist1h4i	1.9	3E-02
NM_134194	Vmn1r225	1.9	2E-02
XR_140478	Gm2710	1.9	4E-02
NM_011964	Psg19	1.9	2E-02
NM_177670	Tmem69	1.9	4E-02
NM_009394	Tnnc2	1.9	1E-02
ENSMUST00000173019	Rps28	1.9	7E-04
NR_024599	Gm11346	1.9	1E-02
ENSMUST00000096366	1700010D01Rik	1.9	4E-02
NM_007542	Bgn	1.8	3E-03
ENSMUST00000177523	Prh1	1.8	3E-02
XR_140534	LOC100861957	1.8	1E-02
NM_053230	Vmn1r41	1.8	2E-02
ENSMUST00000068475	Olfr354	1.8	2E-03
NM_029614	Prss23	1.8	1E-02
NM_175167	Bpifb9a	1.8	2E-02
XR_141165	LOC100861733	1.8	4E-02
NM_011835	Katna1	1.8	9E-03
NM_001254951	Gm4636	1.8	2E-02
NM_001081005	1500012F01Rik	1.8	5E-03
NM_015809	Krtap5-4	1.8	8E-04
AK014756	4833423F13Rik	1.8	2E-02
XM_003085914	LOC100505112	1.8	5E-02
NM_177397	Atp6v1g3	1.8	4E-03
NM_001011784	Olfr1039	1.8	9E-03
NM_001177419	Fbxw28	1.8	3E-02
NM_026011	Arl8b	1.8	1E-02
NR_027478	Gm8580	1.8	1E-03
NM_008681	Ndrg1	1.8	3E-03

NM_009443	Tgoln1	1.8	4E-03
NM_026439	Ccdc80	1.8	2E-03
XR_142437	LOC100862607	1.8	7E-03
NM_130905	Cd209e	1.8	5E-03
NM_015804	Atp11a	1.8	2E-02
NM_177744	Apol10a	1.8	3E-02
XR_034708	1700063K16Rik	1.8	3E-02
XR_140744	4932422M17Rik	1.8	8E-03
ENSMUST00000076262	Olfr193	1.8	4E-02
ENSMUST00000164181	Myl6	1.8	2E-02
NM_019468	G6pd2	1.8	2E-02
BC089468	1700003E24Rik	1.8	1E-03
ENSMUST00000021471	Tmx1	1.7	7E-03
NR_003596	Gm6455	1.7	3E-02
NM_013620	Olfr68	1.7	3E-02
NM_019547	Rbm38	1.7	1E-02
NM_152944	Mmp21	1.7	2E-02
BC087948	BC024386	1.7	1E-02
NM_026168	Ergic2	1.7	4E-02
NM_001033280	Gm94	1.7	7E-03
ENSMUST00000078401	Gm6280	1.7	1E-02
NM_201375	Kng2	1.7	3E-02
XM_982175	Gm7969	1.7	1E-02
ENSMUST00000097423	Rsph3a	1.7	3E-03
BC084675	Rps3a	1.7	4E-03
NM_001164567	Vill	1.7	9E-03
NM_199314	Serpina11	1.7	8E-03
XM_003689165	LOC100505355	1.7	2E-03
NM_007566	Birc6	1.7	2E-02
NM_010570	Irs1	1.7	3E-02
NM_133670	Sult1a1	1.7	2E-02
NM_001177416	Gm6792	1.7	1E-02
ENSMUST00000176838	Vmn1r4	1.7	4E-02
NM_027983	Krt33a	1.7	9E-03
NM_010285	Ghrh	1.7	3E-02
NM_008802	Pde7a	1.7	3E-02
NM_026418	Rgs10	1.7	7E-03
DQ078272	Igk-V28	1.7	1E-02
NM_020268	Klk1b27	1.7	5E-02
NM_001256066	Gm5326	1.7	1E-03
NM_001166725	Vmn1r129	1.7	4E-02
XR_141910	LOC100862110	1.7	2E-02
NR_045264	AI847159	1.7	5E-02
NM_019817	Copz1	1.7	4E-02
NM_010809	Mmp3	1.6	5E-03
NM_029767	Rps9	1.6	8E-03

ENSMUST00000084017	Gm5908	1.6	4E-03
BC083100	Gm5946	1.6	3E-03
XM_003689209	LOC100862451	1.6	2E-02
NM_001011793	Olfr598	1.6	5E-02
NR_044985	2210039B01Rik	1.6	5E-04
NR_040757	0610040F04Rik	1.6	5E-02
NM_001011707	Cyp2c66	1.6	5E-02
NM_001105063	Vmn2r32	1.6	3E-02
NR_045289	2610019E17Rik	1.6	6E-03
NM_009112	S100a10	1.6	4E-03
ENSMUST00000127592	Cd209c	1.6	1E-02
AF510860	Flg	1.6	5E-02
NM_133643	Edaradd	1.6	1E-02
NM_153115	Spag11a	1.6	2E-02
XM_484529	Gm5481	1.6	1E-02
Z31359	Npn2	1.6	2E-02
NM_027303	Ankrd60	1.6	2E-02
AK020289	9130230N09Rik	1.6	2E-02
NM_029336	1700022P22Rik	1.6	2E-02
ENSMUST00000102616	Tekt2	1.6	3E-02
XM_003086733	Gm10144	1.6	1E-02
ENSMUST00000071943	Olfr222	1.6	3E-02
XR_142097	LOC100861795	1.6	2E-02
ENSMUST00000099767	Olfr1251	1.6	2E-02
NM_146126	Sord	1.6	3E-02
XR_142229	Gm17768	1.6	4E-02
NM_173070	Sprr4	1.6	2E-03
NM_181543	Gpr151	1.6	7E-03
NR_046021	Gm6249	1.6	4E-02
NM_026503	1110058L19Rik	1.6	2E-02
NM_009712	Arsb	1.6	4E-02
ENSMUST00000162752	Sh2d1b2	1.6	3E-02
AK084071	Gm10021	1.6	3E-02
NM_009777	C1qb	1.6	4E-02
NM_001243021	Zfp939	1.6	3E-02
NM_001039042	Klk13	1.6	4E-02
NR_024720	2700099C18Rik	1.6	3E-02
NM_177913	A430089I19Rik	1.6	3E-02
NM_001083955	Hba-a2	1.6	4E-03
NM_001166841	Vmn1r158	1.6	3E-02
NM_146826	Olfr969	1.6	1E-02
NM_011822	Pigq	1.6	2E-02
NM_008456	Klk1b5	1.6	4E-02
NR_040609	2010009K17Rik	1.6	4E-02
NM_201645	Ugt1a1	1.6	9E-03
NM_001166758	Vmn1r159	1.6	3E-02

NM_011460	Serpinb9d	1.6	2E-02
NM_010177	Fasl	1.6	1E-02
ENSMUST00000134652	Ttc39d	1.6	2E-02
NM_138665	Sardh	1.6	4E-03
AK090265	G630030J09Rik	1.6	3E-02
NM_177736	Lrrc61	1.6	2E-02
NM_001083916	1810019J16Rik	1.6	2E-02
NM_027221	Krtcap3	1.6	2E-02
NM_025931	Ift27	1.6	2E-02
NM_001042711	Amy2a5	1.6	4E-02
NM_201236	Rhox4e	1.6	4E-02
BC022764	2610528J11Rik	1.6	2E-02
ENSMUST00000001456	Tmem79	1.6	2E-02
NM_026382	Snrnp48	1.6	2E-03
NR_033484	Gm10046	1.6	1E-02
NM_011313	S100a6	1.6	1E-02
ENSMUST00000019920	Clvs2	1.6	2E-03
NM_134141	Ciapin1	1.5	4E-02
NR_045479	1700024B18Rik	1.5	4E-03
NM_010814	Mog	1.5	2E-02
XM_003085800	Gm3908	1.5	6E-03
AK018881	1700066C05Rik	1.5	4E-02
NR_028066	Gm4926	1.5	2E-03
NM_001190381	Olfr596	1.5	4E-02
NR_045416	4930513D17Rik	1.5	2E-02
XM_912278	Gm5292	1.5	1E-02
NM_153784	Ccdc64b	1.5	5E-03
ENSMUST00000052902	Timm8a1	1.5	3E-02
ENSMUST00000035918	Cyp3a11	1.5	3E-02
XM_983418	Gm8066	1.5	1E-02
NM_146813	Olfr651	1.5	2E-02
NM_207575	Olfr1480	1.5	3E-02
NR_040616	A130077B15Rik	1.5	2E-02
AK016119	4930554G24Rik	1.5	2E-02
NM_001102665	Gm14920	1.5	9E-04
NM_026069	Rpl37	1.5	2E-02
XR_105496	Gm20246	1.5	2E-02
NM_001001332	BC117090	1.5	2E-02
NM_133675	1110032A04Rik	1.5	2E-02
XR_104603	Gm19918	1.5	4E-02
NM_001001985	Nat8l	1.5	2E-02
NR_040542	1700036G14Rik	1.5	1E-02
NM_146495	Olfr474	1.5	2E-02
NR_001586	Speer1-ps1	1.5	5E-02
NM_178689	Eno4	1.5	1E-02
XR_033201	4930401B11Rik	1.5	1E-02

NM_001177796	Afap1l2	1.5	2E-02
BC106999	LOC100043315	1.5	2E-02
NR_033583	Spn-ps	1.5	3E-02
ENSMUST00000163078	Ms4a6b	1.5	3E-02
NM_001011835	Olfr1162	1.5	2E-02
NM_001005230	Olfr1024	1.5	2E-02
NM_146681	Olfr1424	1.5	1E-02
ENSMUST00000117377	Spock3	1.5	1E-02
NM_013677	Surf1	1.5	7E-03
XR_105091	Gm19512	1.5	4E-02
NR_045961	4930438E09Rik	1.5	2E-02
NM_011662	Tyrobp	1.5	9E-03
NM_009619	Adam3	1.5	4E-02
NM_027304	H1fnt	1.5	4E-02
NR_015546	C130026L21Rik	1.5	8E-03
ENSMUST00000114222	Gng12	1.5	6E-04
NM_008290	Hsd17b2	1.5	2E-02
NR_033215	3000002C10Rik	1.5	2E-02
NR_045705	I730028E13Rik	1.5	1E-02
NM_146677	Olfr825	1.5	8E-03
NM_010047	Dgcr6	1.5	4E-03
NM_025511	Fam36a	1.5	3E-02
NR_045404	G630055G22Rik	1.5	3E-02
NM_001003664	Gm5409	1.5	4E-02
NM_001164683	Tmem29	1.5	4E-02
XR_107094	Gm19783	1.5	3E-02
NM_198655	4921509C19Rik	1.5	4E-02
NM_028671	Fam122c	1.5	3E-02
ENSMUST00000105971	Tnni2	1.5	3E-02
NM_172880	Tmprss11e	1.5	2E-02
BC150906	AU015228	1.5	1E-02
NM_146345	Olfr1491	1.5	2E-02
NR_040405	Gm6260	1.5	3E-02
NM_001163533	Rtdr1	1.5	4E-03
XR_107942	Gm20299	1.5	4E-02
ENSMUST00000005019	Crabp2	1.5	2E-02
NM_145822	Cd3eap	1.5	1E-02
XM_621774	Gm6003	1.5	3E-02
XR_107106	Gm19389	1.5	1E-03
NM_009426	Trh	1.5	4E-02
NM_023816	Ankrd36	1.5	3E-02
NM_007994	Fbp2	1.5	4E-02
NM_172756	Ankle1	1.5	1E-02
XR_142005	LOC100862601	1.5	5E-02
NM_008062	G6pdx	1.5	3E-02
NM_001098789	Ndufa4l2	1.5	5E-02

ENSMUST00000168151	Gm13286	1.5	1E-02
ENSMUST00000152782	Heg1	1.5	4E-02
NM_009858	Cd8b1	1.5	1E-02
NM_001013817	Sp140	1.5	2E-02
NM_001170537	Mef2c	1.5	4E-02
ENSMUST00000038212	Gzmk	1.5	3E-02
NM_011468	Sprr2a1	1.5	1E-02
NR_038149	1700016P04Rik	1.5	1E-02
NM_028514	Actrt1	1.5	2E-02
NM_153079	Nmur2	1.5	3E-02
BC067002	4632419I22Rik	1.5	2E-02
NM_001199948	Dynlt1f	1.5	5E-03
XR_141635	LOC674295	1.5	4E-03
ENSMUST00000100645	Eddm3b	1.5	4E-02
NM_177347	Ifna13	1.5	2E-02
ENSMUST00000071536	Gm6570	1.5	5E-03
ENSMUST00000160218	Gm16532	1.5	2E-02
NR_045265	2010003O02Rik	1.5	3E-02
ENSMUST00000029991	Ppp3r2	1.5	5E-02
NM_001204371	Oprk1	1.5	4E-03
NM_001252679	Smr2	1.5	1E-03
NM_173069	Speer2	1.5	2E-02
NM_177651	4933409G03Rik	1.5	4E-02
AK020970	B230112J18Rik	1.5	3E-02
NM_007946	Epx	1.5	5E-02
NM_178028	Galp	1.5	1E-02
NM_011858	Odz4	1.5	4E-02
ENSMUST00000032344	Arhgdib	1.5	9E-03
NM_009321	Tbca	1.5	3E-02
ENSMUST00000129439	H13	1.5	1E-02
NR_045052	Gm20187	1.4	3E-02
XM_485074	Gm14135	1.4	1E-02
NM_183268	Defa20	1.4	1E-02
NM_016740	S100a11	1.4	2E-02
NM_022886	Scel	1.4	2E-02
NM_020569	Park7	1.4	2E-02
NR_045968	4930455B14Rik	1.4	1E-03
NR_045358	4930556N09Rik	1.4	5E-02
AK165489	F530104D19Rik	1.4	8E-03
NM_025750	4933417A18Rik	1.4	3E-02
NM_026811	Lce1e	1.4	2E-02
BC115964	Slc36a4	1.4	4E-02
ENSMUST00000081457	Lgals7	1.4	4E-02
ENSMUST00000132883	Gm14295	1.4	5E-02
ENSMUST00000154473	AI413582	1.4	1E-02
ENSMUST00000084253	Epb4.1	1.4	3E-02

NR_045350	4930405J17Rik	1.4	3E-03
NM_026678	Blvra	1.4	1E-02
NM_011254	Rbp1	1.4	3E-02
NR_045161	A830009L08Rik	1.4	1E-02
XM_003084729	Gm7226	1.4	2E-02
NM_026385	Pllp	1.4	2E-02
NM_026899	Ssu72	1.4	2E-02
ENSMUST00000006254	Tbcb	1.4	4E-02
AK013159	2810425M01Rik	1.4	1E-02
XR_141019	Gm9049	1.4	3E-02
NR_028586	Gm11978	1.4	2E-02
ENSMUST00000020963	Dcdc2c	1.4	2E-02
NM_183250	Ccdc72	1.4	2E-02
NM_175165	Tprg	1.4	5E-03
NM_008004	Fgf17	1.4	3E-02
NM_013739	Dok3	1.4	2E-04
NM_011023	Otx1	1.4	3E-02
ENSMUST00000100339	Commd6	1.4	1E-02
NM_010600	Kcnh1	1.4	5E-03
XR_141410	Gm8692	1.4	1E-02
ENSMUST00000026565	Ifitm3	1.4	5E-03
NM_009904	Clgn	1.4	7E-03
NM_007572	C1qa	1.4	3E-02
NM_026730	Gpihbp1	1.4	5E-02
AK139372	Gm8098	1.4	2E-02
NM_027907	Agxt2l1	1.4	3E-02
NM_009987	Cx3cr1	1.4	4E-02
NM_009434	Phlda2	1.4	2E-02
NM_009042	Reg1	1.4	5E-03
NM_007608	Car5a	1.4	4E-02
AK138039	Gm10757	1.4	4E-04
ENSMUST00000119968	Mrpl24	1.4	2E-02
ENSMUST00000072425	Tcf7	1.4	5E-02
XR_108054	LOC100503303	1.4	9E-03
NM_001081982	Nfix	1.4	5E-02
NM_177473	Tmem191c	1.4	4E-02
NM_153508	Clstn3	1.4	2E-02
NM_028598	2410076I21Rik	1.4	3E-02
NM_177905	Piwil4	1.4	2E-02
XR_142180	Gm8564	1.4	8E-03
NM_001252341	Syt1	1.4	4E-02
AK005578	4933432K03Rik	1.4	9E-03
NR_040445	1700017G19Rik	1.4	1E-02
XM_003086377	Spocd1	1.4	2E-02
AK019644	4930473D10Rik	1.4	8E-03
NR_028030	Gm9767	1.4	4E-03

NM_001145759	4930571K23Rik	1.4	3E-02
XM_355309	Gm13194	1.4	1E-02
NM_001004061	Fam170a	1.4	2E-02
ENSMUST00000103152	Cdk5rap3	1.4	3E-02
NM_029106	Gm14354	1.4	6E-03
NM_207233	C1ql2	1.4	1E-02
NM_009690	Cd5l	1.4	2E-02
ENSMUST00000067020	Nkx2-4	1.4	2E-02
ENSMUST00000031982	Hpgds	1.4	2E-02
ENSMUST00000110612	2310003F16Rik	1.4	3E-02
NM_001081424	Chrna10	1.4	5E-02
NM_024220	Ndufc2	1.4	1E-02
NM_001042451	Snca	1.4	8E-03
NR_040483	9530026F06Rik	1.4	1E-02
NM_001099312	Gm11569	1.4	2E-02
NR_046272	7420700N18Rik	1.4	4E-02
ENSMUST00000170719	Sftpa1	1.4	3E-02
NM_009526	Wnt6	1.4	4E-02
NM_181545	Slfn8	1.4	1E-02
NM_001112739	Kcnc1	1.4	4E-02
NM_011185	Psmb1	1.4	3E-02
NM_145535	Sdcbp2	1.4	1E-02
XM_909733	Gm4883	1.4	3E-02
NM_001163730	Gm9125	1.4	4E-02
NM_026754	Ucma	1.4	4E-02
NM_011637	Trex1	1.4	4E-02
NM_001039678	Prhoxnb	1.4	3E-02
ENSMUST00000038570	Nipsnap1	1.4	1E-02
ENSMUST00000164598	Acox2	1.4	7E-03
AK143085	G630018N14Rik	1.4	8E-03
NM_030055	Tmem210	1.4	2E-02
NM_019484	Alyref2	1.4	3E-02
ENSMUST00000073570	Zfp414	1.4	5E-02
NM_001163223	Zfp804b	1.4	1E-02
NM_025851	1700010I14Rik	1.4	3E-02
NM_011028	P2rx6	1.4	7E-03
NM_009133	Stmn3	1.4	5E-02
NM_177469	Gpr123	1.4	1E-03
NM_029802	Arfip2	1.4	3E-03
NM_001206369	Gsn	1.4	2E-02
NM_183318	Rgag4	1.4	3E-02
NM_026758	Mphosph6	1.4	1E-02
NM_177919	Tceal5	1.4	4E-02
BC036300	1700054N08Rik	1.4	4E-02
NR_037984	Gm19461	1.4	2E-02
NR_040277	B230378P21Rik	1.4	2E-02

NM_007926	Aimp1	1.4	5E-03
NM_031393	Sytl1	1.4	1E-02
NR_045188	St18	1.4	3E-02
NM_139226	Onecut3	1.4	5E-02
NM_001042768	Proc	1.4	2E-02
NM_001171010	Slc14a1	1.4	1E-02
ENSMUST00000073080	Cycs	1.4	3E-02
NR_033786	1700052I22Rik	1.4	6E-03
AK147301	Gm10723	1.4	9E-03
NM_206871	Ifna6	1.4	2E-02
NM_001177538	Gm10394	1.4	5E-02
NR_002854	Dlx1as	1.4	2E-02
NR_040516	4930528P14Rik	1.4	4E-02
NM_008972	Ptma	1.4	5E-02
BC051076	BC051076	1.4	2E-02
XR_107370	Gm13431	1.4	1E-02
XR_104931	Gm19862	1.4	5E-02
NM_009872	Cdk5r2	1.4	2E-02
ENSMUST00000055890	Olfr523	1.4	6E-03
BC150693	4930579F01Rik	1.4	4E-02
XM_905288	Gm5218	1.4	5E-02
NR_003652	Gm7337	1.4	4E-02
NR_045938	1700128A07Rik	1.4	4E-03
ENSMUST00000051540	Olfr460	1.4	3E-02
XM_910525	Gm5528	1.4	4E-02
NM_177155	Klri2	1.4	3E-02
XM_003688747	Gm6590	1.4	5E-02
NM_031254	Trem2	1.4	1E-02
AK043525	A830005F24Rik	1.4	1E-02
XM_001479065	Gm8526	1.4	7E-03
XR_142285	LOC621118	1.4	1E-02
AK015742	4930510E17Rik	1.4	5E-02
NM_025821	Carhsp1	1.4	3E-02
NM_021889	Syt9	1.4	4E-02
NR_040614	4930533B01Rik	1.4	3E-03
NM_009643	Ahnak	1.4	4E-02
NM_021882	Pmel	1.4	5E-02
NM_001033233	Tmprss11a	1.4	4E-02
NR_015569	4732471J01Rik	1.4	5E-03
NM_207247	Pglyrp3	1.4	5E-02
NM_010229	Flt3	1.4	4E-02
NM_001013777	Zfp488	1.4	2E-02
NM_001033298	Plk1s1	1.4	2E-02
BC046251	BC046251	1.4	1E-03
NR_040625	9430032N09Rik	1.4	4E-02
NM_134248	Havcr1	1.4	2E-02

ENSMUST00000117687	Ppp2r2b	1.4	3E-02
NR_040482	C030007H22Rik	1.4	1E-02
AK143758	AI314278	1.4	2E-02
NM_029254	Spdya	1.4	2E-02
ENSMUST00000032841	Mrpl46	1.4	5E-02
ENSMUST00000022256	Psmd6	1.4	2E-02
ENSMUST00000056517	Gja10	1.4	4E-02
XR_142300	Gm20333	1.4	3E-02
XM_910785	Gm12770	1.3	5E-02
NM_011077	Phex	1.3	9E-03
ENSMUST00000030614	CK137956	1.3	3E-02
NM_146889	Olfr1302	1.3	3E-02
ENSMUST00000028553	Nop10	1.3	3E-03
NM_009226	Snrpd1	1.3	6E-03
NM_175524	C130060K24Rik	1.3	2E-02
NM_001163247	Birc7	1.3	4E-02
NM_053091	Cox4i2	1.3	5E-03
NM_008939	Prss12	1.3	3E-02
NM_182959	Slc17a8	1.3	2E-02
NM_020051	Ascl3	1.3	1E-02
AK016217	4930564C03Rik	1.3	4E-02
NM_031842	Smarcd1	1.3	9E-03
AK006227	1700021P04Rik	1.3	2E-02
ENSMUST00000044953	Svs2	1.3	3E-02
NR_045715	F420014N23Rik	1.3	4E-02
NM_013698	Txk	1.3	3E-02
NM_001164201	Lass3	1.3	3E-03
ENSMUST00000166768	Spata2l	1.3	2E-02
ENSMUST00000136312	Ubc	1.3	3E-02
NM_001011755	Olfr671	1.3	3E-02
NM_146741	Olfr1497	1.3	5E-02
ENSMUST00000029386	Etfdh	1.3	5E-03
NM_001163243	Fis1	1.3	9E-03
NM_001195413	Cngb1	1.3	1E-02
NM_024284	Hagh	1.3	4E-02
XR_107882	Gm15567	1.3	4E-02
NM_001007571	D8Ertd738e	1.3	2E-02
NM_009459	Ube2h	1.3	3E-02
XR_107564	Gm20271	1.3	5E-02
BC147441	Gm949	1.3	1E-02
NM_010602	Kcnj11	1.3	1E-02
NM_008388	Eif3e	1.3	3E-02
NM_022016	Impg1	1.3	4E-02
NR_015539	9630013A20Rik	1.3	2E-02
NM_001001885	Tmem151a	1.3	4E-02
NM_026731	Ppp1r14a	1.3	5E-02

NM_001081408	Agmat	1.3	4E-02
XM_488314	Gm5751	1.3	5E-02
ENSMUST00000017255	Krt24	1.3	2E-02
NR_045776	9530080O11Rik	1.3	3E-02
ENSMUST00000001122	Slc13a2	1.3	1E-02
NM_175116	Lpar6	1.3	2E-02
BC147620	Gm904	1.3	3E-02
NM_007756	Cplx1	1.3	3E-02
XR_105136	Gm19656	1.3	3E-02
NM_019647	Rpl21	1.3	7E-03
NM_029632	Ppp1r11	1.3	9E-03
NM_015786	Hist1h1c	1.3	2E-02
BC051070	BC051070	1.3	4E-02
NM_010807	Marcksl1	1.3	9E-03
NM_145922	Kcnc4	1.3	4E-03
NR_045167	4930593A02Rik	1.3	4E-02
ENSMUST00000108857	Atox1	1.3	1E-02
ENSMUST00000079875	Olfr1418	1.3	3E-02
NM_001076790	Serpinb6d	1.3	3E-02
NM_023202	Ndufa7	1.3	4E-02
NM_023483	1110032A03Rik	1.3	3E-02
AK086852	Gm5914	1.3	1E-02
ENSMUST00000079590	Myo1a	1.3	4E-03
ENSMUST00000115645	Ranbp1	1.3	9E-03
XM_001472058	LOC100039029	1.3	5E-02
NM_020044	Lat2	1.3	2E-02
NM_001002842	Pram1	1.3	4E-02
XR_104634	Gm15867	1.3	4E-02
NM_019732	Runx3	1.3	4E-02
NM_008947	Psmc1	1.3	2E-02
NM_001164725	Fcrl6	1.3	2E-02
NM_026138	6330407J23Rik	1.3	3E-02
NM_019449	Unc93b1	1.3	4E-02
ENSMUST00000029419	Veph1	1.3	5E-02
NM_007575	Ciita	1.3	3E-02
ENSMUST00000061725	Prss32	1.3	5E-02
XM_001480228	Gm4454	1.3	4E-02
ENSMUST00000042753	C030048B08Rik	1.3	2E-02
NM_029113	4930447A16Rik	1.3	3E-02
ENSMUST00000023144	Prm1	1.3	2E-02
NM_027960	Dpep3	1.3	1E-02
NR_036451	Gm4814	1.3	5E-02
NM_008558	Max	1.3	2E-02
NR_045333	Gm1715	1.3	4E-02
NM_019402	Pabpn1	1.3	4E-02
NM_021432	Nap1l5	1.3	2E-02

NM_009508	Slc32a1	1.3	5E-02
NM_001177373	Klk11	1.3	2E-02
ENSMUST00000129058	Mtap6	1.3	4E-02
AK007825	Gm15821	1.3	2E-02
ENSMUST00000019876	Calr3	1.3	3E-02
NM_177779	Ccdc42	1.3	4E-02
NR_040619	A930019D19Rik	1.3	5E-02
NR_036459	A730018C14Rik	1.3	5E-02
NR_033562	Gm1995	1.3	3E-02
ENSMUST00000113298	Slc22a29	1.3	5E-02
ENSMUST00000030018	Nans	1.3	5E-02
NM_134155	Brms1	1.3	2E-02
BC100489	4921506M07Rik	1.3	4E-02
NM_001164312	Gm4847	1.3	5E-02
NM_001039047	Trim58	1.3	3E-02
NM_013561	Htr3a	1.3	7E-03
NM_009672	Anp32a	1.3	3E-03
NR_045885	Gm10578	1.3	9E-04
NR_015466	7530420F21Rik	1.3	4E-02
BC049642	4930584F24Rik	1.3	2E-03
ENSMUST00000088876	Olfr1321	1.3	4E-02
NM_010492	Ica1	1.3	2E-02
NM_029230	4930564B18Rik	1.3	5E-02
ENSMUST00000044326	2300002M23Rik	1.3	2E-02
NM_182694	Ggn	1.3	4E-03
NM_001253740	Tpm3	1.3	2E-03
XM_001473737	Gm2483	1.3	4E-02
NM_175223	Dnali1	1.3	2E-02
XR_108007	LOC100505026	1.3	3E-02
ENSMUST00000014445	Pam16	1.3	1E-02
ENSMUST00000038644	Rangrf	1.3	5E-02
NM_010758	Mag	1.3	1E-02
NM_029203	Rhox2a	1.3	5E-02
NM_027194	Tm2d2	1.3	2E-03
NM_013489	Cd84	1.3	4E-02
NM_134166	Vmn1r36	1.3	5E-02
NM_026456	Tceb1	1.3	8E-03
NM_001033922	Treml4	1.3	4E-02
AK020953	B230104C08Rik	1.3	5E-02
ENSMUST00000061262	Podxl2	1.3	2E-02
NM_029283	Fam183b	1.3	4E-02
ENSMUST00000095456	Mcf2l	1.3	2E-03
XR_141144	Gm2386	1.3	3E-02
NM_015732	Axin2	1.3	3E-02
NM_026674	Aph1c	1.3	2E-02
ENSMUST00000041463	Pacrg	1.3	5E-02

NM_001163945	Rpl3l	1.3	2E-02
NR_037987	Gm16548	1.3	4E-02
NM_001159424	Il12a	1.3	3E-02
NM_207278	Tigd4	1.3	4E-02
NM_147120	Olfr638	1.3	4E-03
ENSMUST00000019051	Alox12e	1.3	2E-02
ENSMUST00000125995	Tbcel	1.3	9E-03
NM_178679	Zfp365	1.3	4E-02
NR_040418	2310034G01Rik	1.3	4E-02
NR_038041	2310015B20Rik	1.3	8E-03
ENSMUST00000109757	Bpifb4	1.3	1E-03
XM_003689217	Gm4271	1.3	2E-02
NM_001044384	Timp1	1.3	1E-02
NM_021290	Ucn	1.3	4E-02
NM_153108	Defb8	1.3	2E-02
NM_013920	Hnf4g	1.3	4E-02
BC002135	1810037I17Rik	1.3	4E-02
NM_013532	Lilrb4	1.3	3E-02
NM_009930	Col3a1	1.3	2E-02
NM_010189	Fcgrt	1.3	2E-02
ENSMUST00000047025	Otoa	1.3	4E-02
NM_027855	0610007C21Rik	1.3	2E-03
NM_010903	Nfe2l3	1.3	3E-02
NM_025507	Snw1	1.3	9E-03
NR_028101	Gm12191	1.3	1E-02
NR_045344	4933440J02Rik	1.3	4E-02
NM_153588	Mkl2	1.3	3E-02
NM_001099301	Lcn14	1.3	4E-02
BC051480	Gm5144	1.3	5E-02
ENSMUST00000031607	Dtx1	1.3	3E-02
NM_027344	Ctsll3	1.3	5E-02
NM_198967	Tmtc1	1.3	4E-02
ENSMUST00000025707	Zfpl1	1.3	3E-02
NM_025642	2610039C10Rik	1.3	3E-02
ENSMUST00000028721	Tgm5	1.3	3E-02
ENSMUST00000029208	Slc13a3	1.3	2E-02
NR_045341	5730435O14Rik	1.3	1E-02
NM_001001491	Tpm4	1.3	4E-03
NM_028469	3110082I17Rik	1.3	6E-03
NR_040626	9230105E05Rik	1.3	1E-02
NM_026461	1700129C05Rik	1.3	5E-02
NM_025594	Zmat2	1.3	3E-02
NM_021408	Ush2a	1.3	9E-03
NM_001168588	Vrtn	1.3	5E-02
ENSMUST00000042042	Osta	1.3	5E-02
NM_027619	Ttc14	1.3	4E-02

NM_001162896	4930523C07Rik	1.3	4E-02
NM_001164284	C87414	1.3	6E-04
NM_001040136	Pdzd9	1.3	2E-02
BC052651	BC053393	1.3	5E-02
NM_001008419	Aox3l1	1.3	5E-02
NM_008519	Ltb4r1	1.3	5E-02
XM_003085302	Gm15266	1.3	4E-02
BC099580	1700057K13Rik	1.3	7E-03
NM_008468	Kpna6	1.3	3E-03
NR_045395	4930527G23Rik	1.3	4E-02
NM_198617	Tspyl3	1.3	2E-02
NM_001081012	4930473A06Rik	1.3	4E-02
NM_001031772	Lin28b	1.3	5E-02
XR_031666	Gm8675	1.3	1E-02
NM_029992	Tchp	1.3	4E-02
AK016455	4931413K12Rik	1.3	2E-02
NM_133997	Apof	1.3	3E-02
NM_022891	Rpl23	1.3	2E-02
NM_029982	A930009A15Rik	1.3	2E-02
NR_045928	1700081H22Rik	1.3	2E-02
NM_008268	Hoxb5	1.3	7E-04
NM_029835	5730590G19Rik	1.3	3E-02
NM_011079	Phkg1	1.3	5E-02
XR_104608	Gm15853	1.3	3E-02
NM_181345	Npm2	1.3	4E-02
NM_001081108	0910001L09Rik	1.3	3E-02
NM_177449	Lrrc29	1.3	2E-02
NM_001082532	Pigyl	1.3	3E-02
ENSMUST00000121017	Ccdc155	1.3	2E-02
ENSMUST00000113794	Igsf5	1.3	5E-02
ENSMUST00000117513	Slc29a3	1.3	4E-02
NM_010360	Gstm5	1.3	3E-02
NM_001079869	Hoxb3	1.3	4E-02
NM_021483	Pex5l	1.3	3E-02
ENSMUST00000159292	Osgep	1.3	4E-02
ENSMUST00000168578	Tmem238	1.3	3E-02
NM_015740	Bloc1s1	1.3	6E-03
XR_141781	Gm8702	1.3	7E-03
NM_011888	Ccl19	1.3	6E-03
NM_010012	Cyp8b1	1.3	4E-02
NM_001177513	Gm7102	1.3	1E-02
NM_026027	Pfdn1	1.3	2E-02
NM_139291	Cdc26	1.3	3E-02
NM_172442	Dtx4	1.3	2E-02
NM_012001	Cops4	1.3	5E-02
NR_040709	A930011O12Rik	1.3	5E-02

NM_145423	Slc5a8	1.3	4E-02
ENSMUST00000004614	Zfp110	1.3	3E-02
NM_022007	Fxyd7	1.3	1E-03
XR_140943	LOC100861732	1.2	5E-02
AK084134	Gm12866	1.2	3E-03
ENSMUST00000019638	Cops6	1.2	4E-02
ENSMUST00000076703	Gm11425	1.2	2E-02
NM_007829	Daxx	1.2	5E-02
NM_148926	Zfand3	1.2	1E-03
NM_001085348	Rhox2e	1.2	5E-02
NM_153114	Otos	1.2	1E-02
NM_001166389	Ptp4a3	1.2	1E-03
NR_040337	Gm16982	1.2	2E-02
U92703	Ebf2	1.2	2E-02
NM_026610	Ndufb4	1.2	3E-02
NM_027859	Rnf215	1.2	2E-02
NM_007801	Ctsh	1.2	4E-02
BC089026	2310008H04Rik	1.2	5E-03
ENSMUST00000025631	Ostf1	1.2	3E-02
NM_008948	Psmc3	1.2	1E-02
NM_001163721	1190007F08Rik	1.2	3E-02
ENSMUST00000030986	Lrpap1	1.2	2E-02
NM_001033394	Tmem88b	1.2	4E-02
BC103775	Fam136a	1.2	6E-03
NM_008119	Gip	1.2	2E-02
NM_001163749	Camsap3	1.2	7E-03
NM_011282	Ros1	1.2	4E-02
AK129284	Ankhd1	1.2	2E-02
NR_045423	Gm15997	1.2	4E-02
ENSMUST00000025944	Hhex	1.2	2E-02
NM_022023	Gmfb	1.2	9E-03
NM_025394	Tomm7	1.2	2E-02
NM_153551	Dennd1c	1.2	2E-02
NM_011428	Snap25	1.2	5E-02
NM_029959	Lcn9	1.2	5E-02
NM_025836	Plin3	1.2	4E-02
NM_010584	Itln1	1.2	4E-02
NM_019637	Styx	1.2	3E-02
NR_040673	LOC622167	1.2	1E-02
XM_001474574	LOC100040377	1.2	2E-02
ENSMUST00000089559	Ddrgk1	1.2	4E-02
NM_207707	Esr2	1.2	2E-02
NM_001081153	Unc13c	1.2	4E-02
NM_177774	Srsf12	1.2	2E-02
NM_001037744	Timm8a2	1.2	4E-03
NM_021416	Fam184b	1.2	2E-02

NR_015572	1810014B01Rik	1.2	4E-02
NM_001160178	Klhdc7b	1.2	2E-02
NR_040537	4930529L06Rik	1.2	3E-02
NM_153090	Fcrl1	1.2	5E-02
ENSMUST00000025161	Tapbp	1.2	4E-02
NM_027651	Tmem30c	1.2	4E-02
NM_026724	Rpl34	1.2	3E-02
ENSMUST00000037360	Rhov	1.2	4E-02
NM_025323	0610009D07Rik	1.2	5E-03
XM_003689369	LOC100862066	1.2	3E-02
NM_146257	Slc29a4	1.2	3E-02
BC050813	Vwa3b	1.2	1E-02
NM_010441	Hmga2	1.2	4E-03
ENSMUST00000064473	Adarb2	1.2	3E-02
NM_025757	4933439F18Rik	1.2	2E-02
NM_011783	Agr2	1.2	4E-02
NM_010948	Nudc	1.2	5E-03
NM_177328	Grm7	1.2	2E-02
NM_026065	Mrpl42	1.2	3E-02
NM_029306	1700012B09Rik	1.2	4E-02
NM_027966	1700019O17Rik	1.2	5E-02
NM_001136240	Chdh	1.2	4E-02
NM_011613	Tnfsf11	1.2	2E-02
NM_001114079	Pabpc1l	1.2	4E-02
AK086556	Gm14133	1.2	3E-02
NM_009710	Art1	1.2	2E-02
ENSMUST00000115609	Comt	1.2	3E-02
NM_008191	Guca2b	1.2	5E-02
ENSMUST00000147399	Cd101	1.2	3E-02
NM_203345	Ltk	1.2	2E-02
NR_038141	1700008K24Rik	1.2	1E-03
NM_181452	Fam89b	1.2	3E-02
NR_037977	Gm53	1.2	1E-02
ENSMUST00000015017	Surf2	1.2	5E-02
NM_030087	Ndufv3	1.2	7E-03
NM_011542	Tcea3	1.2	5E-02
NR_040303	LOC433374	1.2	4E-02
NM_024474	Emid2	1.2	3E-02
NM_008161	Gpx3	1.2	4E-02
NM_053171	Csmd1	1.2	4E-02
ENSMUST00000120135	Syk	1.2	7E-03
ENSMUST00000065574	Tm2d3	1.2	3E-02
NM_028013	Endod1	1.2	3E-02
NM_011638	Tfrc	-2.7	2E-02
ENSMUST00000087328	Hspa1a	-2.6	2E-02
NM_146810	Olfr912	-2.5	2E-02

NM_001199048	Zfp942	-2.4	2E-02
NM_153093	AF366264	-2.2	4E-02
NR_040704	4930563F08Rik	-2.2	2E-02
XR_107070	BE949265	-2.1	2E-02
NM_207555	Olfr372	-2.1	3E-02
XR_105087	Gm6579	-2.1	4E-02
ENSMUST00000142180	Zfp932	-2.0	2E-02
NM_020275	Tnfrsf10b	-2.0	6E-03
NM_153287	Csrnp1	-2.0	7E-04
NM_001105181	Vmn2r68-ps	-2.0	3E-02
NM_134171	Vmn1r17	-2.0	2E-02
ENSMUST00000050562	Ch25h	-2.0	4E-03
NM_009516	Wee1	-2.0	3E-02
NR_045696	9330133O14Rik	-1.9	1E-03
XR_107634	LOC100505156	-1.9	1E-02
NM_175303	Sall4	-1.9	5E-04
ENSMUST00000014957	Stc1	-1.9	3E-02
NM_177256	Prdx6b	-1.9	1E-02
ENSMUST00000001384	Cnn1	-1.9	5E-03
NM_008209	Mr1	-1.9	2E-04
NM_009485	Vmn2r123	-1.9	4E-02
ENSMUST00000170320	Agbl2	-1.8	2E-02
ENSMUST00000020849	Tom1l1	-1.8	4E-02
NR_003292	Zxda	-1.8	4E-03
NR_045963	Gm1976	-1.8	6E-04
NM_145827	Nlrp3	-1.8	2E-03
NM_028133	Egln3	-1.8	2E-02
NM_001011796	Olfr1249	-1.8	5E-02
NR_037964	2610507I01Rik	-1.8	2E-02
NR_045798	1810034E14Rik	-1.8	3E-03
NM_001104565	Vmn2r103	-1.8	9E-03
NM_172146	Ppat	-1.8	4E-03
NM_178739	Dcaf12l1	-1.8	3E-03
NM_145577	Zfp772	-1.8	2E-02
ENSMUST00000076331	Olfr127	-1.8	3E-02
NM_011898	Spry4	-1.8	5E-02
NM_001104642	Vmn2r27	-1.8	4E-02
NM_011994	Abcd2	-1.8	2E-02
NM_001081473	Zxdb	-1.8	1E-02
AY288427	Gpr141	-1.7	3E-02
NM_001101479	Pabpc4l	-1.7	2E-02
ENSMUST00000061405	Pcdhb21	-1.7	3E-02
ENSMUST00000105105	Hist2h3b	-1.7	9E-03
NR_045865	9230009I02Rik	-1.7	1E-02
NM_146907	Olfr1282	-1.7	3E-02
NM_053235	Vmn1r13	-1.7	5E-02

XM_003689464	LOC100862124	-1.7	3E-02
NM_175191	Gpr22	-1.7	7E-03
NM_177359	Zfp799	-1.7	5E-02
NM_001126491	Gm14459	-1.7	5E-02
NM_026527	Chac2	-1.7	6E-04
NM_146463	Olfr1204	-1.7	3E-02
NM_033563	Klf7	-1.7	3E-03
NM_027981	2310002L09Rik	-1.7	2E-02
NM_001033539	Bex6	-1.7	5E-02
NM_010373	Gzme	-1.7	3E-02
ENSMUST00000053652	Tas2r105	-1.7	2E-02
NM_011400	Slc2a1	-1.7	3E-04
ENSMUST00000076463	Gpr155	-1.7	4E-03
BC061459	D19Ertd737e	-1.7	1E-02
NM_146349	Olfr1128	-1.7	3E-02
NM_001109914	Apold1	-1.7	3E-02
NM_001003666	Zfp457	-1.7	2E-02
ENSMUST00000075433	Cxcl2	-1.6	5E-02
NM_001081306	Ptprz1	-1.6	4E-02
NM_177204	Fam40b	-1.6	5E-03
NM_145565	Sds	-1.6	5E-02
NM_183309	Gm5111	-1.6	2E-02
NR_046302	AF357425	-1.6	3E-02
NR_027810	1110006O24Rik	-1.6	1E-02
BC053409	Ighm	-1.6	4E-02
AK005461	Tbc1d5	-1.6	5E-03
NM_007570	Btg2	-1.6	4E-02
NM_144912	Rad9b	-1.6	4E-04
NM_009639	Crisp3	-1.6	4E-03
NM_053138	Pcdhb13	-1.6	7E-03
NM_013935	Ptpla	-1.6	4E-02
M17720	Igkv4-59	-1.6	5E-02
ENSMUST00000025875	Slc1a1	-1.6	5E-02
NM_001033383	Zfp865	-1.6	1E-02
AK016643	4933403O03Rik	-1.6	4E-02
NM_021275	Kcna4	-1.6	6E-03
NM_010453	Hoxa5	-1.6	2E-03
NM_001114529	Gm4906	-1.6	4E-02
ENSMUST00000011445	Cd209d	-1.6	5E-02
NM_001033415	Shisa3	-1.6	6E-03
NM_026058	Lass4	-1.6	1E-03
BC024071	Serpina4-ps1	-1.6	5E-03
NM_001166497	3110052M02Rik	-1.6	1E-02
NM_031261	Fthl17	-1.6	4E-02
NM_001039373	Mtcp1	-1.6	4E-02
ENSMUST00000120432	Mlkl	-1.6	3E-02

NM_001163013	Ythdc2	-1.6	7E-04
NM_001163473	1810010H24Rik	-1.6	3E-02
ENSMUST00000063750	Rarb	-1.6	2E-03
NM_175318	Spty2d1	-1.6	4E-03
NM_145456	Zswim6	-1.6	6E-03
NM_133485	Ppp1r14c	-1.6	1E-02
NM_001001309	Itga8	-1.6	1E-02
NR_045392	Gm10731	-1.6	3E-02
NM_027388	Pigw	-1.6	5E-02
NM_018791	Zfp108	-1.6	4E-02
NM_001159275	Slc25a2	-1.6	5E-03
NM_001243067	Zfp433	-1.6	2E-02
NM_146930	Olfr791	-1.6	5E-02
NM_013869	Tnfrsf19	-1.6	2E-03
NM_001010840	Taar8c	-1.6	3E-02
NM_176844	Chrna5	-1.6	2E-02
NM_020604	Jph1	-1.6	5E-04
NM_145959	D15Ertd621e	-1.6	1E-02
NR_033219	BC020402	-1.6	2E-02
NM_029508	Pcgf5	-1.6	4E-03
NM_007392	Acta2	-1.6	7E-03
NM_146926	Olfr477	-1.6	4E-02
AB540944	Vmn2r111	-1.6	2E-02
NM_024285	Bves	-1.6	3E-03
NM_207703	Olfr1335	-1.6	1E-02
NM_182995	6330503K22Rik	-1.6	4E-02
NM_001134902	AU019823	-1.5	3E-02
NM_021477	Rbfox1	-1.5	9E-05
BC137951	Fam78a	-1.5	2E-03
NM_021555	Fam203a	-1.5	4E-02
NM_001100464	Abpb	-1.5	5E-02
NM_013647	Rps16	-1.5	3E-02
NM_013636	Ppp1cc	-1.5	1E-02
NM_175175	Plekhf2	-1.5	4E-02
NM_183174	Homez	-1.5	3E-02
NM_177872	Adamts3	-1.5	6E-05
NM_177660	Zbtb10	-1.5	3E-02
XM_894578	Gm6993	-1.5	1E-02
NM_010141	Epha7	-1.5	5E-03
ENSMUST00000030464	Pik3r3	-1.5	7E-03
NM_001081655	Dact3	-1.5	6E-05
BC147387	Gm889	-1.5	2E-02
NM_001199003	Rgs7	-1.5	1E-03
NM_011809	Ets2	-1.5	3E-03
ENSMUST00000174397	Prrx1	-1.5	2E-03
NM_133798	Exd2	-1.5	3E-03

NM_022890	Cldn12	-1.5	3E-02
NM_054087	Slc19a2	-1.5	1E-03
NM_026121	Bag4	-1.5	2E-02
NM_001081112	Ankrd26	-1.5	1E-02
NM_177635	H2-M11	-1.5	4E-03
NM_130450	Elovl6	-1.5	2E-02
NR_037997	2310015D24Rik	-1.5	2E-02
NM_029068	Snx16	-1.5	5E-02
NM_009741	Bcl2	-1.5	1E-02
NM_007904	Ednrb	-1.5	4E-02
ENSMUST00000108379	BC024978	-1.5	1E-02
NM_026252	Cpeb4	-1.5	7E-03
NM_205821	Mrgpra6	-1.5	4E-02
NM_175029	Atg4c	-1.5	2E-02
NM_001163170	Lix1l	-1.5	1E-03
NM_027890	Susd2	-1.5	1E-02
NM_024290	Tnfrsf23	-1.5	5E-02
NM_001164818	0610011L14Rik	-1.5	1E-02
ENSMUST00000109604	Rbm12	-1.5	3E-02
NM_013807	Plk3	-1.5	1E-02
NM_009601	Chrnb1	-1.5	1E-02
ENSMUST00000032233	Tuba8	-1.5	4E-02
NM_178869	Ttll1	-1.5	9E-03
ENSMUST00000085745	Wdr35	-1.5	1E-02
NM_001146275	Iigp1	-1.5	5E-02
AK015602	4930480K23Rik	-1.5	1E-02
NM_001081263	Slc44a5	-1.5	1E-02
NM_001001186	Zfp456	-1.5	5E-02
NM_146882	Olfr874	-1.5	2E-02
NM_019960	Hspb3	-1.5	5E-02
ENSMUST00000068911	Gabrg3	-1.5	3E-02
NM_175028	Adnp2	-1.5	4E-02
NM_010113	Egf	-1.5	2E-02
NM_001013833	Prkg1	-1.5	9E-03
NR_038065	LOC171588	-1.5	4E-02
NM_009447	Tuba4a	-1.5	3E-02
NM_008115	Gfra2	-1.5	4E-02
NM_146359	Olfr564	-1.5	3E-02
AK019918	5330429C05Rik	-1.5	4E-02
NM_010436	H2afx	-1.5	1E-02
NM_017378	Pcdh12	-1.5	2E-02
NM_001085421	Tspyl5	-1.5	3E-02
NM_145401	Prkag2	-1.5	8E-04
AF054620	Aurkc	-1.5	3E-02
NM_177805	Fam179b	-1.5	1E-02
ENSMUST00000108465	Olfr397	-1.5	5E-03

NM_001081281	Trim55	-1.5	2E-02
NM_008301	Hspa2	-1.5	3E-03
NM_020513	Olfr1508	-1.5	5E-02
ENSMUST00000059354	Sigmar1	-1.5	4E-03
NR_030699	A630072M18Rik	-1.5	3E-02
NM_172399	A930038C07Rik	-1.5	3E-02
NM_001080935	B230217C12Rik	-1.5	3E-02
ENSMUST00000039177	Dpyd	-1.5	2E-02
ENSMUST00000027053	Rdh10	-1.5	1E-02
NM_177353	Slc9a7	-1.5	1E-02
ENSMUST00000042391	Fdxacb1	-1.5	8E-03
ENSMUST00000033450	Gpc4	-1.5	2E-02
ENSMUST00000031689	Hyal5	-1.5	2E-02
NM_198702	Lphn3	-1.5	1E-02
NM_172134	Pdxk	-1.5	5E-03
NM_146612	Olfr968	-1.5	2E-02
NM_019576	Thsd1	-1.5	2E-02
NM_207522	BC067068	-1.5	1E-03
NM_001166365	Fam122b	-1.5	4E-02
NM_011200	Ptp4a1	-1.5	2E-03
BC147107	D13Ertd608e	-1.5	2E-02
NM_001166282	Ccdc33	-1.5	2E-02
ENSMUST00000033671	Rps6ka3	-1.5	8E-03
NM_001136090	Poli	-1.5	4E-02
NM_008830	Abcb4	-1.5	1E-02
NM_206872	Vmn1r177	-1.5	5E-02
ENSMUST00000074730	Olfr512	-1.5	4E-02
NM_001166064	Syde2	-1.5	1E-03
ENSMUST00000086978	Cdkl2	-1.5	1E-02
NM_009152	Sema3a	-1.5	2E-02
XM_003689302	LOC100861916	-1.5	3E-02
ENSMUST00000036796	Fstl4	-1.5	1E-02
NM_177161	P4ha3	-1.5	8E-03
NR_040306	6720468P15Rik	-1.5	2E-02
NM_175251	Arid2	-1.5	1E-02
NM_025478	Isoc1	-1.5	1E-03
NM_029951	C330007P06Rik	-1.5	3E-03
ENSMUST00000053958	Olfr303	-1.5	1E-02
NM_011346	Sell	-1.5	4E-02
NM_019501	Pdss1	-1.5	5E-03
ENSMUST00000107833	Prrg2	-1.5	3E-02
NM_001110832	Nfya	-1.5	6E-03
NM_145128	Mgat5	-1.5	3E-02
NM_001145924	5730528L13Rik	-1.5	1E-02
ENSMUST00000114410	Kctd18	-1.5	2E-02
NM_028131	Cenpn	-1.5	3E-02

NM_001170454	Tada2b	-1.5	1E-02
ENSMUST00000029405	Gmps	-1.5	5E-03
ENSMUST00000056176	Vav2	-1.4	3E-03
ENSMUST00000031354	Abcb9	-1.4	1E-03
ENSMUST00000036951	Pebp1	-1.4	1E-02
ENSMUST00000027785	Sdccag8	-1.4	2E-02
NM_001145820	Gpd2	-1.4	2E-02
NM_001013368	E2f8	-1.4	3E-02
ENSMUST00000135345	LOC100505283	-1.4	1E-02
NM_021527	Mkks	-1.4	2E-02
NM_001039967	Zfp869	-1.4	5E-02
NM_009577	Zik1	-1.4	5E-02
NM_001109040	Kif21a	-1.4	5E-03
NM_175515	Intu	-1.4	6E-04
NM_010258	Gata6	-1.4	5E-04
NM_026005	2610301B20Rik	-1.4	2E-03
NR_051982	Dennd2d	-1.4	1E-02
ENSMUST00000041100	Alkbh4	-1.4	1E-02
NM_009216	Sstr1	-1.4	4E-02
XR_141675	Gm19605	-1.4	1E-02
NM_133769	Cyfip2	-1.4	5E-02
NM_001177719	Madd	-1.4	2E-02
NM_019752	Htra2	-1.4	7E-03
NM_172812	Htr2a	-1.4	1E-02
NM_001100449	Taf4b	-1.4	2E-02
NM_178143	Prkaa2	-1.4	3E-03
NM_145547	Zfp189	-1.4	8E-03
NM_173442	Gcnt1	-1.4	2E-02
NM_001011523	Olfr913	-1.4	1E-02
XM_003086743	Gm7866	-1.4	4E-02
NM_026104	Tmco5	-1.4	5E-02
NM_019518	Grasp	-1.4	3E-02
ENSMUST00000114769	Fhl1	-1.4	1E-02
NM_029701	Spcs3	-1.4	2E-02
NM_021377	Sorcs1	-1.4	4E-02
NM_001081405	Vmn2r28	-1.4	5E-02
NM_001033225	Pnrc1	-1.4	5E-03
NM_146074	Tfb1m	-1.4	1E-02
NM_011882	Rnasel	-1.4	2E-02
NR_045886	Gm10658	-1.4	8E-03
NM_199199	Tmem199	-1.4	7E-03
NM_001142724	Kbtbd12	-1.4	8E-03
NM_172385	Zfp536	-1.4	3E-02
NM_007974	F2rl1	-1.4	2E-02
NM_026742	Ndufaf4	-1.4	1E-02
NR_046279	4930483O08Rik	-1.4	2E-02

NM_027711	Iqgap2	-1.4	3E-03
NM_008748	Dusp8	-1.4	3E-02
NM_001195537	Gm10345	-1.4	2E-03
ENSMUST00000111718	Tfpi	-1.4	2E-03
ENSMUST00000036273	Nfkbiz	-1.4	3E-02
NM_001081157	Lmod3	-1.4	3E-02
NM_172126	Adam1a	-1.4	4E-02
NM_001099742	Scgb1c1	-1.4	4E-02
NR_027974	3110070M22Rik	-1.4	2E-02
NM_007730	Col12a1	-1.4	5E-02
NR_030716	5430417L22Rik	-1.4	1E-04
NM_029031	Shpk	-1.4	7E-03
NM_017382	Rab11a	-1.4	2E-02
NM_001163572	Tmem170b	-1.4	6E-03
NR_051981	H2-Q5	-1.4	5E-02
NR_040399	4930570G19Rik	-1.4	2E-02
NM_001122733	Kit	-1.4	2E-02
ENSMUST00000037035	Ripk2	-1.4	2E-02
NM_008904	Ppargc1a	-1.4	2E-03
NM_172863	Zfp697	-1.4	4E-02
NM_011862	Pacsin2	-1.4	1E-02
NM_145939	Alg3	-1.4	2E-02
NM_029075	Stx11	-1.4	5E-02
NM_173376	Rbmx2	-1.4	4E-02
NM_026112	Zfp606	-1.4	2E-02
NM_001110216	Cbx5	-1.4	2E-02
NM_001081282	Ibtk	-1.4	4E-04
BC132158	4931406B18Rik	-1.4	4E-02
BC064105	2610203C22Rik	-1.4	5E-02
NM_028651	Tmtc4	-1.4	7E-03
AK145303	Gm10747	-1.4	2E-02
NM_025522	Dhrs7	-1.4	1E-02
NM_053169	Trim16	-1.4	4E-02
NM_021554	Mettl9	-1.4	4E-02
NR_045422	1700018G05Rik	-1.4	2E-02
NM_001024624	Cdkl5	-1.4	2E-02
ENSMUST00000068045	Actn4	-1.4	2E-02
NM_001012269	Olfr1513	-1.4	3E-02
NM_178669	Clrn3	-1.4	1E-02
NM_019519	Rabggta	-1.4	1E-03
NM_175374	Mtrf1l	-1.4	2E-02
NM_133999	Fig4	-1.4	1E-02
NM_178890	Abtb2	-1.4	1E-02
NM_001167818	Rnls	-1.4	3E-02
NM_023418	Pgam1	-1.4	5E-03
NM_009266	Sephs2	-1.4	4E-03

NM_178214	Hist2h2be	-1.4	3E-02
ENSMUST00000159816	Filip1l	-1.4	3E-03
ENSMUST00000059675	Olfr1444	-1.4	3E-02
NR_028315	Yaf2	-1.4	2E-03
NR_027652	Meg3	-1.4	3E-02
NM_016859	Bysl	-1.4	6E-03
NM_001033145	1190002N15Rik	-1.4	6E-03
NM_028059	Zfp654	-1.4	2E-02
ENSMUST00000167797	Agxt2l2	-1.4	2E-03
NM_019971	Pdgfc	-1.4	4E-02
ENSMUST00000094451	Gpr157	-1.4	7E-03
ENSMUST00000028817	Pcna	-1.4	5E-03
ENSMUST00000069097	Eif2c3	-1.4	5E-02
ENSMUST00000022765	Rab2b	-1.4	2E-02
NM_001199485	4931406C07Rik	-1.4	9E-03
NM_010427	Hgf	-1.4	3E-02
AB097847	Ighg	-1.4	3E-02
NM_172821	Map3k13	-1.4	3E-02
NM_001255990	Gm1553	-1.4	2E-02
ENSMUST00000119878	Dyrk1a	-1.4	9E-05
NM_177843	Gm14461	-1.4	9E-03
NM_197987	Trim37	-1.4	2E-02
NM_001254761	Rnf128	-1.4	2E-02
NM_011406	Slc8a1	-1.4	9E-03
NM_133225	Acbd3	-1.4	2E-03
XM_891513	Rpl31-ps21	-1.4	4E-02
NM_001033306	Shb	-1.4	3E-02
NM_178404	Zc3h6	-1.4	3E-02
ENSMUST00000028377	Scn2a1	-1.4	5E-02
ENSMUST00000063690	Dhrs9	-1.4	2E-02
NM_011712	Wbp5	-1.4	3E-02
NM_008622	Mpv17	-1.4	2E-02
ENSMUST00000109986	Ralgapa2	-1.4	2E-02
NR_046193	4930563M20Rik	-1.4	4E-02
NM_029128	Qtrtd1	-1.4	1E-02
NR_027445	A230072C01Rik	-1.4	3E-02
ENSMUST00000020273	Supv3l1	-1.4	1E-03
ENSMUST00000014562	Hps5	-1.4	4E-02
NM_011535	Tbx3	-1.4	5E-02
NM_028945	D14Abb1e	-1.4	5E-02
ENSMUST00000122965	Elp4	-1.4	4E-02
NM_029875	Slc35e3	-1.4	4E-02
NM_146118	Slc25a25	-1.4	7E-03
ENSMUST00000048096	Tlr4	-1.4	2E-02
NR_036590	1700001K23Rik	-1.4	5E-02
NR_027956	1700052K11Rik	-1.4	4E-04

BC138202	2010002M12Rik	-1.4	3E-02
NM_001085507	Zbtb34	-1.4	4E-02
NM_029536	Gpr165	-1.4	3E-02
NM_001030294	Olfm4	-1.4	3E-02
NM_001024135	Kbtbd7	-1.4	7E-03
NM_146365	Olfr1094	-1.4	2E-02
NM_026443	Mtfp1	-1.4	3E-02
NM_011505	Stxbp4	-1.4	6E-03
ENSMUST00000112916	Nxt2	-1.4	2E-02
ENSMUST00000066650	Dbr1	-1.4	4E-02
ENSMUST00000153294	5830433M19Rik	-1.4	2E-02
NR_045431	4933401D09Rik	-1.4	2E-02
ENSMUST00000114705	Tmem25	-1.4	2E-02
NM_178065	1110018G07Rik	-1.4	4E-03
NM_172814	Lrp12	-1.4	4E-02
NM_026837	Tmem53	-1.4	2E-02
ENSMUST00000029196	Slc2a10	-1.4	5E-02
ENSMUST00000108824	Olfr329-ps	-1.4	2E-02
NM_018809	Ptf1a	-1.4	7E-03
NM_029510	Bcor	-1.4	3E-03
NM_178079	Pm20d1	-1.4	4E-02
ENSMUST00000147695	Lonrf2	-1.4	4E-02
ENSMUST00000045351	Atg2a	-1.4	6E-04
ENSMUST00000018965	Avpi1	-1.4	2E-03
NM_001029842	Slc16a6	-1.4	6E-03
NM_201367	Gpr176	-1.4	5E-04
NM_009740	Bcl10	-1.4	1E-02
NM_001167872	Zfp568	-1.4	3E-02
ENSMUST00000023510	Umps	-1.4	6E-03
NR_027801	Gm1141	-1.4	1E-02
NM_011727	Xlr3c	-1.4	4E-02
AK041907	2010015M23Rik	-1.4	1E-02
NM_178663	Bend7	-1.4	4E-02
ENSMUST00000023043	Adsl	-1.4	1E-03
NM_009632	Parp2	-1.4	4E-02
NM_001109757	Atp7a	-1.4	3E-02
NM_028029	Dnmbp	-1.4	5E-03
NM_013904	Hey2	-1.4	5E-02
NM_023503	Ing2	-1.4	4E-02
NM_175565	Cdv3	-1.4	2E-02
NM_001198872	Dync1i2	-1.4	6E-03
NR_045795	Gm16702	-1.4	5E-02
NM_001164676	Zfp229	-1.4	4E-02
NM_025904	1600012F09Rik	-1.4	2E-02
ENSMUST00000107479	Rapgefl1	-1.4	3E-02
NM_177747	Zfp711	-1.4	5E-02

ENSMUST00000031072	Anapc4	-1.4	4E-02
NM_146042	Rnf144b	-1.4	1E-02
NR_045419	A530050N04Rik	-1.4	3E-02
NM_029447	Nln	-1.4	3E-02
NM_178674	Fbxl21	-1.4	6E-03
NR_036602	4933421O10Rik	-1.4	2E-02
NM_030595	Nbea	-1.4	1E-02
NM_001170489	Aplf	-1.4	6E-03
NM_146997	Olfr178	-1.4	1E-02
ENSMUST00000085206	Slc25a36	-1.4	1E-03
NM_026179	Abhd5	-1.4	1E-03
NM_013855	Abca3	-1.4	2E-02
NM_028118	Wdsub1	-1.4	2E-02
NM_172700	Zmpste24	-1.4	8E-03
ENSMUST00000027071	Lactb2	-1.4	2E-02
NM_011943	Map2k6	-1.4	8E-03
NM_177606	Plekhh2	-1.4	3E-02
NM_021511	Rrs1	-1.4	3E-02
NM_011498	Bhlhe40	-1.4	3E-02
NM_010048	Dgcr2	-1.4	5E-04
ENSMUST00000027494	Ppp1r7	-1.4	2E-02
NM_001085440	Smcr8	-1.4	7E-05
ENSMUST00000052204	Nipa1	-1.4	3E-02
BC100353	1700042B14Rik	-1.4	3E-02
NM_019676	Plcd1	-1.4	6E-03
AK019250	2810030D12Rik	-1.4	4E-02
NM_172867	Zfp462	-1.4	4E-02
NM_173865	Slc41a1	-1.4	7E-03
NM_175127	Fbxo28	-1.4	2E-03
NM_181325	Slc25a15	-1.4	5E-02
NM_029891	Nkrf	-1.4	2E-02
ENSMUST00000085668	Gm5113	-1.4	5E-03
NM_178789	Tmem117	-1.4	2E-02
NM_022030	Sv2a	-1.4	4E-02
NM_013726	Dbf4	-1.4	3E-02
XR_141257	LOC100505224	-1.4	1E-03
NM_001080924	Znrf3	-1.4	2E-03
NM_027289	Nt5dc2	-1.4	3E-02
ENSMUST00000082027	Olfr149	-1.4	3E-02
NM_026984	Mll5	-1.4	3E-02
NM_181266	Zfp120	-1.4	4E-02
NM_001012402	Hs3st6	-1.4	3E-02
ENSMUST00000028035	Cenpl	-1.4	2E-02
NM_011766	Zfpm2	-1.4	2E-02
NM_001122992	Gmeb1	-1.4	8E-03
NM_001100451	Msl2	-1.4	2E-02

NM_008057	Fzd7	-1.4	2E-02
NR_033510	1500017E21Rik	-1.4	3E-02
NM_030614	Fgf16	-1.4	4E-02
NM_146716	Olfr432	-1.4	3E-02
NM_011069	Pex11b	-1.4	2E-03
NM_011600	Tle4	-1.4	5E-02
NM_016910	Ppm1d	-1.4	2E-02
NM_173423	Fem1c	-1.4	2E-03
BC096606	D230037D09Rik	-1.4	4E-02
XR_105139	Gm19765	-1.4	3E-02
NM_015810	Polg2	-1.4	2E-02
NM_153803	Glb1l2	-1.4	1E-02
NM_027444	Bbx	-1.4	4E-02
ENSMUST00000006587	Tmem189	-1.4	1E-03
ENSMUST00000102504	Myo1c	-1.4	1E-02
NR_040644	4933428C19Rik	-1.4	5E-02
ENSMUST00000066378	Rsl24d1	-1.4	4E-02
NM_024437	Nudt7	-1.4	2E-02
NM_028419	Glrx5	-1.4	1E-03
NM_025660	Ribc1	-1.4	5E-02
ENSMUST00000031788	Hibadh	-1.4	2E-03
NM_198612	Gxylt2	-1.4	4E-02
NM_181073	Plekhh1	-1.4	2E-02
NM_001136069	Ldha	-1.4	1E-02
NR_030779	Ankrd10	-1.4	2E-02
NM_172120	Vps41	-1.4	2E-02
NM_133853	Magi3	-1.4	3E-02
NM_001081163	Chsy1	-1.4	7E-03
NM_028882	Sema3d	-1.4	4E-02
ENSMUST00000102542	Atpbd4	-1.4	2E-02
NM_010191	Fdft1	-1.4	9E-03
NM_008258	Hn1	-1.4	2E-04
ENSMUST00000024967	Msh2	-1.4	4E-02
ENSMUST00000067543	Trib1	-1.4	3E-03
XR_141416	Gm4994	-1.4	5E-02
NM_007560	Bmpr1b	-1.4	3E-02
NM_001145978	Parp4	-1.4	1E-02
NM_001199351	Pnck	-1.4	3E-02
NM_001195084	Plscr2	-1.3	4E-02
NM_028696	Obfc2a	-1.3	1E-02
NM_001166030	Mylk4	-1.3	4E-02
NR_027865	1700054K19Rik	-1.3	5E-02
NM_013616	Olfr65	-1.3	4E-02
NM_025566	Tnfaip8l1	-1.3	2E-02
NM_011890	Sgcb	-1.3	1E-02
ENSMUST00000016488	Ppdpf	-1.3	2E-02

NM_028705	Herc3	-1.3	4E-02
BC099383	E330017A01Rik	-1.3	2E-02
ENSMUST00000115188	Rhox3c	-1.3	7E-05
NM_147219	Abca5	-1.3	2E-02
NM_025590	Acot11	-1.3	8E-03
NM_022032	Perp	-1.3	3E-02
NM_001040400	Tet2	-1.3	9E-03
NM_026039	Med18	-1.3	6E-03
NM_001081087	Kbtbd10	-1.3	3E-02
NM_025900	Dek	-1.3	5E-03
NM_172506	Boc	-1.3	5E-02
ENSMUST00000165443	Nup50	-1.3	3E-02
NM_178603	Mrpl50	-1.3	2E-02
NM_001033268	Fam120a	-1.3	1E-03
NM_011732	Ybx1	-1.3	2E-02
NM_001081200	Crnn	-1.3	5E-02
NM_138753	Hexim1	-1.3	2E-02
ENSMUST00000041621	Lipt1	-1.3	1E-02
NM_016679	Keap1	-1.3	1E-02
ENSMUST00000168088	Gm6337	-1.3	2E-02
NM_001195284	Fsd1l	-1.3	2E-02
NM_199012	Fchsd2	-1.3	1E-02
NM_007530	Bcap29	-1.3	4E-02
NM_010288	Gja1	-1.3	4E-02
NM_025830	Wwp2	-1.3	1E-02
NM_027227	Glod5	-1.3	5E-02
NM_026219	Uqcrb	-1.3	1E-02
ENSMUST00000055506	Gtf3c1	-1.3	7E-04
NM_001080711	Dfnb59	-1.3	3E-02
NM_001205219	Sorbs2	-1.3	1E-02
NM_145958	Kbtbd2	-1.3	1E-02
NM_029952	Zfp955a	-1.3	2E-02
NR_033584	Gm10280	-1.3	2E-02
NM_175482	Usp28	-1.3	5E-03
NM_027442	Ddo	-1.3	3E-02
ENSMUST00000036333	Prkx	-1.3	5E-03
NR_036615	Srsf7	-1.3	6E-06
NM_030152	Nol3	-1.3	1E-02
AK149623	D1Ertd448e	-1.3	2E-02
NM_015829	Slc25a13	-1.3	3E-02
NR_027704	Svip	-1.3	8E-03
NM_007964	Evi5	-1.3	4E-02
NM_001007570	Slc25a42	-1.3	6E-03
NM_028749	Npl	-1.3	4E-02
NM_198308	Pdpr	-1.3	2E-03
NM_018879	Nprl2	-1.3	4E-02

NM_028376	Pfn4	-1.3	5E-02
NM_146639	Olfr1138	-1.3	3E-03
NM_009126	Serpinb3a	-1.3	2E-02
NM_080448	Srgap3	-1.3	1E-03
NM_001081001	Brca2	-1.3	3E-02
NM_008773	P2ry2	-1.3	4E-02
ENSMUST00000056508	Clcn3	-1.3	2E-02
NM_010332	Ednra	-1.3	1E-02
NM_030235	Avl9	-1.3	3E-02
NM_010244	Fv1	-1.3	3E-02
NM_025855	Echdc1	-1.3	4E-02
ENSMUST00000038014	Dnajc16	-1.3	2E-03
NM_145390	Tnpo2	-1.3	3E-04
NM_146216	Vac14	-1.3	2E-02
ENSMUST00000033539	F8	-1.3	4E-02
NM_001033410	Gm757	-1.3	3E-02
ENSMUST00000114988	Itfg3	-1.3	1E-02
NM_001164625	2210021J22Rik	-1.3	2E-02
NM_146189	Mybpc2	-1.3	2E-02
NM_133721	Itga9	-1.3	2E-02
BC028495	2810428I15Rik	-1.3	4E-02
NM_015771	Lats2	-1.3	5E-02
NM_021788	Sap30	-1.3	9E-03
NM_175750	Plxna4	-1.3	1E-02
ENSMUST00000030469	1520402A15Rik	-1.3	3E-02
NM_172783	Phka2	-1.3	4E-02
NM_026921	Isca1	-1.3	5E-03
NM_178113	Ncapd3	-1.3	6E-03
NM_016858	Rab33b	-1.3	9E-04
NM_001081145	Tigd2	-1.3	2E-03
NM_018744	Sema6a	-1.3	5E-02
NM_009783	Cacna1g	-1.3	8E-03
ENSMUST00000088217	Tbl1x	-1.3	7E-04
NM_001164497	Papd5	-1.3	6E-03
NR_040657	D830005E20Rik	-1.3	2E-02
NR_037590	Wls	-1.3	1E-02
NM_009761	Bnip3l	-1.3	2E-02
NM_007395	Acvr1b	-1.3	7E-03
NM_178908	Fam26e	-1.3	9E-03
NM_177545	Vangl1	-1.3	3E-02
NM_172410	Nup93	-1.3	5E-02
NM_173754	Usp43	-1.3	4E-02
NR_002864	Peg13	-1.3	1E-02
NM_020035	Pigo	-1.3	6E-03
ENSMUST00000046011	Nol10	-1.3	5E-02
NM_199448	Fez2	-1.3	2E-03

NM_029945	Smpd4	-1.3	1E-02
NM_173368	Chd6	-1.3	5E-02
ENSMUST00000066497	Zfp191	-1.3	5E-02
ENSMUST00000081542	Kcnd2	-1.3	9E-03
NM_178597	Camk2g	-1.3	3E-03
ENSMUST00000035295	Degs1	-1.3	1E-03
NM_001024911	Septin10	-1.3	1E-02
NM_183417	Cdk2	-1.3	2E-02
NR_024025	Mtmr2	-1.3	5E-02
ENSMUST00000160860	Klhl3	-1.3	3E-02
ENSMUST00000135088	Ywhaq	-1.3	4E-02
NM_053162	Mrpl34	-1.3	5E-02
NM_009311	Tac1	-1.3	5E-02
ENSMUST00000086023	Wbscr17	-1.3	2E-02
NM_172992	Phtf2	-1.3	3E-03
NM_015816	Lsm4	-1.3	3E-02
AK156394	4833438C02Rik	-1.3	3E-02
NR_045279	LOC432958	-1.3	4E-02
NM_008619	Mov10	-1.3	1E-02
NM_001078167	Srsf1	-1.3	4E-02
ENSMUST00000026408	Gdf11	-1.3	6E-05
NM_178710	Sik2	-1.3	8E-03
ENSMUST00000019143	Slc35b4	-1.3	1E-02
NM_001101483	Tmem22	-1.3	4E-02
NM_009086	Polr1b	-1.3	2E-02
NM_152813	Plcd3	-1.3	2E-02
NM_008882	Plxna2	-1.3	5E-02
NR_015588	4932441J04Rik	-1.3	3E-03
NM_009502	Vcl	-1.3	7E-03
ENSMUST00000027358	Bcs1l	-1.3	1E-02
NM_172635	Patl1	-1.3	1E-03
NM_001033441	Alg10b	-1.3	2E-03
NM_153501	Pank2	-1.3	3E-02
ENSMUST00000088295	Chpf2	-1.3	6E-03
NM_001252481	Smad2	-1.3	3E-02
ENSMUST00000113234	Grsf1	-1.3	1E-02
NM_027652	Ept1	-1.3	1E-02
ENSMUST00000070597	Retsat	-1.3	3E-02
NM_001039551	Cnnm3	-1.3	2E-02
ENSMUST00000040182	Ccdc88a	-1.3	2E-02
NM_027878	Dram1	-1.3	2E-02
NM_001081309	Pik3r4	-1.3	1E-02
NM_172288	Nup133	-1.3	9E-03
NM_172302	Cpsf7	-1.3	3E-04
NM_001168668	Fam114a2	-1.3	4E-02
ENSMUST00000009435	Pttg1ip	-1.3	7E-03

NM_010022	Dbt	-1.3	6E-03
NM_001033329	Arhgef9	-1.3	2E-02
ENSMUST00000109235	Stau1	-1.3	5E-02
ENSMUST00000016569	Pds5b	-1.3	4E-03
NM_001159361	Dip2b	-1.3	4E-03
NM_028546	1700066M21Rik	-1.3	4E-02
NM_001177877	1700108M19Rik	-1.3	2E-02
NM_009323	Tbx15	-1.3	3E-02
NM_019920	Lamtor3	-1.3	8E-03
NM_198110	Gnl3l	-1.3	3E-02
NM_175538	E130304F04Rik	-1.3	4E-02
ENSMUST00000003369	Plag1	-1.3	4E-02
NM_145743	Lace1	-1.3	5E-02
NM_029363	2010109A12Rik	-1.3	3E-02
ENSMUST00000043237	Trp53inp2	-1.3	4E-05
ENSMUST00000152594	Zbtb38	-1.3	5E-02
NM_001047604	Ttc21b	-1.3	5E-02
NM_016913	Porcn	-1.3	4E-02
ENSMUST00000032969	Pold3	-1.3	2E-02
ENSMUST00000170094	LOC100044625	-1.3	5E-02
NM_173788	Npr2	-1.3	1E-02
NR_002891	Gm5512	-1.3	4E-02
NM_001164441	Ankrd33b	-1.3	1E-02
NM_026566	9430023L20Rik	-1.3	8E-03
NM_018878	Paxip1	-1.3	9E-03
NM_029752	Bri3bp	-1.3	3E-02
NM_001162941	Mapre2	-1.3	2E-02
NM_026666	Ubn1	-1.3	5E-02
ENSMUST00000041317	Ammecr1	-1.3	5E-03
NM_177025	Cobll1	-1.3	2E-03
NM_024181	Dnajc10	-1.3	2E-02
NM_001115009	Synrg	-1.3	2E-02
ENSMUST00000149740	Hif1a	-1.3	3E-02
NM_028840	Armc1	-1.3	3E-02
NM_001199272	Gopc	-1.3	3E-02
NM_028150	Supt7l	-1.3	3E-02
NM_001166669	Gemin5	-1.3	4E-02
NM_153057	Nomo1	-1.3	5E-03
NM_008042	Fpr3	-1.3	4E-02
NM_001001493	BC056474	-1.3	3E-03
NM_001042653	Oip5	-1.3	4E-02
NM_029654	Atg2b	-1.3	9E-03
ENSMUST00000165640	Zfp113	-1.3	2E-03
NM_001178058	Txnrd3	-1.3	1E-02
ENSMUST00000071592	Prmt7	-1.3	2E-02
ENSMUST00000023467	Pak2	-1.3	2E-02

ENSMUST00000135383	Engase	-1.3	3E-02
NM_001146048	Lrrc1	-1.3	1E-02
NM_026993	Ddah1	-1.3	3E-02
ENSMUST00000163832	Gbe1	-1.3	3E-02
ENSMUST00000172278	Chrm2	-1.3	1E-02
NM_001199321	Zfp94	-1.3	4E-02
NM_009390	Tll1	-1.3	4E-02
NM_009752	Glb1	-1.3	4E-03
ENSMUST00000110835	Elf1	-1.3	5E-02
ENSMUST00000039571	2410004B18Rik	-1.3	2E-02
NM_001024846	Zfp62	-1.3	3E-03
BC118515	4930578C19Rik	-1.3	4E-02
ENSMUST00000102521	Rap1gap2	-1.3	3E-02
NM_146179	Zfp418	-1.3	4E-02
NM_146001	Hip1	-1.3	2E-02
ENSMUST00000133157	Polr1e	-1.3	3E-02
NM_172740	Zfp420	-1.3	3E-02
NM_133949	Ptov1	-1.3	2E-02
NM_008515	Lrrfip1	-1.3	4E-03
ENSMUST00000046807	Slc16a9	-1.3	2E-02
NM_148943	Usp9y	-1.3	1E-02
NM_001128084	Arhgap21	-1.3	1E-03
NM_198296	9130011E15Rik	-1.3	2E-02
NM_178615	Rgmb	-1.3	4E-02
ENSMUST00000116259	Mtap7	-1.3	5E-03
NM_001042407	Pex10	-1.3	3E-02
NM_001162538	Odf2l	-1.3	3E-03
NM_008594	Mfge8	-1.3	4E-02
NM_175214	Kif27	-1.3	2E-02
NM_027007	Zfp397	-1.3	5E-03
NM_178142	Lcorl	-1.3	2E-02
NR_033782	4921531C22Rik	-1.3	4E-02
NM_201368	Xkr8	-1.3	4E-02
NM_008604	Mme	-1.3	2E-02
ENSMUST00000114952	Galnt11	-1.3	3E-02
NM_026341	Nudt13	-1.3	4E-02
NM_027952	1700008I05Rik	-1.3	5E-02
ENSMUST00000068262	Nt5c1a	-1.3	7E-03
NM_134084	Ppif	-1.3	3E-03
NM_001098231	Pdp1	-1.3	3E-02
NM_146090	Zadh2	-1.3	1E-02
NM_013669	Snap91	-1.3	2E-02
NM_172806	Btbd7	-1.3	7E-03
ENSMUST00000075540	Mcm9	-1.3	2E-02
NM_027081	Fam116b	-1.3	2E-02
NM_001033172	Rab11fip2	-1.3	3E-03

NR_002687	Gm5424	-1.3	2E-02
NM_001110100	Banp	-1.3	2E-02
ENSMUST00000091144	Elf2	-1.3	3E-02
ENSMUST00000003152	Stk11	-1.3	1E-02
ENSMUST00000023214	Dgat1	-1.3	9E-03
ENSMUST00000077938	Haghl	-1.3	5E-02
NM_023794	Etv5	-1.3	5E-02
NM_010832	Msl3	-1.3	4E-02
ENSMUST00000029106	Zbtb46	-1.3	3E-02
NM_018830	Asah2	-1.3	3E-02
NM_001136076	P4ha2	-1.3	2E-02
NM_021886	Cenph	-1.3	2E-02
NM_013862	Rabgap1l	-1.3	5E-03
ENSMUST00000112787	Gm3099	-1.3	2E-02
NM_145975	Ddx46	-1.3	8E-03
ENSMUST00000066279	Sh3bp4	-1.3	2E-03
NM_011669	Usp12	-1.3	2E-02
NM_009204	Slc2a4	-1.3	2E-02
ENSMUST00000031728	Pop7	-1.3	8E-03
NM_021422	Dnaja4	-1.3	2E-02
NM_011279	Rnf7	-1.3	2E-02
NM_028048	Slc25a35	-1.3	3E-02
NM_009433	Tspyl1	-1.3	1E-02
NM_001004143	Usp22	-1.3	4E-02
ENSMUST00000028250	Mrrf	-1.3	2E-02
NR_004446	H2-K2	-1.3	5E-02
NM_030114	Herc4	-1.3	2E-02
ENSMUST00000084535	Fam123b	-1.3	8E-03
NM_010411	Hdac3	-1.3	4E-03
ENSMUST00000113738	N4bp2	-1.3	3E-02
ENSMUST00000111569	Caprin2	-1.3	5E-02
NM_194462	Akap9	-1.3	7E-04
ENSMUST00000124549	Comtd1	-1.3	2E-02
ENSMUST00000025166	Cdh2	-1.3	2E-03
NM_152134	Homer1	-1.3	6E-03
NM_183262	Stk35	-1.3	3E-02
NM_146424	Olfr888	-1.3	8E-03
NR_027829	Gm10638	-1.3	6E-04
NM_028127	Frmd6	-1.3	2E-03
ENSMUST00000026324	Acot9	-1.3	2E-02
ENSMUST00000065767	Kdelc1	-1.3	4E-02
AK006644	1700039M10Rik	-1.3	2E-03
NM_009367	Tgfb2	-1.3	3E-02
ENSMUST00000060444	Zfp3	-1.3	4E-02
NM_008071	Gabrb3	-1.3	3E-02
NM_001081557	Camta1	-1.3	4E-03

NM_011358	Srsf2	-1.3	3E-02
NR_045036	Khdrbs1	-1.3	9E-03
NM_001081247	Polr3a	-1.3	4E-02
AK018906	1700084C01Rik	-1.3	4E-02
NM_028230	Shmt2	-1.3	2E-02
ENSMUST00000079869	Znrf2	-1.3	5E-03
NM_207619	V1rd19	-1.3	5E-03
ENSMUST00000015391	Nipsnap3b	-1.3	8E-03
NM_172778	Maob	-1.3	4E-02
NM_001112703	Abl1	-1.3	3E-02
NM_023842	Dsp	-1.3	5E-03
NM_028198	Xpo5	-1.3	9E-04
NM_010324	Got1	-1.3	1E-03
XR_142182	LOC100862136	-1.3	3E-02
NR_026942	E330013P04Rik	-1.3	4E-02
NM_001159907	Gm17296	-1.3	4E-02
ENSMUST00000038108	Ndufc1	-1.3	5E-02
NM_007868	Dmd	-1.3	6E-03
ENSMUST00000136822	B3gnt9-ps	-1.3	4E-02
NM_153515	Ammecr1l	-1.3	3E-02
NM_018814	Pcnx	-1.3	6E-04
NM_130892	Rtn4ip1	-1.3	1E-02
NM_016769	Smad3	-1.3	3E-02
NR_027375	Ythdf3	-1.3	4E-03
NM_153583	Atg4d	-1.3	3E-03
NM_001099276	Pik3c2b	-1.3	1E-02
NM_153129	Pacs1	-1.3	2E-02
ENSMUST00000024858	Galnt14	-1.3	9E-03
BC023836	B230354K17Rik	-1.3	1E-02
NM_019427	Epb4.1l4b	-1.3	1E-02
ENSMUST00000066451	Lrp2bp	-1.3	5E-02
NM_146157	C230096C10Rik	-1.3	4E-02
NM_021543	Pcdh8	-1.3	4E-02
AK085917	2610209C05Rik	-1.3	4E-02
NM_001102611	Smyd4	-1.3	2E-02
ENSMUST00000063517	Spats2	-1.3	3E-02
X00438	Tcrb-J	-1.3	3E-02
NM_022000	Gnas	-1.3	3E-03
ENSMUST00000019470	Psme3	-1.3	7E-03
NM_009090	Polr2c	-1.3	7E-03
NM_001142580	Vipar	-1.3	4E-02
NM_001080706	Btaf1	-1.3	4E-02
NM_133983	Cd276	-1.3	3E-03
ENSMUST00000048026	Hoxa11	-1.3	4E-02
NM_001080769	Uhrf1bp1	-1.3	2E-03
NM_009211	Smarcc1	-1.3	2E-02

NM_007513	Slc7a1	-1.3	5E-02
ENSMUST00000088896	Tmcc1	-1.3	4E-02
NM_144868	Pcnxl3	-1.3	2E-02
NM_001113518	Arhgef7	-1.3	4E-02
ENSMUST00000093469	Psd3	-1.3	4E-02
NM_025780	Thap2	-1.3	3E-02
NM_019998	Alg2	-1.3	3E-02
NM_028597	Thoc3	-1.3	1E-02
NM_025730	Lrrk2	-1.3	3E-03
NM_207625	Acsl4	-1.3	3E-02
NM_172394	Nup88	-1.3	1E-02
ENSMUST00000124334	Mus81	-1.3	1E-02
NM_009423	Traf4	-1.3	2E-02
NM_028227	Brap	-1.3	3E-02
ENSMUST00000057423	Ppapdc3	-1.3	4E-02
NM_175121	Slc38a2	-1.3	8E-03
BC060272	Nrxn2	-1.3	3E-02
NM_023908	Slco3a1	-1.3	2E-02
ENSMUST00000037141	Setd7	-1.3	2E-02
NM_007806	Cyba	-1.3	5E-02
NM_026111	Qpctl	-1.3	1E-02
NM_015774	Ero1l	-1.3	4E-02
NM_144731	Galnt7	-1.3	3E-02
NM_001136064	Bscl2	-1.3	3E-02
ENSMUST00000080368	Atp8a2	-1.3	4E-02
XR_106545	Gm19894	-1.3	1E-02
NM_001081298	Lphn2	-1.3	2E-02
ENSMUST00000045281	Smg6	-1.3	3E-02
NM_011705	Vrk1	-1.3	5E-02
ENSMUST00000031411	Aldh2	-1.3	2E-03
ENSMUST00000033621	Gla	-1.3	1E-02
BC071241	9430016H08Rik	-1.3	3E-02
NM_001256311	E030018B13Rik	-1.3	3E-02
NM_001177534	Gm15881	-1.3	4E-02
NM_001135559	Sos2	-1.3	4E-03
XM_003086546	LOC674392	-1.3	8E-03
NM_172443	Tbc1d16	-1.3	9E-03
NM_001168535	Cdadc1	-1.3	3E-02
NM_010154	Erbb4	-1.3	3E-02
NM_025382	Tmem57	-1.3	4E-02
ENSMUST00000115238	Ahcyl2	-1.3	1E-03
NM_001252463	Mlst8	-1.3	3E-02
ENSMUST00000032429	Med21	-1.3	3E-02
NM_001081426	Dip2c	-1.3	8E-03
NM_001112729	Tti2	-1.3	4E-02
ENSMUST00000164095	Herc2	-1.3	1E-02

AK006331	1700025D23Rik	-1.3	5E-02
NM_026254	Tbc1d23	-1.3	4E-03
ENSMUST00000119129	Cdon	-1.3	2E-02
NM_010475	Hsd17b1	-1.3	1E-02
XM_003689230	Gm5942	-1.3	4E-02
NM_001013028	AI597468	-1.3	4E-02
ENSMUST00000026076	Gfra1	-1.3	3E-02
NM_173378	Trp53bp2	-1.3	2E-02
NR_002928	Gm1943	-1.3	6E-03
NR_040638	4930565A17	-1.3	3E-02
ENSMUST00000067530	Vapb	-1.3	5E-02
NM_027134	Mtfmt	-1.3	1E-02
NR_030677	BC025920	-1.3	4E-02
NM_172644	Dars2	-1.3	3E-02
NM_146807	Olfr136	-1.3	1E-02
NM_027834	9130008F23Rik	-1.3	3E-02
ENSMUST00000029049	Chmp4c	-1.3	9E-03
ENSMUST00000037918	Tmem33	-1.3	2E-02
NM_178798	Slc7a6	-1.3	2E-02
NM_011062	Pdpk1	-1.3	4E-02
NM_183150	BC003965	-1.3	4E-02
NM_011218	Ptprs	-1.3	1E-03
ENSMUST00000102849	Usp20	-1.3	1E-03
NM_001081363	Cenpf	-1.3	2E-02
NM_026154	Mrpl10	-1.3	2E-02
NM_010561	Ilf3	-1.3	2E-02
NM_001111027	Runx1t1	-1.3	3E-02
NR_045008	Gm20300	-1.3	7E-03
ENSMUST00000055104	Tceal1	-1.3	3E-02
NM_026404	Slc35a4	-1.3	3E-03
NM_024188	Oxct1	-1.3	1E-02
NM_021350	Chml	-1.3	3E-02
NM_001253706	Septin6	-1.3	2E-02
NM_133857	Usp53	-1.3	2E-02
ENSMUST00000105364	Ndufs7	-1.3	1E-03
NM_025362	Dnajc30	-1.3	2E-02
NM_145590	BC017158	-1.3	4E-02
NM_001110843	Cacna2d1	-1.3	1E-02
NM_172404	Ccbl1	-1.3	3E-02
ENSMUST00000035106	Slc25a38	-1.3	1E-02
NM_001033271	Tmem55b	-1.3	3E-02
NM_026102	Daam1	-1.3	2E-02
NM_021881	Qk	-1.3	4E-03
NM_027854	0610007L01Rik	-1.3	6E-03
NM_173026	Zbtb11	-1.3	3E-02
NM_001038587	Adar	-1.3	3E-02

NM_001198860	Ctbp1	-1.3	2E-03
NM_001168297	Fbxo30	-1.3	5E-02
NM_172682	Fam160a1	-1.3	4E-02
NM_173779	Sowahd	-1.3	2E-02
ENSMUST00000040576	Parm1	-1.3	3E-03
ENSMUST00000027384	Atic	-1.3	6E-03
NM_027491	Rragd	-1.2	5E-02
NM_011514	Suv39h1	-1.2	1E-02
ENSMUST00000034475	Snx19	-1.2	2E-02
NM_025826	Acadsb	-1.2	5E-03
NM_001001984	Kdm2a	-1.2	7E-04
NM_001081293	Rprd2	-1.2	9E-03
NM_001033439	Lrch1	-1.2	4E-02
NM_008679	Ncoa3	-1.2	3E-02
NM_001199043	Lgals8	-1.2	5E-03
ENSMUST00000096744	Myadm	-1.2	4E-02
NM_022323	Moap1	-1.2	2E-02
NM_019653	Wsb1	-1.2	3E-02
ENSMUST00000160696	Phc1	-1.2	3E-02
NM_001199141	Zmynd11	-1.2	2E-02
NM_172874	Podn	-1.2	2E-02
NM_021607	Ncstn	-1.2	3E-02
ENSMUST00000109353	Tubgcp6	-1.2	2E-02
NM_144888	Mavs	-1.2	3E-02
ENSMUST00000080598	Bre	-1.2	5E-02
ENSMUST00000002391	Tm9sf1	-1.2	3E-02
ENSMUST00000057551	Slbp	-1.2	1E-02
NM_134054	1110002B05Rik	-1.2	2E-02
NM_030132	Utp23	-1.2	2E-02
NM_011264	Rev3l	-1.2	4E-02
NM_027895	Ulk3	-1.2	1E-03
NM_130861	Slco1a5	-1.2	3E-02
NM_145385	Mlf2	-1.2	4E-04
NM_130796	Snx18	-1.2	2E-02
NM_008772	P2ry1	-1.2	7E-03
NM_009497	Vamp2	-1.2	2E-02
ENSMUST00000067101	Kcnj3	-1.2	4E-02
NM_027931	Tars2	-1.2	4E-02
NM_001081203	Sbno1	-1.2	7E-04
ENSMUST00000050668	Zfp770	-1.2	4E-02
NM_019654	Socs5	-1.2	4E-02
ENSMUST00000031077	Zcchc4	-1.2	5E-02
AK140616	Gm15983	-1.2	5E-02
ENSMUST00000164416	Pcid2	-1.2	1E-02
NM_008847	Pip5k1a	-1.2	4E-02
NM_153801	Tecrl	-1.2	2E-03

ENSMUST00000091609	Cltb	-1.2	1E-02
NM_018763	Chst2	-1.2	1E-02
BC031781	BC031781	-1.2	3E-02
NM_018831	Dclre1a	-1.2	1E-02
NM_001033430	Jhdm1d	-1.2	2E-02
NM_054045	Hist2h3c2	-1.2	4E-02
NM_133687	Cxxc5	-1.2	3E-03
NM_001128151	Cecr2	-1.2	4E-03
NM_010124	Eif4ebp2	-1.2	5E-02
NM_198429	Nfatc1	-1.2	4E-02
ENSMUST00000029116	Pcmtd2	-1.2	5E-03
NM_178376	Rraga	-1.2	3E-02
NM_173406	Jazf1	-1.2	4E-02
NM_031169	Kcnmb1	-1.2	5E-02
ENSMUST00000136348	Bcorl1	-1.2	3E-02
NM_021494	Dennd5a	-1.2	2E-02
NM_013507	Eif4g2	-1.2	6E-03
NM_177128	Iqcb1	-1.2	2E-02
NM_001042421	Kntc1	-1.2	2E-02
NR_030711	2210403K04Rik	-1.2	3E-03
AK014034	3110009M11Rik	-1.2	3E-02
NM_026389	Poldip2	-1.2	1E-02
ENSMUST00000112624	Oxsm	-1.2	2E-02
NM_133705	Pycr2	-1.2	4E-02
NM_009536	Ywhae	-1.2	1E-02
ENSMUST00000096495	Med14	-1.2	3E-02
NM_027920	March8	-1.2	4E-02
NM_001146349	Rnf217	-1.2	2E-02
BC028457	1500032L24Rik	-1.2	1E-02
NM_177324	Sbf2	-1.2	2E-02
NM_021356	Gab1	-1.2	5E-02
NM_026556	Dynll2	-1.2	2E-02
NM_009107	Rxrg	-1.2	4E-02
NM_022813	Scamp2	-1.2	3E-02
NM_001025309	Pja2	-1.2	1E-02
NM_009358	Ppp2r5d	-1.2	4E-02
NM_178363	Ylpm1	-1.2	1E-03
ENSMUST00000113063	Il1rapl2	-1.2	1E-02
NM_001111121	Ccdc6	-1.2	4E-02
NM_178743	Slc26a11	-1.2	2E-02
NM_010799	Minpp1	-1.2	3E-02
NM_001033474	Atxn7l3b	-1.2	4E-03
NM_019739	Foxo1	-1.2	4E-02
NM_172514	Tmem71	-1.2	2E-02
ENSMUST00000105502	Foxo3	-1.2	3E-02
NM_001024604	Ankrd28	-1.2	1E-02

NM_144874	Cox15	-1.2	1E-02
NM_017367	Ccni	-1.2	2E-02
NM_018808	Dnajb1	-1.2	4E-02
NM_027901	Gtf3c2	-1.2	4E-02
NM_028871	Hnrnpr	-1.2	5E-02
ENSMUST00000114548	Cadm1	-1.2	2E-02
NM_001163475	Zfp746	-1.2	4E-02
NM_138681	Bcas3	-1.2	1E-02
NM_001164679	Ano8	-1.2	2E-02
ENSMUST00000117160	Cdh13	-1.2	5E-02
ENSMUST00000136872	Mtch2	-1.2	2E-02
NM_001033466	Zbtb2	-1.2	4E-02
NM_008715	Ints6	-1.2	2E-02
NM_172282	Tmco3	-1.2	1E-02
NM_007772	Hivep1	-1.2	5E-03
NM_001159634	Prrc2b	-1.2	1E-04
AK016136	4930555O08Rik	-1.2	4E-02
NM_027083	Lyzl6	-1.2	4E-02
ENSMUST00000028087	Ppp6c	-1.2	4E-02
NM_001001983	Pi4ka	-1.2	1E-02
NR_045269	1700011J10Rik	-1.2	3E-02
NM_001039123	Defb18	-1.2	4E-02
NM_172606	March6	-1.2	8E-03
NM_020575	March7	-1.2	4E-02
NM_001039156	Triobp	-1.2	4E-02
NM_001033385	D630037F22Rik	-1.2	3E-02
NM_144920	Plekha5	-1.2	2E-02
ENSMUST00000040179	Ttll5	-1.2	1E-02
NM_018884	Pdzrn3	-1.2	6E-03
NM_001201413	Apbb2	-1.2	4E-03
NM_133750	Fam118a	-1.2	1E-02
NM_008086	Gas1	-1.2	4E-02
NM_178403	Pus7	-1.2	9E-03
NM_008349	Il10rb	-1.2	4E-02
NM_001253690	Patz1	-1.2	3E-02
NM_019730	Nme3	-1.2	3E-02
NM_016709	Auh	-1.2	3E-02
NM_025950	Cdc37l1	-1.2	5E-03
NM_030695	Lrba	-1.2	5E-03
ENSMUST00000122010	Anapc7	-1.2	4E-02
NM_026856	Zfp644	-1.2	2E-02
NM_001039552	2210404J11Rik	-1.2	5E-02
NM_013906	Adamts8	-1.2	2E-02
ENSMUST00000115578	Ufd1l	-1.2	4E-03
NM_001174078	Smarca4	-1.2	1E-03
NR_033514	2310015A10Rik	-1.2	4E-02

NM_133987	Slc6a8	-1.2	3E-02
NM_001079932	Trim72	-1.2	3E-02
NM_007415	Parp1	-1.2	3E-02
NM_001037923	Lekr1	-1.2	4E-03
AK082638	Sf3b3	-1.2	5E-02
NM_173756	Lin52	-1.2	8E-03
NM_024214	Tomm20	-1.2	4E-02
NM_182992	Mypn	-1.2	9E-04
ENSMUST00000057561	Wwc2	-1.2	2E-02
NM_011100	Prkacb	-1.2	1E-02
NM_001190852	Pdlim5	-1.2	3E-02
NM_001256112	Fam54b	-1.2	4E-02
NM_027485	Med26	-1.2	9E-03
NM_026748	Ints1	-1.2	4E-03
NM_009532	Xrcc1	-1.2	5E-02
NM_001081057	Tecpr2	-1.2	1E-02
NM_011211	Ptprd	-1.2	4E-02
ENSMUST00000103033	2310067B10Rik	-1.2	2E-02
NM_177648	Dolk	-1.2	1E-02
NM_001024560	Snx32	-1.2	5E-02
ENSMUST00000003044	Pnkp	-1.2	4E-03
ENSMUST00000039476	Arhgef11	-1.2	4E-03
NM_145432	Heatr6	-1.2	2E-02
NM_054093	Ube3b	-1.2	9E-03
NM_001163502	C130039O16Rik	-1.2	2E-02
NM_001033338	Rimbp3	-1.2	8E-03
NM_001017985	C2cd3	-1.2	8E-03
NM_013477	Atp6v0d1	-1.2	6E-03
NM_001037749	Slc22a14	-1.2	4E-02
NM_013556	Hprt	-1.2	3E-02
NM_178693	Coq4	-1.2	2E-02
NM_173189	Mcph1	-1.2	2E-02
ENSMUST00000017332	Ccdc56	-1.2	2E-02
ENSMUST00000105809	Ubxn10	-1.2	4E-02
NM_011104	Prkce	-1.2	6E-03
NM_133770	Adck4	-1.2	4E-02
NM_026175	Sf3a1	-1.2	1E-02
NM_172871	Klhl9	-1.2	9E-03
NM_030252	BC003266	-1.2	3E-02
NM_001025067	Lrig2	-1.2	2E-02
NM_172260	Cep68	-1.2	4E-02
NM_007896	Mapre1	-1.2	4E-02
NM_029857	Tmco4	-1.2	4E-02
ENSMUST00000004683	Mcoln1	-1.2	1E-02
ENSMUST00000071001	Tmem80	-1.2	2E-02
NM_001110209	Lnp	-1.2	2E-02

NM_010433	Hipk2	-1.2	8E-03
ENSMUST00000107692	Trim2	-1.2	1E-02
ENSMUST00000159592	Ssh1	-1.2	2E-02
ENSMUST00000147337	Mapkap1	-1.2	2E-02
NM_008866	Lypla1	-1.2	7E-03
NM_145824	Ranbp10	-1.2	5E-02
NM_001115010	Lin54	-1.2	3E-02
ENSMUST00000091259	Slc7a14	-1.2	2E-02
NM_172903	Man2a2	-1.2	2E-02
ENSMUST00000021802	Cap2	-1.2	2E-02
NM_178659	Jmjd4	-1.2	3E-02
NM_146173	Tspan33	-1.2	2E-02
NM_025903	Ifrd2	-1.2	5E-03
NM_001045536	Zzef1	-1.2	4E-02
ENSMUST00000040735	Amdhd2	-1.2	3E-02
NM_019711	Rbms2	-1.2	6E-04
NM_175413	Lrrc39	-1.2	3E-03
NM_025516	Ergic3	-1.2	2E-02
NM_001039644	Edem3	-1.2	9E-03
NM_001253822	Irx3	-1.2	2E-02
NR_045070	4930592A05Rik	-1.2	1E-02
NM_027326	Mllt3	-1.2	4E-02
NM_207659	Hook3	-1.2	5E-02
NM_001163852	Tbc1d24	-1.2	2E-02
ENSMUST00000031594	Sdsl	-1.2	6E-03
ENSMUST00000106497	Grb2	-1.2	1E-02
NM_172429	Smndc1	-1.2	4E-02
NM_008828	Pgk1	-1.2	4E-02
NM_007862	Dlg1	-1.2	4E-02
NM_153563	Fam40a	-1.2	2E-02
ENSMUST00000049544	Ccdc141	-1.2	4E-02
NM_134079	Adk	-1.2	4E-02
ENSMUST00000018470	Ywhab	-1.2	7E-03
NM_001199245	Kcnip4	-1.2	4E-02
ENSMUST00000154647	Phyhd1	-1.2	5E-02
BC130272	Fam65c	-1.2	3E-02
NM_139144	Ogt	-1.2	2E-02
